# Synthesis of Functionalized 1*H*-Imidazoles via Denitrogenative Transformation of 5-Amino-1,2,3-Triazoles

**DOI:** 10.3390/molecules30071401

**Published:** 2025-03-21

**Authors:** Pavel S. Gribanov, Anna N. Philippova, Alexander F. Smol’yakov, Diana N. Tukhvatullina, Viktoria A. Vlasova, Maxim A. Topchiy, Andrey F. Asachenko, Sergey N. Osipov

**Affiliations:** 1 A. N. Nesmeyanov Institute of Organoelement Compounds, Russian Academy of Sciences, 28/1 Vavilova Str., 119334 Moscow, Russia; anyfil96@gmail.com (A.N.P.); rengenhik@gmail.com (A.F.S.); dianatukhvatullina123@mail.ru (D.N.T.); vlasova_vika2006@mail.ru (V.A.V.); 2 D. I. Mendeleev University of Chemical Technology of Russia, 9 Miusskaya pl., 125047 Moscow, Russia; 3 A.V. Topchiev Institute of Petrochemical Synthesis, Russian Academy of Sciences, Leninskiy Prospect 29, 119991 Moscow, Russia; maxtopchiy@ips.ac.ru (M.A.T.); aasachenko@ips.ac.ru (A.F.A.)

**Keywords:** imidazoles, 5-amino-1,2,3-triazoles, denitrogenative transformation, pyridotriazoles

## Abstract

An efficient access to novel 2-substituted 1*H*-imidazole derivatives was developed based on acid-mediated denitrogenative transformation of 5-amino-1,2,3-triazole derivatives available through dipolar azide−nitrile cycloaddition (DCR). The proposed approach includes intramolecular cyclization of 5-amino-4-aryl-1-(2,2-diethoxyethyl) 1,2,3-triazoles followed by triazole ring opening and insertion of in situ formed carbene intermediate into the O-H bond of different alcohols under acidic conditions.

## 1. Introduction

Nitrogen-containing compounds, particularly imidazoles, are highly significant in heterocyclic chemistry due to their diverse chemical properties and applications in agriculture and pharmacology [1,2] as well as in functional materials [3,4,5]. The imidazole core is an essential structural element of many compounds whose derivatives are widely used in organic synthesis [6,7,8], e.g., as directing groups in transition metal-catalyzed C-H activation reactions [9,10] and as NHC ligands in homogeneous catalysis [11,12,13].

The transition metal-catalyzed ring opening of easily accessible 1,2,3-triazoles is well-known for the generation of metallocarbene intermediates, and they undergo a wide range of synthetically useful transformations like cycloadditions, ring expansions, ylide formation and so forth to access different heterocyclic compounds [14,15,16,17,18,19,20], including imidazoles [2,21,22,23]. However, such reactions require the use of precious Rh-, Co-, or Ni-complexes as the catalysts. Therefore, the development of alternative strategies, especially cheap metal-free methods of the 1,2,3-triazole ring opening to afford important heterocyclic derivatives, is highly desirable.

It is known that the stability of the 1,2,3-triazole core is considerably affected by the electronic nature of the substituents at the N1, C4 and C5 atoms [23]. The presence of a donor amino group at position C5 [24] and acceptor substituents (e.g., sulfonyl, perfluoroalkyl or carbonyl groups) at position N1 [21,25,26,27] significantly increases the polarity of the N-N bond, as a result of which this 1,2,3-triazole can exist in equilibrium with the corresponding tautomeric diazoamidine. It is also known that some conjugated 1,2,3-triazole-containing heterocyclic systems, such as pyridotriazoles [17,28,29] or in situ generated triazolobenzoxazoles [29,30], have a high potential for electrocyclic ring opening. The formation of systems based on the corresponding diazo intermediates opens up possibilities for their further modification via transannulation to afford a new nitrogen-containing heterocyclic ring by trapping these intermediates with different reagents [20,28,31,32,33].

**Scheme 1:**
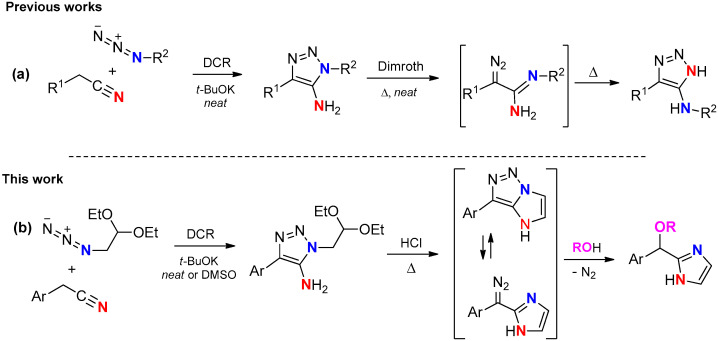
Synthetic approaches to (**a**) 5-amino-1,2,3-triazole derivatives and (**b**) 1*H*-imidazoles.

Based on the above-mentioned concepts and our own studies in the synthesis of functional 5-amino-1,2,3-triazoles through a combination of the azide-nitrile dipolar cycloaddition (DCR) reaction with the Dimroth rearrangement (Scheme 1a) [34,35,36], here we report on a novel efficient approach to biologically relevant 2-substituted imidazole derivatives via acid-mediated denitrogenative transformation of the 5-amino-1,2,3-triazole ring followed by O-H carbene insertion (Scheme 1b). To the best of our knowledge, this type of transformation has not been previously investigated for 5-amino-1,2,3-triazole derivatives.

## 2. Results

### Synthesis

**Scheme 2:**
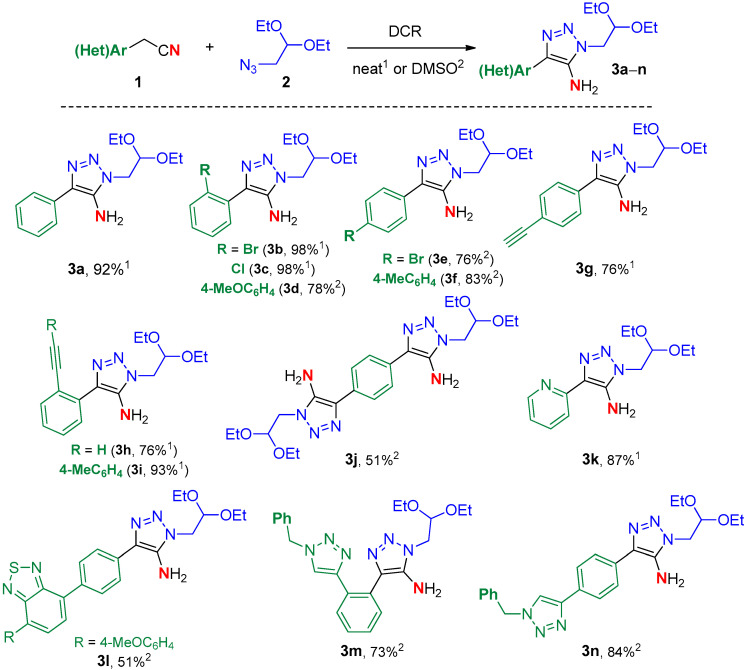
Synthesis of 5-amino-1-(2,2-diethoxyethyl)-1,2,3-triazole derivatives **3a**–**n**. Reaction conditions: ^1^ **1** (0.5 mmol), **2** (1.1 equiv.), KOtBu (0.1 equiv.), neat, 25 °C, 3 h; ^2^ **1** (0.5 mmol), **2** (3.0 equiv.), KOtBu (0.5 equiv.), DMSO, 70 °C, 3 h.

At the beginning of our study, a new series of starting 5-amino-1-(2,2-diethoxyethyl) 1,2,3-triazoles **3** has been prepared via dipolar [3+2] cycloaddition (DCR) of a wide range of aromatic and heteroaromatic acetonitriles **1** with diethyl 2-azidoacetaldehyde diethyl acetal **2** according to an efficient and simple protocol developed in our previous works [34,36]. The method allowed us to easily obtain a number of corresponding 5-amino-1,2,3-triazole derivatives **3a**–**n** with different substituents at the C4 position (Scheme 2). For the reaction of liquid R-CH_2_CN (for the synthesis of **3a**–**c**, **3g**–**i** and **3k**), solvent-free conditions were used [34]; for solid nitriles insoluble in the azide **2**, we used DMSO as a solvent to afford the corresponding products **3d**–**f**, **3j** and **3l**–**n** [36].

**Scheme 3:**
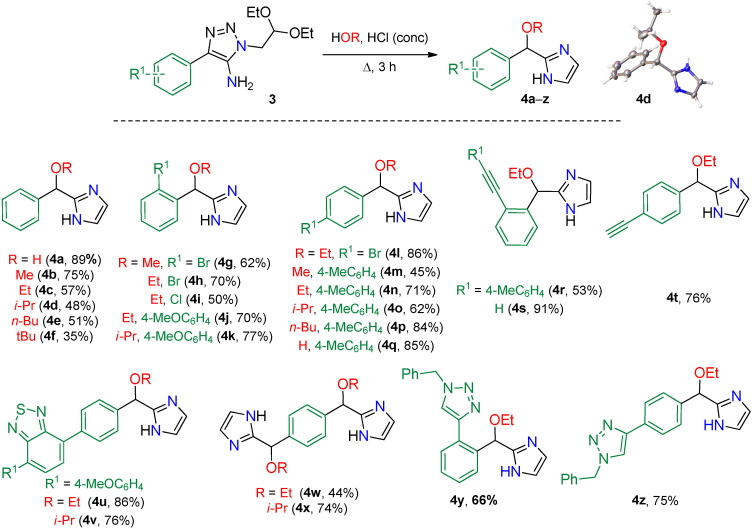
Synthesis of 1*H*-imidazole derivatives **4a**–**z**. Reaction conditions: **3** (0.5 mmol), ROH (10 mL), conc. HCl (0.5 mmol), reflux, 3 h.

The molecules containing vicinal functionalities such as amino- and protected acetal groups are often used in synthetic organic chemistry to furnish nitrogen-containing heterocyclic compounds, in particular pyrrole and indole derivatives, through intermolecular cyclization under acidic conditions [37,38,39,40]. Following this strategy and literature precedent concerning acid hydrolysis of α-(1,2,3-triazole)-substituted acetals [41], 5-amino-1-(2,2-diethoxyethyl)-4-phenyl-1*H*-1,2,3-triazole **3a** was initially hydrolyzed by heating in conc. HCl to determine what would happen after deprotection of the aldehyde function. As a result, it was revealed that the reaction smoothly occurs at 90°C and goes to completion for 3 h to afford (1*H*-imidazol-2-yl)(phenyl)methanol **4a** in 89% yield as a transannulation product (Scheme 3). It should be noted that the compound **4a** has been previously described by Yus’s group [42] via an isoprene-mediated lithiation of protected imidazole derivatives to use as a ligand in a palladium-catalyzed Hiyama reaction [6].

The strategy tested for the preparation of **4a** was further used to react other starting 5-amino-substituted 1,2,3-triazole acetals **3**. After an initial screening of reaction conditions, we found that the best yield of the desired product **4** can be achieved by refluxing of acetals **3** alcohol solutions (for MeOH, EtOH, *i*-PrOH, *n*-BuOH and *t*-BuOH) in the presence of 0.5 mL conc. HCl (see Section 3.4.3). As a result, we have prepared various 2-(alkoxy(hetaryl)methyl) 1*H*-imidazole derivatives **4a**–**z** in good to excellent yields (Scheme 3). The steric and electronic nature of the substituents and their location in the phenyl ring at position C4 do not have a significant effect on the outcome of the reaction. The examples of **4u** and **4v** demonstrate the possibility of incorporating the imidazole core into the benzothiadiazole-based (BTD) luminophores, which are important units of innovative optoelectronic devices [36,43,44].

**Figure 1:**
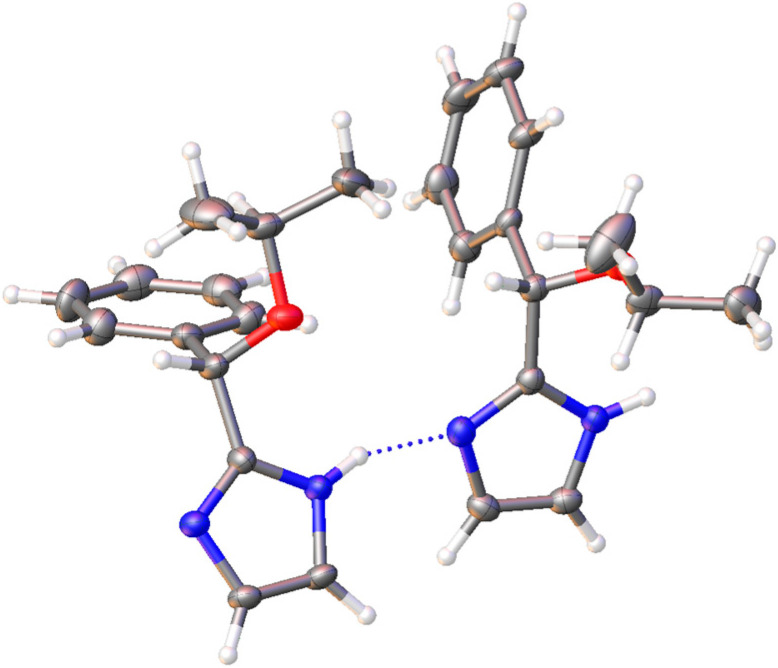
Crystal structure of **4d** (CCDC 2426762) demonstrating intermolecular interactions N2A…N5B of 2.8327(2) and N2A…H5B of 2.0100 A°. Doted line is the standard designation in cristallography for intermolecular contacts.

All compounds obtained were fully characterized by standard physicochemical methods. In ^1^H NMR spectra of imidazole derivatives **4**, the following characteristic signals were observed: the corresponding singlets in the 11.0–12.0 ppm region (1H of the N-H group), 6.5–7.0 ppm (2H at the imidazole double bond) and 5.0–6.0 ppm (1H at the quaternary carbon atom). In addition, the structure of **4d** was unambiguously confirmed by X-ray analysis. Compound **4d** crystallizes in a crystal as two independent molecules (z = 8). Both molecules are in a common position. The formation of a strong intermolecular N(5B)-H(5B)…N(2A) interaction is observed in the crystal (Figure 1).

**Scheme 4:**
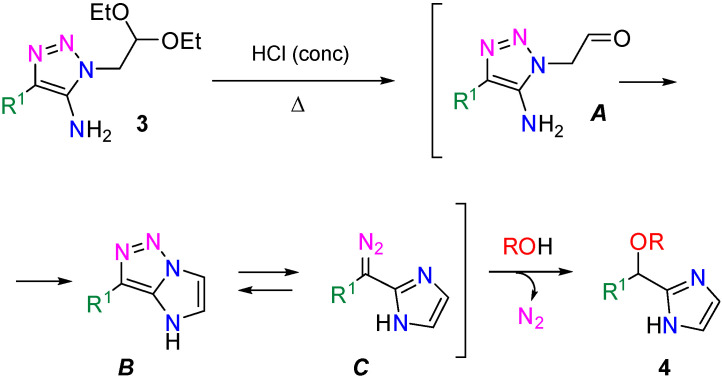
Proposed mechanism of imidazole formation.

A plausible mechanism for the reaction involves an initial hydrolysis of the starting 5-amino-1,2,3-triazoloacetal **3** to the corresponding aldehyde **A**, which can further undergo intramolecular cyclization to imidazotriazole **B** existing in equilibrium with its tautomeric form **C**. The diazo compound **C** then eliminates nitrogen and undergoes the carbene insertion into the O-H bond of the corresponding alcohol to afford the final 2-substituted imidazole **4** (Scheme 4).

**Scheme 5:**
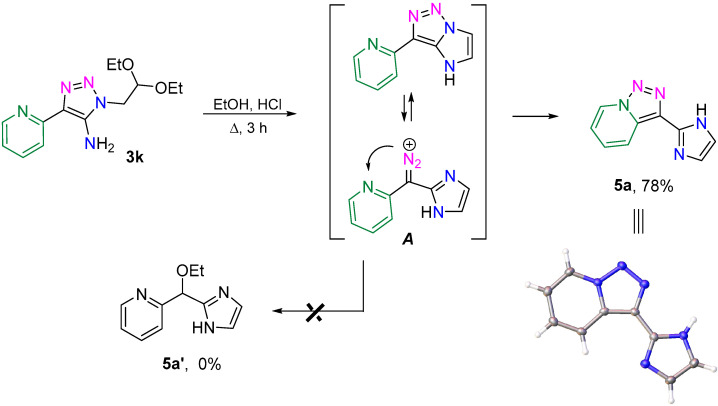
Synthesis of [1,2,3]triazolo[1,5-*a*]pyridine derivative **5a** (CCDC 2426763).

An interesting exception has been found in the case of 4-(pyridin-2-yl)-substituted 1,2,3-triazole **3k** that transforms into the corresponding extremely stable 3-(1*H*-imidazol-2-yl)-[1,2,3]triazolo[1,5-*a*]pyridine **5a** under standard conditions in good yield (Scheme 5). The formation of the expected imidazole-containing ether **5a’** as a result of carbene insertion into the ethanol O-H bond was not detected. It is not excluded that the pyridine ring stabilized diazo intermediate **A** hampers nitrogen elimination due to more favorable intermolecular N-N bond formation. It is worth noting that compounds with a 1,2,3-triazolo-[1,5-a]-pyridine framework can be used in organic and medicinal chemistry for the preparation of biologically relevant molecules [31,45,46].

## 3. Materials and Methods

### 3.1. General Information

All reagents were used as purchased from Sigma-Aldrich. Starting with 2-(4-bromophenyl)acetonitrile [47], 2-(pyridin-2-yl)acetonitrile [48], 2-(4-((trimethylsilyl) ethynyl)phenyl)acetonitrile [49], 2-(4′-methyl-[1,1′-biphenyl]-4-yl)acetonitrile [50], 2-(4-(7-(4-methoxyphenyl)benzo[*c*][1,2,5]thiadiazol-4-yl)phenyl)acetonitrile [36], 2-(2-(*p*-tolylethynyl)phenyl)acetonitrile [51], 2,2′-(1,4-phenylene)diacetonitrile [52] and (*a*NHC)CuCl [53] were synthesized according to published procedures. Analytical data were in accordance with the literature data. Analytical TLC was performed with Merck silica gel 60 F 254 plates (Darmstadt, Germany); visualization was accomplished with UV light or iodine vapors. Chromatography was carried out using Merck silica gel (Kieselgel 60, 0.063–0.200 mm) and petroleum ether/ethyl acetate as an eluent. The ^1^H and ^13^C NMR spectra were recorded on a Varian Inova 400 spectrometer operating at 400 and 101 MHz, respectively. Chemical shifts are given in ppm using residual solvent signals as internal standards. High-resolution mass spectrometry spectra were obtained using an AB Sciex Triple TOF 5600+ (Framingham, MA, USA), which supported different ionization sources. The melting points were determined on a Stuart SMP 10 melting point apparatus (Wertheim, Germany) and are uncorrected.

### 3.2. Crystal Structure Determination

Single-crystal X-ray diffraction experiments were carried out with a Bruker SMART APEX CCD area detector diffractometer (Bruker, Billerica, MA, USA). The crystal was kept at 120 K during data collection. Using Olex2 [54], the structure was solved with the SHELXT [55] structure solution program using intrinsic phasing and refined with the XL [56] refinement package using least squares minimisation.

Crystal Data for **4d** C_13_H_16_N_2_O (*M* = 216.28 g/mol): monoclinic, space group P2_1_/n (no. 14), *a* = 9.9055(7) Å, *b* = 18.8699(14) Å, *c* = 12.9213(10) Å, *β* = 92.299(3), *V* = 2413.3(3) Å^3^, *Z* = 2, *T* = 120 K, μ(MoKα) = 0.077 mm^−1^, *Dcalc* = 1.191 g/cm^3^, 33,287 reflections measured (3.822 ≤ 2Θ ≤ 51.996), 4736 unique (R_int_ = 0.0486, R_sigma_ = 0.0390), which were used in all calculations. The final *R*_1_ was 0.0674 (I > 2σ(I)) and *wR*_2_ was 0.1841.

Crystal Data for **5a** C_9_H_7_N_5_ (*M* = 185.20 g/mol): monoclinic, space group P2_1_/n (no. 14), *a* = 4.1391(3) Å, *b* = 16.4192(14) Å, *c* = 12.1450(9) Å, *β* = 98.262(4), *V* = 816.82(11) Å^3^, *Z* = 4, *T* = 120 K, μ(MoKα) = 0.101 mm^−1^, *Dcalc* = 1.506 g/cm^3^, 8267 reflections measured (4.2 ≤ 2Θ ≤ 51.984), 1584 unique (R_int_ = 0.0338, R_sigma_ = 0.0324), which were used in all calculations. The final *R*_1_ was 0.0339 (I > 2σ(I)) and *wR*_2_ was 0.0859.

Deposition numbers 2426762 (for **4d**) and 2426763 (for **5a**), respectively, contain the supplementary crystallographic data for this paper. These data are provided free of charge by the joint Cambridge Crystallographic Data Centre service www.ccdc.cam.ac.uk/structures (accessed on 11 March 2025).

### 3.3. Preparation and Characterization of Starting Acetonitrile Derivatives

*2-(4′-methoxy-[1,1′-biphenyl]-2-yl)acetonitrile*

The title compound was synthesized according to literature procedure [36] from 2-(2-bromophenyl)acetonitrile and (4-methoxyphenyl)boronic acid as a light-yellow solid (44% yield). ^1^H NMR (400 MHz, Chloroform-*d*) δ 7.56–7.49 (m, 1H, *H*_Ar_), 7.41–7.36 (m, 2H, *H*_Ar_), 7.32–7.27 (m, 1H, *H*_Ar_), 7.22 (d, *J* = 8.6 Hz, 2H, *H*_Ar_), 6.99 (d, *J* = 8.6 Hz, 2H, *H*_Ar_), 3.87 (s, 3H, ArOC*H*_3_), 3.64 (s, 2H, C*H_2_*CN). ^13^C NMR (101 MHz, Chloroform-*d*) δ 159.3, 141.7, 132.3, 130.7, 130.2, 129.0, 128.3, 128.0, 118.5, 116.1, 114.2, 55.4, 22.2. HRMS (ESI) C_15_H_14_NO, *m*/*z*: calc. for [M+H]^+^: 224.1070; found: 224.1070.

*2-(2-((trimethylsilyl)ethynyl)phenyl)acetonitrile*

The title compound was synthesized from 2-(2-bromophenyl)acetonitrile according to literature procedures [57,58] as a light yellow oil (yield 92%). ^1^H NMR (400 MHz, Chloroform-*d*) δ 7.49 (t, *J* = 7.3 Hz, 2H, *H*_Ar_), 7.37 (d, *J* = 7.0 Hz, 1H, *H*_Ar_), 7.29 (t, *J* = 7.4 Hz, 1H, *H*_Ar_), 3.90 (s, 2H, C*H*_2_CN), 0.28 (s, 9H, Si(C*H*_3_)_3_). ^13^C NMR (101 MHz, Chloroform-*d*) δ 132.7, 132.3, 129.4, 128.2, 128.1, 122.8, 101.7, 101.5, 22.8, 0.0. HRMS (APPI) C_13_H_16_NSi, *m*/*z*: calc. for [M+H]^+^: 214.1047; found: 214.1046.

*2-(2-ethynylphenyl)acetonitrile*

The title compound was synthesized from 2-(2-((trimethylsilyl)ethynyl)phenyl) acetonitrile according to literature procedures [57] as a light yellow oil (yield 87%). Analytical data were in accordance with the literature [59]. ^1^H NMR (400 MHz, Chloroform-*d*) δ 7.52 (d, *J* = 7.6 Hz, 1H, *H*_Ar_), 7.49 (d, *J* = 7.7 Hz, 1H, *H*_Ar_), 7.39 (t, *J* = 7.6 Hz, 1H, *H*_Ar_), 7.31 (d, *J* = 7.5 Hz, 1H, *H*_Ar_), 3.91 (s, 2H, C*H*_2_CN), 3.40 (s, 1H, Ar-CC*H*). ^13^C NMR (101 MHz, Chloroform-*d*) δ 133.1, 132.5, 129.7, 128.2, 128.2, 121.7, 117.4, 83.6, 80.5, 22.7.

*2-(4-ethynylphenyl)acetonitrile*

The title compound was synthesized from 2-(4-((trimethylsilyl)ethynyl)phenyl) acetonitrile according to literature procedures [57] as a light yellow oil (yield 96%). Analytical data were in accordance with the literature [49]. ^1^H NMR (400 MHz, Chloroform-*d*) δ 7.50 (d, *J* = 8.2 Hz, 2H, *H*_Ar_), 7.29 (d, *J* = 8.0 Hz, 2H, *H*_Ar_), 3.75 (s, 2H, C*H*_2_CN), 3.11 (s, 1H, Ar-CC*H*). ^13^C NMR (101 MHz, Chloroform-*d*) δ 132.9, 130.6, 128.0, 122.2, 117.4, 82.8, 78.2, 23.7.

*2-(2-(1-benzyl-1H-1,2,3-triazol-4-yl)phenyl)acetonitrile*

The title compound was synthesized according to literature procedure [60]. A screw-cap vial equipped with a magnetic stir bar was charged with 2-(2-ethynylphenyl)acetonitrile (0.75 mmol), benzyl azide (1.5 equiv.) and 1 mol% (*a*NHC)CuCl. The reaction mixture was stirred for 24 h at 40 °C. After 24 h, the reaction mixture was dissolved in dichloromethane and evaporated to dryness in vacuo. Purification by chromatography (eluent—hexane: ethyl acetate 2:1) yielded an analytically pure product as a yellow solid (yield 62%). M.p. 99–101 °C. ^1^H NMR (400 MHz, Chloroform-*d*) δ 7.66 (s, 1H, *H*_trz_), 7.56 (d, *J* = 7.5 Hz, 1H, *H*_Ar_), 7.43–7.31 (m, 8H, *H*_Ar_), 5.59 (s, 2H, ArC*H*_2_), 4.21 (s, 2H, C*H*_2_CN). ^13^C NMR (101 MHz, Chloroform-*d*) δ 147.2, 134.5, 129.8, 129.5, 129.4, 129.3, 129.0, 129.0, 128.5, 128.4, 128.2, 121.9, 118.4, 54.4, 22.9. HRMS (ESI) C_17_H_15_N_4_, *m*/*z*: calc. for [M+H]^+^: 275.1291; found: 275.1293.

*2-(4-(1-benzyl-1H-1,2,3-triazol-4-yl)phenyl)acetonitrile*

The title compound was synthesized according to literature procedure [60]. A screw-cap vial equipped with a magnetic stir bar was charged with 2-(4-ethynylphenyl)acetonitrile (0.75 mmol), benzyl azide (1.5 equiv.) and 1 mol% (*a*NHC)CuCl. The reaction mixture was stirred for 24 h at 40 °C. After 24 h, the reaction mixture was dissolved in dichloromethane and evaporated to dryness in vacuo. Purification by chromatography (eluent—hexane: ethyl acetate 2:1) yielded an analytically pure product as a light yellow solid (yield 57%). M.p. 166–167 °C. ^1^H NMR (400 MHz, Chloroform-*d*) δ 7.81 (d, *J* = 8.2 Hz, 2H, *H*_Ar_), 7.68 (s, 1H, *H*_trz_), 7.41–7.31 (m, 7H, *H*_Ar_), 5.58 (s, 2H, ArC*H*_2_), 3.77 (s, 2H, C*H*_2_CN). ^13^C NMR (101 MHz, Chloroform-*d*) δ 147.4, 134.7, 130.6, 129.7, 129.3, 129.0, 128.5, 128.2, 126.5, 119.8, 117.8, 54.4, 23.5. HRMS (ESI) C_17_H_15_N_4_, *m*/*z*: calc. for [M+H]^+^: 275.1291; found: 275.1297.

### 3.4. Preparation and Characterization of Novel Compounds

#### 3.4.1. General Procedure **A** for Solvent-Free Preparation of 5-Amino-1-(2,2-Diethoxyethyl)-1,2,3-Triazoles **3** via Dipolar Azide−Nitrile Cycloaddition (DCR)

A 25 mL round-bottom flask equipped with a magnetic stir bar was charged with 0.5 mmol of acetonitrile derivative **1** and 1.1 equiv. of diethyl 2-azidoacetaldehyde diethyl acetal **2**. The resulting mixture was placed into a water bath (room temperature), and 0.1 equiv. of powdered potassium tert-butoxide was added portionwise. The reaction mixture was allowed to stir for 3 h at room temperature. After 3 h, the reaction mixture was dissolved in an EtOAc/H_2_O mixture (1:1, 10 mL); the organic phase was separated and the aqueous mixture was extracted with ethyl acetate (3 × 5 mL). The organic extract was evaporated to dryness in vacuo. Purification by chromatography (eluent—hexane: ethyl acetate 2:1) yielded an analytically pure product.

#### 3.4.2. General Procedure **B** for Preparation of 5-Amino-1-(2,2-Diethoxyethyl)-1,2,3-Triazoles **3** via Dipolar Azide−Nitrile Cycloaddition (DCR) in DMSO

A 25 mL round-bottom flask equipped with a magnetic stir bar was charged with 0.5 mmol of acetonitrile derivative **1**, 3.0 equiv. of diethyl 2-azidoacetaldehyde diethyl acetal **2** and DMSO (8 mL). The resulting mixture was placed into a water bath (room temperature), and 0.5 equiv. of powdered potassium tert-butoxide was added portionwise. The reaction mixture was allowed to stir for 3 h at 70 °C. On completion, the mixture was poured into water and extracted with dichloromethane (3 × 10 mL). The combined organic phases were washed with brine, dried over MgSO_4_, filtered and concentrated under reduced pressure. Purification by chromatography (eluent—hexane: ethyl acetate 2:1) yielded an analytically pure product.

#### 3.4.3. General Procedure **C** for Preparation of 1*H*-Imidazole Derivatives **4**

A 25 mL round-bottomed flask, equipped with a magnetic stir bar and reflux condenser, was charged with a mixture of 0.5 mmol of 5-amino-1-(2,2-diethoxyethyl)-1,2,3-triazole **3a**-**n**, corresponding ROH (10 mL) and 0.5 mL of conc. HCl. The resulting mixture was refluxed for 3 h. On completion, the mixture was neutralized by a water solution of NaOH and extracted with dichloromethane (3 × 10 mL). The combined organic phases were washed with brine, dried over MgSO_4_, filtered and concentrated under reduced pressure. Purification by chromatography (ethyl acetate) yielded an analytically pure product.

#### 3.4.4. General Procedure **D** for CuAAC Synthesis of **4y** and **4z**

To a solution of **3g** or **3i** (0.3 mmol) in anhydrous toluene (2 mL), the corresponding amount of benzyl azide (2 equiv.) and CuTC (copper (I) thiophene-2-carboxylate) (5 mol.%) was added. The reaction mixture was stirred at room temperature for 4 h at 50 °C. Upon the completion of the reaction (monitored by TLC), the mixed solvent was removed under reduced pressure and the residue was purified by column chromatography on silica gel (ethyl acetate), yielding an analytically pure product.

*1-(2,2-diethoxyethyl)-4-phenyl-1H-1,2,3-triazol-5-amine* (**3a**)

Compound **3a** was synthesized according to general procedure A (yield 92%) as a yellow oil. ^1^H NMR (400 MHz, Chloroform-*d*) δ 7.63 (d, *J* = 7.5 Hz, 2H, *H*_Ar_), 7.41 (t, *J* = 7.6 Hz, 2H, *H*_Ar_), 7.29–7.24 (m, 1H, *H*_Ar_), 4.76 (t, *J* = 5.3 Hz, 1H, C*H*(OEt)_2_), 4.39 (s, 2H, N*H*_2_), 4.33 (d, *J* = 5.3 Hz, 2H, C*H*_2_CH), 3.80–3.72 (m, 2H, OC*H_2_*CH_3_), 3.57–3.49 (m, 2H, OC*H_2_*CH_3_), 1.17 (t, *J* = 7.0 Hz, 6H, OCH_2_C*H*_3_). ^13^C NMR (101 MHz, Chloroform-*d*) δ 139.0, 132.0, 130.5, 129.0, 126.9, 125.8, 102.5, 64.5, 50.0, 15.3. HRMS (ESI) C_14_H_21_N_4_O_2_, *m*/*z*: calc. for [M+H]^+^: 277.1659; found: 277.1665.

*4-(2-bromophenyl)-1-(2,2-diethoxyethyl)-1H-1,2,3-triazol-5-amine* (**3b**)

Compound **3b** was synthesized according to general procedure A (yield 98%) as a light brown oil. ^1^H NMR (400 MHz, Chloroform-*d*) δ 7.61 (d, *J* = 8.0 Hz, 1H, *H*_Ar_), 7.50 (d, *J* = 7.6 Hz, 1H, *H*_Ar_), 7.35 (t, *J* = 7.5 Hz, 1H, *H*_Ar_), 7.20 (t, *J* = 7.7 Hz, 1H, *H*_Ar_), 4.79 (t, *J* = 5.4 Hz, 1H, C*H*(OEt)_2_), 4.33 (d, *J* = 5.4 Hz, 2H, C*H*_2_CH), 4.23 (s, 2H, N*H*_2_), 3.80–3.73 (m, 2H, OC*H*_2_CH_3_), 3.57–3.50 (m, 2H, OC*H*_2_CH_3_), 1.18 (t, *J* = 7.0 Hz, 6H, OCH_2_C*H*_3_). ^13^C NMR (101 MHz, Chloroform-*d*) δ 139.5, 133.0, 132.6, 132.5, 130.9, 129.7, 127.7, 122.7, 102.5, 64.5, 49.9, 15.3. HRMS (ESI) C_14_H_20_BrN_4_O_2_, *m*/*z*: calc. for [M+H]^+^: 355.0764; found: 355.0770.

*4-(2-chlorophenyl)-1-(2,2-diethoxyethyl)-1H-1,2,3-triazol-5-amine* (**3c**)

Compound **3c** was synthesized according to general procedure A (yield 98%) as a yellow oil. ^1^H NMR (400 MHz, Chloroform-*d*) δ 7.61–7.57 (m, 1H, *H*_Ar_), 7.43 (d, *J* = 7.6 Hz, 1H, *H*_Ar_), 7.37–7.28 (m, 2H, *H*_Ar_), 4.80 (t, *J* = 5.4 Hz, 1H, C*H*(OEt)_2_), 4.35 (d, *J* = 5.4 Hz, 2H, C*H*_2_CH), 4.28 (s, 2H, N*H*_2_), 3.81–3.74 (m, 2H, OC*H_2_*CH_3_), 3.58–3.52 (m, 2H, OC*H*_2_CH_3_), 1.19 (t, *J* = 7.0 Hz, 6H, OCH_2_C*H*_3_). ^13^C NMR (101 MHz, Chloroform-*d*) δ 139.9, 132.2, 132.2, 130.5, 129.8, 129.3, 129.3, 127.2, 102.6, 64.6, 49.9, 15.3. HRMS (ESI) C_14_H_20_ClN_4_O_2_, *m*/*z*: calc. for [M+H]^+^: 311.1269; found: 355. 311.1274.

*1-(2,2-diethoxyethyl)-4-(4′-methoxy-[1,1′-biphenyl]-2-yl)-1H-1,2,3-triazol-5-amine* (**3d**)

Compound **3d** was synthesized according to general procedure B (yield 78%) as a yellow oil. ^1^H NMR (400 MHz, Chloroform-*d*) δ 7.68–7.63 (m, 1H, *H*_Ar_), 7.40–7.34 (m, 3H, *H*_Ar_), 7.23 (d, *J* = 9.2 Hz, 2H, *H*_Ar_), 6.82 (d, *J* = 8.7 Hz, 2H, *H*_Ar_), 4.66 (t, *J* = 5.2 Hz, 1H, C*H*(OEt)_2_), 4.17 (d, *J* = 5.2 Hz, 2H, C*H*_2_CH), 3.77 (s, 3H, ArOC*H*_3_), 3.70–3.62 (m, 2H, OC*H_2_*CH_3_), 3.48–3.40 (m, 4H, N*H*_2_ and OC*H*_2_CH_3_), 1.09 (t, *J* = 7.0 Hz, 6H, OCH_2_C*H*_3_). ^13^C NMR (101 MHz, Chloroform-*d*) δ 158.9, 139.4, 139.0, 133.7, 131.5, 130.2, 130.1, 129.8, 128.3, 127.5, 114.1, 102.3, 64.0, 55.2, 50.1, 15.3. HRMS (ESI) C_21_H_27_N_4_O_3_, *m*/*z*: calc. for [M+H]^+^: 383.2078; found: 383.2077.

*4-(4-bromophenyl)-1-(2,2-diethoxyethyl)-1H-1,2,3-triazol-5-amine* (**3e**)

Compound **3e** was synthesized according to general procedure A (yield 76%) as a yellow solid. M.p. 101–102 °C. ^1^H NMR (400 MHz, Chloroform-*d*) δ 7.52 (s, 4H, *H*_Ar_), 4.76 (td, *J* = 5.2, 1.0 Hz, 1H, C*H*(OEt)_2_), 4.43 (s, 2H, N*H*_2_), 4.33 (d, *J* = 5.2 Hz, 2H, C*H_2_*CH), 3.81–3.73 (m, 2H, OCH_2_C*H*_3_), 3.58–3.50 (m, 2H, OCH_2_C*H*_3_), 1.19 (t, *J* = 7.0 Hz, 6H, OCH_2_C*H*_3_). ^13^C NMR (101 MHz, Chloroform-*d*) δ 139.1, 132.1, 130.9, 129.5, 127.2, 120.6, 102.4, 64.5, 50.1, 15.3. HRMS (ESI) C_14_H_20_BrN_4_O_2_, *m*/*z*: calc. for [M+H]^+^: 355.0764; found: 355.0770.

*1-(2,2-diethoxyethyl)-4-(4′-methyl-[1,1′-biphenyl]-4-yl)-1H-1,2,3-triazol-5-amine* (**3f**)

Compound **3f** was synthesized according to general procedure B (yield 83%) as a yellow solid. M.p. 140–142 °C. ^1^H NMR (400 MHz, Chloroform-*d*) δ 7.71 (d, *J* = 8.3 Hz, 2H, *H*_Ar_), 7.65 (d, *J* = 8.1 Hz, 2H, *H*_Ar_), 7.51 (d, *J* = 8.0 Hz, 2H, *H*_Ar_), 7.24 (d, *J* = 7.9 Hz, 2H, *H*_Ar_), 4.78 (t, *J* = 5.3 Hz, 1H, C*H*(OEt)_2_), 4.43 (s, 2H, N*H*_2_), 4.36 (d, *J* = 5.3 Hz, 2H, C*H*_2_CH), 3.82–3.74 (m, 2H, OC*H*_2_CH_3_), 3.59–3.51 (m, 2H, OC*H*_2_CH_3_), 2.38 (s, 3H, Ar*CH*_3_), 1.20 (t, *J* = 7.0 Hz, 6H, OCH_2_C*H*_3_). ^13^C NMR (101 MHz, Chloroform-*d*) δ 139.6, 139.1, 137.9, 137.2, 130.7, 129.7, 128.9, 127.5, 126.9, 126.1, 105.2, 64.6, 50.1, 21.2, 15.4. HRMS (ESI) C_21_H_27_N_4_O_2_, *m*/*z*: calc. for [M+H]^+^: 367.2129; found: 367.2135.

*1-(2,2-diethoxyethyl)-4-(4-ethynylphenyl)-1H-1,2,3-triazol-5-amine* (**3g**)

Compound **3g** was synthesized according to general procedure A (yield 63%) as a yellow oil. ^1^H NMR (400 MHz, Chloroform-*d*) δ 7.62 (d, *J* = 8.1 Hz, 2H, *H*_Ar_), 7.51 (d, *J* = 8.0 Hz, 2H, *H*_Ar_), 4.75 (t, *J* = 5.2 Hz, 1H, C*H*(OEt)_2_), 4.47 (s, 2H, N*H*_2_), 4.32 (d, *J* = 5.1 Hz, 2H, C*H_2_*CH), 3.80–3.72 (m, 2H, OC*H_2_*CH_3_), 3.57–3.49 (m, 2H, OC*H*_2_CH_3_), 3.09 (s, 1H, Ar-CC*H*), 1.18 (t, *J* = 7.0 Hz, 6H, OCH_2_C*H*_3_). ^13^C NMR (101 MHz, Chloroform-*d*) δ 139.4, 132.7, 132.5, 129.6, 125.2, 120.3, 102.3, 83.7, 77.6, 64.5, 50.0, 15.3. HRMS (ESI) C_16_H_21_N_4_O_2_, *m*/*z*: calc. for [M+H]^+^: 301.1666; found: 301.1664.

*1-(2,2-diethoxyethyl)-4-(2-ethynylphenyl)-1H-1,2,3-triazol-5-amine* (**3h**)

Compound **3h** was synthesized according to general procedure A (yield 53%) as a light brown oil. ^1^H NMR (400 MHz, Chloroform-*d*) δ 7.70 (d, *J* = 7.9 Hz, 1H, *H*_Ar_), 7.59 (d, *J* = 7.6 Hz, 1H, *H*_Ar_), 7.45 (t, *J* = 7.6 Hz, 1H, *H*_Ar_), 7.31 (t, *J* = 7.6 Hz, 1H, *H*_Ar_), 4.81 (t, *J* = 5.4 Hz, 1H, C*H*(OEt)_2_), 4.42 (s, 2H, N*H*_2_), 4.37 (d, *J* = 5.4 Hz, 2H, C*H_2_*CH), 3.78 (dd, *J* = 9.2, 7.1 Hz, 2H, OC*H*_2_CH_3_), 3.56 (dd, *J* = 9.2, 7.1 Hz, 2H, OC*H*_2_CH_3_), 3.22 (s, 1H, Ar-CC*H*), 1.20 (t, *J* = 7.0 Hz, 6H, OCH_2_C*H_3_*). ^13^C NMR (101 MHz, Chloroform-*d*) δ 139.8, 134.6, 133.7, 130.6, 130.3, 129.5, 127.5, 119.3, 102.5, 83.3, 81.0, 64.4, 50.0, 15.4. HRMS (ESI) C_16_H_21_N_4_O_2_, *m*/*z*: calc. for [M+H]^+^: 301.1666; found: 301.1659.

*1-(2,2-diethoxyethyl)-4-(2-(p-tolylethynyl)phenyl)-1H-1,2,3-triazol-5-amine* (**3i**)

Compound **3i** was synthesized according to general procedure A (yield 93%) as a light brown oil. ^1^H NMR (400 MHz, Chloroform-*d*) δ 7.75 (d, *J* = 7.8 Hz, 1H, *H*_Ar_), 7.61 (d, *J* = 7.7 Hz, 1H, *H*_Ar_), 7.42 (t, *J* = 7.6 Hz, 1H, *H*_Ar_), 7.37 (d, *J* = 8.0 Hz, 2H, *H*_Ar_), 7.33 (t, *J* = 7.7 Hz, 1H, *H*_Ar_), 7.12 (d, *J* = 7.8 Hz, 2H, *H*_Ar_), 4.82 (t, *J* = 5.2 Hz, 1H, 1H, C*H*(OEt)_2_), 4.56 (s, 2H, N*H*_2_), 4.39 (d, *J* = 5.3 Hz, 2H, C*H*_2_CH), 3.75 (dd, *J* = 9.0, 7.2 Hz, 2H, OC*H*_2_CH_3_), 3.53 (dd, *J* = 9.0, 7.1 Hz, 2H, OC*H*_2_CH_3_), 2.35 (s, 3H, Ar*CH*_3_), 1.13 (t, *J* = 7.0 Hz, 6H, OCH_2_C*H*_3_). ^13^C NMR (101 MHz, Chloroform-*d*) δ 139.9, 138.8, 133.9, 132.9, 131.5, 130.9, 130.2, 129.2, 128.7, 127.4, 120.6, 119.9, 102.4, 93.5, 88.2, 64.4, 50.0, 21.6, 15.2. HRMS (ESI) C_23_H_27_N_4_O_2_, *m*/*z*: calc. for [M+H]^+^: 391.2129; found: 391.2132.

*4,4′-(1,4-phenylene)bis(1-(2,2-diethoxyethyl)-1H-1,2,3-triazol-5-amine)* (**3j**)

Compound **3j** was synthesized according to general procedure B (yield 51%) as a pale yellow solid. M.p. 177–179 °C. ^1^H NMR (400 MHz, Chloroform-*d*) δ 7.70 (s, 4H, *H*_Ar_), 4.77 (t, *J* = 5.3 Hz, 2H, C*H*(OEt)_2_), 4.47 (s, 4H, N*H*_2_), 4.34 (d, *J* = 5.2 Hz, 4H, C*H*_2_CH), 3.81–3.74 (m, 4H, OC*H_2_*CH_3_), 3.58–3.51 (m, 4H, OC*H*_2_CH_3_), 1.19 (t, *J* = 7.0 Hz, 12H, OCH_2_C*H*_3_). ^13^C NMR (101 MHz, Chloroform-*d*) δ 139.1, 130.4, 130.2, 126.1, 102.4, 64.5, 50.0, 15.3. HRMS (ESI) C_22_H_35_N_8_O_4_, *m*/*z*: calc. for [M+H]^+^: 475.2776; found: 475.2780.

*1-(2,2-diethoxyethyl)-4-(pyridin-2-yl)-1H-1,2,3-triazol-5-amine* (**3k**)

Compound **3k** was synthesized according to general procedure A (yield 87%) as a light brown oil. ^1^H NMR (400 MHz, Chloroform-*d*) δ 8.48 (d, *J* = 4.9 Hz, 1H, *H*_Ar_), 8.08 (d, *J* = 8.1 Hz, 1H, *H*_Ar_), 7.70 (td, *J* = 8.0, 1.7 Hz, 1H, *H*_Ar_), 7.09–7.04 (m, 1H, *H*_Ar_), 5.86 (s, 2H, N*H_2_*), 4.78 (t, *J* = 5.3 Hz, 1H, C*H*(OEt)_2_), 4.34 (d, *J* = 5.3 Hz, 2H, C*H*_2_CH), 3.82–3.75 (m, 2H, OC*H_2_*CH_3_), 3.59–3.53 (m, 2H, OC*H_2_*CH_3_), 1.21 (t, *J* = 7.0 Hz, 6H, OCH_2_C*H*_3_). ^13^C NMR (101 MHz, Chloroform-*d*) δ 153.0, 148.3, 142.8, 136.4, 127.8, 120.2, 118.3, 102.2, 64.2, 49.7, 15.2. HRMS (ESI) C_13_H_20_N_5_O_2_, *m*/*z*: calc. for [M+H]^+^: 278.1612; found: 278.1617.

*1-(2,2-diethoxyethyl)-4-(4-(7-(4-methoxyphenyl)benzo[c][1,2,5]thiadiazol-4-yl)phenyl)-1H-1,2,3-triazol-5-amine* (**3l**)

Compound **3l** was synthesized according to general procedure B (yield 46%) as a red solid. M.p. 156–158 °C. ^1^H NMR (400 MHz, Chloroform-*d*) δ 8.04 (d, *J* = 8.3 Hz, 2H, *H*_Ar_), 7.92 (d, *J* = 8.7 Hz, 2H, *H*_Ar_), 7.84 (d, *J* = 8.2 Hz, 2H, *H*_Ar_), 7.78 (d, *J* = 7.3 Hz, 1H, *H*_BTD_), 7.72 (d, *J* = 7.3 Hz, 1H, *H*_BTD_), 7.07 (d, *J* = 8.6 Hz, 2H, *H*_Ar_), 4.81 (t, *J* = 5.3 Hz, 1H, C*H*(OEt)_2_), 4.52 (s, 2H, N*H*_2_), 4.39 (d, *J* = 5.3 Hz, 2H, C*H*_2_CH), 3.89 (s, 3H, ArOC*H*_3_), 3.84–3.77 (m, 2H, OC*H*_2_CH_3_), 3.61–3.54 (m, 2H, OC*H*_2_CH_3_), 1.22 (t, *J* = 7.0 Hz, 6H, *OCH_2_CH*_3_). ^13^C NMR (101 MHz, Chloroform-*d*) δ 160.0, 154.3, 154.2, 139.4, 135.9, 133.0, 132.2, 131.9, 130.5, 130.1, 129.9, 129.8, 128.1, 127.4, 125.8, 114.2, 102.6, 64.6, 55.5, 50.1, 15.4. HRMS (ESI) C_27_H_29_N_6_O_3_S, *m*/*z*: calc. for [M+H]^+^: 517.2016; found: 517.2018.

*4-(2-(1-benzyl-1H-1,2,3-triazol-4-yl)phenyl)-1-(2,2-diethoxyethyl)-1H-1,2,3-triazol-5-amine* (**3m**)

Compound **3m** was synthesized according to general procedure B (yield 73%) as a light brown oil. ^1^H NMR (400 MHz, Chloroform-*d*) δ 7.94 (d, *J* = 7.5 Hz, 1H, *H*_Ar_), 7.50–7.42 (m, 3H, *H*_Ar_), 7.35–7.31 (m, 3H, *H*_Ar_), 7.22–7.18 (m, 3H, *H*_Ar_), 5.45 (s, 2H, C*H_2_*Ar), 4.68 (t, *J* = 5.2 Hz, 1H, C*H*(OEt)_2_), 4.12 (d, *J* = 5.2 Hz, 2H, C*H*_2_CH), 3.81 (s, 2H, N*H*_2_), 3.74–3.68 (m, 2H, OC*H*_2_CH_3_), 3.51–3.45 (m, 2H, OC*H*_2_CH_3_), 1.13 (t, *J* = 7.0 Hz, 6H, OCH_2_C*H*_3_). ^13^C NMR (101 MHz, Chloroform-*d*) δ 146.7, 139.4, 134.8, 131.2, 130.4, 130.3, 129.6, 129.2, 129.1, 128.7, 128.7, 128.7, 128.0, 122.8, 102.3, 64.4, 54.1, 49.8, 15.3. HRMS (ESI) C_23_H_28_N_7_O_2_, *m*/*z*: calc. for [M+H]^+^: 434.2299; found: 434.2297.

*4-(4-(1-benzyl-1H-1,2,3-triazol-4-yl)phenyl)-1-(2,2-diethoxyethyl)-1H-1,2,3-triazol-5-amine* (**3n**)

Compound **3n** was synthesized according to general procedure B (yield 84%) as a light brown oil. ^1^H NMR (400 MHz, Chloroform-*d*) δ 7.85 (d, *J* = 8.2 Hz, 2H, *H*_Ar_), 7.70 (d, *J* = 8.1 Hz, 3H, *H*_Ar_), 7.40–7.36 (m, 3H, *H*_Ar_), 7.33–7.30 (m, 2H, *H*_Ar_), 5.57 (s, 2H, C*H_2_*Ar), 4.78 (t, *J* = 5.2 Hz, 1H, C*H*(OEt)_2_), 4.46 (s, 2H, N*H_2_*), 4.36 (d, *J* = 5.2 Hz, 2H, C*H_2_*CH), 3.79 (dd, *J* = 9.2, 7.0 Hz, 2H, OC*H*_2_CH_3_), 3.56 (dd, *J* = 9.2, 7.1 Hz, 2H, OC*H_2_*CH_3_), 1.21 (t, *J* = 7.0 Hz, 6H, OCH_2_C*H_3_*). ^13^C NMR (101 MHz, Chloroform-*d*) δ 148.0, 139.2, 134.8, 131.8, 130.1, 129.3, 129.1, 128.9, 128.2, 126.3, 126.0, 119.6, 102.5, 64.6, 54.4, 50.1, 15.4. HRMS (ESI) C_23_H_28_N_7_O_2_, *m*/*z*: calc. for [M+H]^+^: 434.2299; found: 434.2304.

*(1H-imidazol-2-yl)(phenyl)methanol* (**4a**) [6]

Compound **4a** was synthesized from **3a** according to general procedure C via boiling in conc. HCl (white solid yield 89%). M.p. 196–198 °C (lit.: 192–194 °C [42]). ^1^H NMR (400 MHz, DMSO-*d*_6_) δ 11.99 (s, 1H, N*H*), 7.39 (d, *J* = 7.6 Hz, 2H, *H*_Ar_), 7.30 (t, *J* = 7.5 Hz, 2H, *H*_Ar_), 7.21 (t, *J* = 7.2 Hz, 1H, *H*_Ar_), 6.97 (s, 1H, -*H*C=C*H-*), 6.75 (s, 1H, -*H*C=C*H-*), 6.22 (d, *J* = 4.5 Hz, 1H, O*H*), 5.71 (d, *J* = 4.4 Hz, 1H, C*H*OH). ^13^C NMR (101 MHz, DMSO-*d*_6_) δ 150.2, 143.4, 128.0, 127.0, 126.4, 115.9, 69.6. HRMS (ESI) C_10_H_11_N_2_O, *m*/*z*: calc. for [M+H]^+^: 175.0866; found: 157.0869.

*2-(methoxy(phenyl)methyl)-1H-imidazole* (**4b**)

Compound **4b** was synthesized according to general procedure C (yield 75%) from **3a** and MeOH as a pale yellow solid. M.p. 99–100 °C. ^1^H NMR (400 MHz, DMSO-*d*_6_) δ 12.08 (s, 1H, N*H*), 7.40–7.32 (m, 4H, *H*_Ar_), 7.30–7.25 (m, 1H, *H*_Ar_), 6.93 (s, 2H, -*H*C=C*H-*), 5.38 (s, 1H, C*H*OR), 3.26 (s, 3H, OC*H_3_*). ^1^H NMR (400 MHz, Chloroform-*d*) δ 7.37–7.29 (m, 5H, *H*_Ar_), 6.98 (s, 2H, -*H*C=C*H-*), 5.44 (s, 1H, C*H*OR), 3.40 (s, 3H, OC*H_3_*). ^13^C NMR (101 MHz, DMSO-*d*_6_) δ 147.2, 140.0, 128.2, 127.7, 126.9, 109.6, 79.3, 56.5. HRMS (ESI) C_11_H_13_N_2_O, *m*/*z*: calc. for [M+H]^+^: 189.1022; found: 189.1022.

*2-(ethoxy(phenyl)methyl)-1H-imidazole* (**4c**)

Compound **4c** was synthesized according to general procedure C (yield 57%) from **3a** and EtOH as a white solid. M.p. 127–129 °C. ^1^H NMR (400 MHz, DMSO-*d*_6_) δ 12.00 (s, 1H, N*H*), 7.38 (t, *J* = 8.8 Hz, 2H, *H*_Ar_), 7.33 (d, *J* = 7.6 Hz, 2H, *H*_Ar_), 7.29–7.24 (m, 1H, *H*_Ar_), 6.92 (s, 2H, -*H*C=C*H-*), 5.48 (s, 1H, C*H*OR), 3.46–3.38 (m, 2H), 1.16 (t, *J* = 7.0 Hz, 3H, C*H_3_*). ^1^H NMR (400 MHz, Chloroform-*d*) δ 7.38–7.33 (m, 4H, *H*_Ar_), 7.32–7.29 (m, 1H, *H*_Ar_), 6.98 (s, 2H, -*H*C=C*H-*), 5.54 (s, 1H, C*H*OR), 3.60–3.52 (m, 2H, OC*H_2_*CH_3_), 1.25 (t, *J* = 7.0 Hz, 3H, OCH_2_C*H_3_*). ^13^C NMR (101 MHz, DMSO-*d*_6_) δ 147.6, 140.4, 128.2, 127.5, 126.8, 77.4, 64.0, 15.1. HRMS (ESI) C_12_H_15_N_2_O, *m*/*z*: calc. for [M+H]^+^: 203.1179; found: 203.1166.

*2-(isopropoxy(phenyl)methyl)-1H-imidazole* (**4d**)

Compound **4d** was synthesized according to general procedure C (yield 48%) from **3a** and *i*-PrOH as a white solid. M.p. 83–85 °C. ^1^H NMR (400 MHz, DMSO-*d*_6_) δ 11.94 (s, 1H, N*H*), 7.38 (d, *J* = 7.2 Hz, 2H, *H*_Ar_), 7.32 (t, *J* = 7.4 Hz, 2H, *H*_Ar_), 7.25 (t, *J* = 7.1 Hz, 1H, *H*_Ar_), 6.91 (s, 2H, -*H*C=C*H-*), 5.58 (s, 1H, C*H*OR), 3.55 (p, *J* = 6.1 Hz, 1H, OC*H*(CH_3_)_2_), 1.14 (d, *J* = 6.0 Hz, 3H, OCH(C*H_3_*)_2_), 1.11 (d, *J* = 6.1 Hz, 3H, OCH(C*H_3_*)_2_). ^1^H NMR (400 MHz, Chloroform-*d*) δ 7.34 (d, *J* = 7.3 Hz, 2H, *H*_Ar_), 7.30 (d, *J* = 7.0 Hz, 2H, *H*_Ar_), 7.26–7.23 (m, 1H, *H*_Ar_), 6.93 (s, 2H, -*H*C=C*H-*), 5.66 (s, 1H, C*H*OR), 3.73–3.66 (m, 1H, OC*H*(CH_3_)_2_), 1.18 (d, *J* = 6.2 Hz, 3H, OCH(C*H_3_*)_2_), 1.16 (d, *J* = 6.2 Hz, 3H, OCH(C*H_3_*)_2_). ^13^C NMR (101 MHz, DMSO-*d*_6_) δ 148.0, 140.9, 128.1, 127.4, 126.8, 74.8, 69.1, 22.1. HRMS (ESI) C_13_H_17_N_2_O, *m*/*z*: calc. for [M+H]^+^: 217.1335; found: 217.1333.

*2-(butoxy(phenyl)methyl)-1H-imidazole* (**4e**)

Compound **4e** was synthesized according to general procedure C (yield 51%) from **3a** and *n*-BuOH as a light yellow solid. M.p. 93–95 °C. ^1^H NMR (400 MHz, DMSO-*d*_6_) δ 11.95 (s, 1H, N*H*), 7.40–7.31 (m, 4H, *H*_Ar_), 7.28–7.24 (m, 1H, *H*_Ar_), 6.92 (s, 2H, -*H*C=C*H-*), 5.45 (s, 1H, C*H*OR), 3.39–3.33 (m, 2H, O(C*H_2_*)_3_CH_3_), 1.57–1.48 (m, 2H, O(C*H_2_*)_3_CH_3_), 1.38–1.30 (m, 2H, O(C*H_2_*)_3_CH_3_), 0.85 (t, *J* = 7.1 Hz, 3H, O(CH_2_)_3_C*H_3_*). ^1^H NMR (400 MHz, Chloroform-*d*) δ 7.36 (t, *J* = 8.2 Hz, 4H, *H*_Ar_), 7.29 (d, *J* = 7.2 Hz, 1H, *H*_Ar_), 6.98 (s, 2H, -*H*C=C*H-*), 5.54 (s, 1H, C*H*OR), 3.54–3.46 (m, 2H, O(C*H_2_*)_3_CH_3_), 1.65–1.58 (m, 2H, O(C*H_2_*)_3_CH_3_), 1.43–1.36 (m, 2H, O(C*H_2_*)_3_CH_3_), 0.90 (t, *J* = 7.4 Hz, 3H, O(CH_2_)_3_C*H_3_*). ^13^C NMR (101 MHz, DMSO-*d*_6_) δ 147.5, 140.4, 128.2, 127.5, 126.8, 77.6, 68.2, 31.3, 18.9, 13.8. HRMS (ESI) C_14_H_19_N_2_O, *m*/*z*: calc. for [M+H]^+^: 231.1492; found: 231.1493.

*2-(tert-butoxy(phenyl)methyl)-1H-imidazole* (**4f**)

Compound **4f** was synthesized according to general procedure C (yield 35%) from **3a** and *t*-BuOH as a white solid. M.p. 129–131 °C. ^1^H NMR (400 MHz, Chloroform-*d*) δ 7.39 (d, *J* = 7.6 Hz, 2H, *H*_Ar_), 7.30 (t, *J* = 7.4 Hz, 2H, *H*_Ar_), 7.22 (t, *J* = 7.3 Hz, 1H, *H*_Ar_), 6.96 (s, 2H, -*H*C=C*H-*), 5.82 (s, 1H, C*H*OR), 1.23 (s, 9H, OC(C*H_3_*)_3_). ^1^H NMR (400 MHz, DMSO-*d*_6_) δ 11.83 (s, 1H, N*H*), 7.35 (d, *J* = 7.7 Hz, 2H, *H*_Ar_), 7.29 (t, *J* = 7.4 Hz, 2H, *H*_Ar_), 7.21 (t, *J* = 7.1 Hz, 1H, *H*_Ar_), 6.88 (s, 2H, -*H*C=C*H-*), 5.72 (s, 1H, C*H*OR), 1.15 (s, 9H, OC(C*H_3_*)_3_). ^13^C NMR (101 MHz, Chloroform-*d*) δ 150.5, 142.0, 128.7, 128.5, 127.6, 126.8, 126.4, 76.0, 70.7, 28.5. HRMS (ESI) C_14_H_19_N_2_O, *m*/*z*: calc. for [M+H]^+^: 231.1492; found: 231.1495.

*2-((2-bromophenyl)(methoxy)methyl)-1H-imidazole* (**4g**)

Compound **4g** was synthesized according to general procedure C (yield 62%) from **3b** and MeOH as a white solid. M.p. 159–160 °C. ^1^H NMR (400 MHz, Chloroform-*d*) δ 7.56 (d, *J* = 8.0 Hz, 1H, *H*_Ar_), 7.41 (d, *J* = 7.7 Hz, 1H, *H*_Ar_), 7.30 (t, *J* = 7.5 Hz, 1H, *H*_Ar_), 7.16 (t, *J* = 7.6 Hz, 1H, *H*_Ar_), 6.98 (s, 2H, -*H*C=C*H-*), 5.81 (s, 1H, C*H*OR), 3.41 (s, 3H, OC*H_3_*). ^1^H NMR (400 MHz, DMSO-*d*_6_) δ 12.15 (s, 1H, N*H*), 7.60 (d, *J* = 8.0 Hz, 1H, *H*_Ar_), 7.54 (d, *J* = 7.7 Hz, 1H, *H*_Ar_), 7.42 (t, *J* = 7.5 Hz, 1H, *H*_Ar_), 7.26 (t, *J* = 7.7 Hz, 1H, *H*_Ar_), 6.95 (s, 2H, -*H*C=C*H-*), 5.64 (s, 1H, C*H*OR), 3.28 (s, 3H, OC*H_3_*). ^13^C NMR (101 MHz, Chloroform-*d*) δ 146.7, 138.2, 133.2, 129.9, 128.8, 128.0, 123.9, 122.3, 78.6, 57.5. HRMS (ESI) C_11_H_12_BrN_2_O, *m*/*z*: calc. for [M+H]^+^: 267.0128; found: 267.0132.

*2-((2-bromophenyl)(ethoxy)methyl)-1H-imidazole* (**4h**)

Compound **4h** was synthesized according to general procedure C (yield 70%) from **3b** and EtOH as a light brown solid. M.p. 147–149 °C. ^1^H NMR (400 MHz, Chloroform-*d*) δ 7.57 (d, *J* = 8.0 Hz, 1H, *H*_Ar_), 7.41 (d, *J* = 7.8 Hz, 1H, *H*_Ar_), 7.32 (t, *J* = 7.5 Hz, 1H, *H*_Ar_), 7.17 (t, *J* = 7.5 Hz, 1H, *H*_Ar_), 7.01 (s, 2H, -*H*C=C*H-*), 5.94 (s, 1H, C*H*OR), 3.60 (q, *J* = 7.0 Hz, 2H, OC*H_2_*CH_3_), 1.27 (t, *J* = 7.0 Hz, 3H, OCH_2_C*H_3_*). ^1^H NMR (400 MHz, DMSO-*d*_6_) δ 12.11 (s, 1H, N*H*), 7.58 (t, *J* = 7.6 Hz, 2H, *H*_Ar_), 7.42 (t, *J* = 7.5 Hz, 1H, *H*_Ar_), 7.25 (t, *J* = 7.6 Hz, 1H, *H*_Ar_), 7.07 (s, 1H, -*H*C=C*H-*), 6.80 (s, 1H, =C*H*), 5.73 (s, 1H, C*H*OR), 3.45 (q, *J* = 7.0 Hz, 2H, OC*H_2_*CH_3_), 1.15 (t, *J* = 7.0 Hz, 3H, OCH*_2_*C*H_3_*). ^13^C NMR (101 MHz, Chloroform-*d*) δ 147.0, 138.7, 133.1, 129.7, 128.9, 127.9, 123.8, 122.2, 76.8, 65.2, 15.3. HRMS (ESI) C_12_H_14_BrN_2_O, *m*/*z*: calc. for [M+H]^+^: 281.0284; found: 281.0290.

*2-((2-chlorophenyl)(ethoxy)methyl)-1H-imidazole* (**4i**)

Compound **4i** was synthesized according to general procedure C (yield 50%) from **3c** and EtOH as a white solid. M.p. 148–149 °C. ^1^H NMR (400 MHz, Chloroform-*d*) δ 9.42 (s, 1H, N*H*), 7.40 (dd, *J* = 7.3, 1.9 Hz, 1H, *H*_Ar_), 7.36 (dd, *J* = 7.5, 1.6 Hz, 1H, *H*_Ar_), 7.26–7.22 (m, 2H, *H*_Ar_), 7.07–6.92 (m, 2H, -*H*C=C*H-*), 5.95 (s, 1H, C*H*OR), 3.58 (q, *J* = 7.0 Hz, 2H, OC*H_2_*CH_3_), 1.24 (t, *J* = 7.0 Hz, 3H, OCH*_2_*C*H_3_*). ^1^H NMR (400 MHz, DMSO-*d*_6_) δ 12.11 (s, 1H, N*H*), 7.60 (d, *J* = 7.1 Hz, 1H, *H*_Ar_), 7.44–7.36 (m, 2H, *H*_Ar_), 7.35–7.30 (m, 1H, *H*_Ar_), 6.93 (s, 2H, -*H*C=C*H-*), 5.78 (s, 1H, C*H*OR), 3.45 (q, *J* = 7.0 Hz, 2H, OC*H_2_*CH_3_), 1.15 (t, *J* = 7.0 Hz, 3H, OCH*_2_*C*H_3_*). ^13^C NMR (101 MHz, Chloroform-*d*) δ 147.1, 137.1, 133.6, 129.7, 129.4, 128.7, 127.2, 122.2, 74.6, 65.2, 15.3. HRMS (ESI) C_12_H_14_ClN_2_O, *m*/*z*: calc. for [M+H]^+^: 237.0789; found: 281.0795.

*2-(ethoxy(4′-methoxy-[1,1′-biphenyl]-2-yl)methyl)-1H-imidazole* (**4j**)

Compound **4j** was synthesized according to general procedure C (yield 70%) from **3d** and EtOH as a pale yellow solid. M.p. 158–159 °C. ^1^H NMR (400 MHz, Chloroform-*d*) δ 7.38–7.35 (m, 1H, *H*_Ar_), 7.31–7.23 (m, 5H, *H*_Ar_), 6.92 (s, 2H, -*H*C=C*H-*), 6.89 (d, *J* = 8.4 Hz, 2H, *H*_Ar_), 5.59 (s, 1H, C*H*OR), 3.80 (s, 3H, ArOC*H_3_*), 3.25 (p, *J* = 7.3 Hz, 2H, OC*H_2_*CH), 1.04 (t, *J* = 7.0 Hz, 3H, OCH*_2_*C*H_3_*). ^1^H NMR (400 MHz, DMSO-*d*_6_) δ 11.85 (s, 1H, N*H*), 7.56 (d, *J* = 7.1 Hz, 1H, *H*_Ar_), 7.33 (d, *J* = 8.7 Hz, 4H, *H*_Ar_), 7.22–7.18 (m, 1H, *H*_Ar_), 7.00 (d, *J* = 8.6 Hz, 2H, *H*_Ar_), 6.92 (s, 2H, -*H*C=C*H-*), 5.48 (s, 1H, C*H*OR), 3.82 (s, 3H, ArOC*H_3_*), 3.25–3.19 (m, 2H, OC*H_2_*CH_3_), 1.02 (t, *J* = 7.0 Hz, 3H, OCH*_2_*C*H_3_*). ^13^C NMR (101 MHz, Chloroform-*d*) δ 158.9, 148.8, 142.2, 137.2, 133.0, 130.7, 130.4, 128.0, 127.7, 127.7, 113.6, 74.8, 64.4, 55.4, 15.3. HRMS (ESI) C_19_H_21_N_2_O_2_, *m*/*z*: calc. for [M+H]^+^: 309.1598; found: 309.1604.

*2-(isopropoxy(4′-methoxy-[1,1′-biphenyl]-2-yl)methyl)-1H-imidazole* (**4k**)

Compound **4k** was synthesized according to general procedure C (yield 77%) from **3d** and *i*-PrOH as a white solid. M.p. 167–169 °C. ^1^H NMR (400 MHz, Chloroform-*d*) δ 7.43–7.39 (m, 1H, *H*_Ar_), 7.37 (d, *J* = 8.6 Hz, 2H, *H*_Ar_), 7.33–7.29 (m, 2H, *H*_Ar_), 7.26–7.23 (m, 1H, *H*_Ar_), 6.96 (s, 2H, -*H*C=C*H-*), 6.92 (d, *J* = 8.6 Hz, 2H, *H*_Ar_), 5.76 (s, 1H, C*H*OR), 3.83 (s, 3H, ArOC*H_3_*), 3.42 (h, *J* = 6.0 Hz, 1H, OC*H*(CH_3_)_2_), 0.97 (d, *J* = 6.2 Hz, 3H, OCH(C*H_3_*)_2_), 0.91 (d, *J* = 6.0 Hz, 3H, OCH(C*H_3_*)_2_). ^1^H NMR (400 MHz, DMSO-*d*_6_) δ 11.96 (s, 1H, N*H*), 7.60 (d, *J* = 7.6 Hz, 1H, *H*_Ar_), 7.38 (d, *J* = 8.4 Hz, 2H, *H*_Ar_), 7.36–7.28 (m, 2H, *H*_Ar_), 7.19 (d, *J* = 7.4 Hz, 1H, *H*_Ar_), 7.05 (s, 1H, -*H*C=C*H-*), 7.01 (d, *J* = 8.3 Hz, 2H, *H*_Ar_), 6.81 (s, 1H, -*H*C=C*H-*), 5.62 (s, 1H, C*H*OR), 3.80 (s, 3H, ArOC*H_3_*), 3.32–3.25 (m, 1H, OC*H*(CH_3_)_2_), 0.91–0.87 (m, 6H, OCH(C*H_3_*)_2_). ^13^C NMR (101 MHz, Chloroform-*d*) δ 158.9, 149.4, 142.0, 137.7, 133.0, 130.9, 130.3, 127.9, 127.7, 113.6, 71.9, 69.3, 55.4, 22.9, 21.5. HRMS (ESI) C_20_H_23_N_2_O_2_, *m*/*z*: calc. for [M+H]^+^: 323.1754; found: 323.1761.

*2-((4-bromophenyl)(ethoxy)methyl)-1H-imidazole* (**4l**)

Compound **4l** was synthesized according to general procedure C (yield 86%) from **3e** and EtOH as a pale yellow solid. M.p. 153–154 °C. ^1^H NMR (400 MHz, Chloroform-*d*) δ 9.34 (s, 1H, N*H*), 7.47 (d, *J* = 8.4 Hz, 2H, *H*_Ar_), 7.26–7.23 (d, *J* = 8.3 Hz, 2H, *H*_Ar_), 6.99 (s, 2H, -*H*C=C*H-*), 5.49 (s, 1H, C*H*OR), 3.59–3.52 (m, 2H, OC*H_2_*CH_3_), 1.25 (t, *J* = 7.0 Hz, 3H, OCH*_2_*C*H_3_*). ^1^H NMR (400 MHz, DMSO-*d*_6_) δ 12.04 (s, 1H, N*H*), 7.54 (d, *J* = 8.3 Hz, 2H, *H*_Ar_), 7.33 (d, *J* = 8.2 Hz, 2H, *H*_Ar_), 7.05 (s, 1H, -*H*C=C*H-*), 6.80 (s, 1H, -*H*C=C*H-*), 5.48 (s, 1H, C*H*OR), 3.43 (q, *J* = 7.0 Hz, 2H, OC*H_2_*CH_3_), 1.16 (t, *J* = 7.0 Hz, 3H, OCH*_2_*C*H_3_*). ^13^C NMR (101 MHz, Chloroform-*d*) δ 148.0, 138.7, 131.8, 128.6, 122.1, 77.4, 65.2, 15.3. HRMS (ESI) C_12_H_14_BrN_2_O, *m*/*z*: calc. for [M+H]^+^: 281.0284; found: 281.0290.

*2-(methoxy(4′-methyl-[1,1′-biphenyl]-4-yl)methyl)-1H-imidazole* (**4m**)

Compound **4m** was synthesized according to general procedure C (yield 45%) from **3f** and MeOH as a light brown solid. M.p. 147–149 °C. ^1^H NMR (400 MHz, DMSO-*d*_6_) δ 12.10 (s, 1H, N*H*), 7.61 (d, *J* = 8.0 Hz, 2H, *H*_Ar_), 7.54 (d, *J* = 7.9 Hz, 2H, *H*_Ar_), 7.44 (d, *J* = 8.1 Hz, 2H, *H*_Ar_), 7.26 (d, *J* = 7.9 Hz, 2H, *H*_Ar_), 6.94 (s, 2H, -*H*C=C*H-*), 5.42 (s, 1H, C*H*OR), 3.29 (s, 3H, ArC*H_3_*), 2.33 (s, 3H, OC*H_3_*). ^13^C NMR (101 MHz, DMSO-*d*_6_) δ 147.2, 139.4, 138.8, 136.9, 136.7, 129.5, 128.9, 127.4, 126.4, 126.2, 125.0, 78.9, 56.5, 20.6. HRMS (ESI) C_18_H_19_N_2_O, *m*/*z*: calc. for [M+H]^+^: 279.1492; found: 279.1496.

*2-(ethoxy(4′-methyl-[1,1′-biphenyl]-4-yl)methyl)-1H-imidazole* (**4n**)

Compound **4n** was synthesized according to general procedure C (yield 71%) from **3f** and EtOH as a light brown solid. M.p. 136–138 °C. ^1^H NMR (400 MHz, Chloroform-*d*) δ 9.31 (s, 1H, N*H*), 7.56 (d, *J* = 8.1 Hz, 2H, *H*_Ar_), 7.47–7.42 (m, 4H, *H*_Ar_), 7.24 (d, *J* = 8.0 Hz, 2H, *H*_Ar_), 7.02 (s, 2H, -*H*C=C*H-*), 5.59 (s, 1H, C*H*OR), 3.61 (dq, *J* = 14.3, 7.3 Hz, 2H, OC*H_2_*CH_3_), 2.39 (s, 3H, ArC*H_3_*), 1.28 (t, *J* = 7.0 Hz, 3H, OCH*_2_*C*H_3_*). ^1^H NMR (400 MHz, DMSO-*d*_6_) δ 12.03 (s, 1H, N*H*), 7.60 (d, *J* = 8.1 Hz, 2H, *H*_Ar_), 7.54 (d, *J* = 7.9 Hz, 2H, *H*_Ar_), 7.45 (d, *J* = 8.0 Hz, 2H, *H*_Ar_), 7.26 (d, *J* = 7.8 Hz, 2H, *H*_Ar_), 6.93 (s, 2H, -*H*C=C*H-*), 5.52 (s, 1H, C*H*OR), 3.49–3.42 (m, 2H, OC*H_2_*CH_3_), 2.33 (s, 3H, ArC*H_3_*), 1.18 (t, *J* = 7.0 Hz, 3H, OCH*_2_*C*H_3_*). ^13^C NMR (101 MHz, Chloroform-*d*) δ 148.6, 141.0, 138.3, 138.0, 137.2, 129.6, 128.9, 127.5, 127.3, 127.2, 127.0, 77.8, 65.1, 21.2, 15.4. HRMS (ESI) C_19_H_21_N_2_O, *m*/*z*: calc. for [M+H]^+^: 293.1648; found: 293.1648.

*2-(isopropoxy(4′-methyl-[1,1′-biphenyl]-4-yl)methyl)-1H-imidazole* (**4o**)

Compound **4o** was synthesized according to general procedure C (yield 62%) from **3f** and *i*-PrOH as a yellow solid. M.p. 174–175 °C. ^1^H NMR (400 MHz, Chloroform-*d*) δ 7.54 (d, *J* = 8.1 Hz, 2H, *H*_Ar_), 7.46–7.42 (m, 4H, *H*_Ar_), 7.23 (d, *J* = 7.8 Hz, 2H, *H*_Ar_), 7.01 (s, 2H, -*H*C=C*H-*), 5.72 (s, 1H, C*H*OR), 3.80–3.74 (m, 1H, OC*H*(CH_3_)_2_), 2.39 (s, 3H, ArC*H_3_*), 1.24 (d, *J* = 6.3 Hz, 3H, OCH(C*H_3_*)_2_), 1.22 (d, *J* = 6.1 Hz, 3H, OCH(C*H_3_*)_2_). ^1^H NMR (400 MHz, DMSO-*d*_6_) δ 11.98 (s, 1H, N*H*), 7.59 (d, *J* = 8.0 Hz, 2H, *H*_Ar_), 7.53 (d, *J* = 7.8 Hz, 2H, *H*_Ar_), 7.44 (d, *J* = 8.1 Hz, 2H, *H*_Ar_), 7.26 (d, *J* = 7.8 Hz, 2H, *H*_Ar_), 6.93 (s, 2H, -*H*C=C*H-*), 5.63 (s, 1H, C*H*OR), 3.62–3.56 (m, 1H, OC*H*(CH_3_)_2_), 2.33 (s, 3H, ArC*H_3_*), 1.16 (d, *J* = 6.1 Hz, 3H, OCH(C*H_3_*)_2_), 1.13 (d, *J* = 6.0 Hz, 3H, OCH(C*H_3_*)_2_). ^13^C NMR (101 MHz, Chloroform-*d*) δ 149.0, 140.8, 138.9, 137.9, 137.1, 129.5, 128.8, 127.2, 127.1, 127.0, 75.1, 70.2, 22.6, 22.1, 21.2. HRMS (ESI) C_20_H_23_N_2_O, *m*/*z*: calc. for [M+H]^+^: 307.1805; found: 307.1809.

*2-(butoxy(4′-methyl-[1,1′-biphenyl]-4-yl)methyl)-1H-imidazole* (**4p**)

Compound **4p** was synthesized according to general procedure C (yield 84%) from **3f** and *n*-BuOH as a white solid. M.p. 138–140 °C. ^1^H NMR (400 MHz, Chloroform-*d*) δ 7.55 (d, *J* = 8.1 Hz, 2H, *H*_Ar_), 7.47–7.41 (m, 4H, *H*_Ar_), 7.23 (d, *J* = 8.0 Hz, 2H, *H*_Ar_), 7.01 (s, 2H, -*H*C=C*H-*), 5.57 (s, 1H, C*H*OR), 3.59–3.49 (m, 2H, O(C*H_2_*)_3_CH_3_), 2.39 (s, 3H, ArC*H_3_*), 1.66–1.60 (m, 2H, O(C*H_2_*)_3_CH_3_), 1.46–1.39 (m, 2H, O(C*H_2_*)_3_CH_3_), 0.92 (t, *J* = 7.4 Hz, 3H, O(CH_2_)_3_C*H_3_*). ^1^H NMR (400 MHz, DMSO-*d*_6_) δ 11.95 (s, 1H, N*H*), 7.60 (d, *J* = 8.2 Hz, 2H, *H*_Ar_), 7.53 (d, *J* = 8.1 Hz, 2H, *H*_Ar_), 7.44 (d, *J* = 8.2 Hz, 2H, *H*_Ar_), 7.25 (d, *J* = 8.0 Hz, 2H, *H*_Ar_), 7.04 (s, 1H, -*H*C=C*H-*), 6.81 (s, 1H, -*H*C=C*H-*), 5.49 (s, 1H, C*H*OR), 3.43–3.38 (m, 2H, O(C*H_2_*)_3_CH_3_), 2.33 (s, 3H, ArC*H_3_*), 1.57–1.52 (m, 2H, O(C*H_2_*)_3_CH_3_), 1.39–1.33 (m, 2H, O(C*H_2_*)_3_CH_3_), 0.86 (t, *J* = 7.4 Hz, 3H, O(CH_2_)_3_C*H_3_*). ^13^C NMR (101 MHz, Chloroform-*d*) δ 148.7, 141.1, 138.3, 138.0, 137.3, 129.6, 128.9, 127.5, 127.3, 127.3, 127.1, 78.0, 69.5, 32.0, 21.2, 19.5, 14.0. HRMS (ESI) C_21_H_25_N_2_O, *m*/*z*: calc. for [M+H]^+^: 321.1961; found: 321.1968.

*(1H-imidazol-2-yl)(4′-methyl-[1,1′-biphenyl]-4-yl)methanol* (**4q**)

Compound **4q** was synthesized from **3f** according to general procedure C via boiling in conc. HCl (light brown solid, yield 85%). M.p. 148–149 °C. ^1^H NMR (400 MHz, DMSO-*d*_6_) δ 7.58 (d, *J* = 8.1 Hz, 2H, *H*_Ar_), 7.53 (d, *J* = 8.0 Hz, 2H, *H*_Ar_), 7.45 (d, *J* = 8.3 Hz, 2H, *H*_Ar_), 7.25 (d, *J* = 8.0 Hz, 2H, *H*_Ar_), 6.92 (s, 2H, -*H*C=C*H-*), 6.22 (s, 1H, OH), 5.77 (s, 1H, C*H*OH), 2.33 (s, 3H, ArC*H_3_*). ^13^C NMR (101 MHz, DMSO-*d*_6_) δ 150.0, 142.0, 138.9, 137.1, 136.6, 129.5, 128.9, 126.9, 126.4, 126.0, 69.2, 20.6. HRMS (ESI) C_17_H_17_N_2_O, *m*/*z*: calc. for [M+H]^+^: 265.1335; found: 265.1342.

*2-(ethoxy(2-(p-tolylethynyl)phenyl)methyl)-1H-imidazole* (**4r**)

Compound **4r** was synthesized according to general procedure C (yield 53%) from **3i** and EtOH as a white solid. M.p. 137–139 °C. ^1^H NMR (400 MHz, Chloroform-*d*) δ 7.55 (d, *J* = 7.1 Hz, 1H, *H*_Ar_), 7.40 (t, *J* = 8.2 Hz, 3H, *H*_Ar_), 7.35–7.29 (m, 2H, *H*_Ar_), 7.15 (d, *J* = 7.8 Hz, 2H, *H*_Ar_), 7.00 (s, 2H, -*H*C=C*H-*), 6.08 (s, 1H, C*H*OR), 3.68–3.61 (m, 2H, OC*H_2_*CH_3_), 2.36 (s, 3H, ArC*H_3_*), 1.26 (t, *J* = 7.0 Hz, 1H, OCH*_2_*C*H_3_*). ^1^H NMR (400 MHz, DMSO-*d*_6_) δ 12.13 (s, 1H, N*H*), 7.57 (d, *J* = 7.9 Hz, 1H, *H*_Ar_), 7.51 (d, *J* = 7.5 Hz, 1H, *H*_Ar_), 7.47 (d, *J* = 8.0 Hz, 2H, *H*_Ar_), 7.44–7.39 (m, 1H, *H*_Ar_), 7.34 (t, *J* = 7.6 Hz, 1H, *H*_Ar_), 7.25 (d, *J* = 8.2 Hz, 2H, *H*_Ar_), 6.95 (s, 2H, =C*H*), 5.94 (s, 1H, C*H*OR), 3.49 (q, *J* = 7.1 Hz, 2H, OC*H_2_*CH_3_), 2.34 (s, 3H, ArC*H_3_*), 1.16 (t, *J* = 7.0 Hz, 3H, OCH*_2_*C*H_3_*). ^13^C NMR (101 MHz, Chloroform-*d*) δ 147.9, 140.9, 138.8, 133.2, 132.7, 131.6, 129.8, 129.3, 128.8, 128.2, 127.9, 127.4, 123.0, 120.1, 94.6, 86.6, 76.1, 65.2, 21.6, 15.4. HRMS (ESI) C_26_H_25_N_4_O_2_S, *m*/*z*: calc. for [M+H]^+^: 317.1648; found: 317.1655.

*2-(ethoxy(2-ethynylphenyl)methyl)-1H-imidazole* (**4s**)

Compound **4s** was synthesized according to general procedure C (yield 91%) from **3h** and EtOH as a light brown solid. M.p. 148–150 °C. ^1^H NMR (400 MHz, Chloroform-*d*) δ 7.52 (d, *J* = 7.8 Hz, 1H, *H*_Ar_), 7.37 (dd, *J* = 11.2, 7.4 Hz, 2H, *H*_Ar_), 7.28 (d, *J* = 1.0 Hz, 1H, *H*_Ar_), 7.00 (s, 2H, -*H*C=C*H-*), 6.04 (s, 1H, C*H*OR), 3.63–3.57 (m, 2H, OC*H_2_*CH_3_), 3.32 (s, 1H, Ar-CC*H*), 1.28–1.25 (m, 3H, OCH*_2_*C*H_3_*). ^1^H NMR (400 MHz, DMSO-*d*_6_) δ 12.11 (s, 1H, N*H*), 7.53 (d, *J* = 8.0 Hz, 1H, *H*_Ar_), 7.46 (d, *J* = 7.7 Hz, 1H, *H*_Ar_), 7.42 (t, *J* = 7.5 Hz, 1H, *H*_Ar_), 7.31 (t, *J* = 7.6 Hz, 1H, *H*_Ar_), 6.93 (s, 2H, -*H*C=C*H-*), 5.87 (s, 1H, C*H*OR), 4.41 (s, 1H, Ar-CC*H*), 3.46–3.40 (m, 2H, OC*H_2_*CH_3_), 1.14 (t, *J* = 7.0 Hz, 3H, OCH*_2_*C*H_3_*). ^13^C NMR (101 MHz, Chloroform-*d*) δ 147.8, 141.8, 133.3, 129.5, 128.1, 127.1, 121.7, 82.4, 81.4, 75.8, 65.1, 29.8. HRMS (ESI) C_14_H_15_N_2_O, *m*/*z*: calc. for [M+H]^+^: 227.1179; found: 227.1183.

*2-(ethoxy(4-ethynylphenyl)methyl)-1H-imidazole* (**4t**)

Compound **4t** was synthesized according to general procedure C (yield 76%) from **3g** and EtOH as a white solid. M.p. 155–157 °C. ^1^H NMR (400 MHz, DMSO-*d*_6_) δ 11.90 (s, 1H, N*H*), 7.44 (d, *J* = 8.1 Hz, 2H, *H*_Ar_), 7.38 (d, *J* = 8.0 Hz, 2H, *H*_Ar_), 7.06–6.75 (m, 2H, -*H*C=C*H-*), 5.50 (s, 1H, C*H*OR), 4.08 (s, 1H, Ar-CC*H*), 3.49–3.43 (m, 2H, OC*H_2_*CH_3_), 1.17 (t, *J* = 7.0 Hz, 3H, OCH*_2_*C*H_3_*). ^13^C NMR (101 MHz, Chloroform-*d*) δ 148.0, 140.4, 132.3, 126.8, 122.2, 121.8, 112.9, 83.4, 77.5, 77.5, 65.1, 15.3. HRMS (ESI) C_14_H_15_N_2_O, *m*/*z*: calc. for [M+H]^+^: 227.1179; found: 227.1181.

*4-(4-(ethoxy(1H-imidazol-2-yl)methyl)phenyl)-7-(4-methoxyphenyl)benzo[c][1,2,5]thiadiazole* (**4u**)

Compound **4u** was synthesized according to general procedure C (yield 86%) from **3l** and EtOH as a yellow solid. M.p. 164–166 °C. ^1^H NMR (400 MHz, Chloroform-*d*) δ 7.96–7.91 (m, 4H, *H*_Ar_), 7.73 (s, 2H, *H*_Ar_), 7.57 (d, *J* = 8.2 Hz, 2H, *H*_Ar_), 7.08 (d, *J* = 8.7 Hz, 2H, *H*_Ar_), 7.03 (s, 2H, -*H*C=C*H-*), 5.67 (s, 1H, C*H*OR), 3.90 (s, 3H, ArOC*H_3_*), 3.71–3.60 (m, 2H, OC*H_2_*CH_3_), 1.31 (d, *J* = 7.0 Hz, 3H, OCH*_2_*C*H_3_*). ^13^C NMR (101 MHz, Chloroform-*d*) δ 160.0, 154.2, 148.5, 139.6, 137.5, 133.2, 132.3, 130.6, 130.0, 129.6, 128.4, 127.4, 127.1, 114.3, 77.8, 65.3, 55.6, 15.4. HRMS (ESI) C_25_H_23_N_4_O_2_S, *m*/*z*: calc. for [M+H]^+^: 443.1536; found: 443.1541.

*4-(4-((1H-imidazol-2-yl)(isopropoxy)methyl)phenyl)-7-(4-methoxyphenyl)benzo[c][1,2,5]thiadiazole* (**4v**)

Compound **4v** was synthesized according to general procedure C (yield 76%) from **3l** and *i*-PrOH as a yellow solid. M.p. 175–177 °C. ^1^H NMR (400 MHz, Chloroform-*d*) δ 7.92 (dd, *J* = 8.2, 5.1 Hz, 4H, *H*_Ar_), 7.71 (s, 2H, *H*_Ar_), 7.57 (d, *J* = 8.0 Hz, 2H, *H*_Ar_), 7.08 (d, *J* = 8.3 Hz, 2H, *H*_Ar_), 7.02 (s, 2H, -*H*C=C*H-*), 5.79 (s, 1H, C*H*OR), 3.89 (s, 3H, ArOC*H_3_*), 3.85–3.80 (m, 1H, OC*H*(CH_3_)_2_), 1.27 (d, *J* = 6.3 Hz, 3H, OCH(C*H_3_*)_2_), 1.24 (d, *J* = 6.0 Hz, 3H, OCH(C*H_3_*)_2_). ^1^H NMR (400 MHz, DMSO-*d*_6_) δ 11.86 (s, 1H, N*H*), 8.00 (d, *J* = 8.7 Hz, 2H, *H*_Ar_), 7.97 (d, *J* = 8.1 Hz, 2H, *H*_Ar_), 7.90 (s, 2H), 7.56 (d, *J* = 8.0 Hz, 2H, *H*_Ar_), 7.12 (d, *J* = 8.5 Hz, 2H, *H*_Ar_), 7.04 (s, 1H, -*H*C=C*H-*), 6.84 (s, 1H, -*H*C=C*H-*), 5.69 (s, 1H, C*H*OR), 3.86 (s, 3H, ArOC*H_3_*), 3.72–3.66 (m, 1H, OC*H*(CH_3_)_2_), 1.21 (d, *J* = 6.0 Hz, 3H, OCH(C*H_3_*)_2_), 1.17 (d, *J* = 6.0 Hz, 3H, OCH(C*H_3_*)_2_). ^13^C NMR (101 MHz, Chloroform-*d*) δ 160.0, 154.3, 149.0, 140.2, 137.3, 133.2, 132.3, 130.6, 130.0, 129.5, 128.4, 127.4, 127.1, 114.3, 75.1, 70.5, 55.5, 22.8, 22.1. HRMS (ESI) C_26_H_25_N_4_O_2_S, *m*/*z*: calc. for [M+H]^+^: 457.1693; found: 457.1696.

*1,4-bis(ethoxy(1H-imidazol-2-yl)methyl)benzene* (**4w**)

Compound **4w** was synthesized according to general procedure C (yield 44%) from **3j** and EtOH as a white solid. M.p. 196–198 °C. ^1^H NMR (400 MHz, DMSO-*d*_6_) δ 11.97 (s, 2H, N*H*), 7.35 (s, 4H, *H*_Ar_), 6.90 (s, 4H, -*H*C=C*H-*), 5.45 (s, 2H, C*H*OR), 3.43–3.38 (m, 4H, OC*H_2_*CH_3_), 1.14 (t, *J* = 7.0 Hz, 6H, OCH*_2_*C*H_3_*). ^13^C NMR (101 MHz, DMSO-*d*_6_) δ 147.4, 139.6, 126.6, 126.6, 77.1, 63.9, 15.1. HRMS (ESI) C_18_H_23_N_4_O_2_, *m*/*z*: calc. for [M+H]^+^: 327.1816; found: 327.1820.

*1,4-bis((1H-imidazol-2-yl)(isopropoxy)methyl)benzene* (**4x**)

Compound **4x** was synthesized according to general procedure C (yield 76%) from **3j** and *i*-PrOH as a white solid. M.p. 206–207 °C. ^1^H NMR (400 MHz, DMSO-*d*_6_) δ 11.87 (s, 2H, N*H*), 7.33 (s, 4H, *H*_Ar_), 7.00 (s, 2H, -*H*C=C*H-*), 6.76 (s, 2H, -*H*C=C*H-*), 5.55 (s, 2H, C*H*OR), 3.56–3.50 (m, 2H, OC*H*(CH_3_)_2_), 1.12 (d, *J* = 6.1 Hz, 6H, OCH(C*H_3_*)_2_), 1.09 (d, *J* = 6.1 Hz, 6H, OCH(C*H_3_*)_2_). ^13^C NMR (101 MHz, DMSO-*d*_6_) δ 147.9, 140.0, 126.5, 126.5, 116.5, 74.6, 69.0, 22.0. HRMS (ESI) C_20_H_27_N_4_O_2_, *m*/*z*: calc. for [M+H]^+^: 355.2129; found: 355.2136.

*1-benzyl-4-(2-(ethoxy(1H-imidazol-2-yl)methyl)phenyl)-1H-1,2,3-triazole* (**4y**)

Compound **4y** was synthesized according to general procedure C from **3m** and EtOH (yield 66%) or general procedure D from **3i** (yield 98%) as a white solid. M.p. 147–149 °C. ^1^H NMR (400 MHz, Chloroform-*d*) δ 7.85–7.81 (m, 2H, *H*_Ar_), 7.41–7.35 (m, 7H, *H*_Ar_), 7.31 (d, *J* = 7.5 Hz, 1H, *H*_Ar_), 7.03 (s, 2H, -*H*C=C*H-*), 6.00 (s, 1H, C*H*OR), 5.63 (s, 2H, ArC*H_2_*), 3.46–3.35 (m, 2H, OC*H_2_*CH_3_), 1.06 (t, *J* = 6.8 Hz, 3H, OCH*_2_*C*H_3_*). ^1^H NMR (400 MHz, DMSO-*d*_6_) δ 8.62 (s, 1H, N*H*), 7.64–7.58 (m, 2H, *H*_Ar_), 7.44–7.40 (m, 3H, *H*_Ar_), 7.38 (s, 1H, *H*_Ar_), 7.36–7.34 (m, 3H, *H*_Ar_), 7.09 (s, 2H, -*H*C=C*H-*), 6.07 (s, 1H, C*H*OR), 5.69 (s, 2H, ArC*H_2_*), 3.35 (dd, *J* = 6.9, 3.0 Hz, 2H, OC*H_2_*CH_3_), 1.05 (t, *J* = 7.0 Hz, 3H, OCH*_2_*C*H_3_*). ^13^C NMR (101 MHz, DMSO-*d*_6_) δ 145.2, 137.3, 135.9, 133.1, 129.6, 129.1, 128.8, 128.1, 127.9, 127.8, 127.6, 124.2, 73.6, 63.9, 53.0, 15.0. HRMS (ESI) C_21_H_22_N_5_O, *m*/*z*: calc. for [M+H]^+^: 360.1819; found: 360.1824.

*1-benzyl-4-(4-(ethoxy(1H-imidazol-2-yl)methyl)phenyl)-1H-1,2,3-triazole* (**4z**)

Compound **4z** was synthesized according to general procedure C from **3n** and EtOH (yield 75%) and general procedure D from **3g** (yield 92%) as a white solid. M.p. 167–169 °C. ^1^H NMR (400 MHz, Chloroform-*d*) δ 7.75 (d, *J* = 8.1 Hz, 2H, *H*_Ar_), 7.64 (s, 1H, *H*_Ar_), 7.42–7.37 (m, 5H, *H*_Ar_), 7.31–7.28 (m, 2H, *H*_Ar_), 7.00 (s, 2H, -*H*C=C*H-*), 5.58 (s, 1H, C*H*OR), 5.57 (s, 2H, ArC*H_2_*), 3.60–3.54 (m, 2H, OC*H_2_*CH_3_), 1.26–1.24 (m, 3H, OCH*_2_*C*H_3_*). ^1^H NMR (400 MHz, DMSO-*d*_6_) δ 12.02 (s, 1H, N*H*), 8.61 (s, 1H, *H*_Ar_), 7.81 (d, *J* = 8.2 Hz, 2H, *H*_Ar_), 7.44 (d, *J* = 8.2 Hz, 2H, *H*_Ar_), 7.36 (q, *J* = 6.8 Hz, 5H, *H*_Ar_), 6.93 (s, 2H, -*H*C=C*H-*), 5.64 (s, 2H, ArC*H_2_*), 5.50 (s, 1H, C*H*OR), 3.46–3.41 (m, 2H, OC*H_2_*CH_3_), 1.17 (t, *J* = 7.0 Hz, 3H, OCH*_2_*C*H_3_*). ^13^C NMR (101 MHz, Chloroform-*d*) δ 147.9, 139.4, 134.8, 130.4, 129.3, 128.9, 128.2, 127.4, 126.0, 119.8, 65.1, 54.3, 15.3. HRMS (ESI) C_21_H_22_N_5_O, *m*/*z*: calc. for [M+H]^+^: 360.1819; found: 360.1823.

*3-(1H-imidazol-2-yl)-[1,2,3]triazolo [1,5-a]pyridine* (**5a**)

Compound **5a** was synthesized according to general procedure C (yield 78%) from **3k** in EtOH as a white solid. M.p. 210–212 °C. ^1^H NMR (400 MHz, DMSO-*d*_6_) δ 12.92 (s, 1H, N*H*), 9.12 (d, *J* = 7.0 Hz, 1H, *H*_Ar_), 8.38 (d, *J* = 8.8 Hz, 1H, *H*_Ar_), 7.56–7.51 (m, 1H, *H*_Ar_), 7.27 (t, *J* = 6.9 Hz, 1H, *H*_Ar_), 7.24 (s, 1H, -*H*C=C*H-*), 7.11 (s, 1H, -*H*C=C*H-*). ^13^C NMR (101 MHz, DMSO-*d*_6_) δ 139.4, 130.3, 130.2, 129.0, 127.0, 125.8, 119.1, 117.1, 116.7. HRMS (ESI) C_9_H_8_N_5_, *m*/*z*: calc. for [M+H]^+^: 186.0774; found: 186.0774.

## 4. Conclusions

In summary, we elaborated on a highly efficient transition metal-free access to new functionalized 1*H*-imidazole derivatives. The method is based on the acid-mediated denitrogenative transformation of 5-amino-4-aryl-1-(2,2-diethoxyethyl)-1,2,3-triazole derivatives involving intramolecular cyclization of 5-amino-1,2,3-triazole acetals followed by triazole ring opening and insertion of in situ formed carbene intermediate into the O-H bond of different alcohols to afford the previously inaccessible 2-substituted imidazoles in good to excellent yields with a broad substrate scope. To the best of our knowledge, this type of transformation has not been previously investigated for 5-amino-1,2,3-triazole derivatives.

## Data Availability

Data are contained within the article and Supplementary Materials.

## Supplementary Materials

The following supporting information can be downloaded at https://www.mdpi.com/article/10.3390/molecules30071401/s1: The following are available in File S1: copies of the ^1^H and ^13^C NMR spectra for all novel compounds as well as the crystallography data for **4d** and **5a**.
