# Supplementary material for: Synthesis of Functionalized 1H-Imidazoles via Denitrogenative Transformation of 5-Amino-1,2,3-Triazoles
Source: Molecules. 2025 Mar 21;30(7):1401. doi: 10.3390/molecules30071401 (PMC11990592; doi:10.3390/molecules30071401)
Supplement: Supplementary file 1 [file molecules-30-01401-s001.zip › molecules-3526493-supplementary.pdf]

Supporting information *for*

Synthesis of functionalized 1*H*-imidazoles *via* denitrogenative transformation of 5-amino-1,2,3-triazoles

Pavel S. Gribanov, Anna N. Philippova, Diana N. Tukhvatullina, Viktoria A. Vlasova, Maxim A. Topchiy, Alexander F. Smol'yakov, Andrey F. Asachenko and Sergey N. Osipov

X-ray crystallography of 4d and 5a:

Table S1. Crystal data and structure refinement for **4d** (CCDC 2426762) and **5a** (CCDC 2426763).

Identification code	**4d**	**5a**
Empirical formula	$C_{13}H_{16}N_2O$	$C_9H_7N_5$
Formula weight	216.28	185.20
Temperature/K	120	120
Crystal system	monoclinic	monoclinic
Space group	$P2_1/n$	$P2_1/n$
a/Å	9.9055(7)	4.1391(3)
b/Å	18.8699(14)	16.4192(14)
c/Å	12.9213(10)	12.1450(9)
α/°	90	90
β/°	92.299(3)	98.262(4)
γ/°	90	90
Volume/Å^3	2413.3(3)	816.82(11)
Z	8	4
ρ_{calc}g/cm^3	1.191	1.506
μ/mm^{-1}	0.077	0.101
F(000)	928.0	384.0
Crystal size/mm^3	0.42 × 0.33 × 0.21	0.33 × 0.08 × 0.04
Radiation	MoKα (λ = 0.71073)	MoKα (λ = 0.71073)
2Θ range for data collection/°	3.822 to 51.996	4.2 to 51.984
Index ranges	$-12 \leq h \leq 12$, $-23 \leq k \leq 23$, $-15 \leq l \leq 15$	$-5 \leq h \leq 5$, $-20 \leq k \leq 20$, $-13 \leq l \leq 14$
Reflections collected	33287	8267
Independent reflections	4736 [R_{int} = 0.0486, R_{sigma} = 0.0390]	1584 [R_{int} = 0.0338, R_{sigma} = 0.0324]
Data/restraints/parameters	4736/0/293	1584/0/127
Goodness-of-fit on F^2	1.033	1.059
Final R indexes [I>=2σ (I)]	R_1 = 0.0674, wR_2 = 0.1739	R_1 = 0.0339, wR_2 = 0.0835
Final R indexes [all data]	R_1 = 0.0843, wR_2 = 0.1842	R_1 = 0.0438, wR_2 = 0.0859
Largest diff. peak/hole / e Å^{-3}	0.98/-0.37	0.19/-0.22

NMR spectra

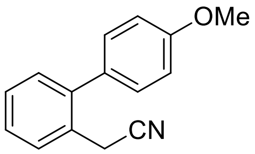

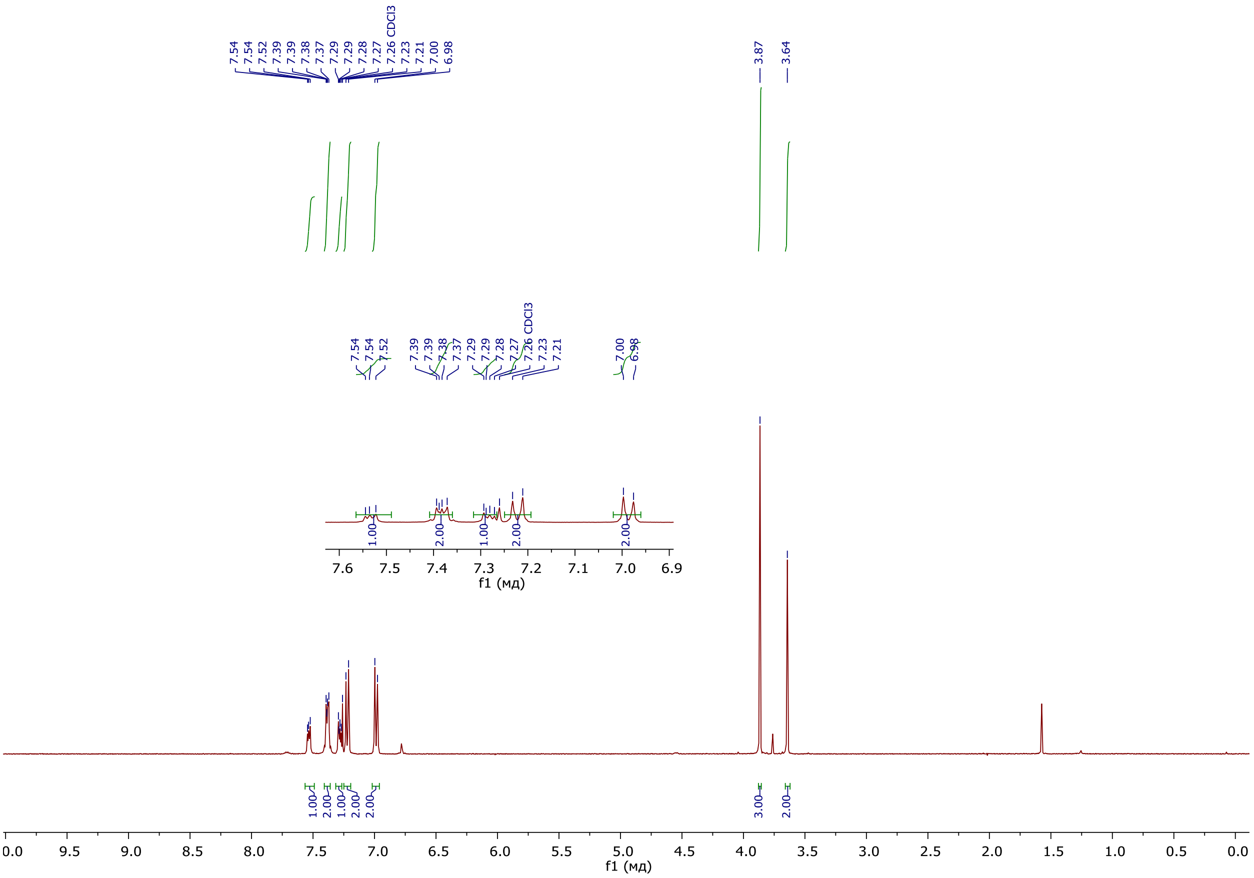

Figure S1. ^{1}H NMR (400 MHz, Chloroform-*d*) spectrum of 2-(4'-methoxy-[1,1'-biphenyl]-2-yl)acetonitrile.

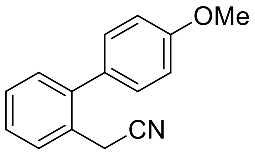

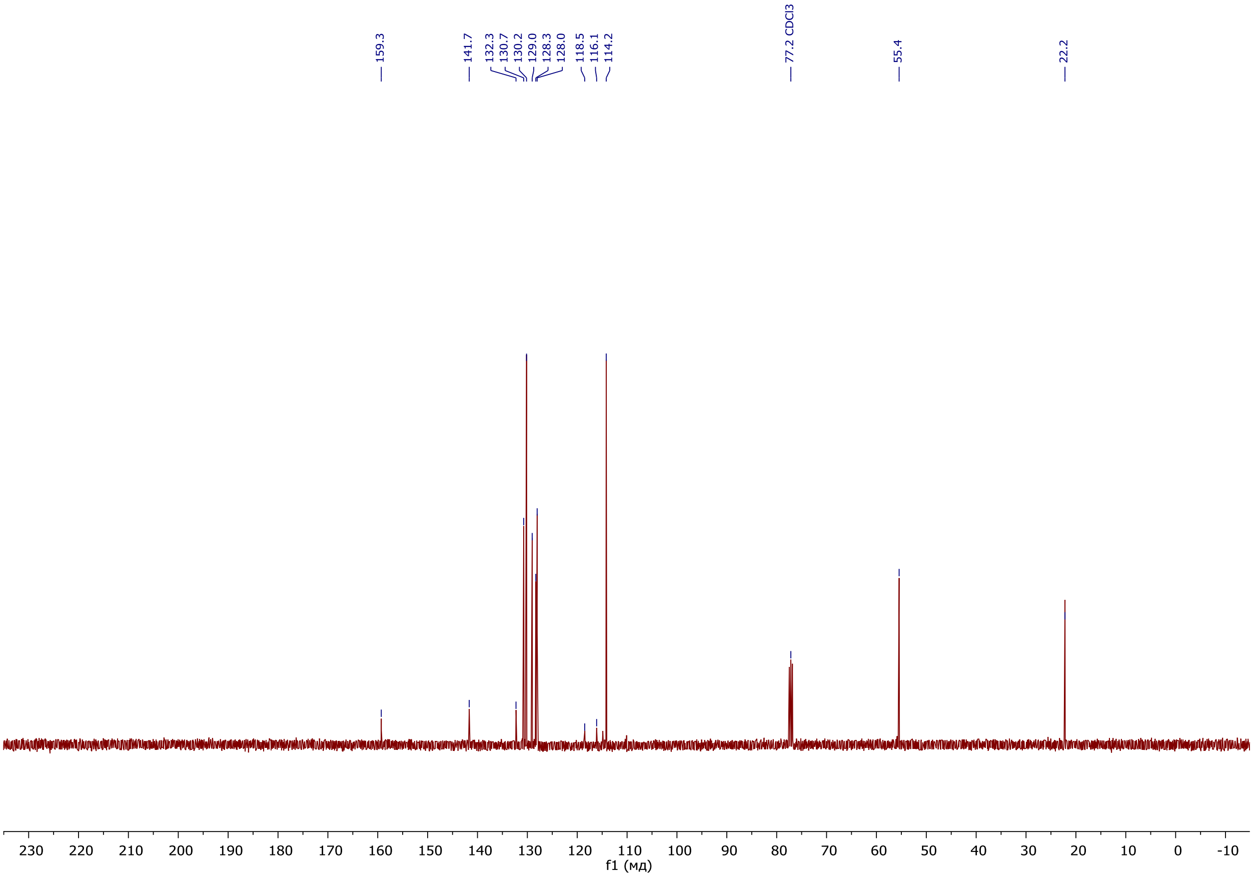

Figure S2. ^{13}C NMR (101 MHz, Chloroform-*d*) spectrum of 2-(4'-methoxy-[1,1'-biphenyl]-2-yl)acetonitrile

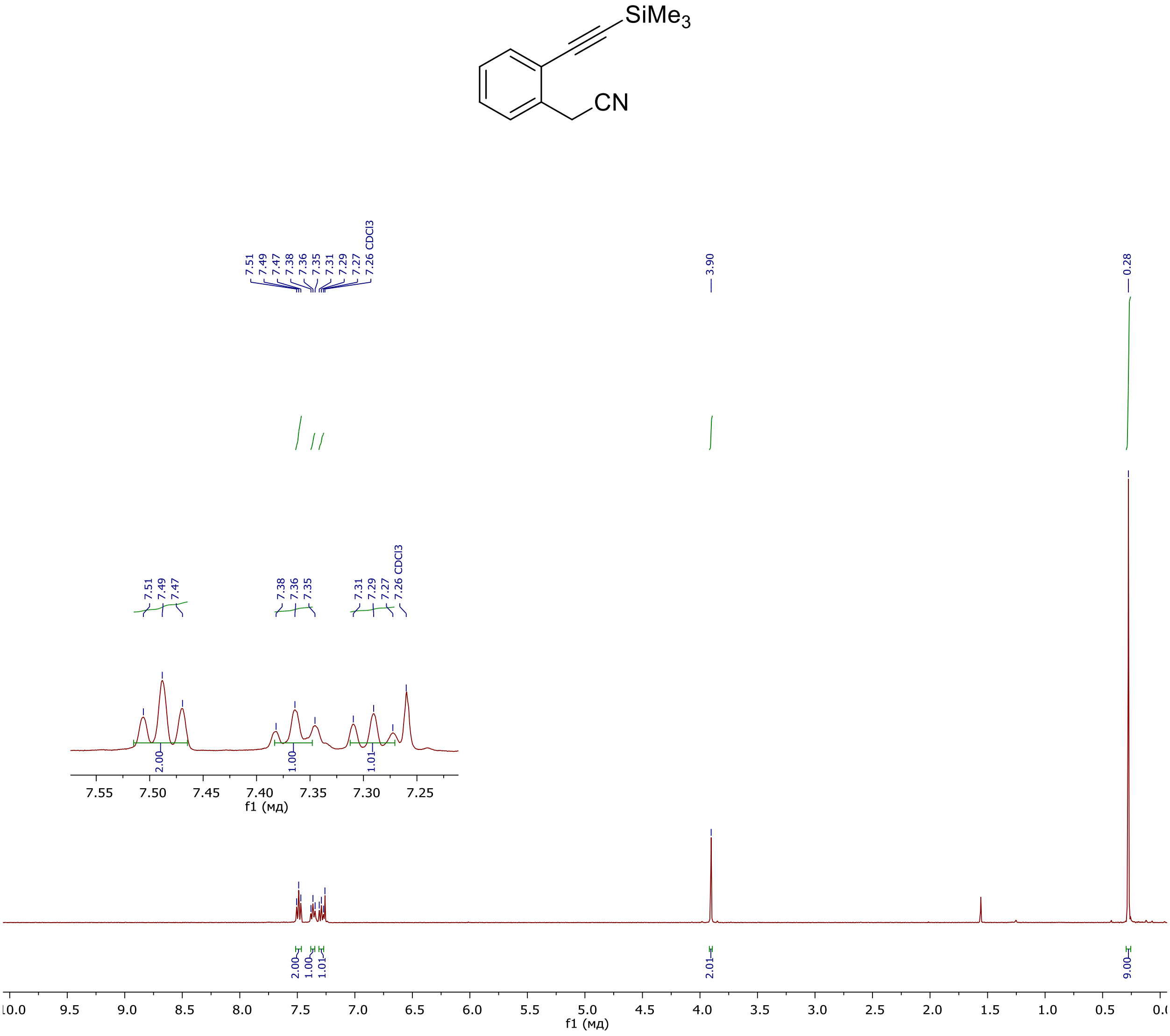

Figure S3. ^{1}H NMR (400 MHz, Chloroform-*d*) spectrum of 2-(2-((trimethylsilyl)ethynyl) phenyl)acetonitrile.

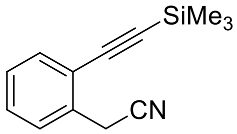

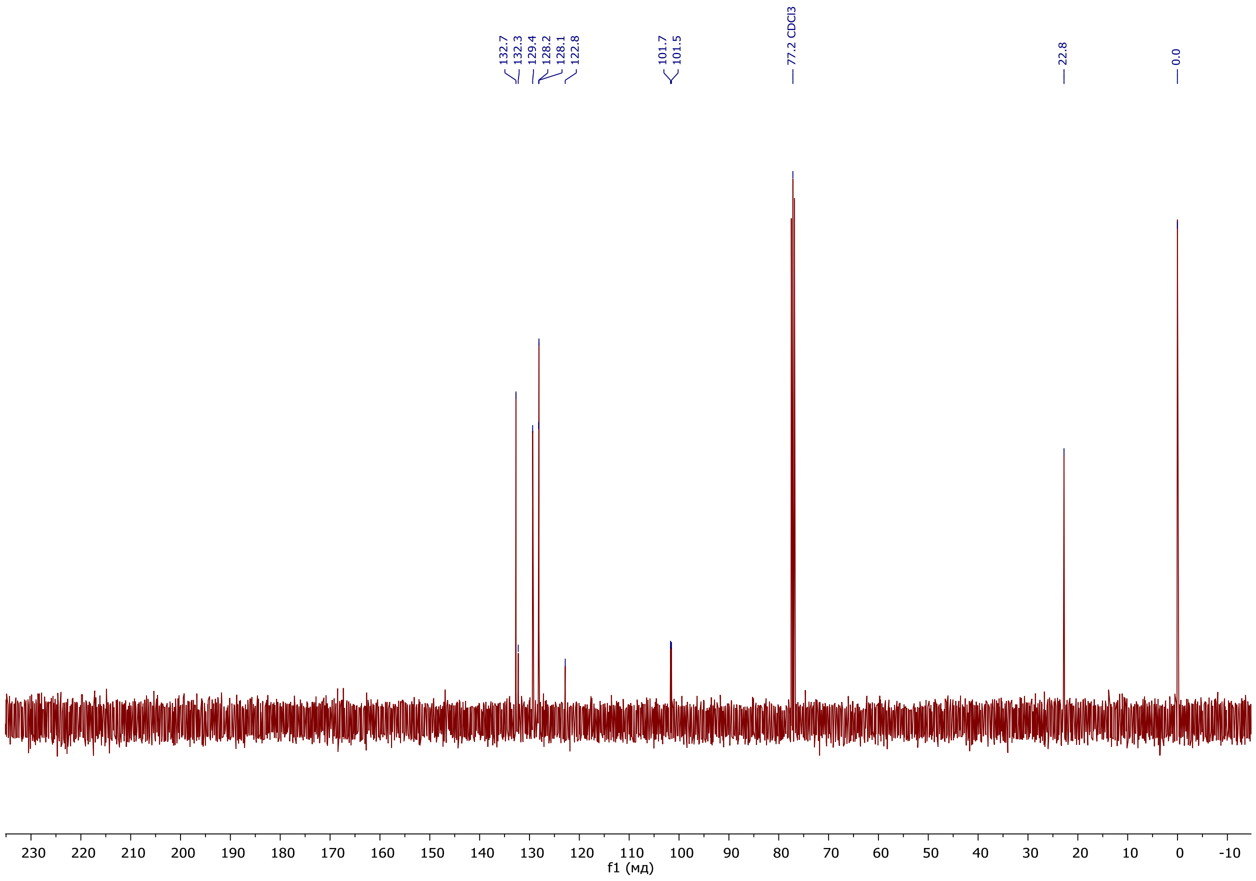

Figure S4. ^{13}C NMR (101 MHz, Chloroform-*d*) spectrum of 2-(2-((trimethylsilyl)ethynyl) phenyl)acetonitrile.

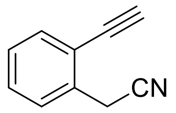

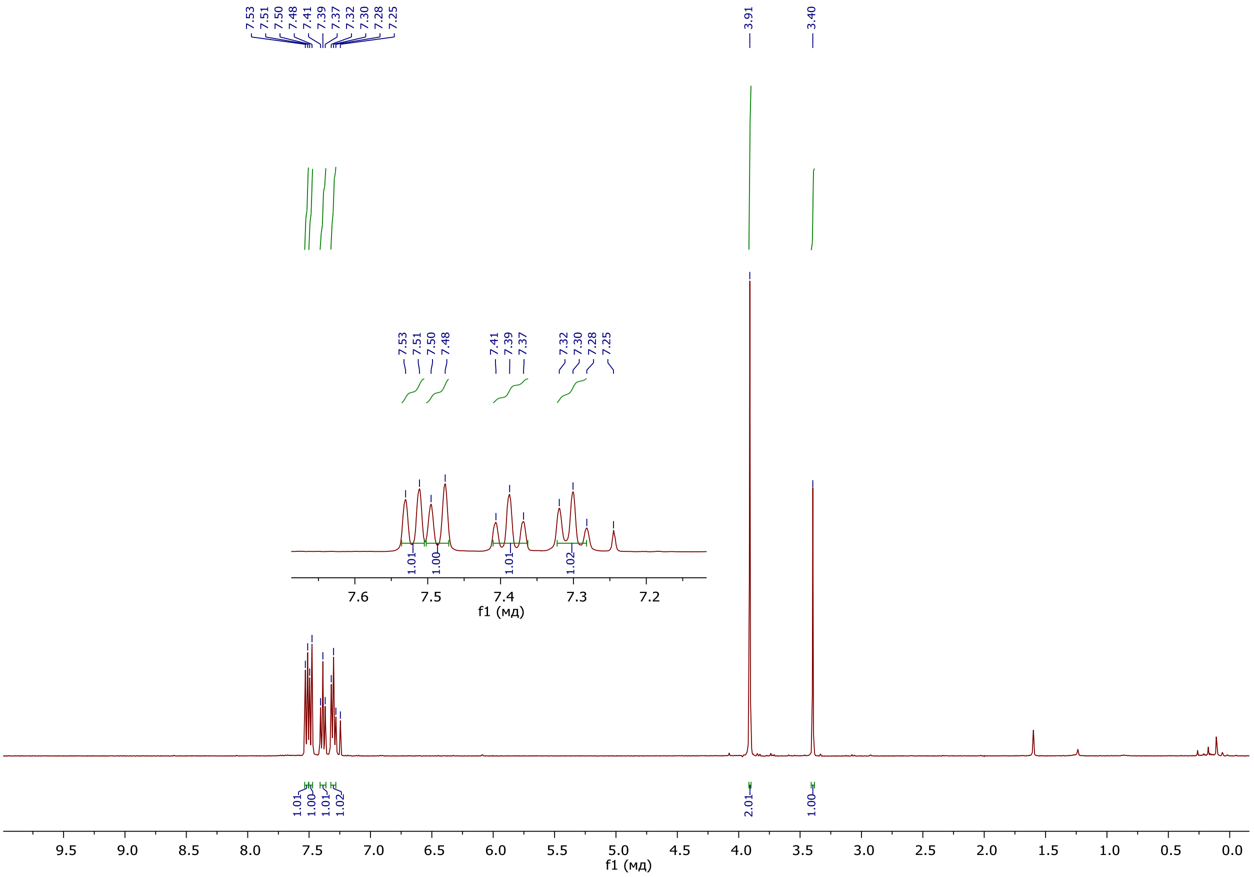

Figure S5. ^{1}H NMR (400 MHz, Chloroform-*d*) spectrum of 2-(2-ethynylphenyl)acetonitrile.

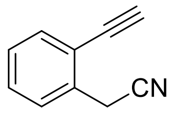

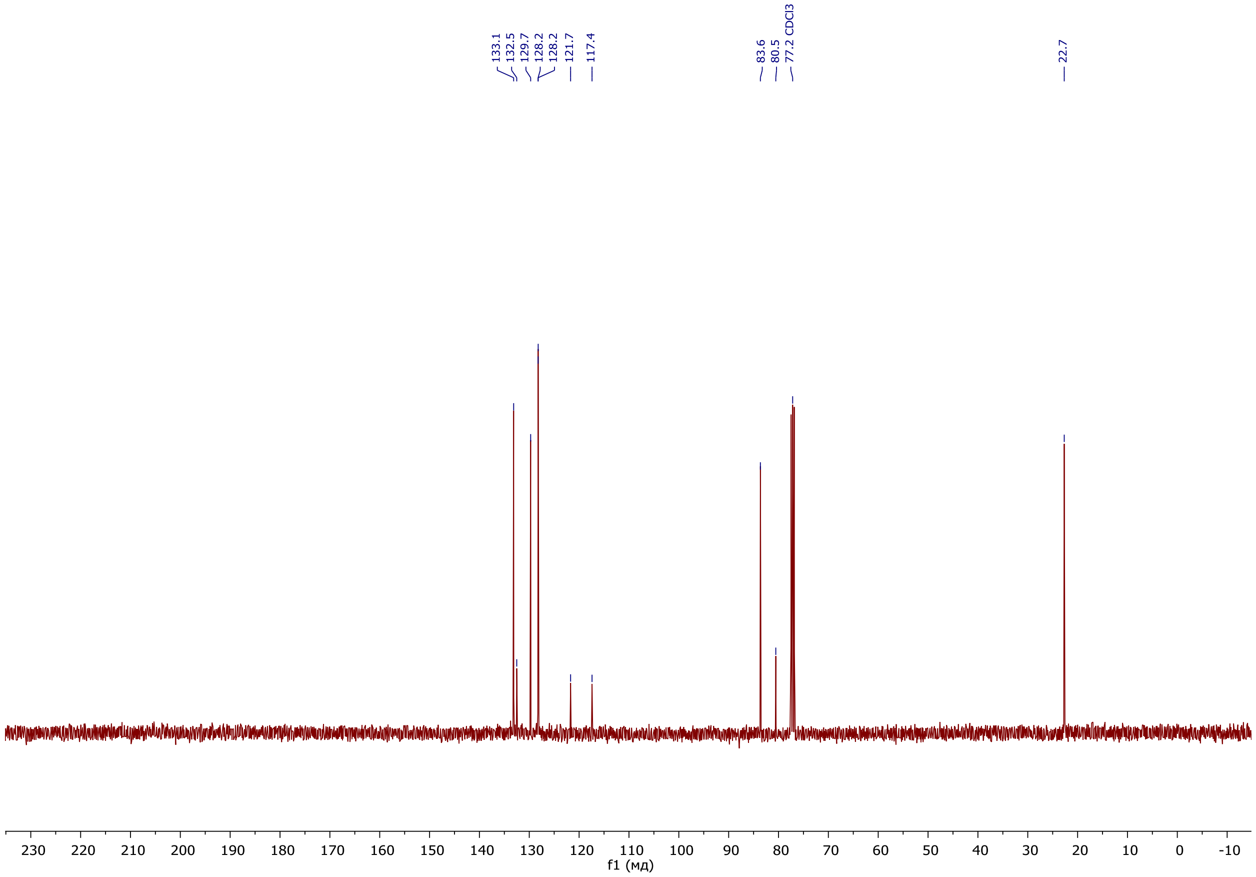

Figure S6. ^{13}C NMR (101 MHz, Chloroform-*d*) spectrum of 2-(2-ethynylphenyl)acetonitrile.

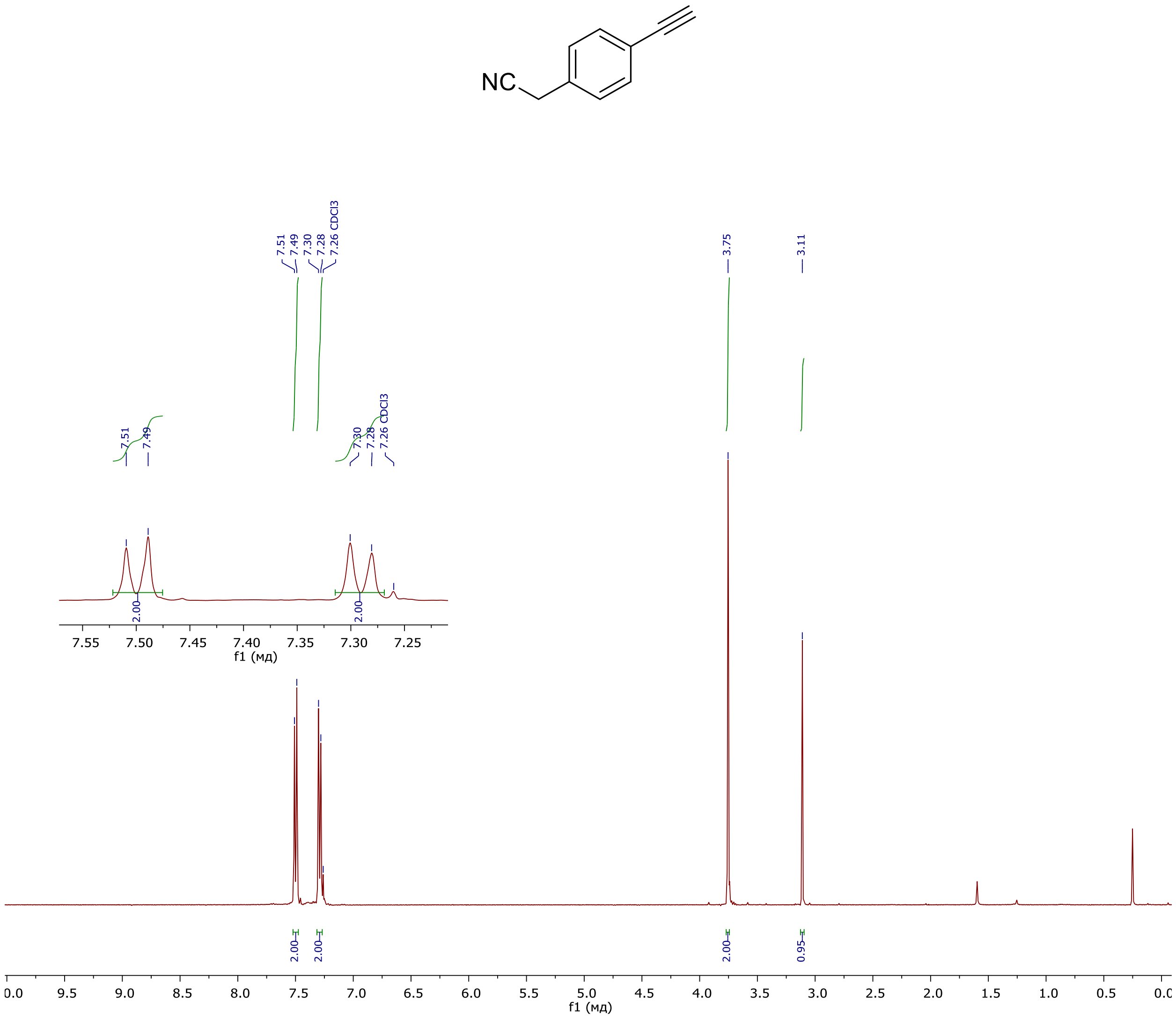

Figure S7. ^{1}H NMR (400 MHz, Chloroform-*d*) spectrum of 2-(4-ethynylphenyl)acetonitrile.

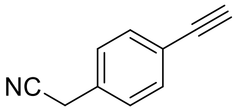

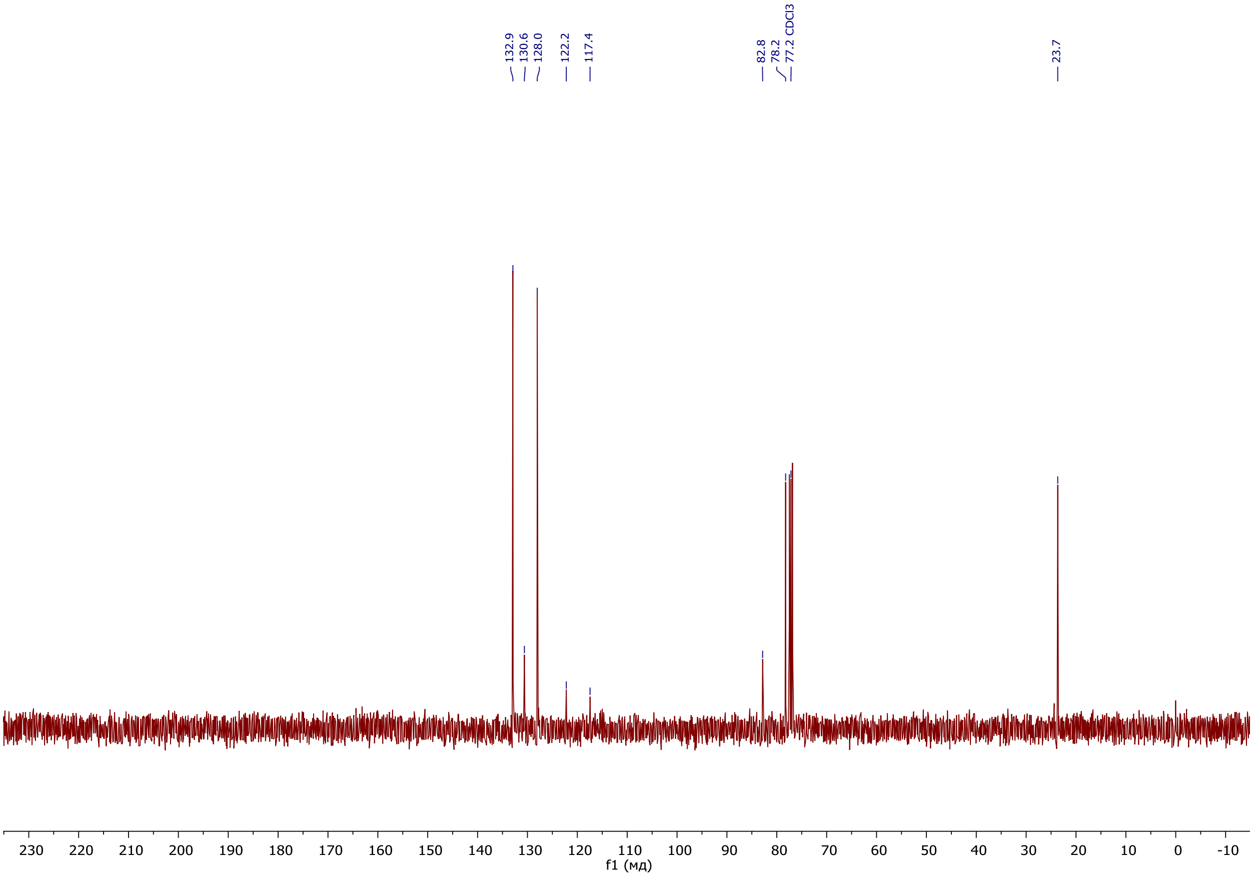

Figure S8. ^{13}C NMR (101 MHz, Chloroform-*d*) spectrum of 2-(4-ethynylphenyl)acetonitrile.

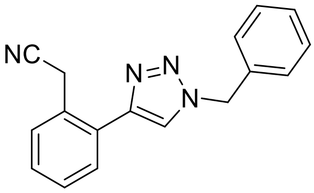

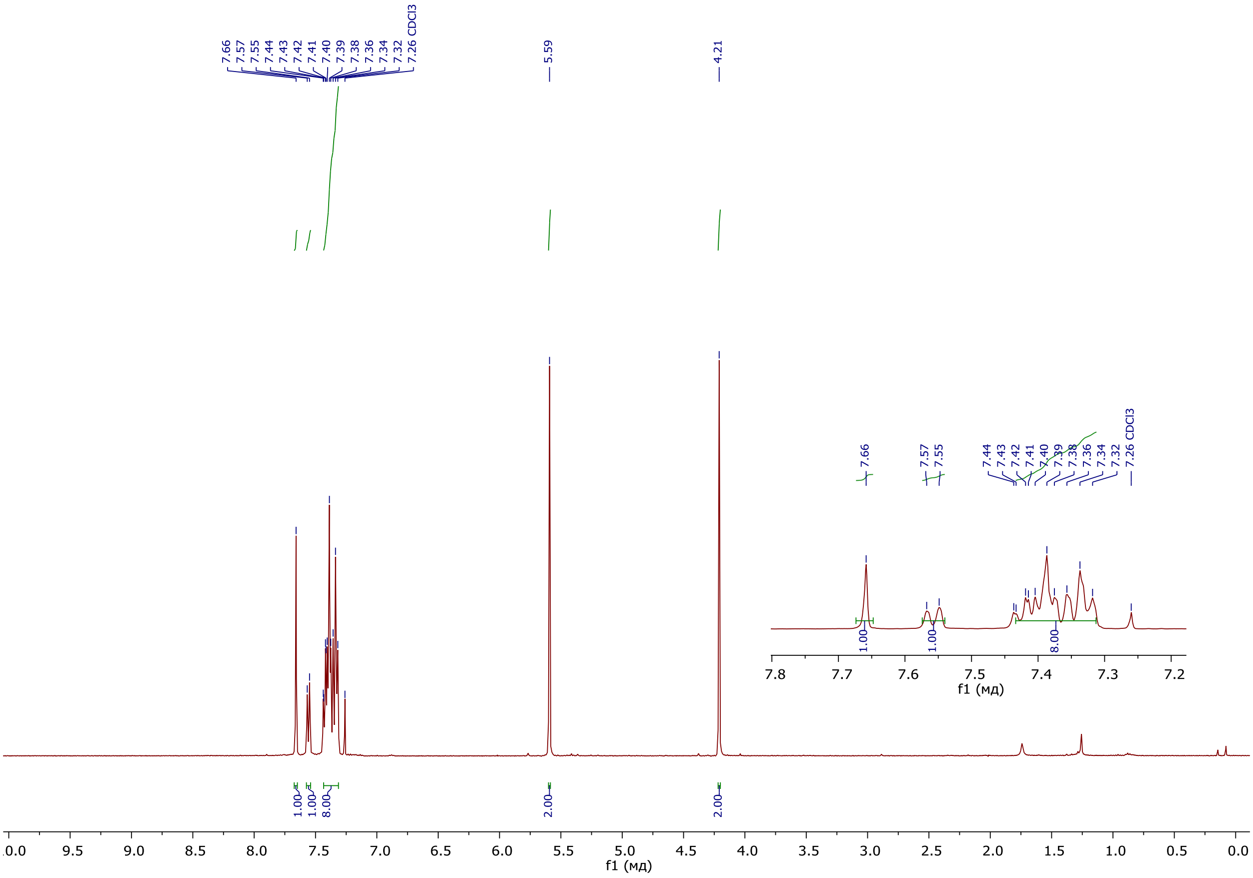

Figure S9. ^{1}H NMR (400 MHz, Chloroform-*d*) spectrum of 2-(2-(1-benzyl-1H-1,2,3-triazol-4-yl)phenyl)acetonitrile.

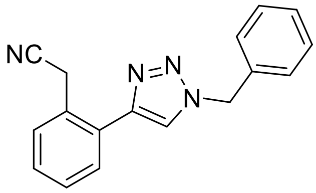

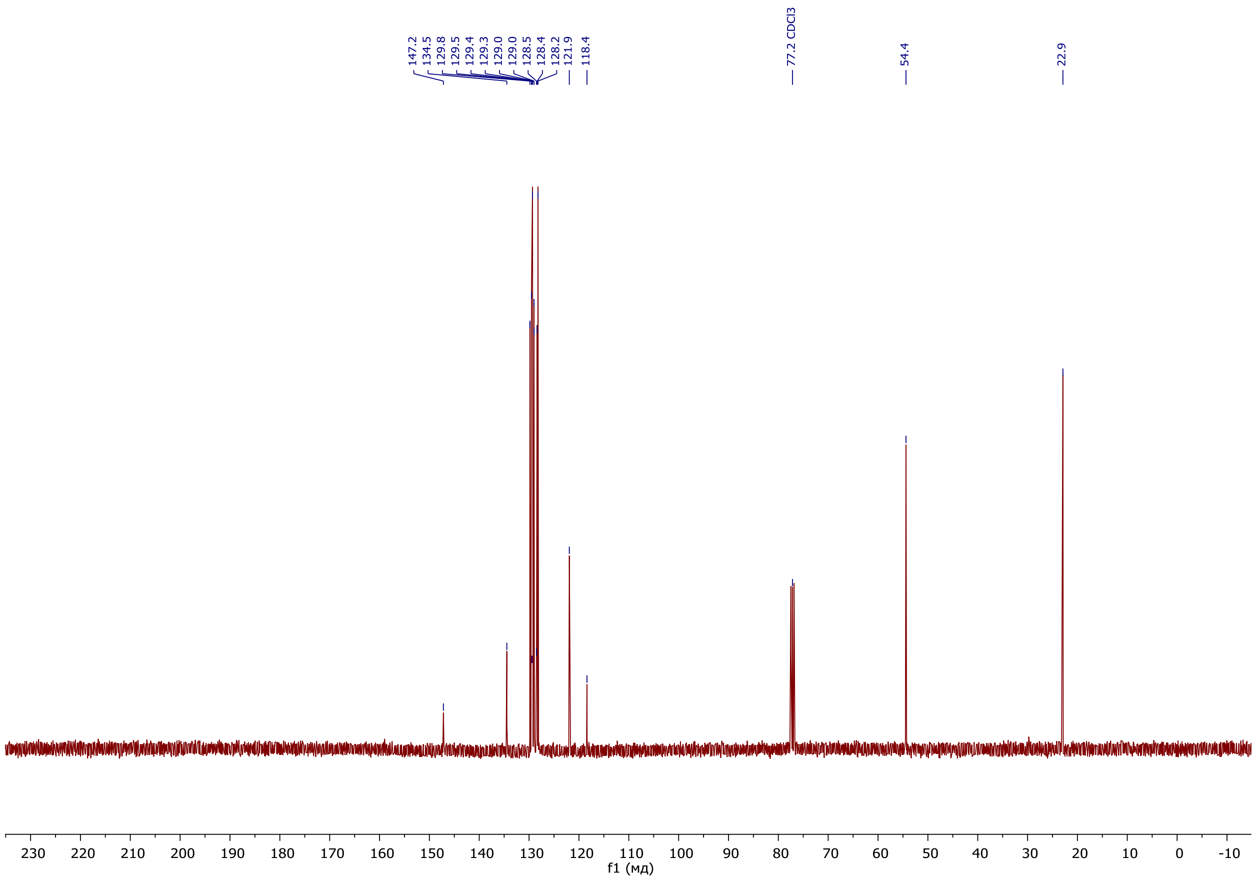

Figure S10. ^{13}C NMR (101 MHz, Chloroform-*d*) spectrum of 2-(2-(1-benzyl-1H-1,2,3-triazol-4-yl)phenyl)acetonitrile.

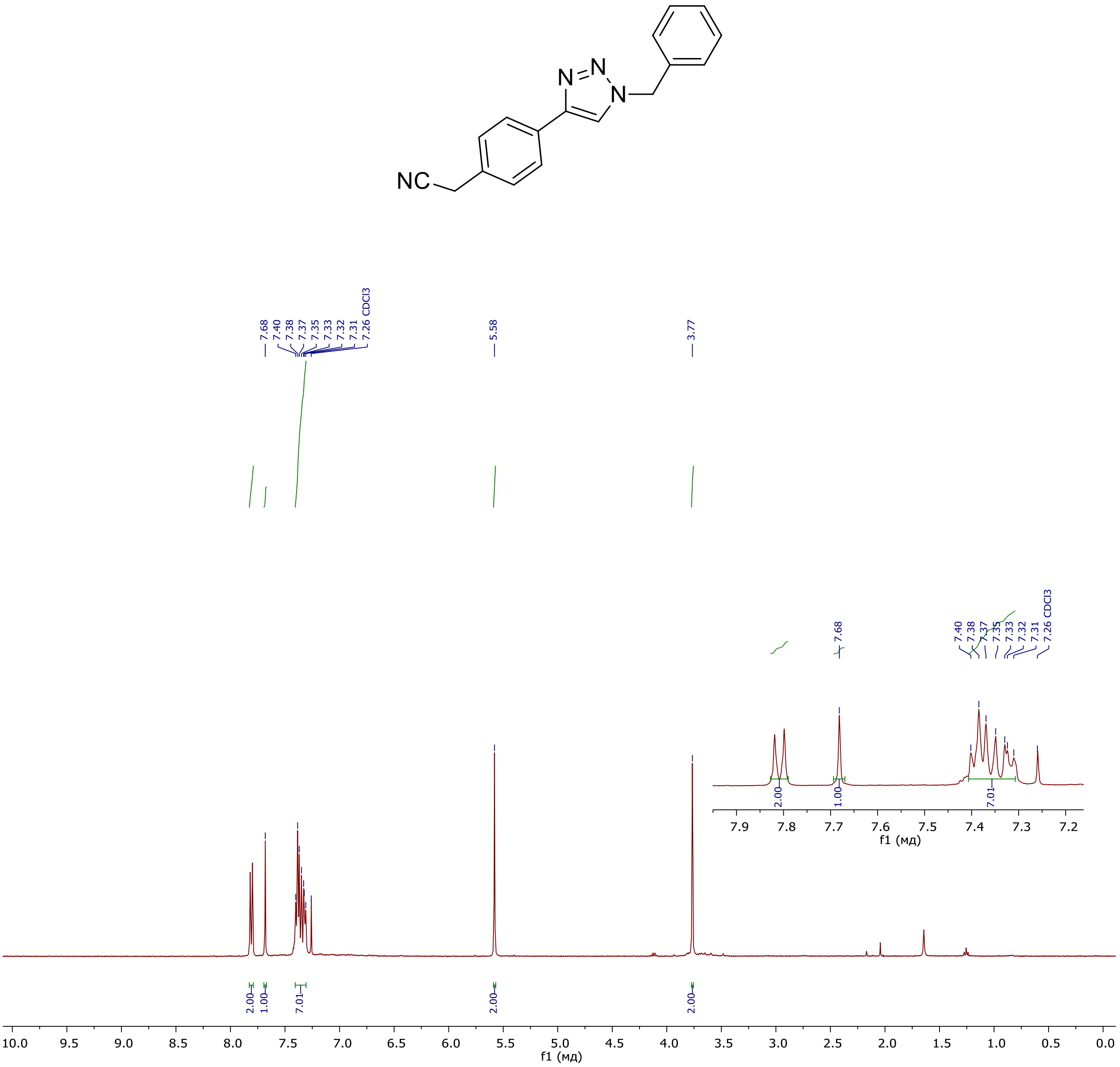

Figure S11. ^{1}H NMR (400 MHz, Chloroform-*d*) spectrum of 2-(4-(1-benzyl-1H-1,2,3-triazol-4-yl)phenyl)acetonitrile.

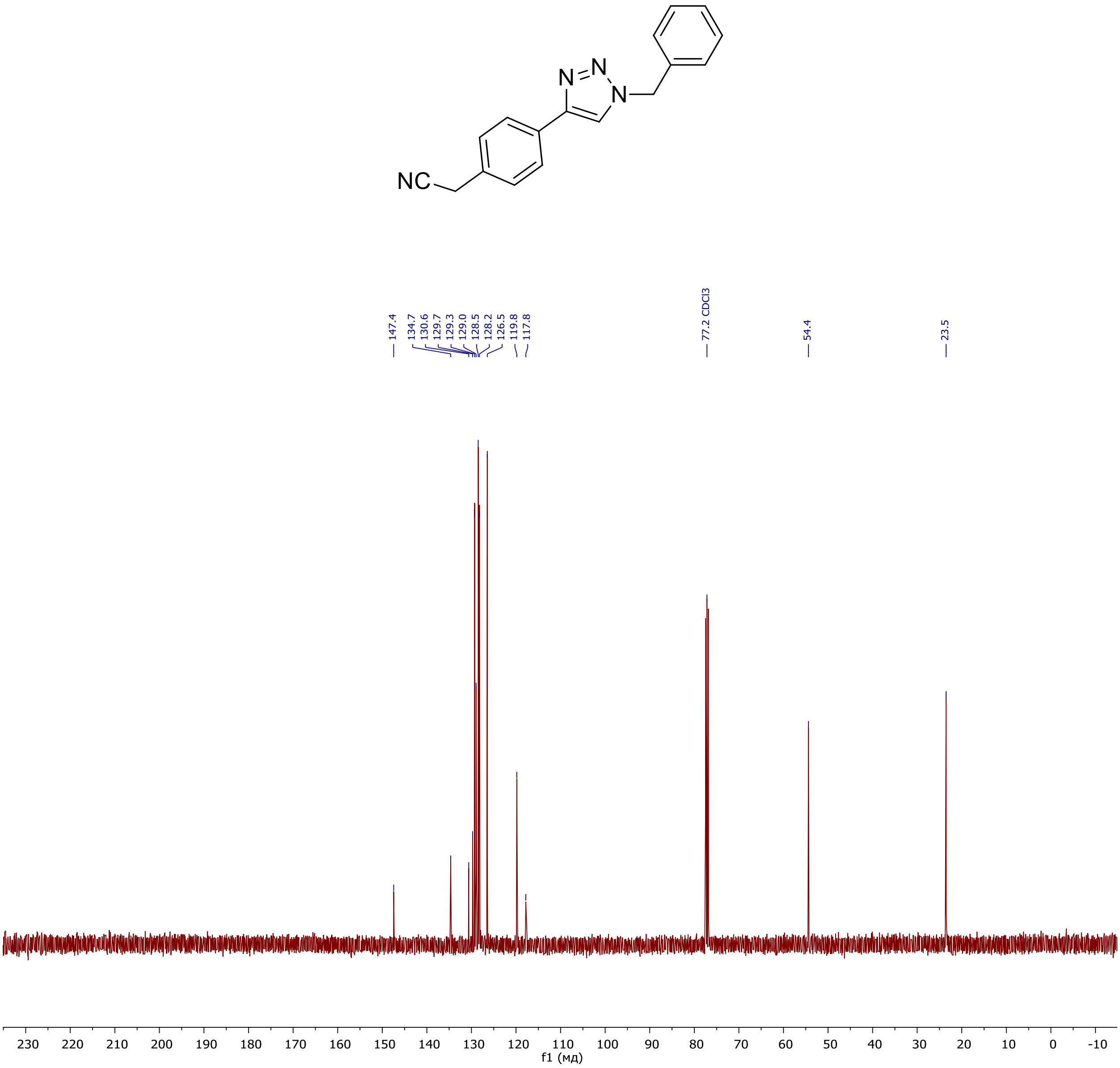

Figure S12. ^{13}C NMR (101 MHz, Chloroform-*d*) spectrum of 2-(4-(1-benzyl-1H-1,2,3-triazol-4-yl)phenyl)acetonitrile.

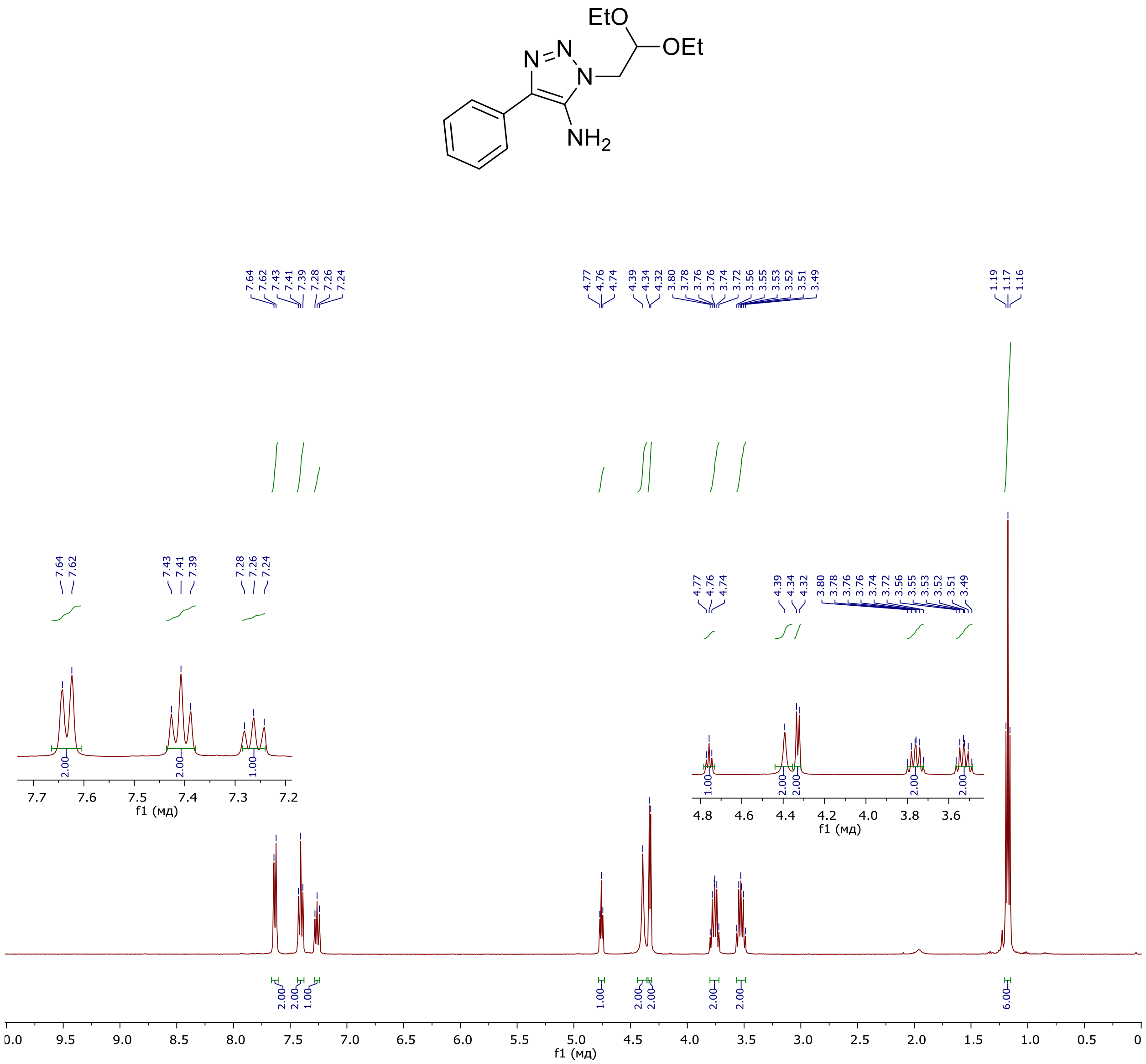

Figure S13. ^{1}H NMR (400 MHz, Chloroform-*d*) spectrum of compound **3a**.

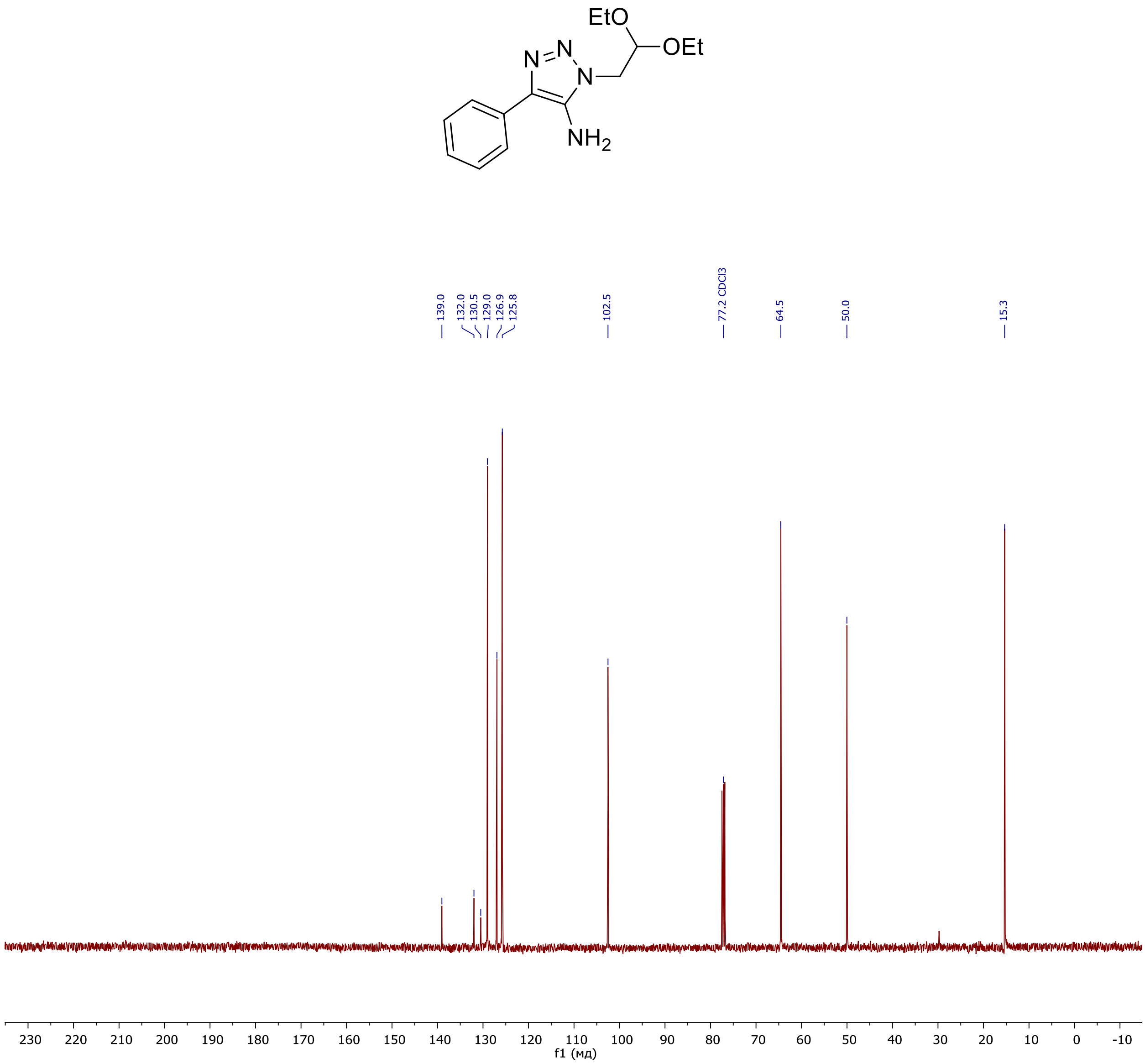

Figure S14. ^{13}C NMR (101 MHz, Chloroform-*d*) spectrum of compound **3a**.

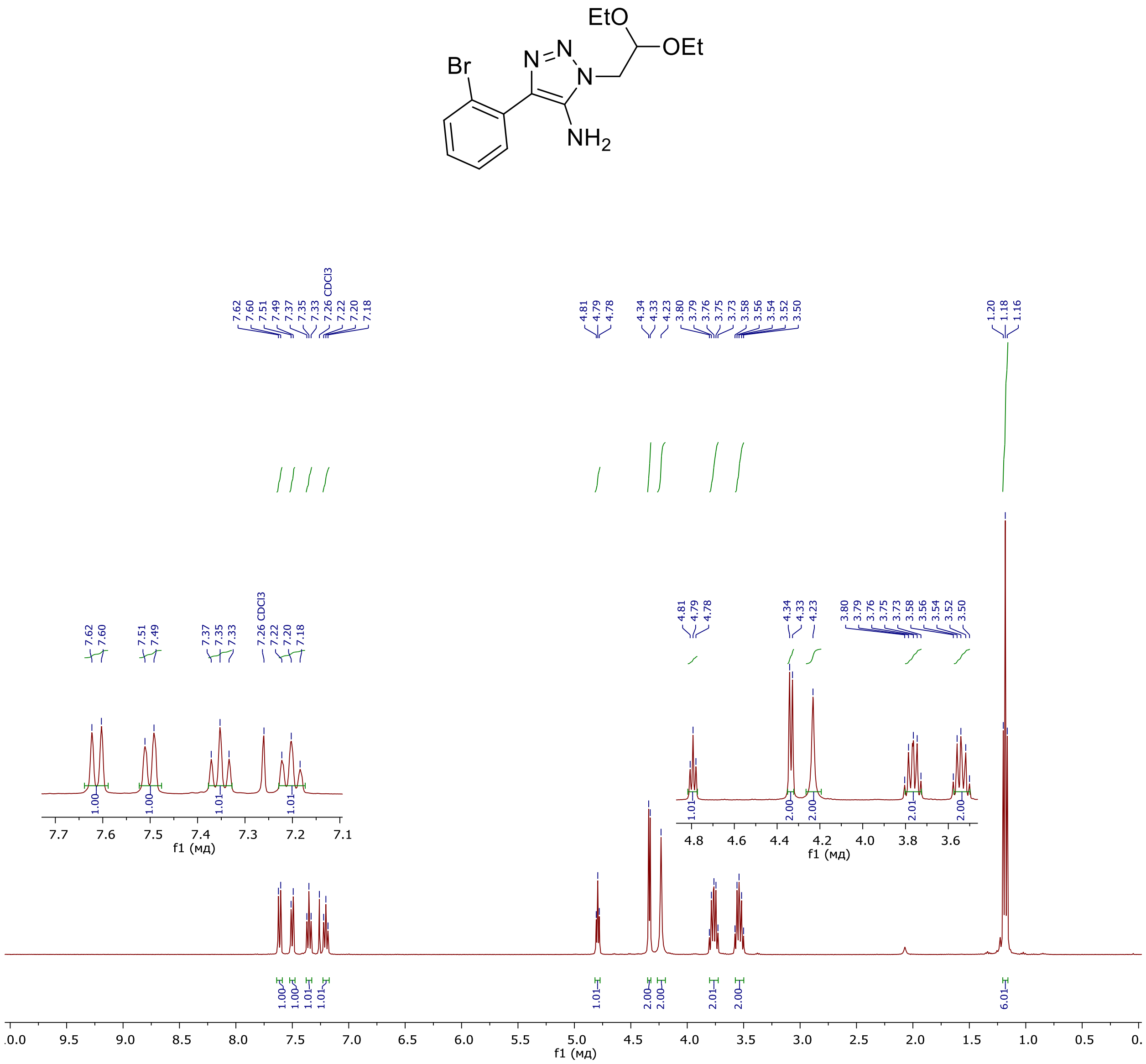

Figure S15. ^{1}H NMR (400 MHz, Chloroform-*d*) spectrum of compound **3b**.

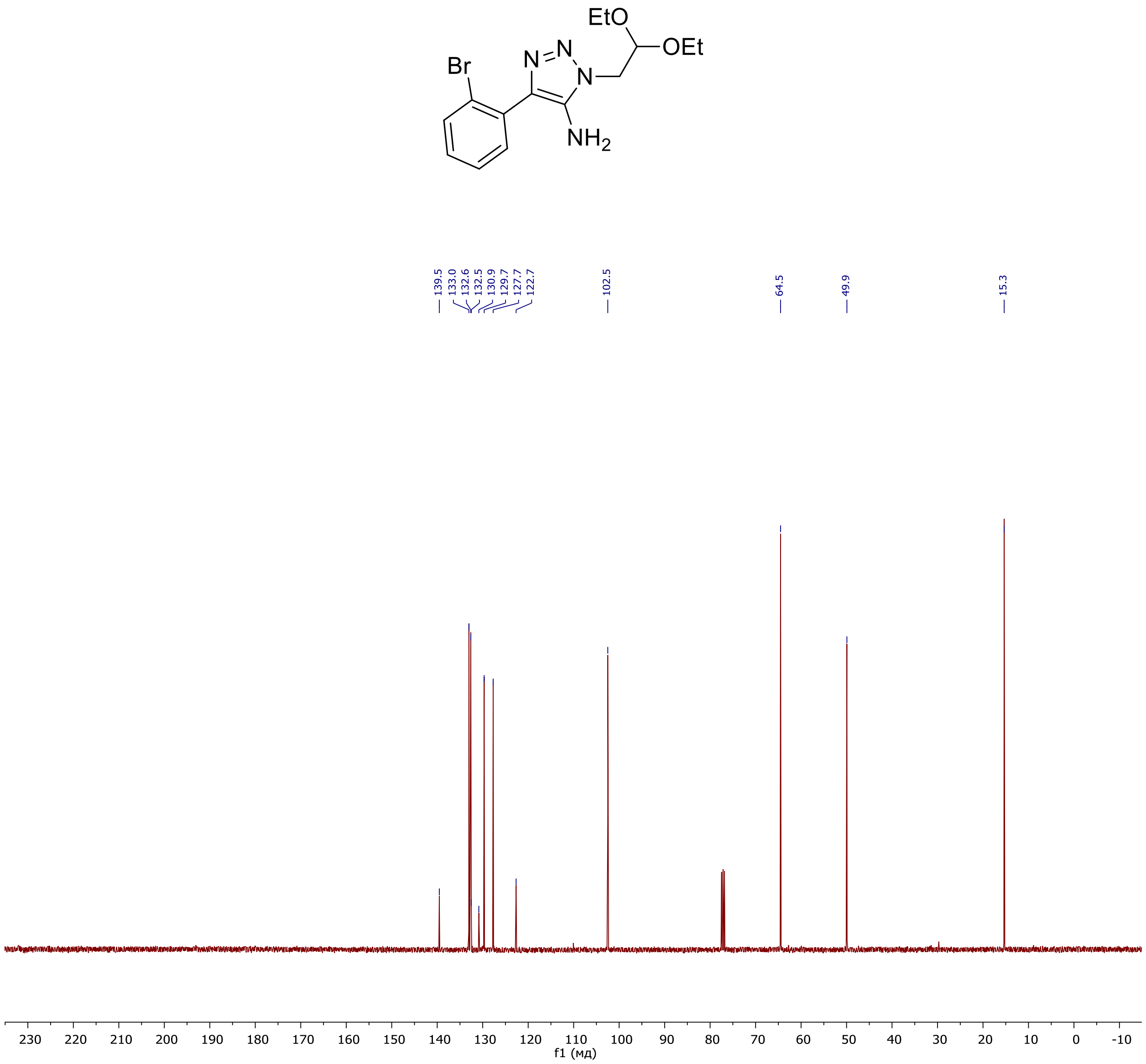

Figure S16. ^{13}C NMR (101 MHz, Chloroform-*d*) spectrum of compound **3b**.

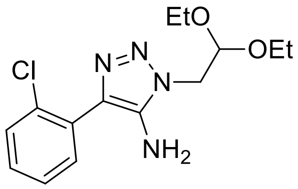

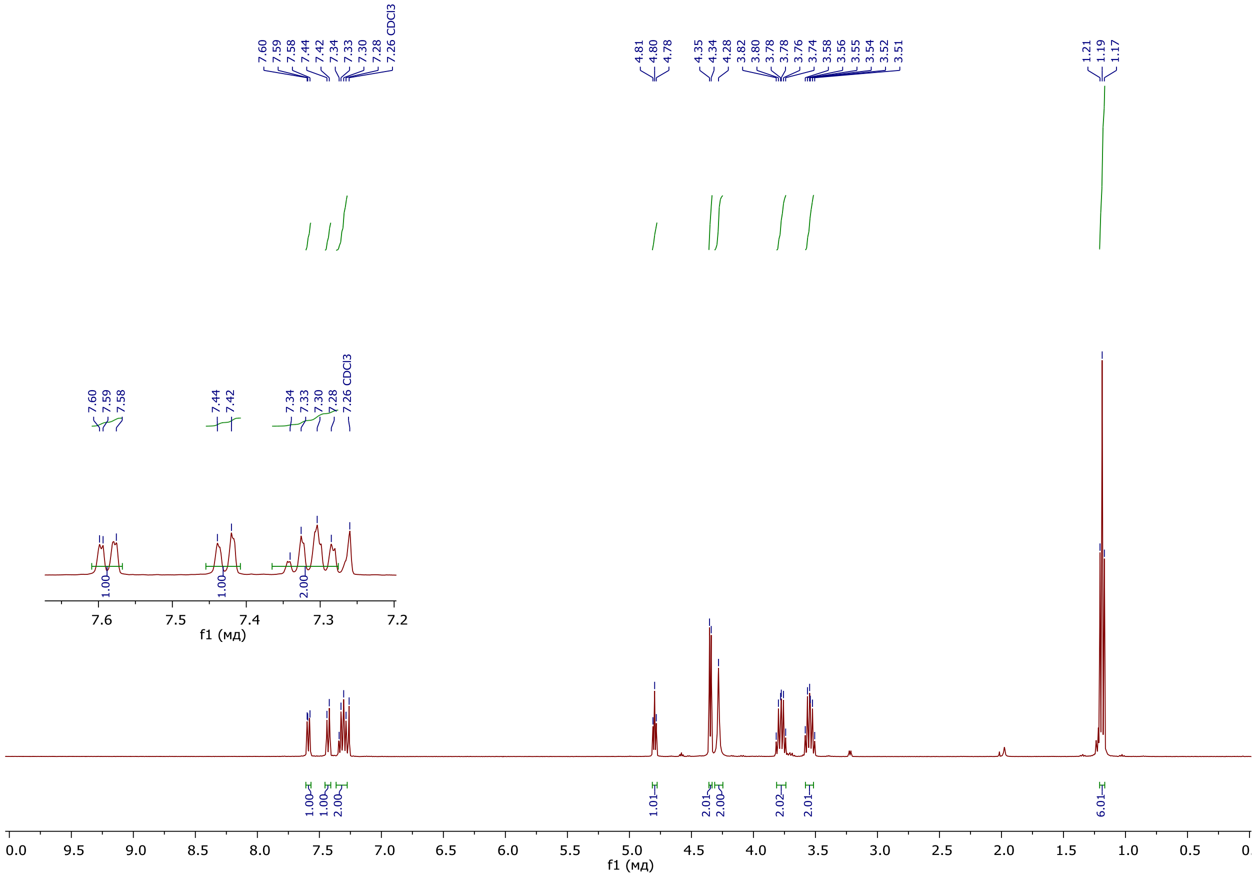

Figure S17. ^{1}H NMR (400 MHz, Chloroform-*d*) spectrum of compound **3c**.

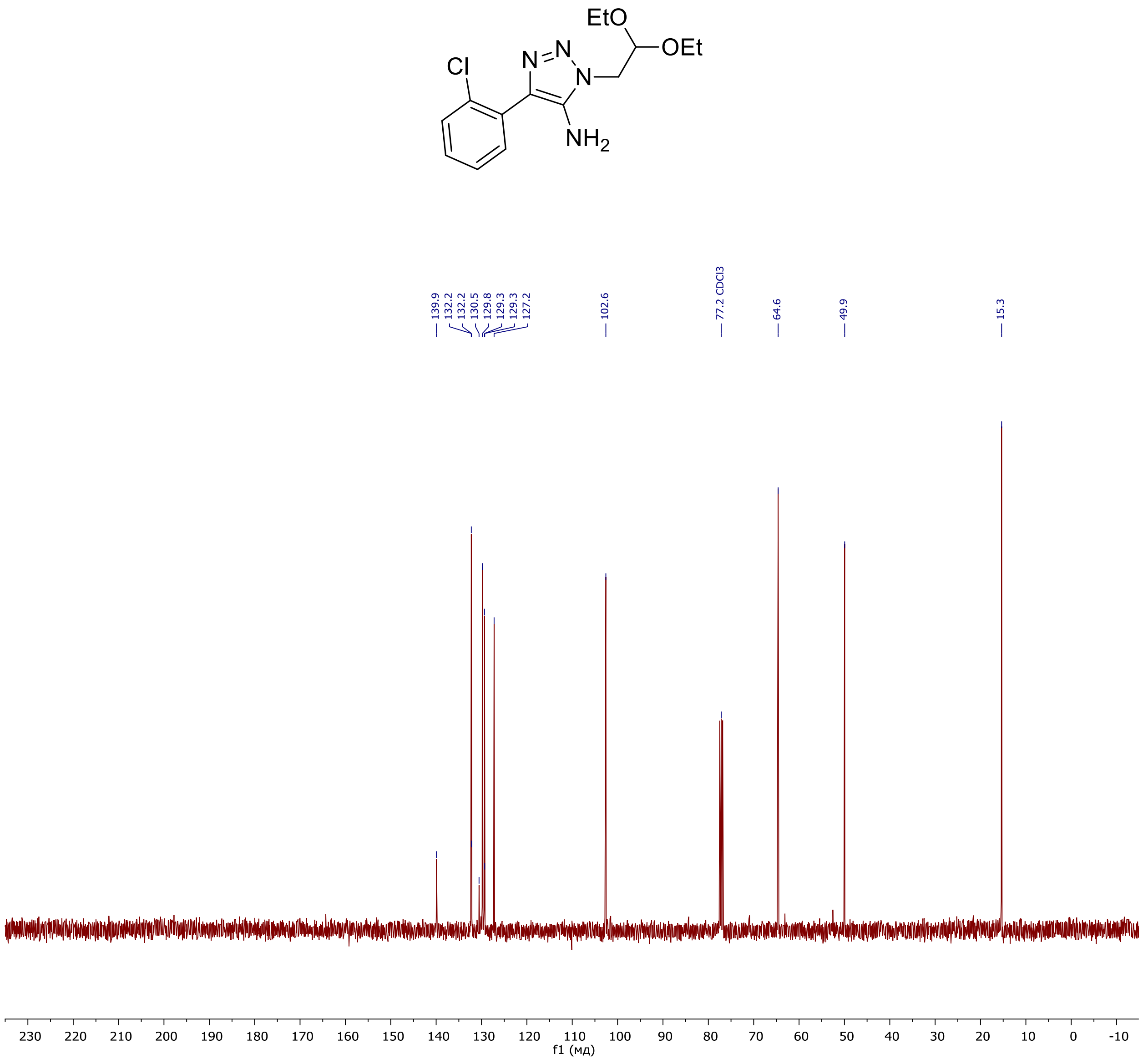

Figure S18. ^{13}C NMR (101 MHz, Chloroform-*d*) spectrum of compound **3c**.

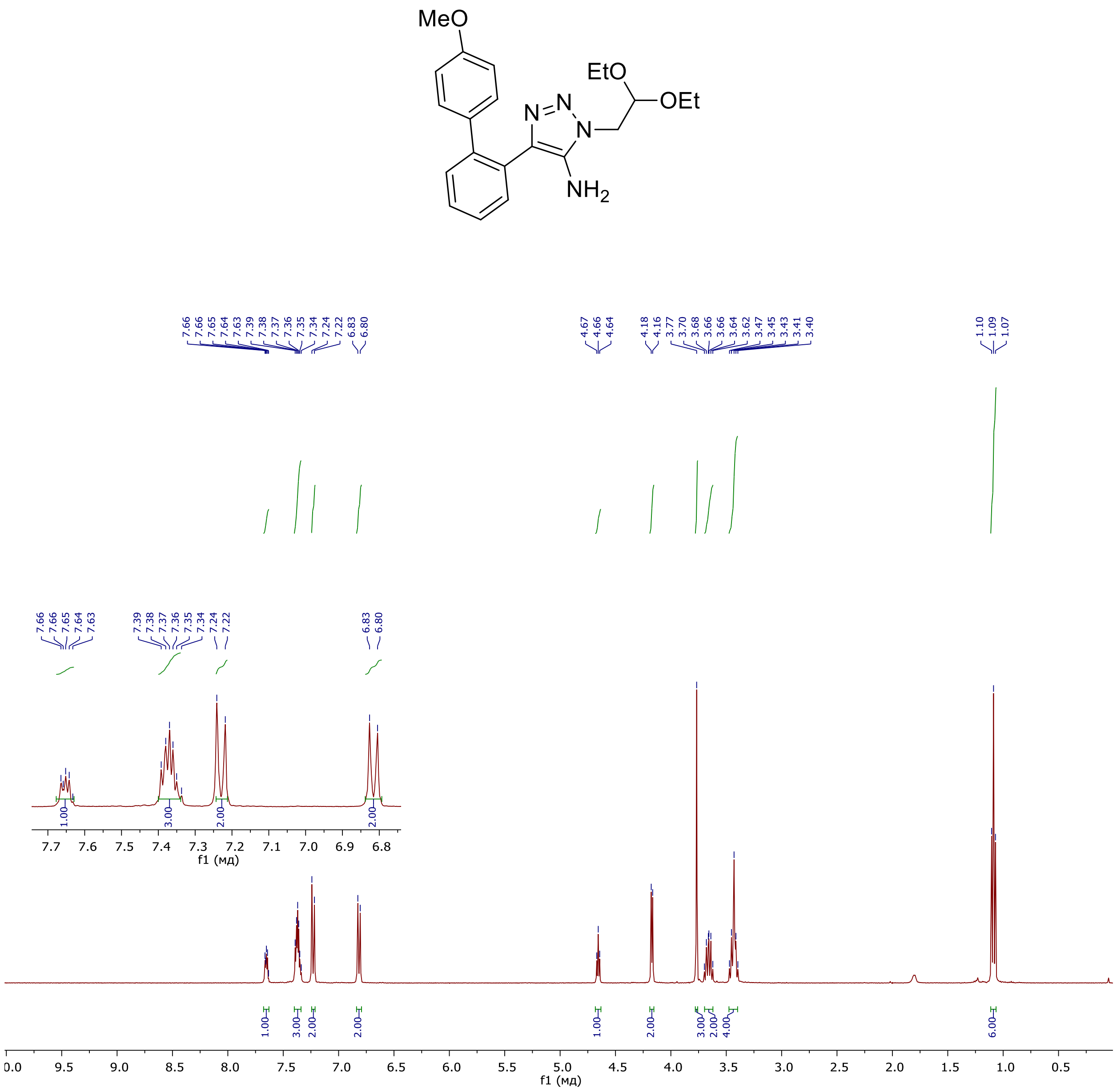

Figure 19 ^{1}H NMR (400 MHz, Chloroform-*d*) spectrum of compound **3d**.

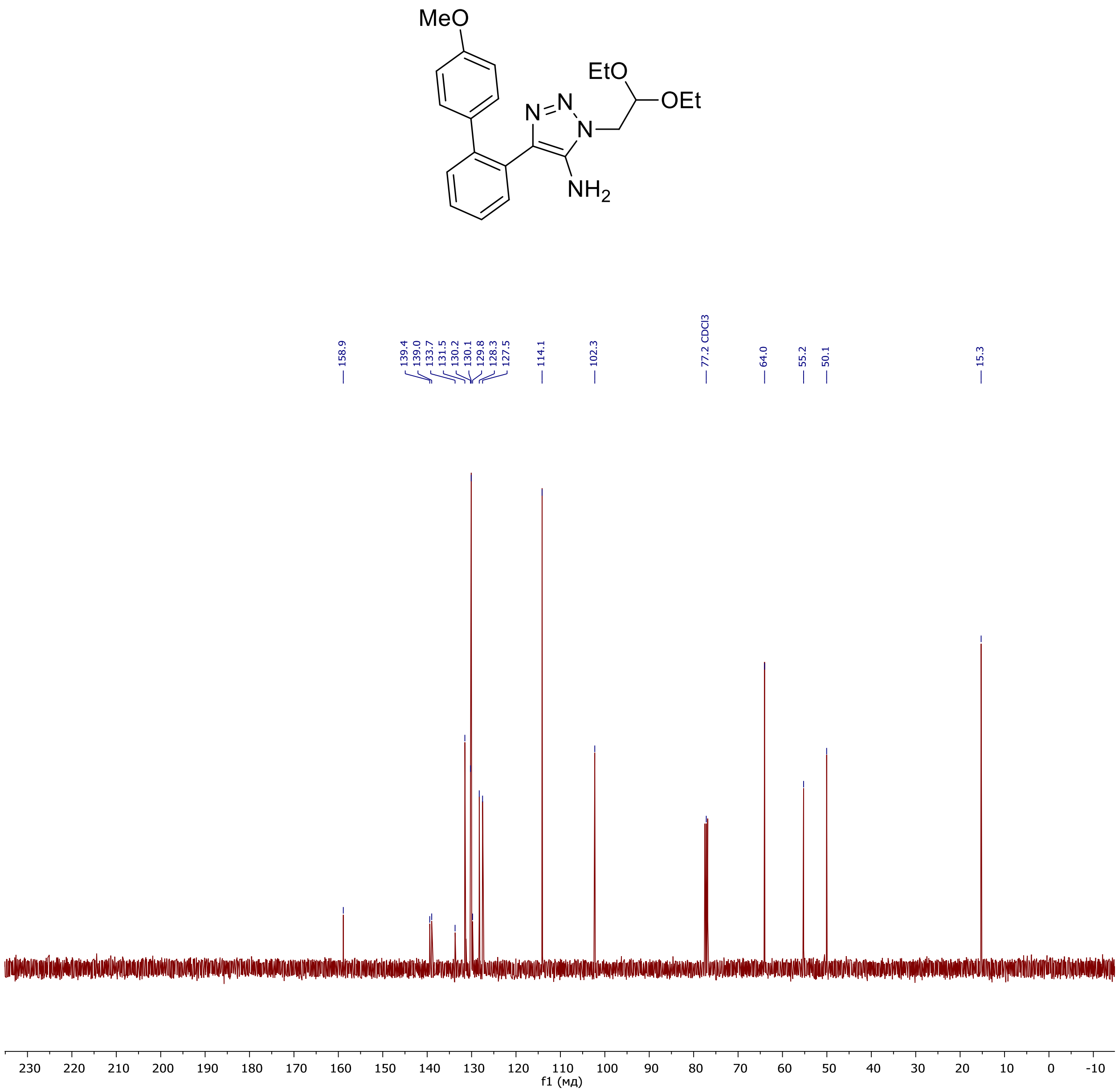

Figure S20. ^{13}C NMR (101 MHz, Chloroform-*d*) spectrum of compound **3d**.

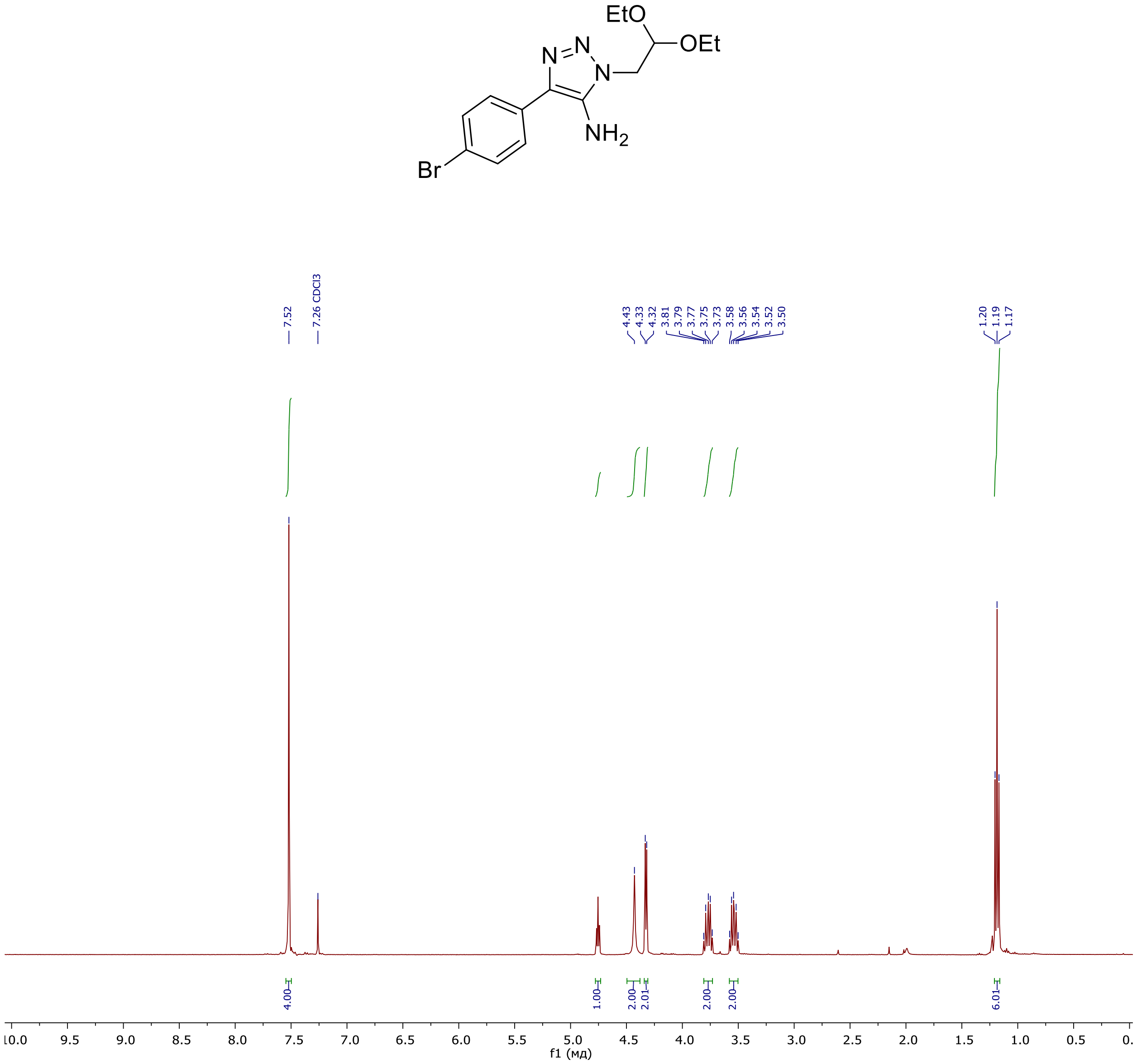

Figure S21. ^{1}H NMR (400 MHz, Chloroform-*d*) spectrum of compound **3e**.

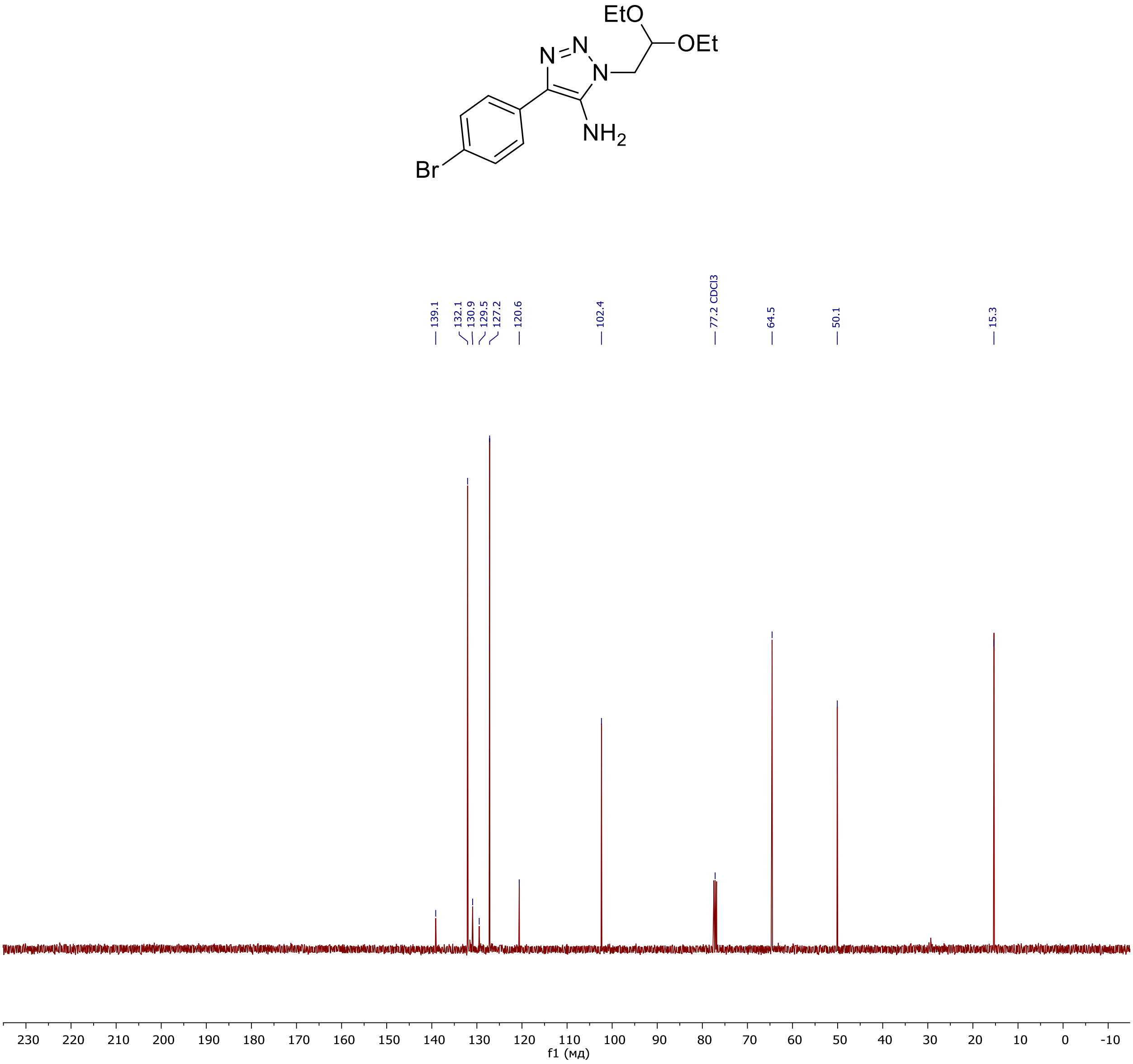

Figure S22. ^{13}C NMR (101 MHz, Chloroform-*d*) spectrum of compound **3e**.

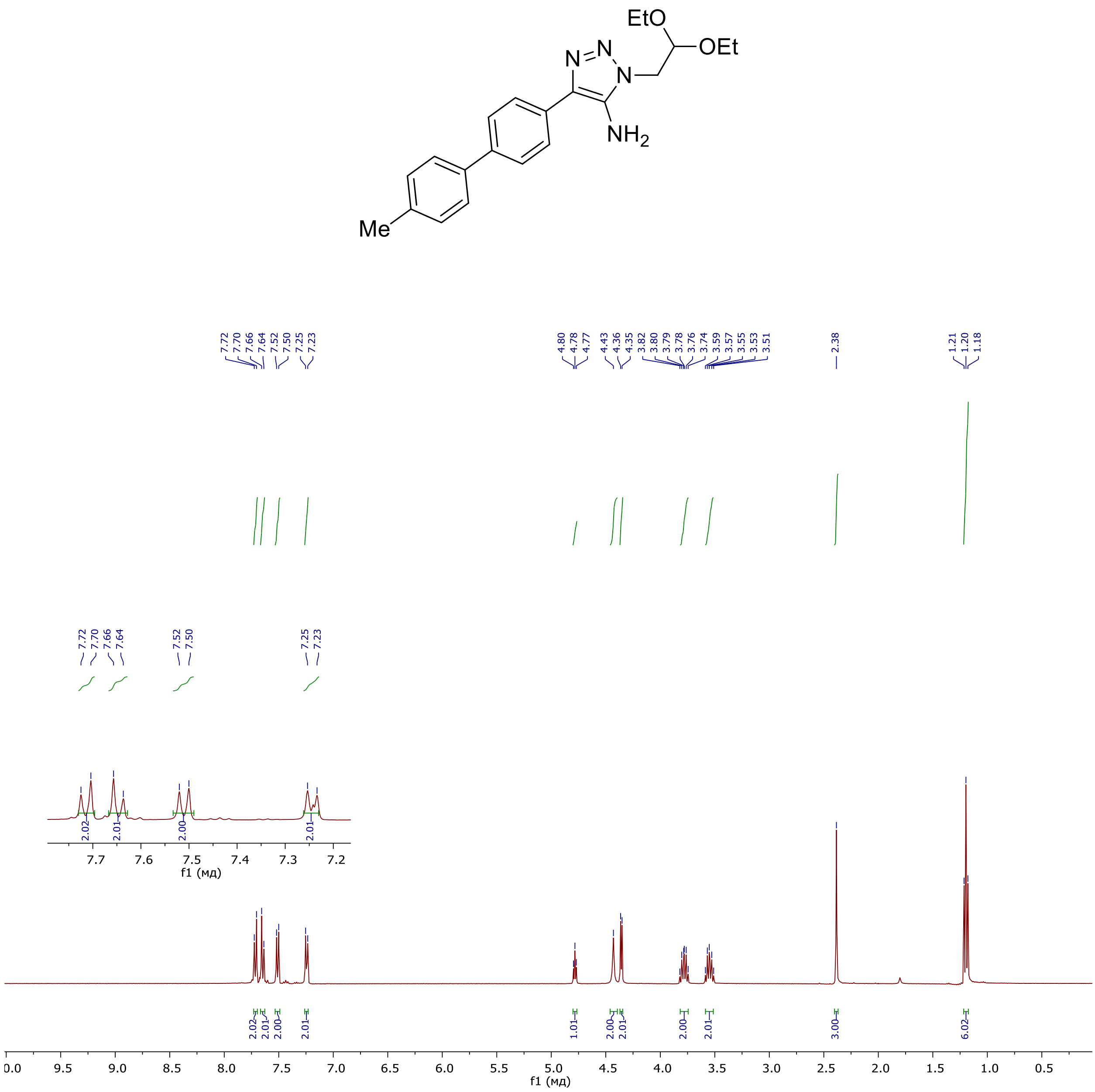

Figure S23. ^{1}H NMR (400 MHz, Chloroform-*d*) spectrum of compound **3f**.

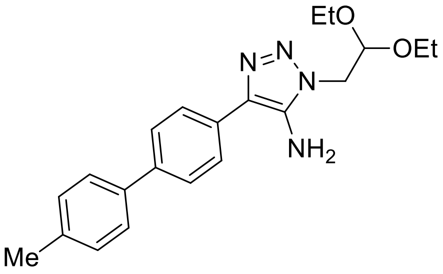

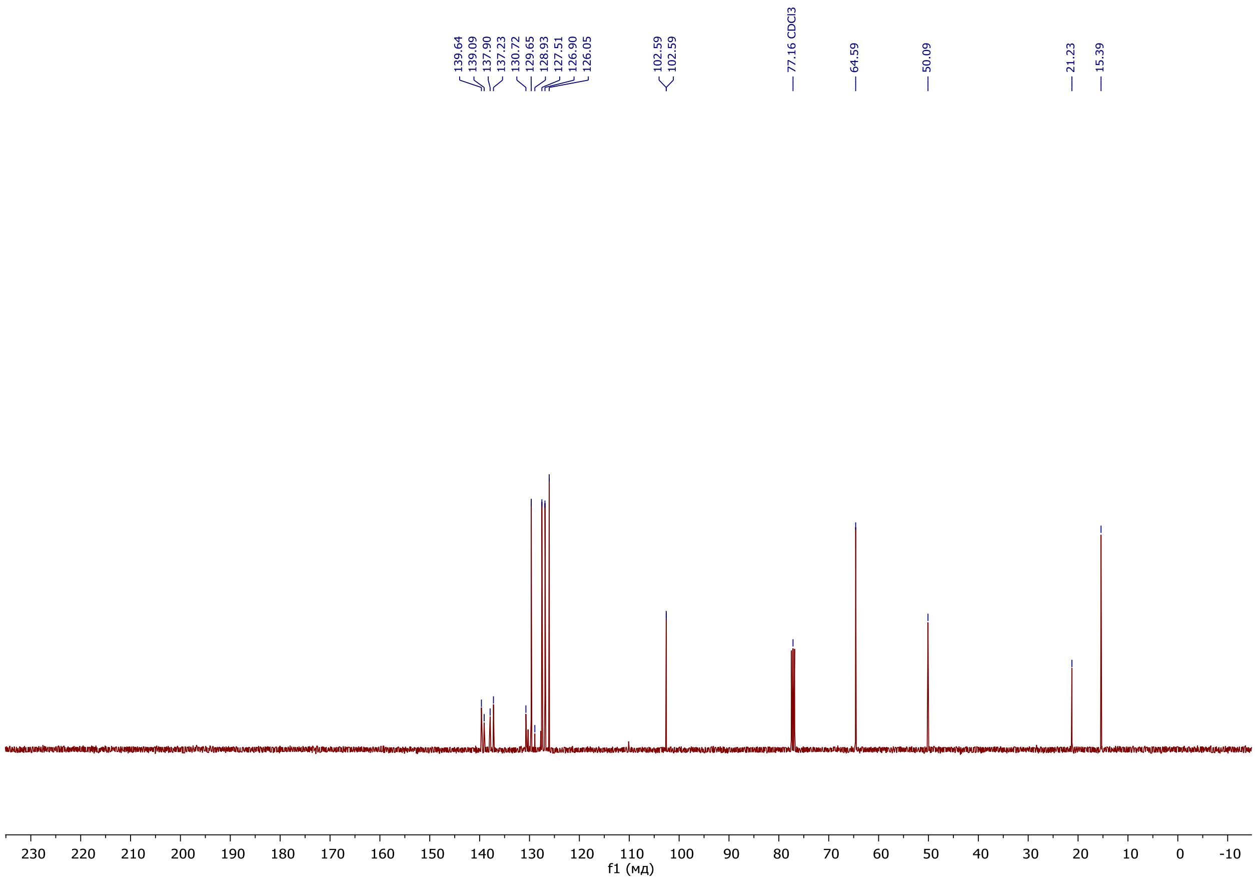

Figure S24. ^{13}C NMR (101 MHz, Chloroform-*d*) spectrum of compound **3f**.

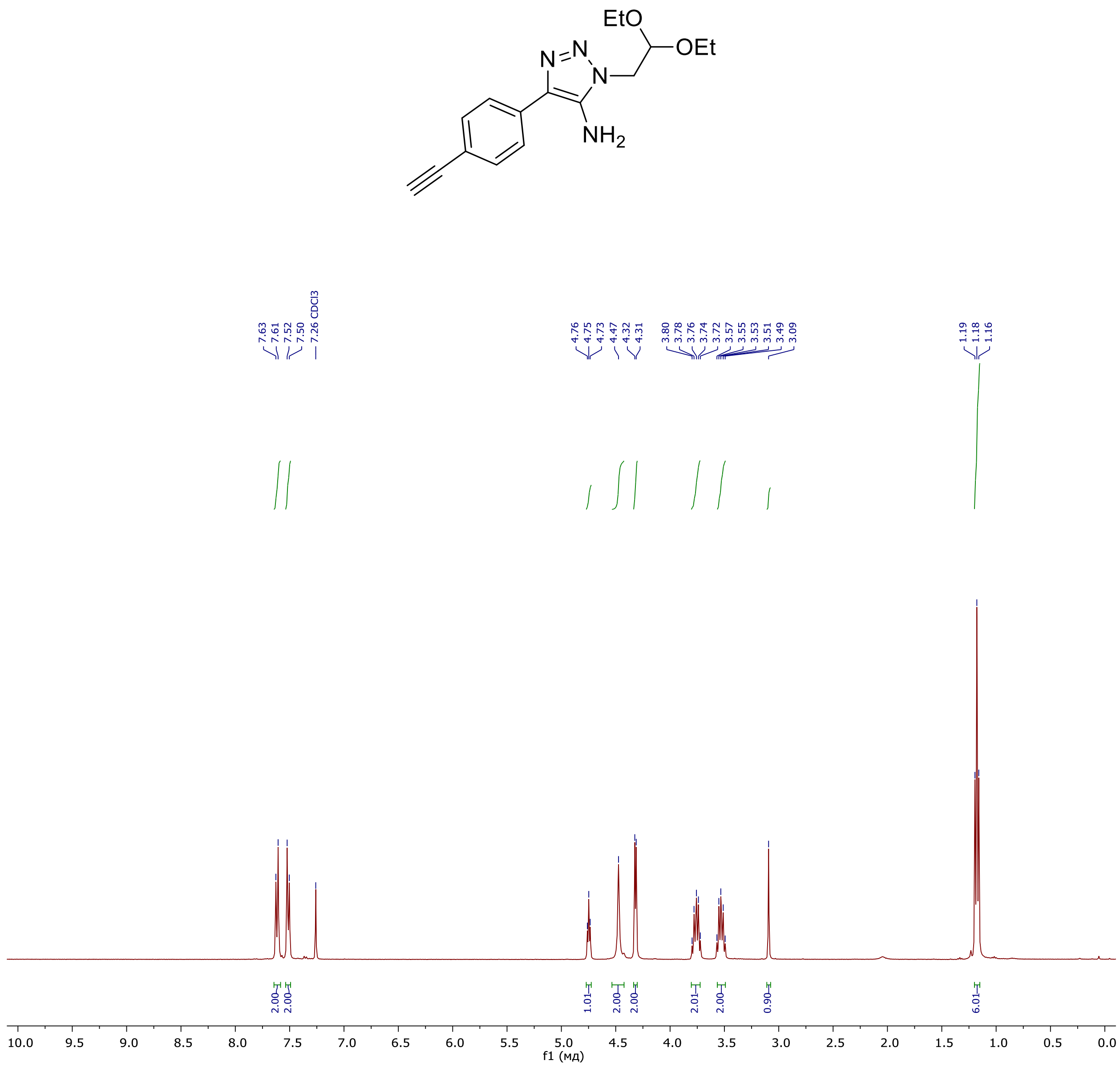

Figure 25 ^{1}H NMR (400 MHz, Chloroform-*d*) spectrum of compound **3g**.

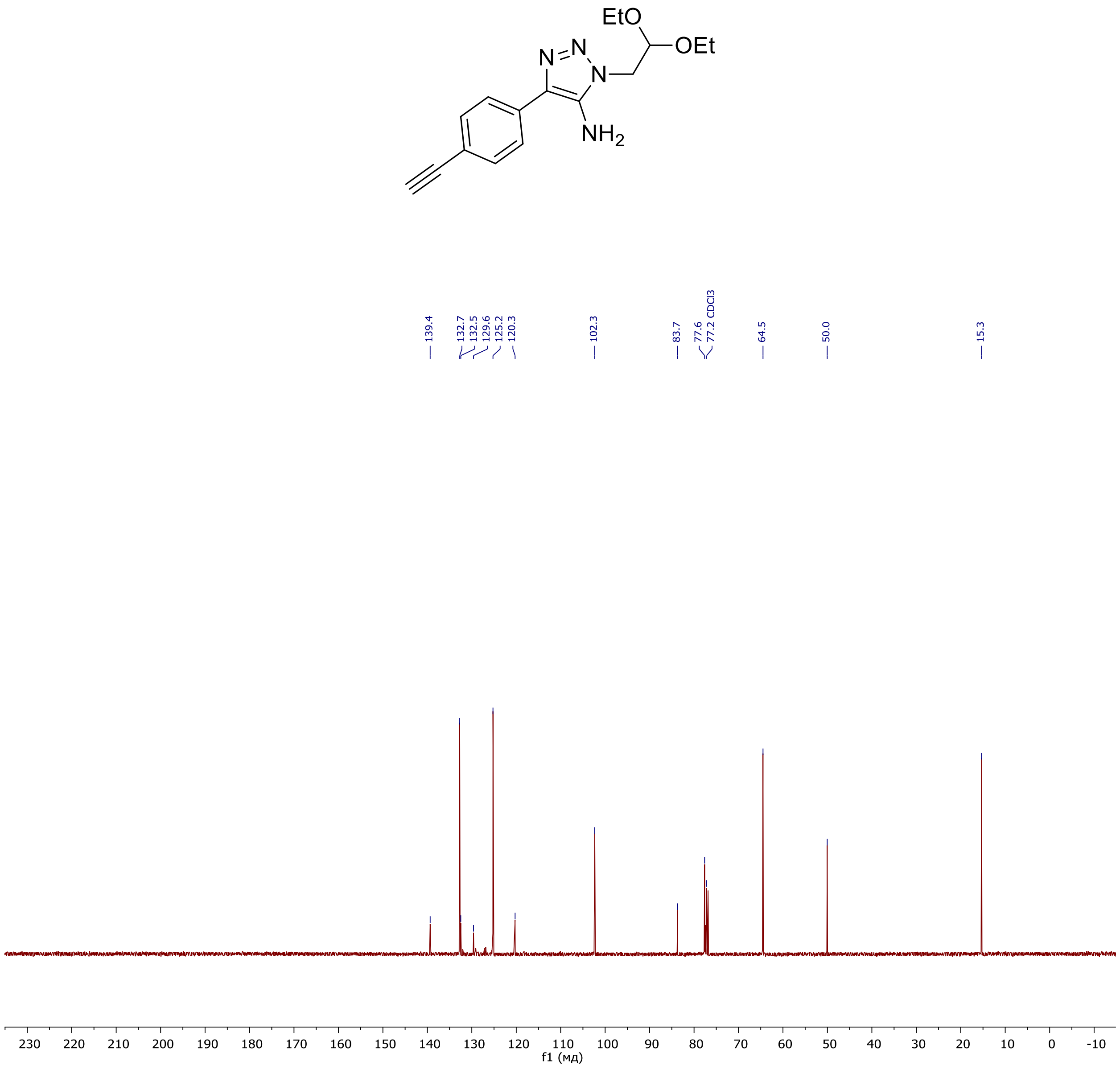

Figure S26. ^{13}C NMR (101 MHz, Chloroform-*d*) spectrum of compound **3g**.

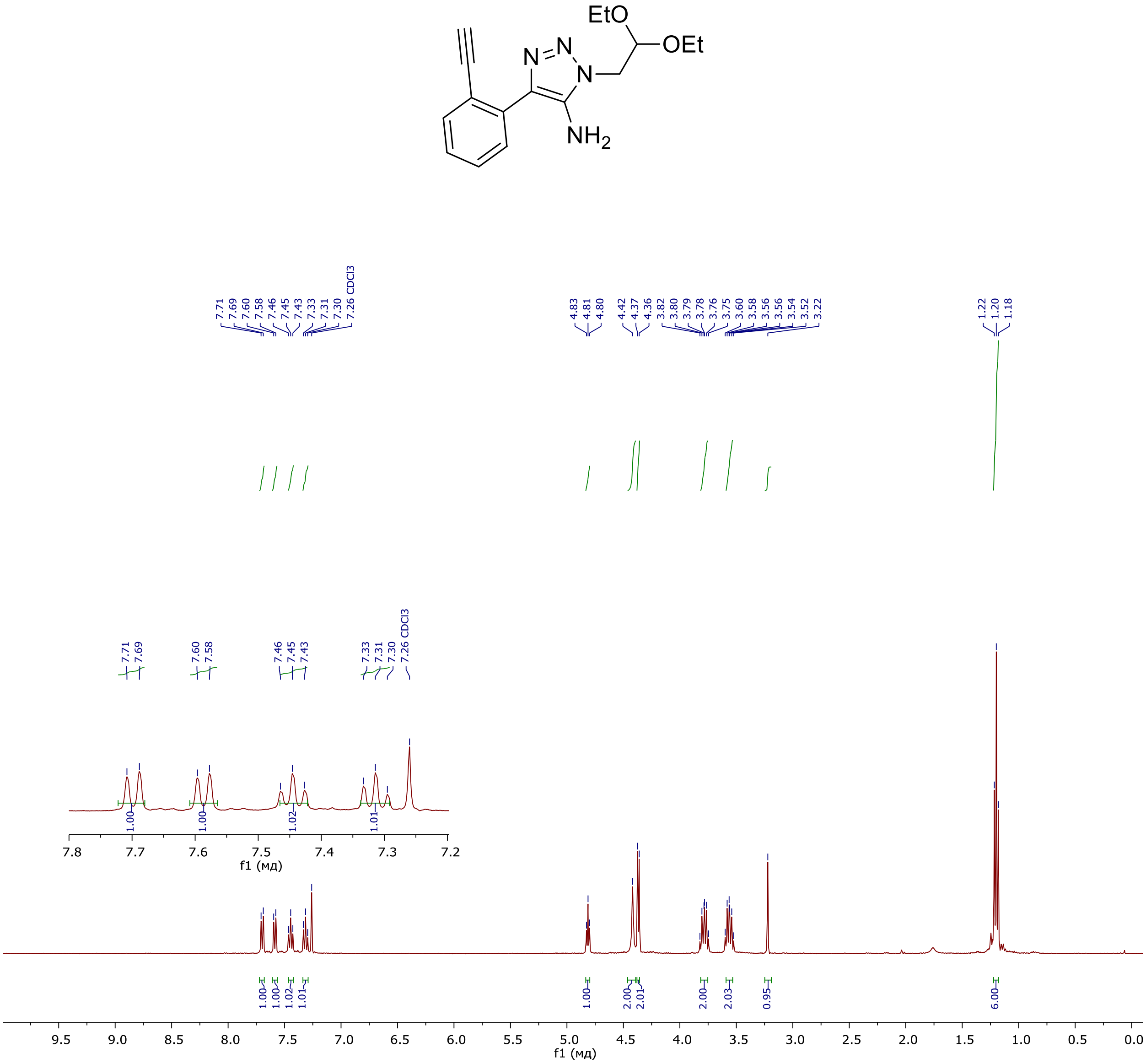

Figure 27 ^{1}H NMR (400 MHz, Chloroform-*d*) spectrum of compound **3h**.

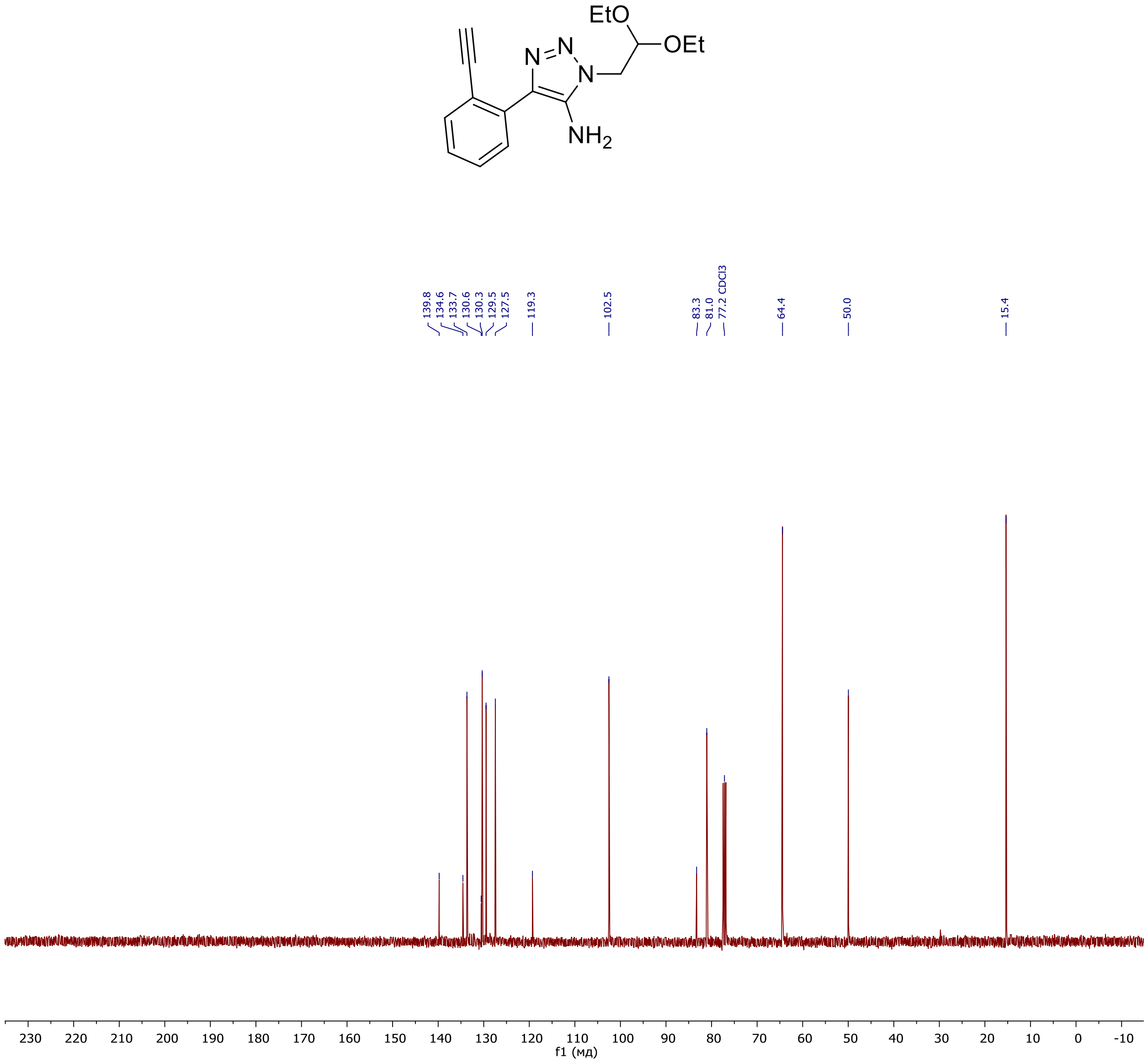

Figure S28. ^{13}C NMR (101 MHz, Chloroform-*d*) spectrum of compound **3h**.

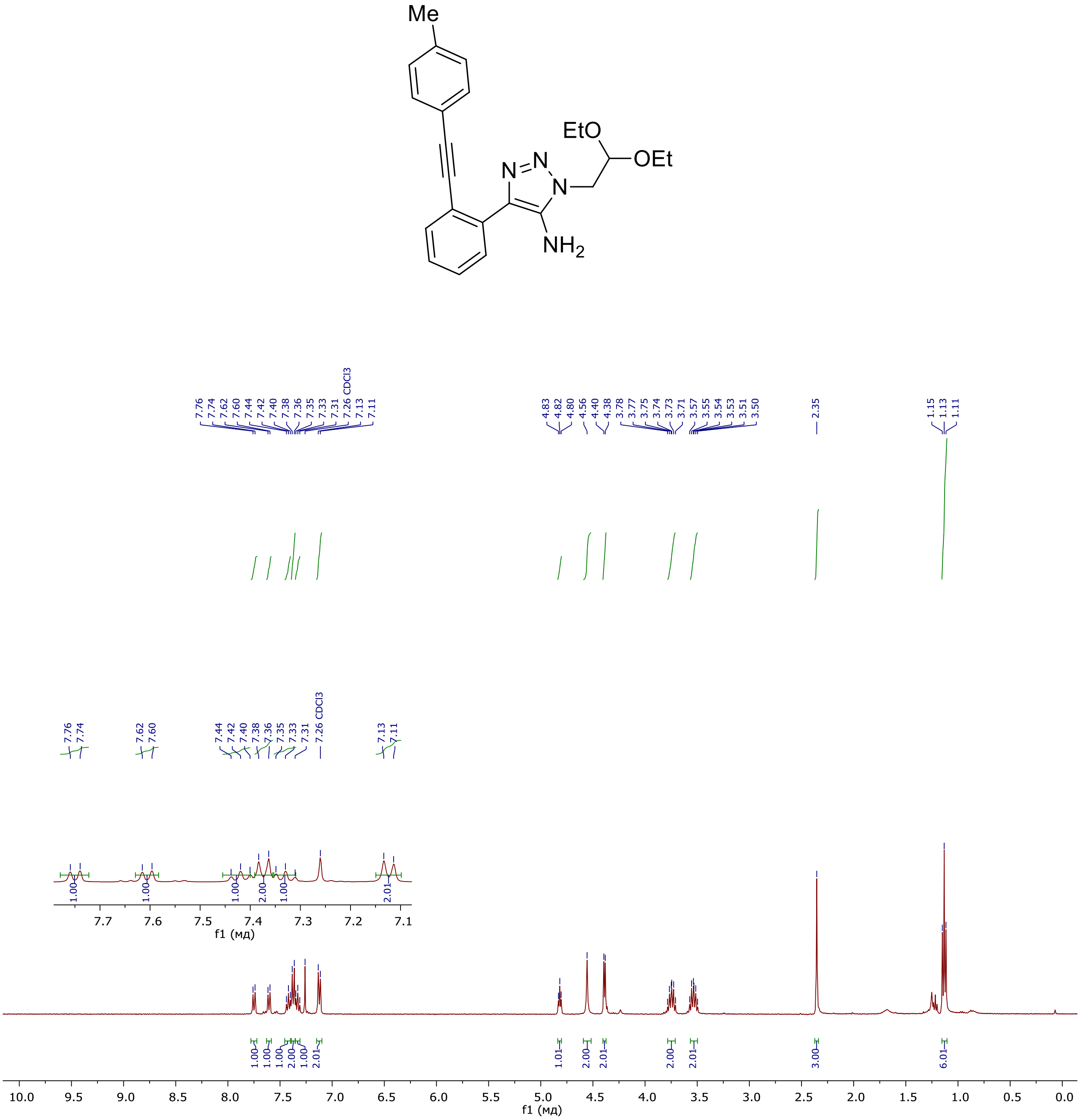

Figure 29 ^{1}H NMR (400 MHz, Chloroform-*d*) spectrum of compound **3i**.

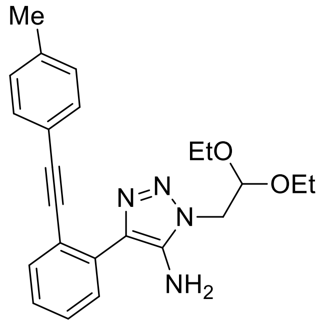

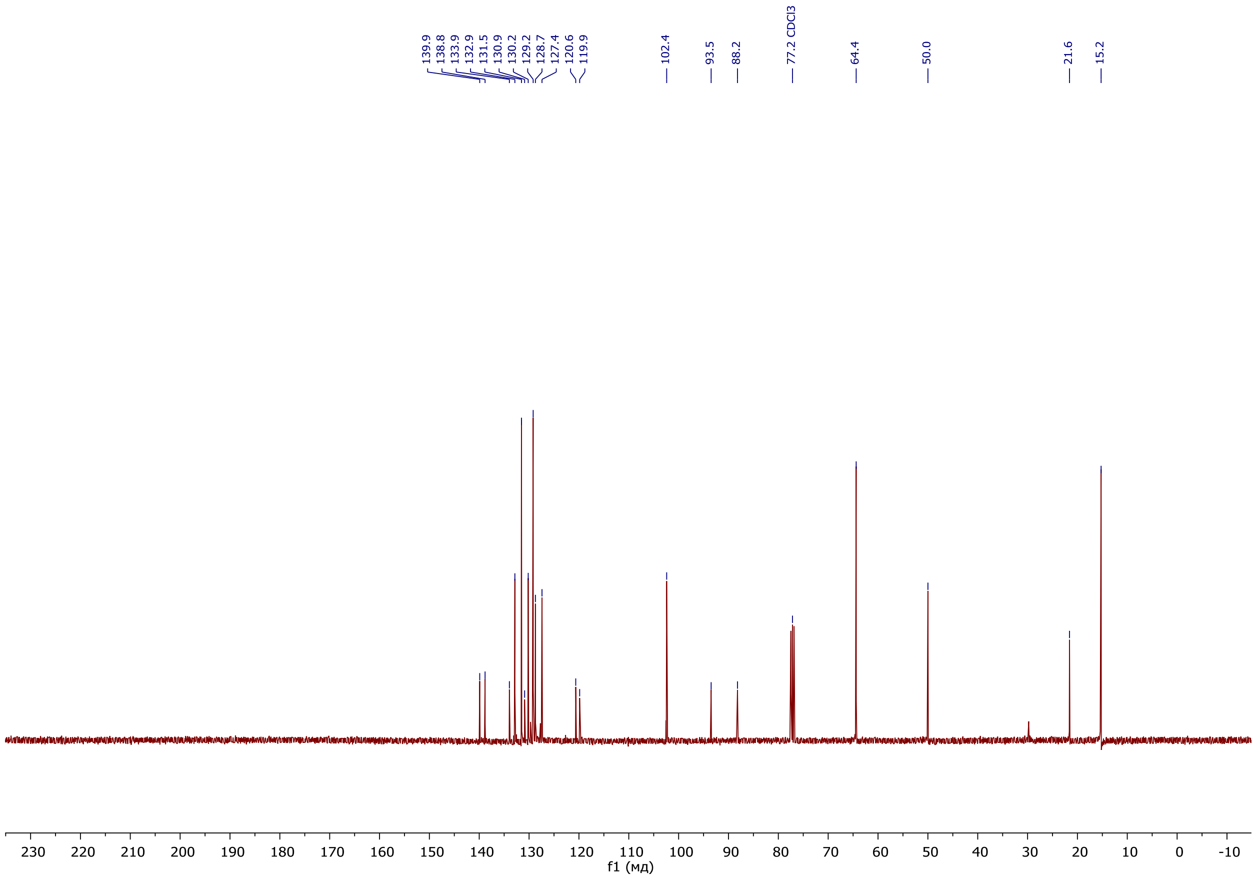

Figure S30. ^{13}C NMR (101 MHz, Chloroform-*d*) spectrum of compound **3i**.

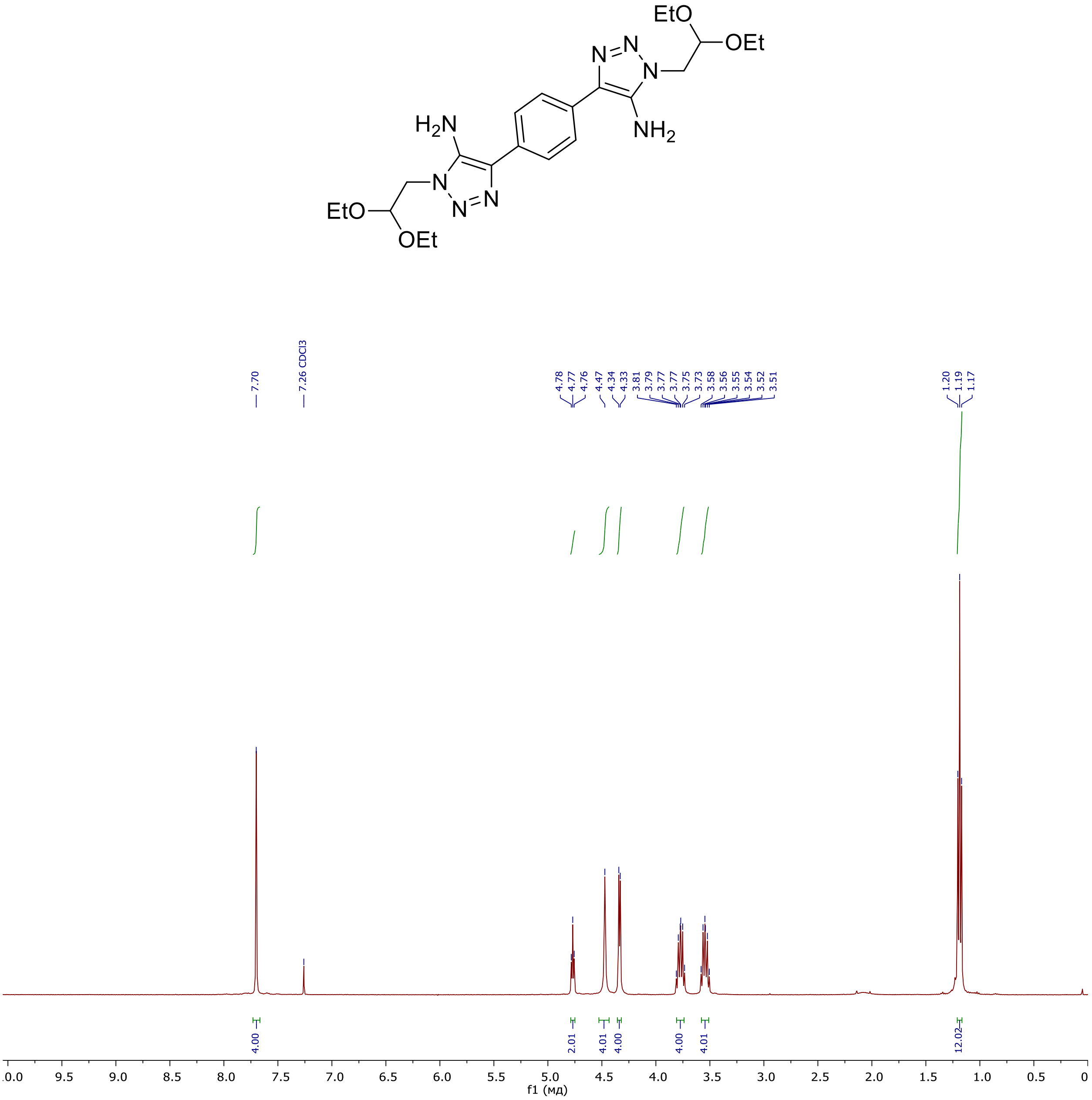

Figure 31 ^{1}H NMR (400 MHz, Chloroform-*d*) spectrum of compound **3j**.

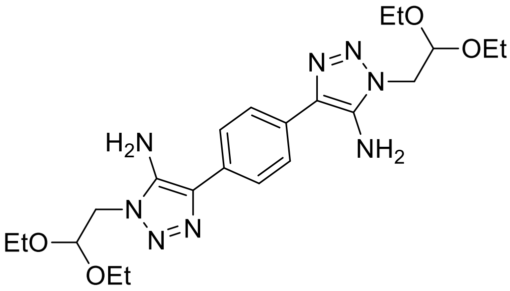

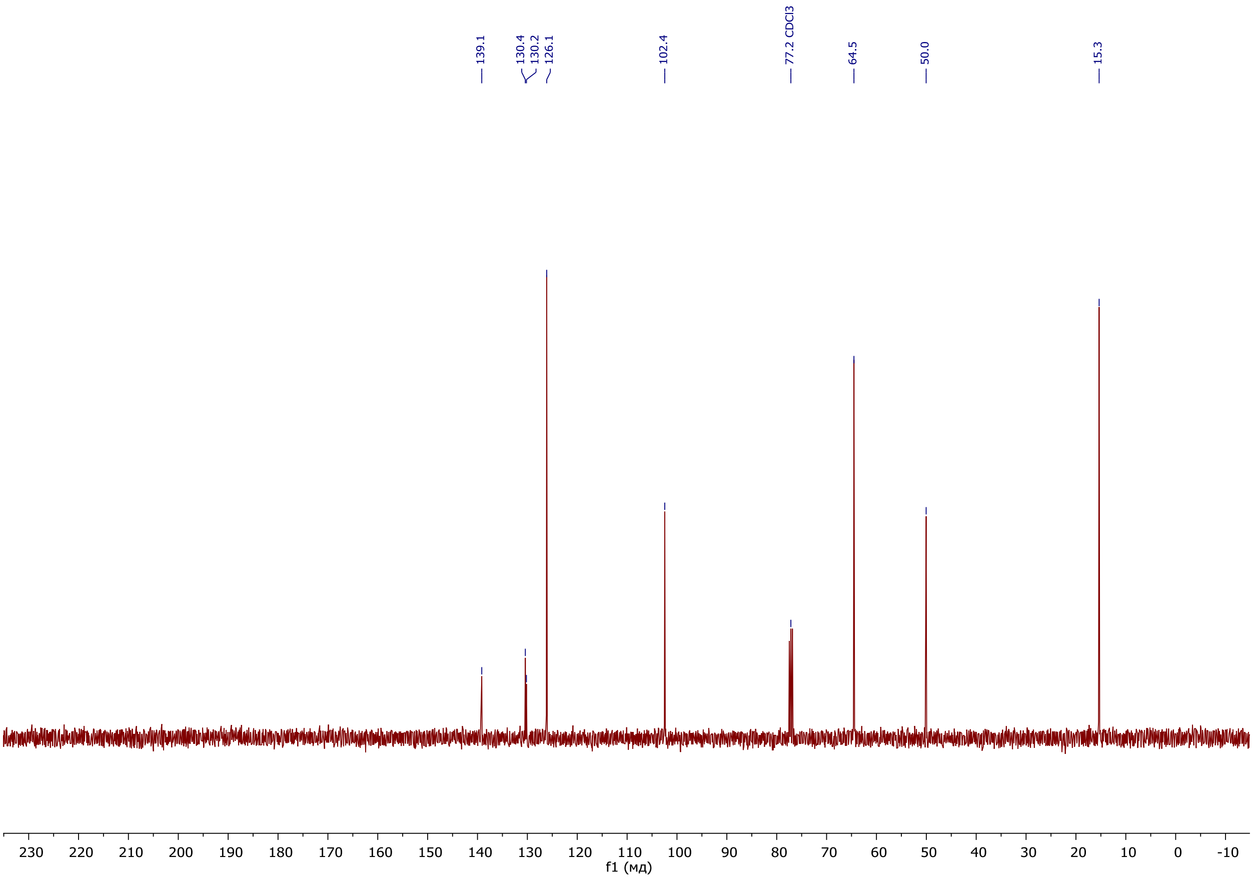

Figure S32. ^{13}C NMR (101 MHz, Chloroform-*d*) spectrum of compound **3j**.

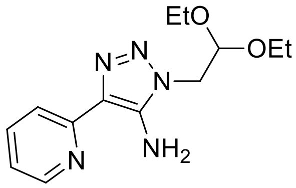

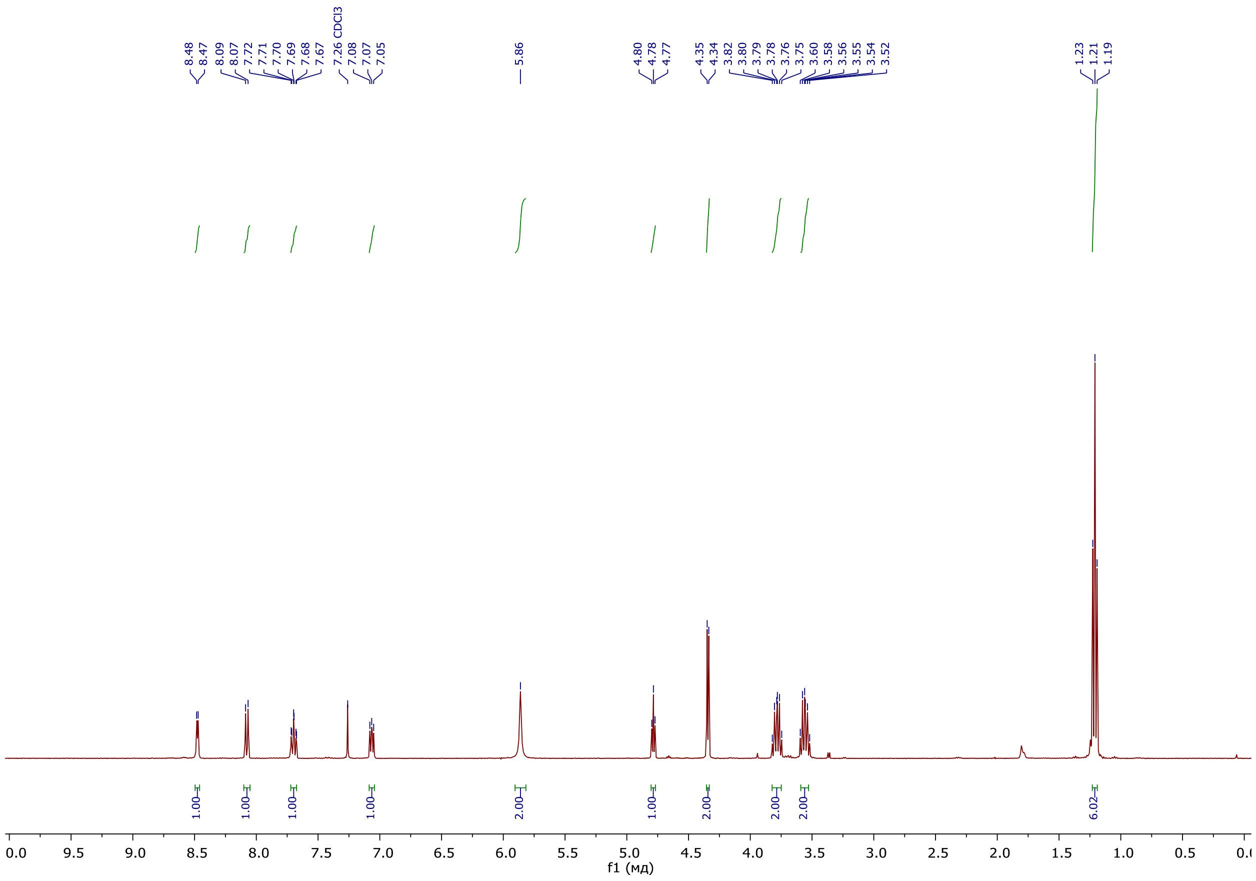

Figure S33 ^{1}H NMR (400 MHz, Chloroform-*d*) spectrum of compound **3k**.

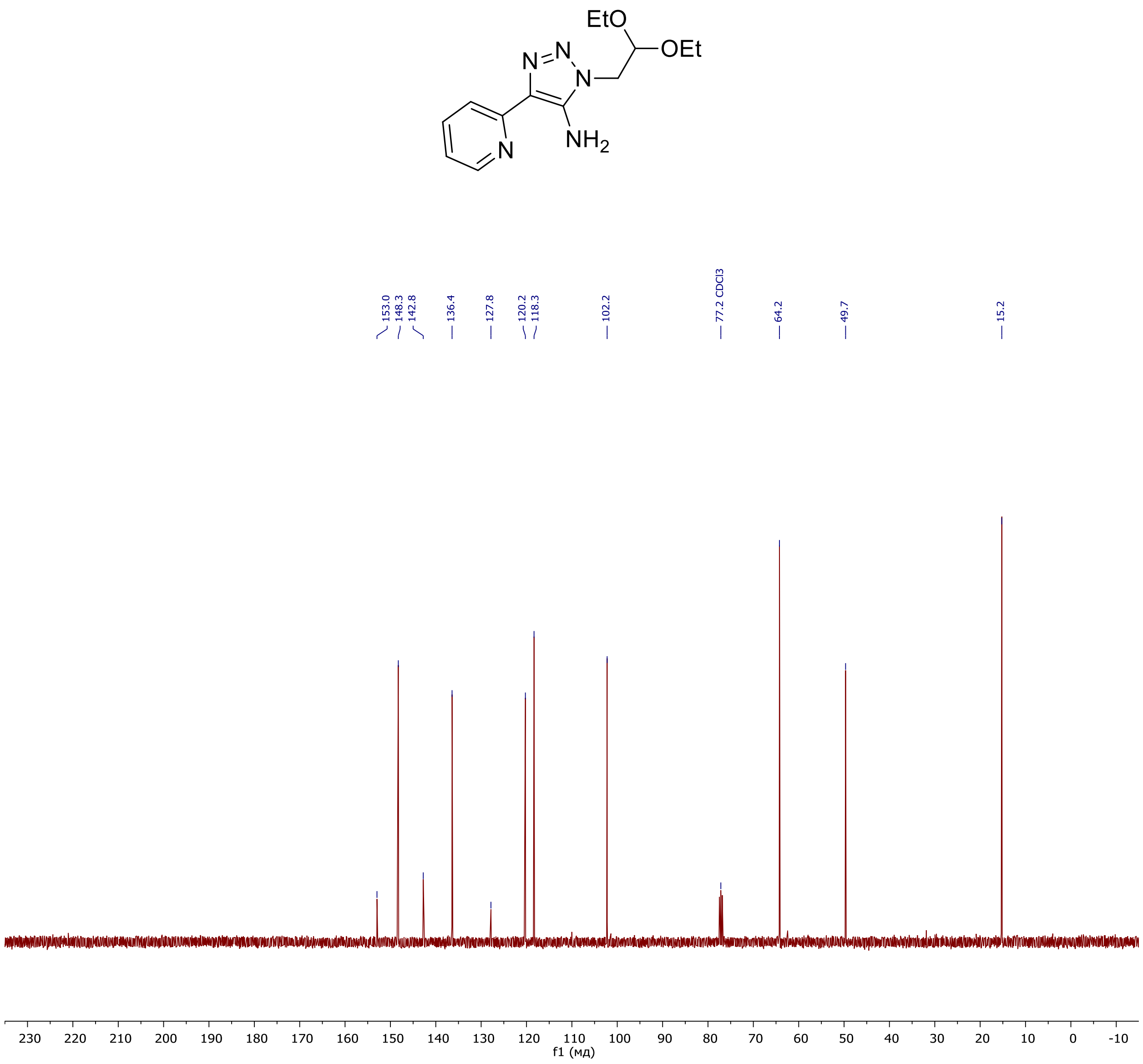

Figure S34. ^{13}C NMR (101 MHz, Chloroform-*d*) spectrum of compound **3k**.

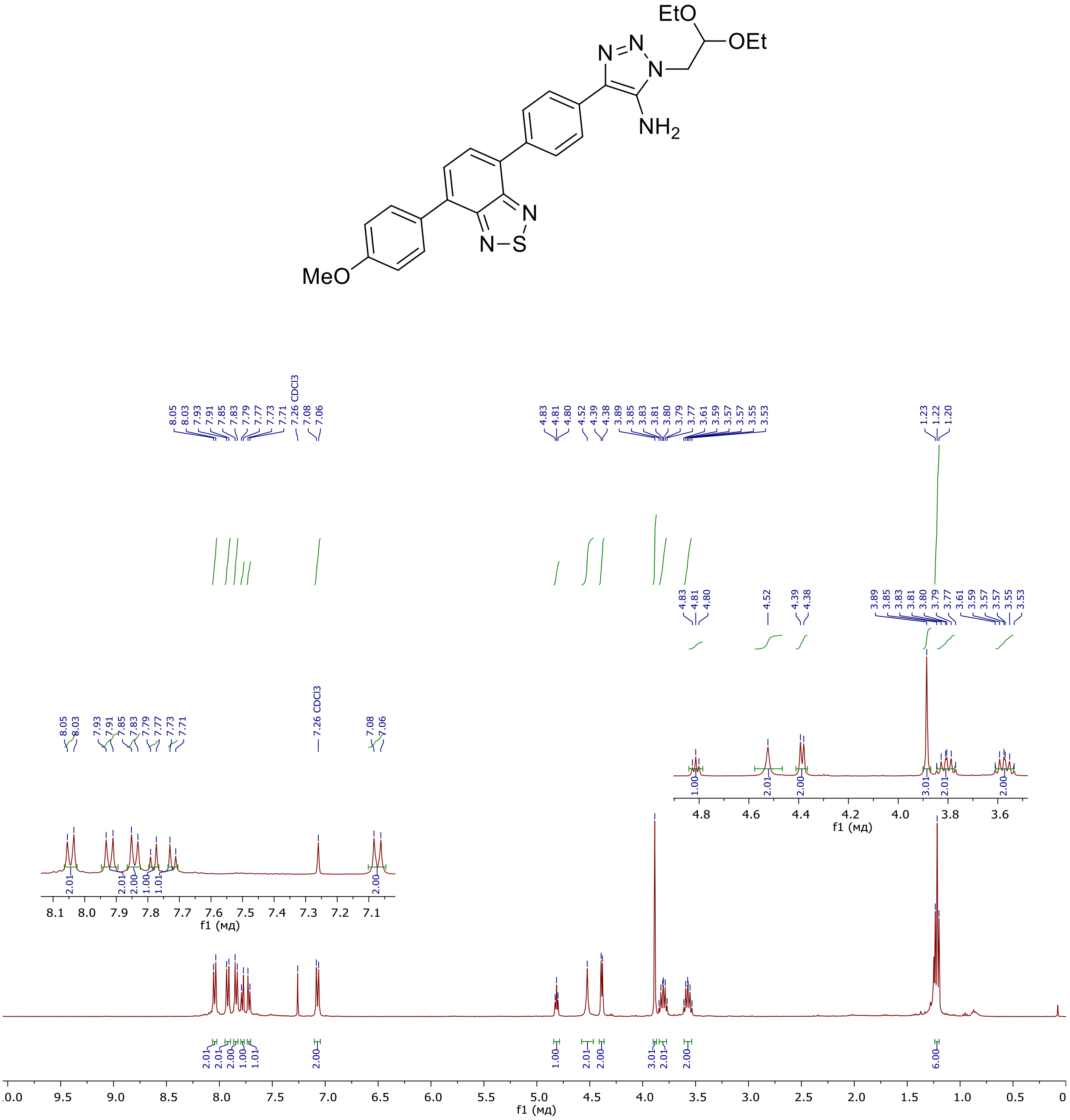

Figure S35. ^{1}H NMR (400 MHz, Chloroform-*d*) spectrum of compound **3l**.

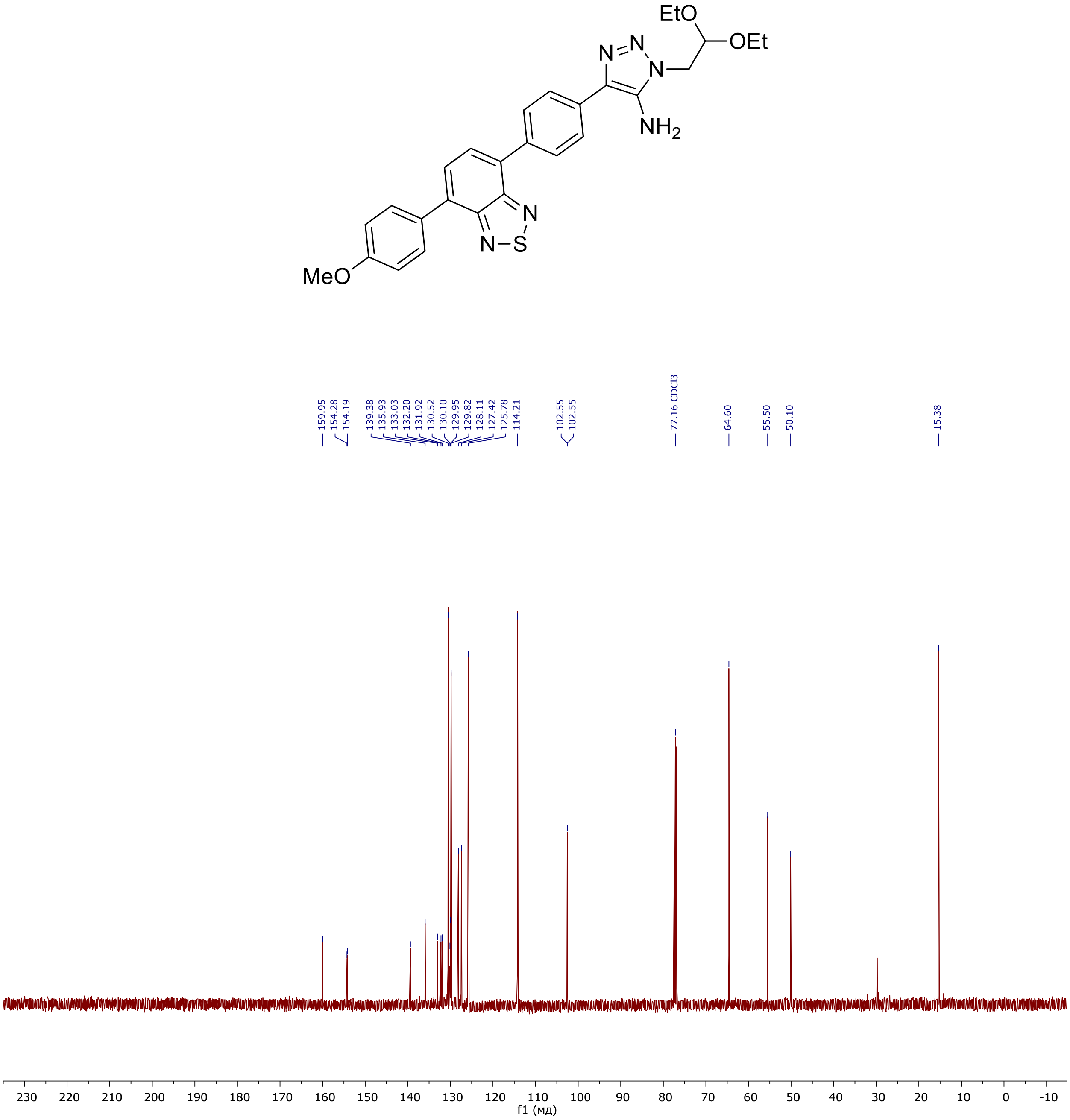

Figure S36. ^{13}C NMR (101 MHz, Chloroform-*d*) spectrum of compound **3l**.

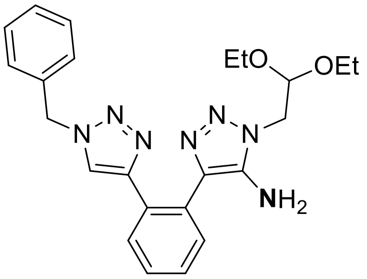

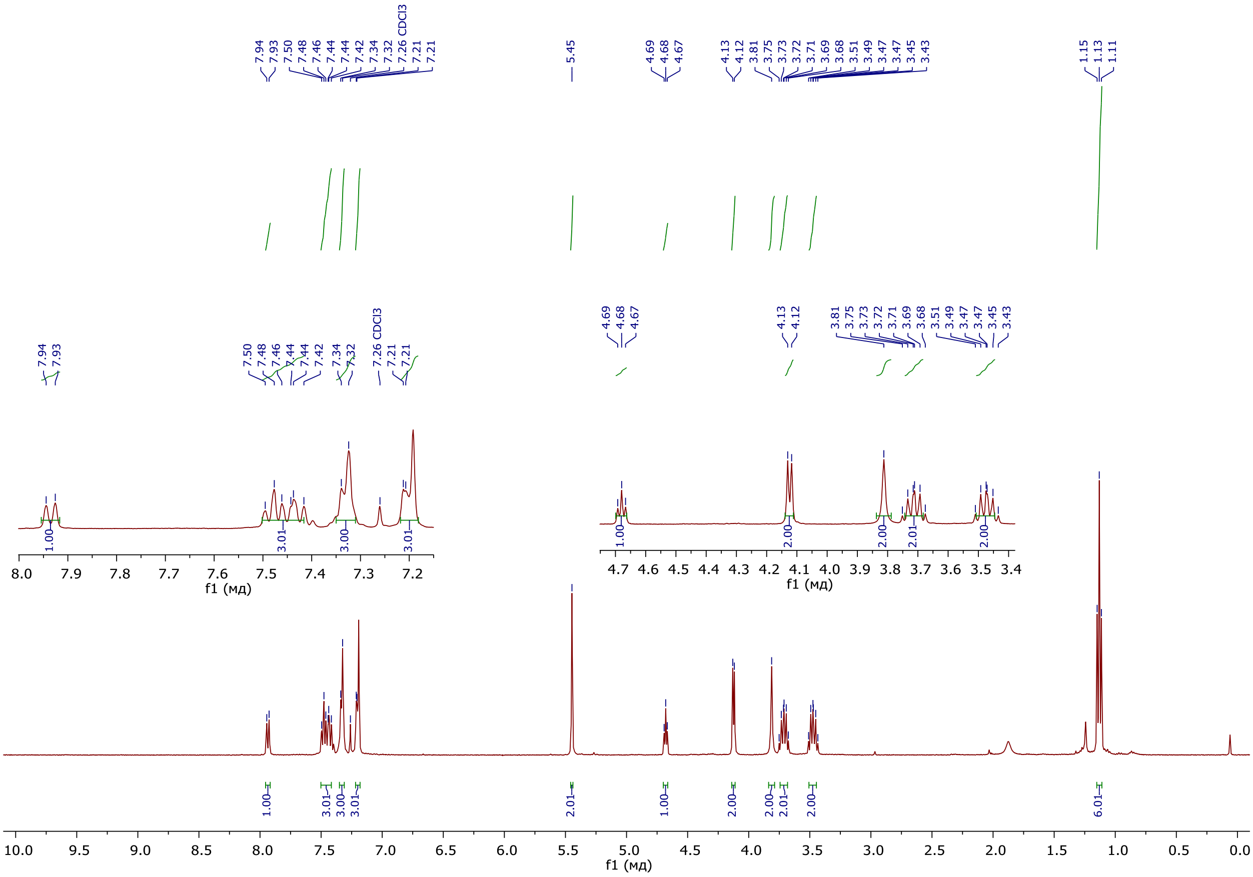

Figure S37. ^{1}H NMR (400 MHz, Chloroform-*d*) spectrum of compound **3m**.

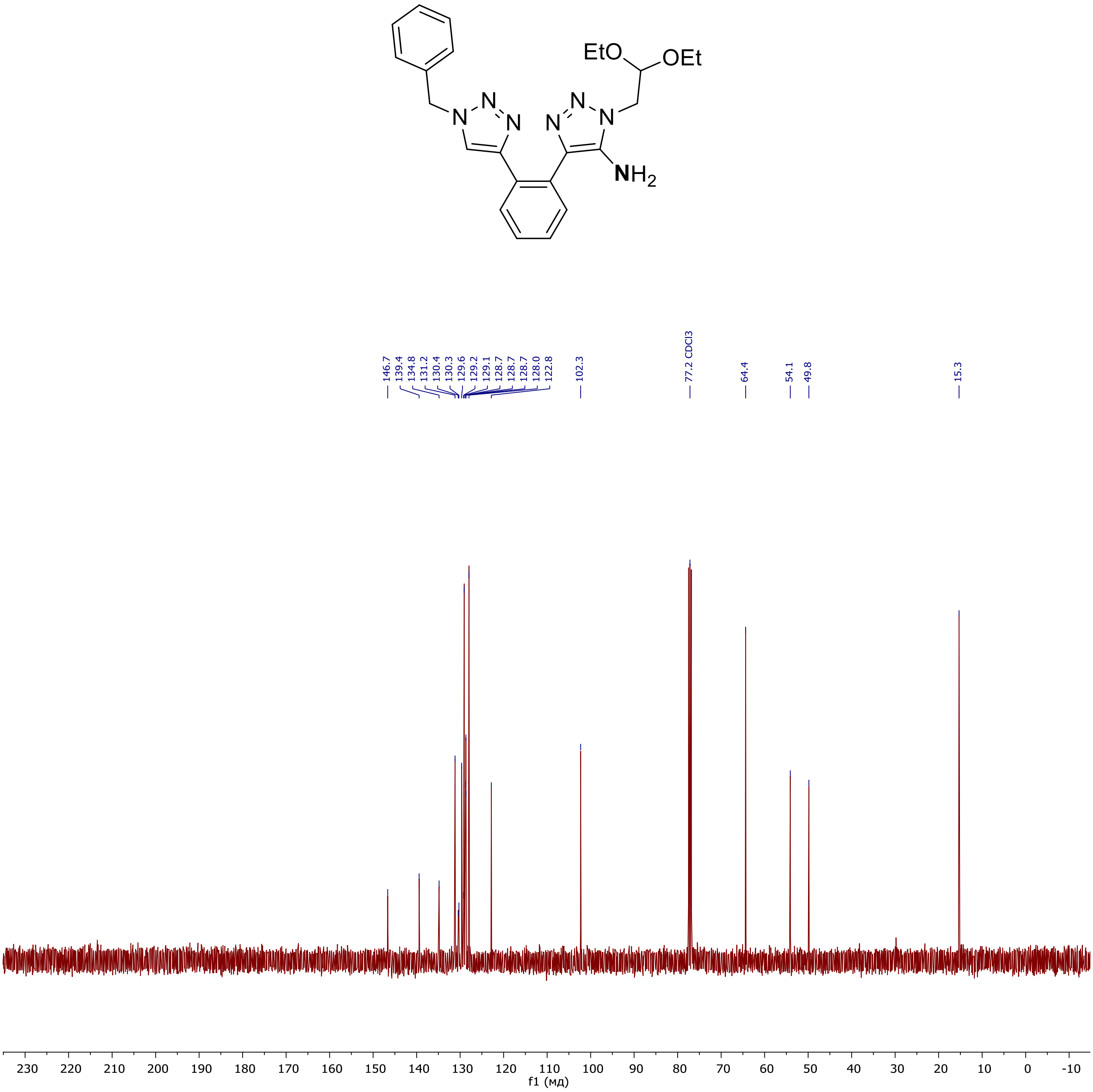

Figure S38. ^{13}C NMR (101 MHz, Chloroform-*d*) spectrum of compound **3m**.

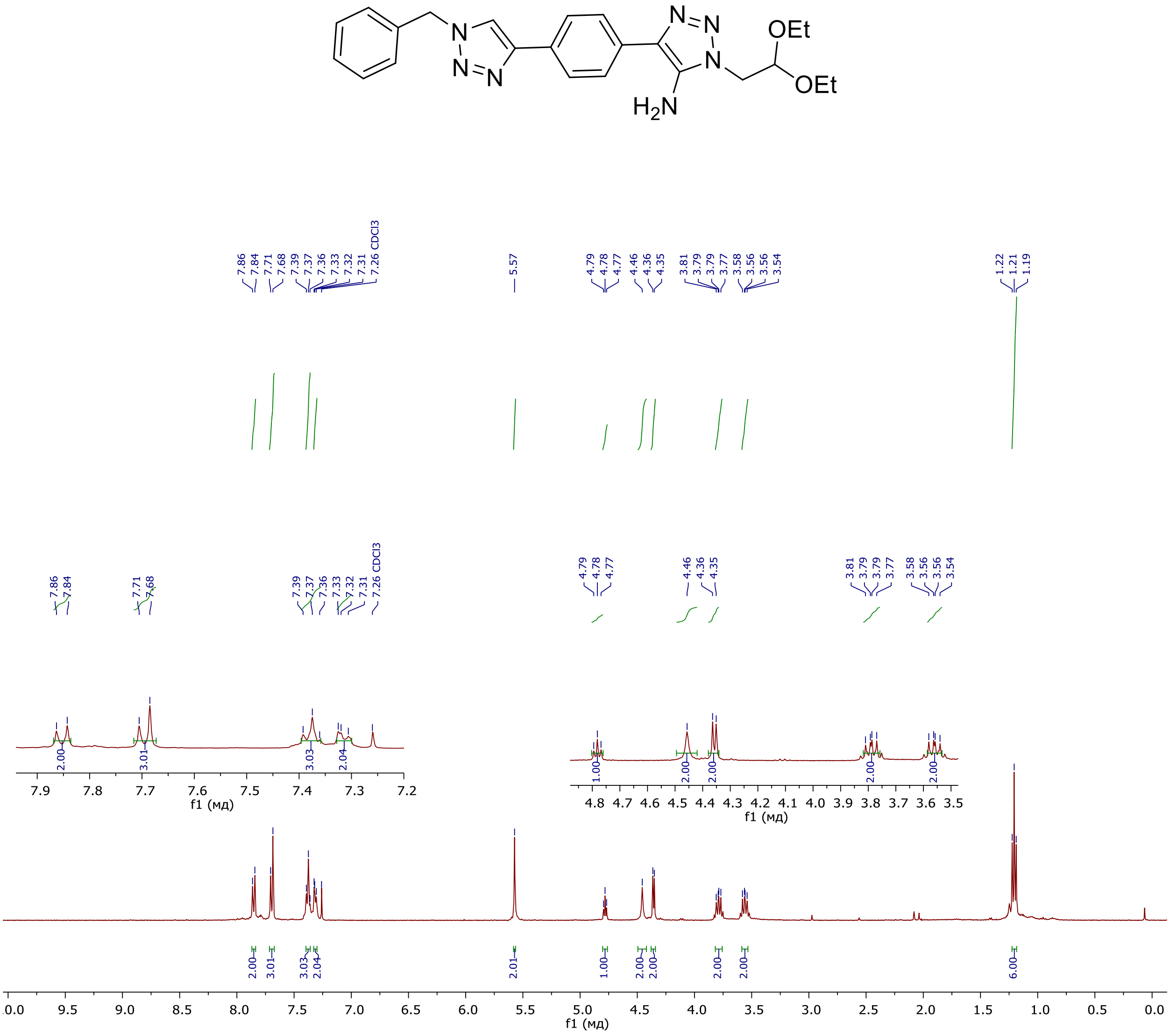

Figure S39. ^{1}H NMR (400 MHz, Chloroform-*d*) spectrum of compound **3n**.

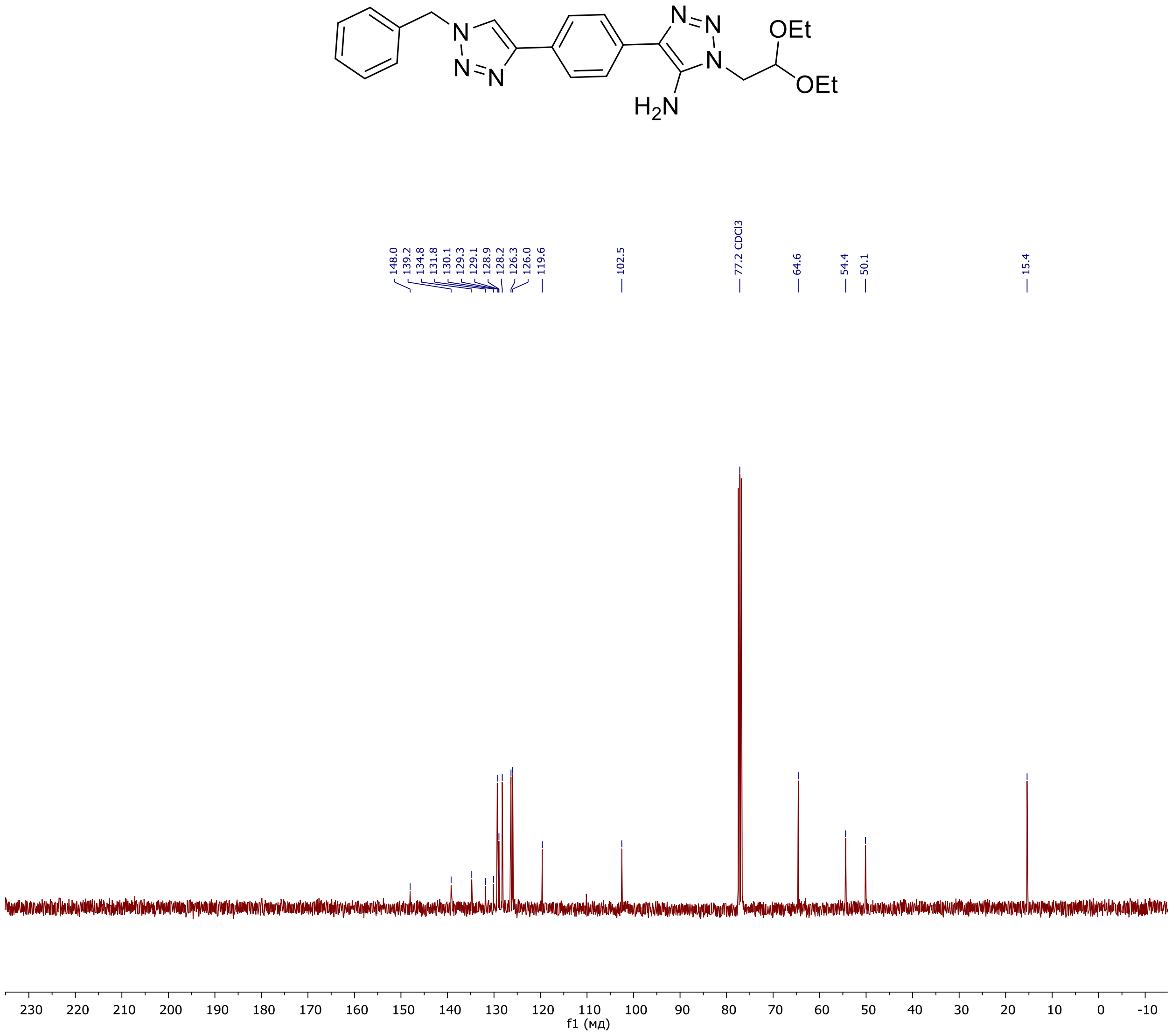

Figure S40. ^{13}C NMR (101 MHz, Chloroform-*d*) spectrum of compound **3n**.

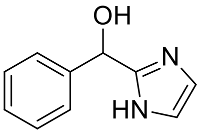

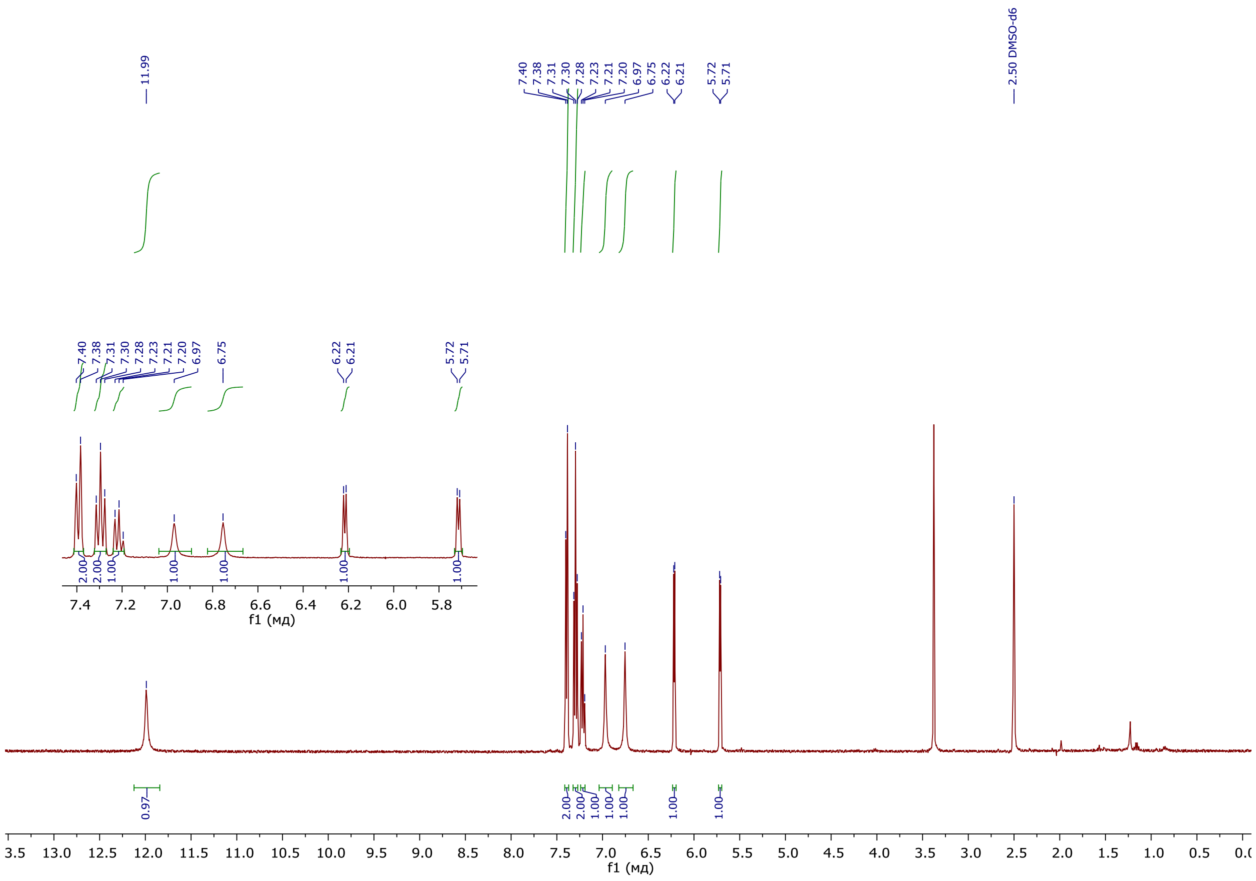

Figure S41. ^{1}H NMR (400 MHz, DMSO-d_6) spectrum of compound **4a**.

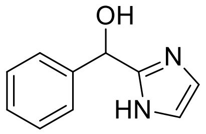

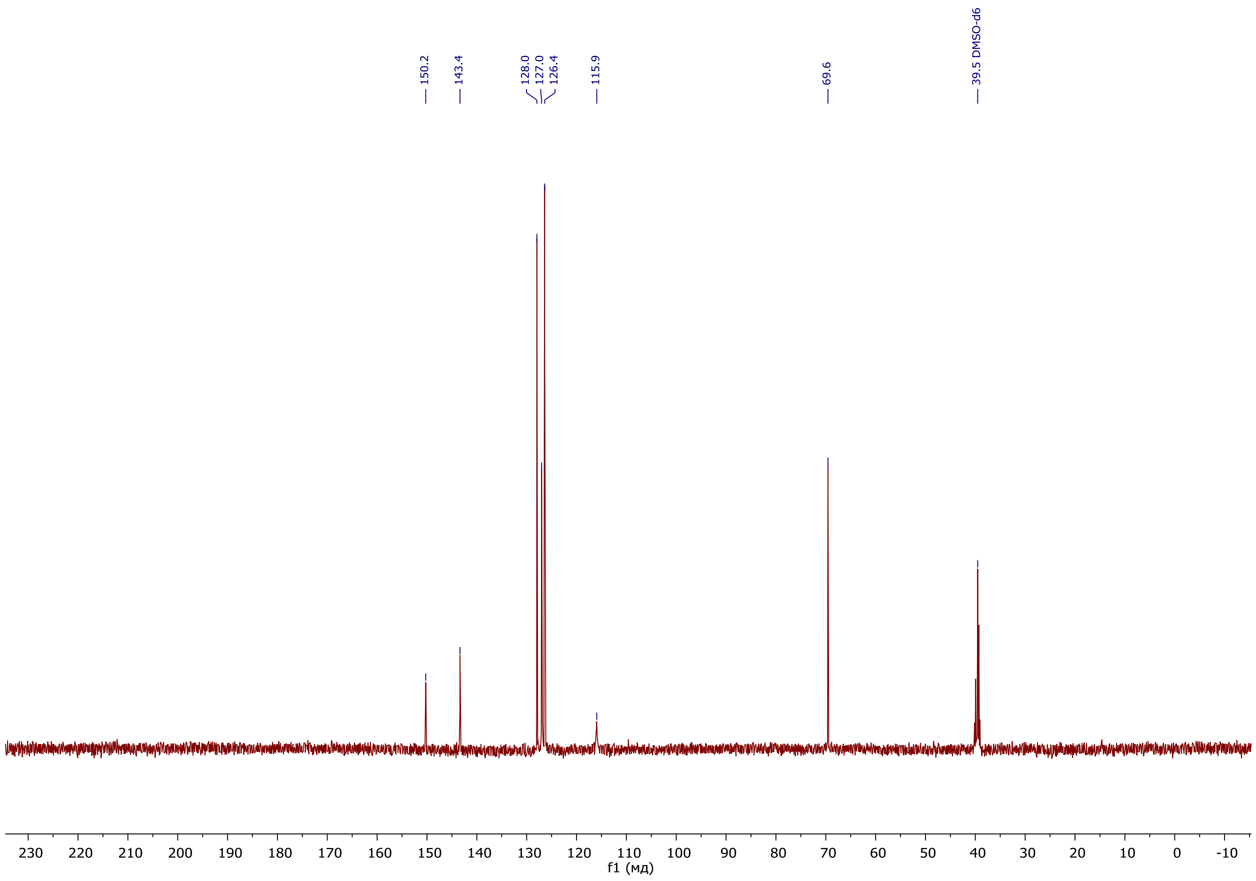

Figure S42. ^{13}C NMR (101 MHz, DMSO-d_6) spectrum of compound **4a**.

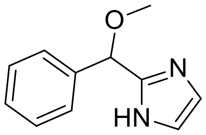

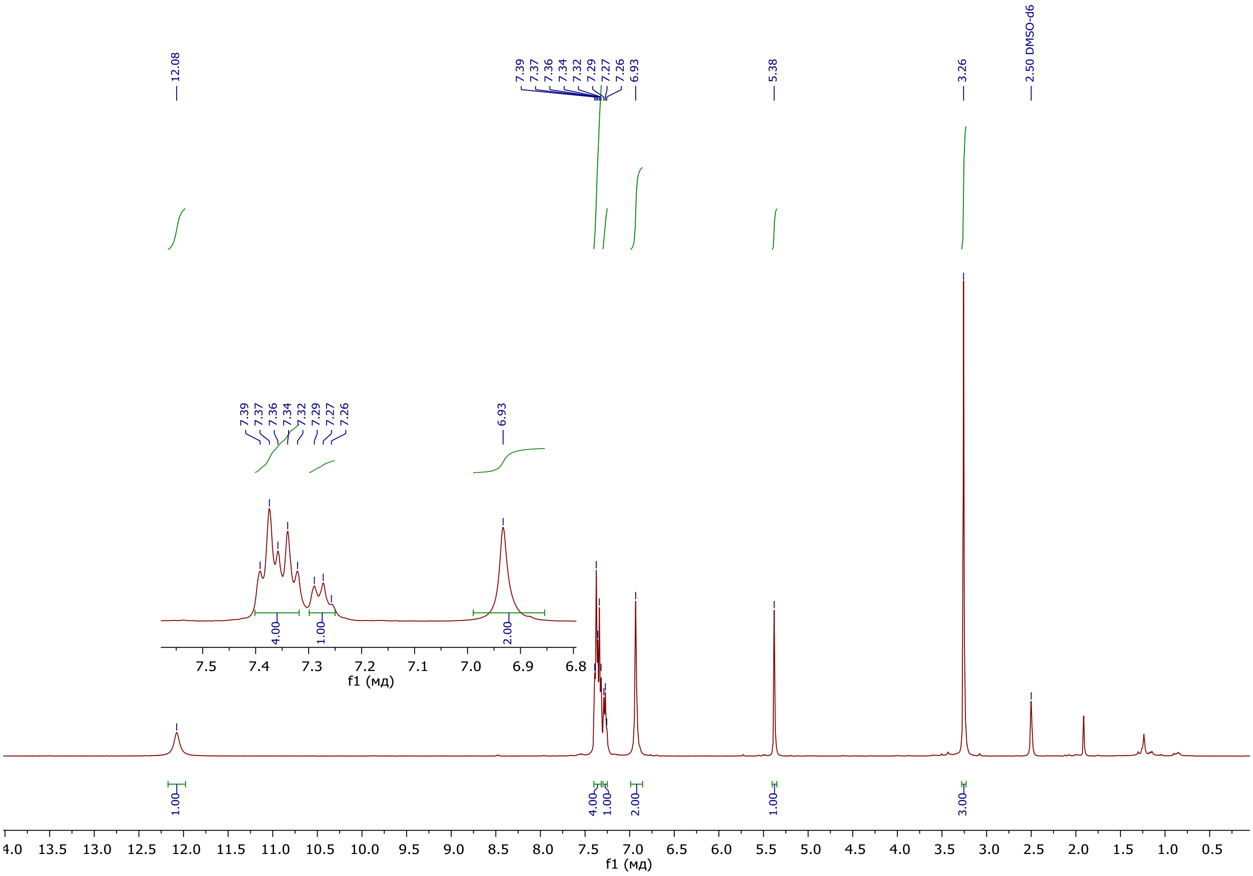

Figure 43 ^{1}H NMR (400 MHz, DMSO-d_6) spectrum of compound **4b**.

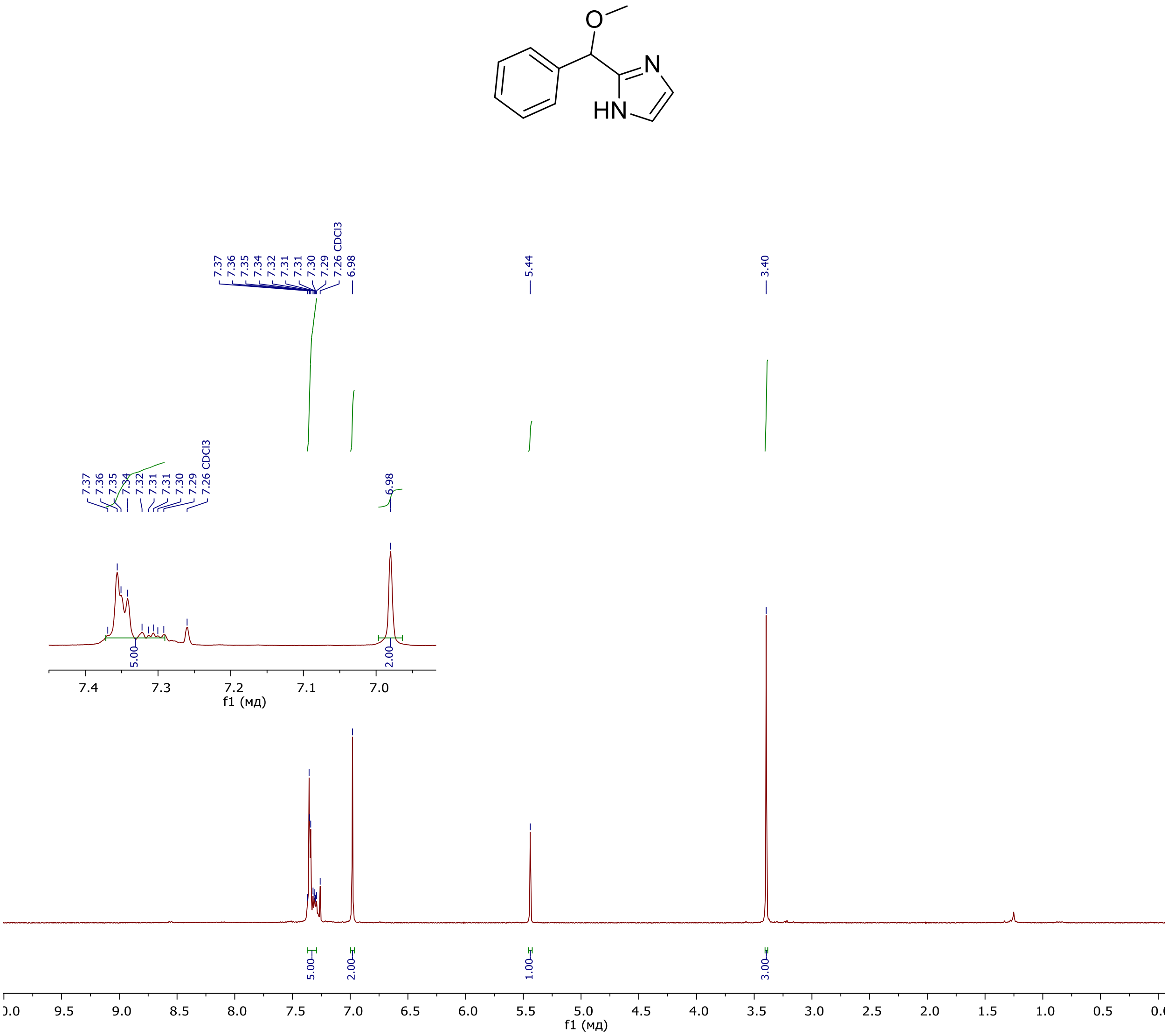

Figure 44 ^{1}H NMR (400 MHz, Chloroform-*d*) spectrum of compound **4b**.

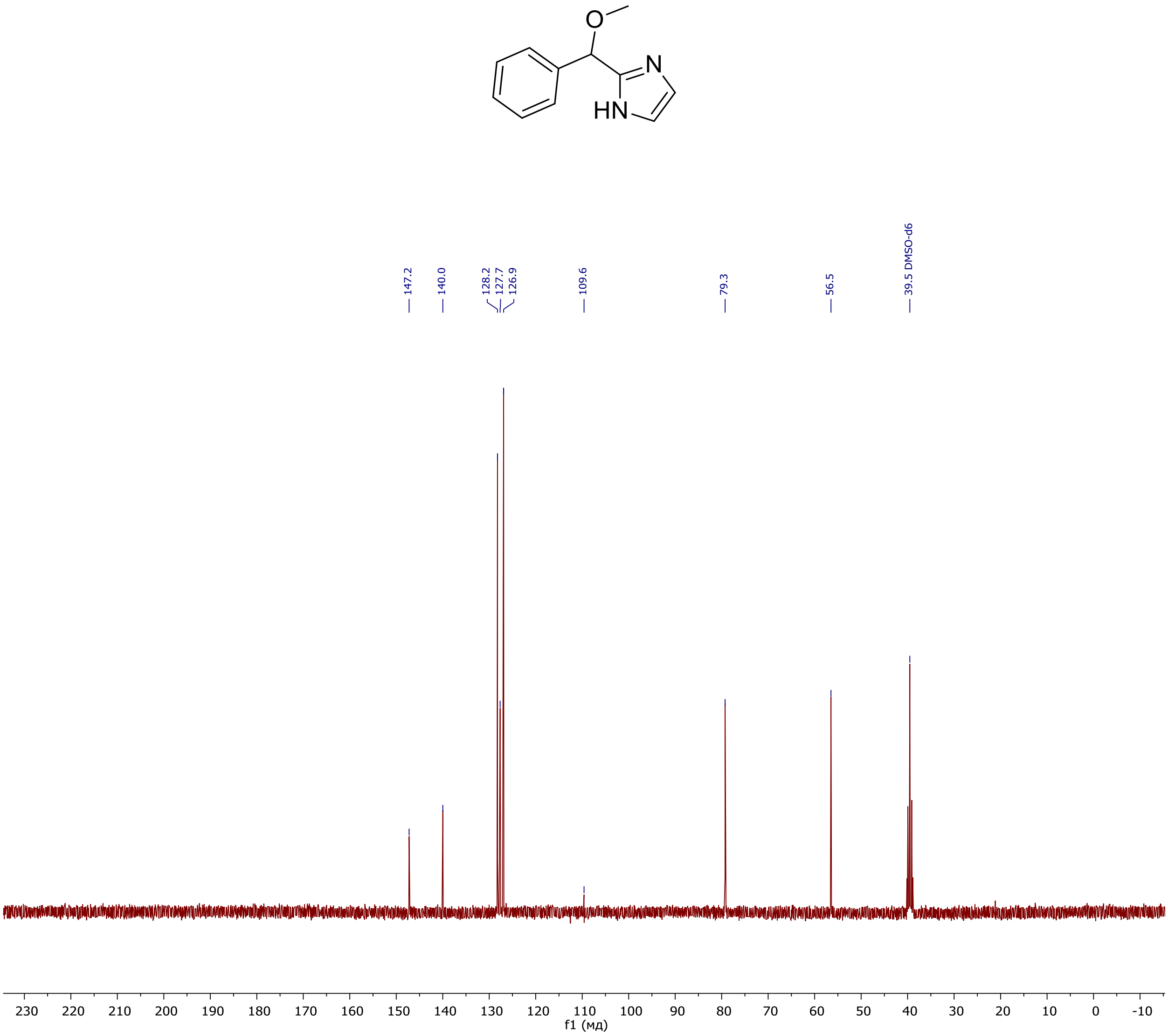

Figure S45. ^{13}C NMR (101 MHz, DMSO-d_6) spectrum of compound **4b**.

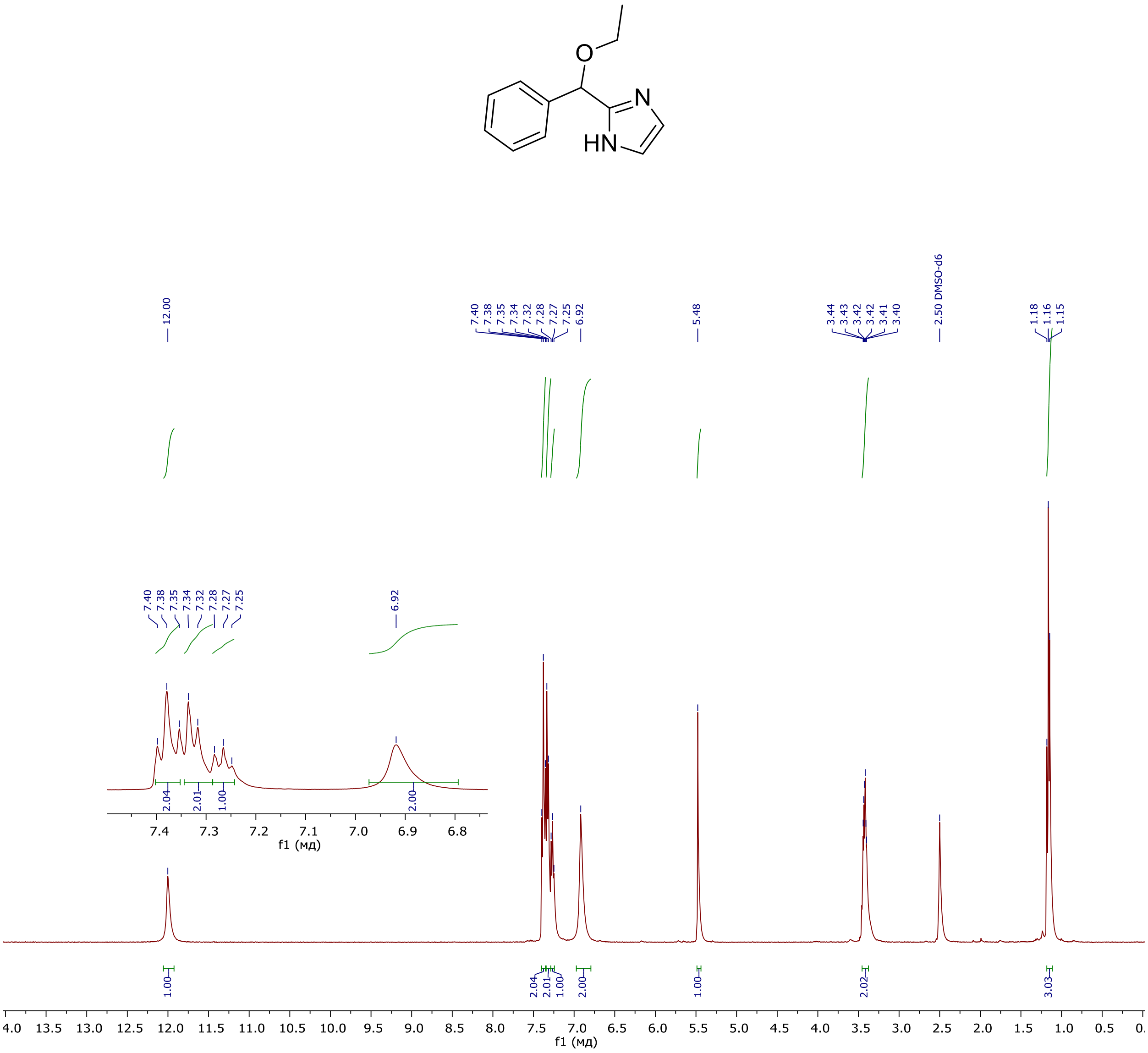

Figure 46 ^{1}H NMR (400 MHz, DMSO-d_6) spectrum of compound **4c**.

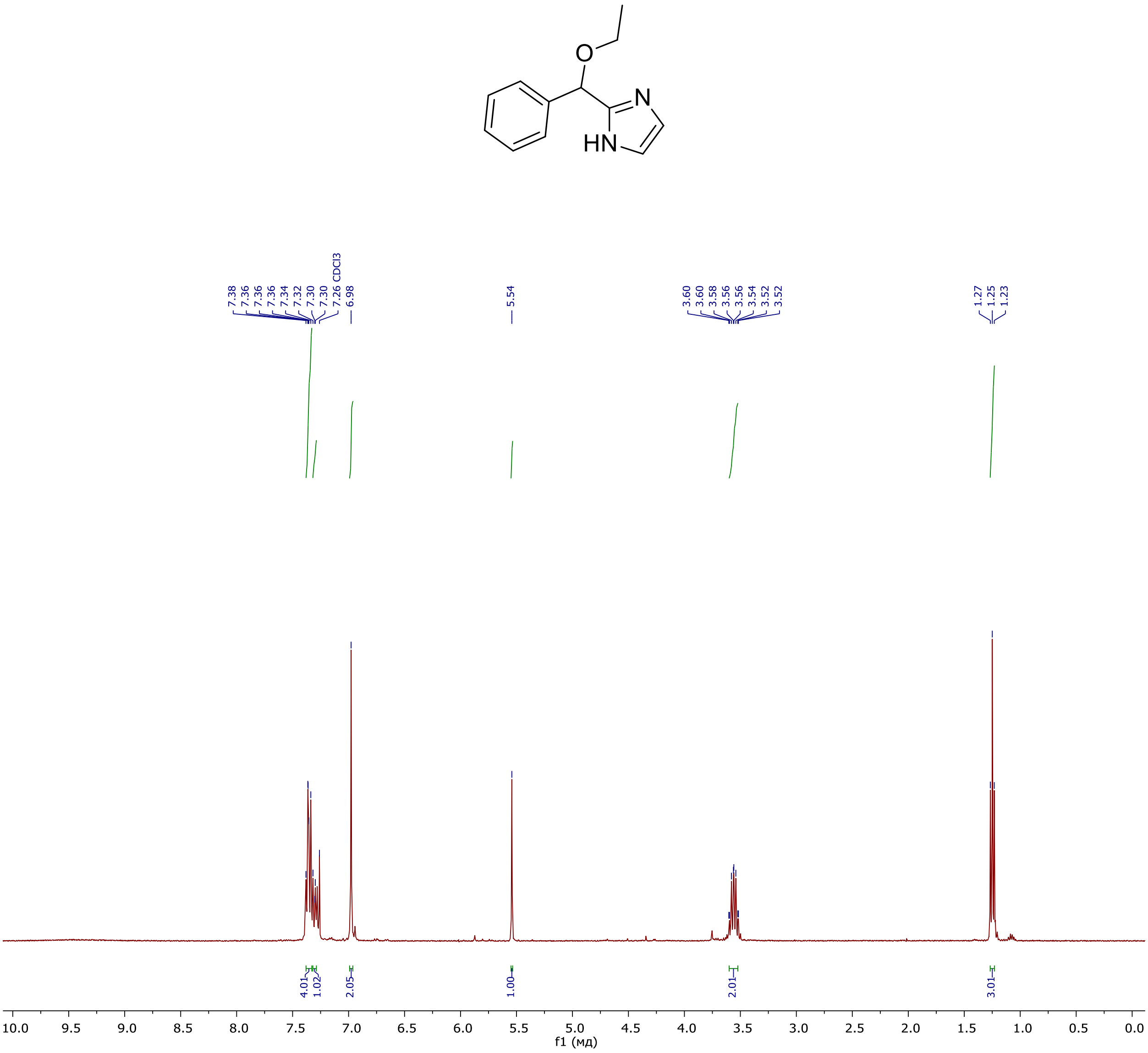

Figure 47 ^{1}H NMR (400 MHz, Chloroform-*d*) spectrum of compound **4c**.

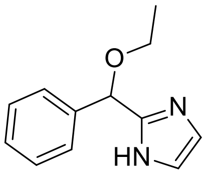

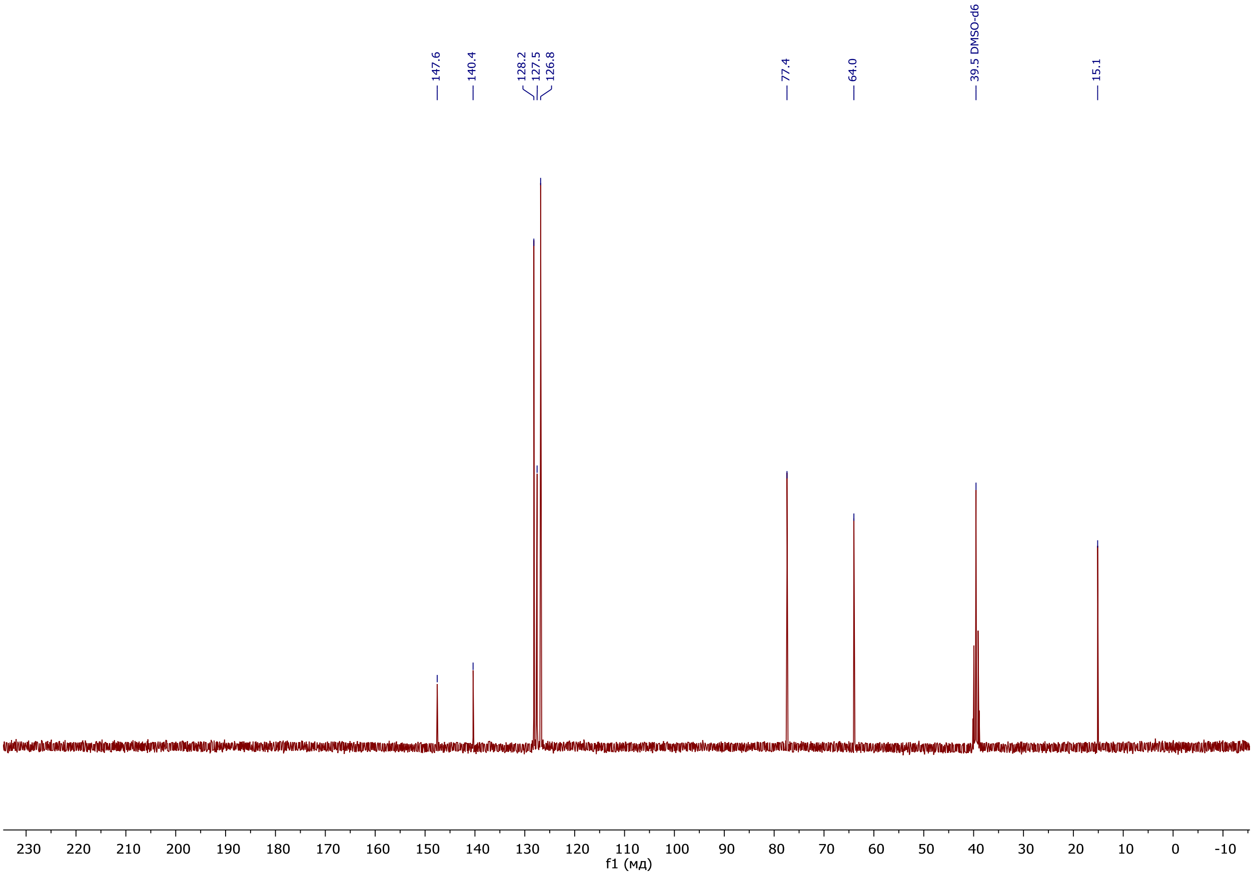

Figure S48. ^{13}C NMR (101 MHz, DMSO-d_6) spectrum of compound **4c**.

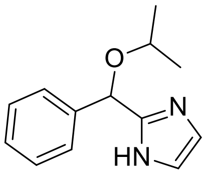

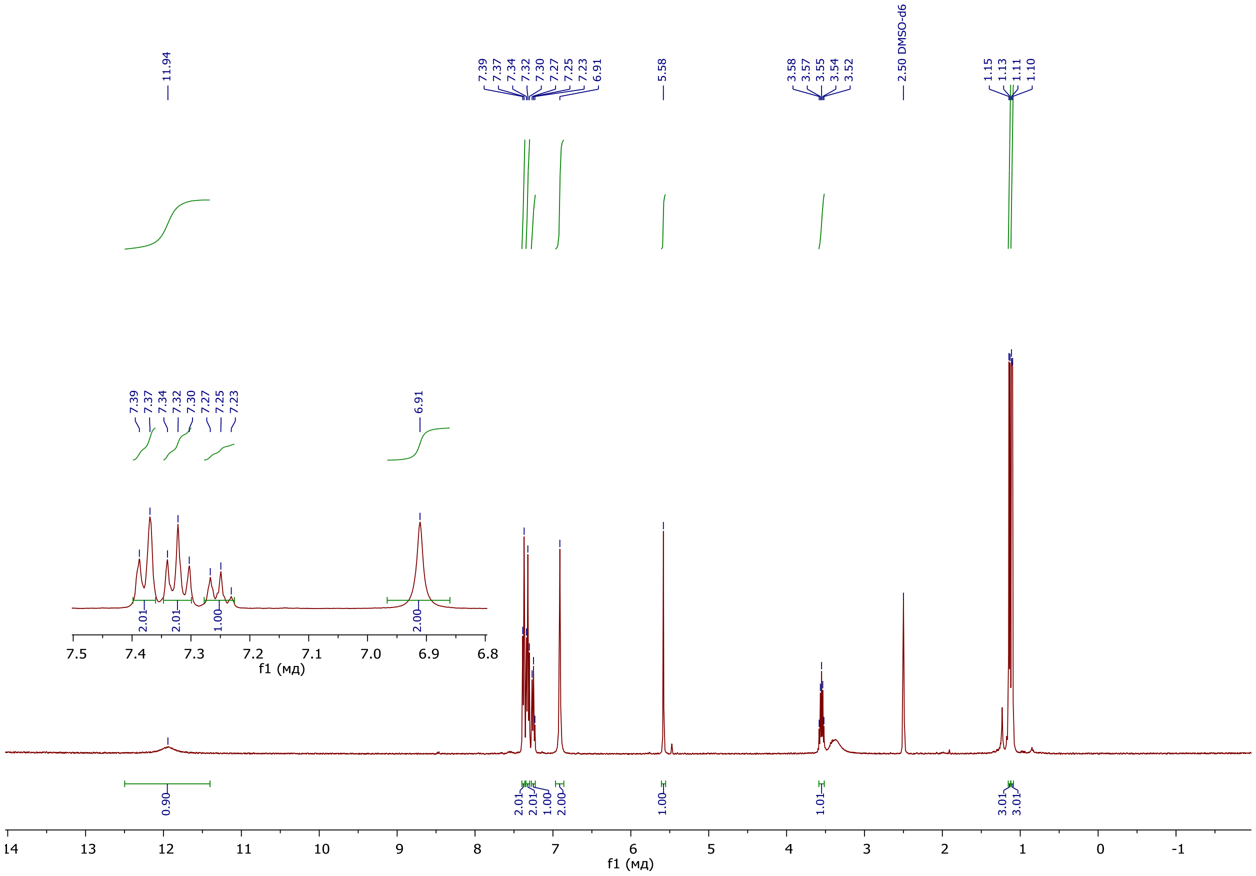

Figure S49. ^{1}H NMR (400 MHz, DMSO-d_6) spectrum of compound **4d**.

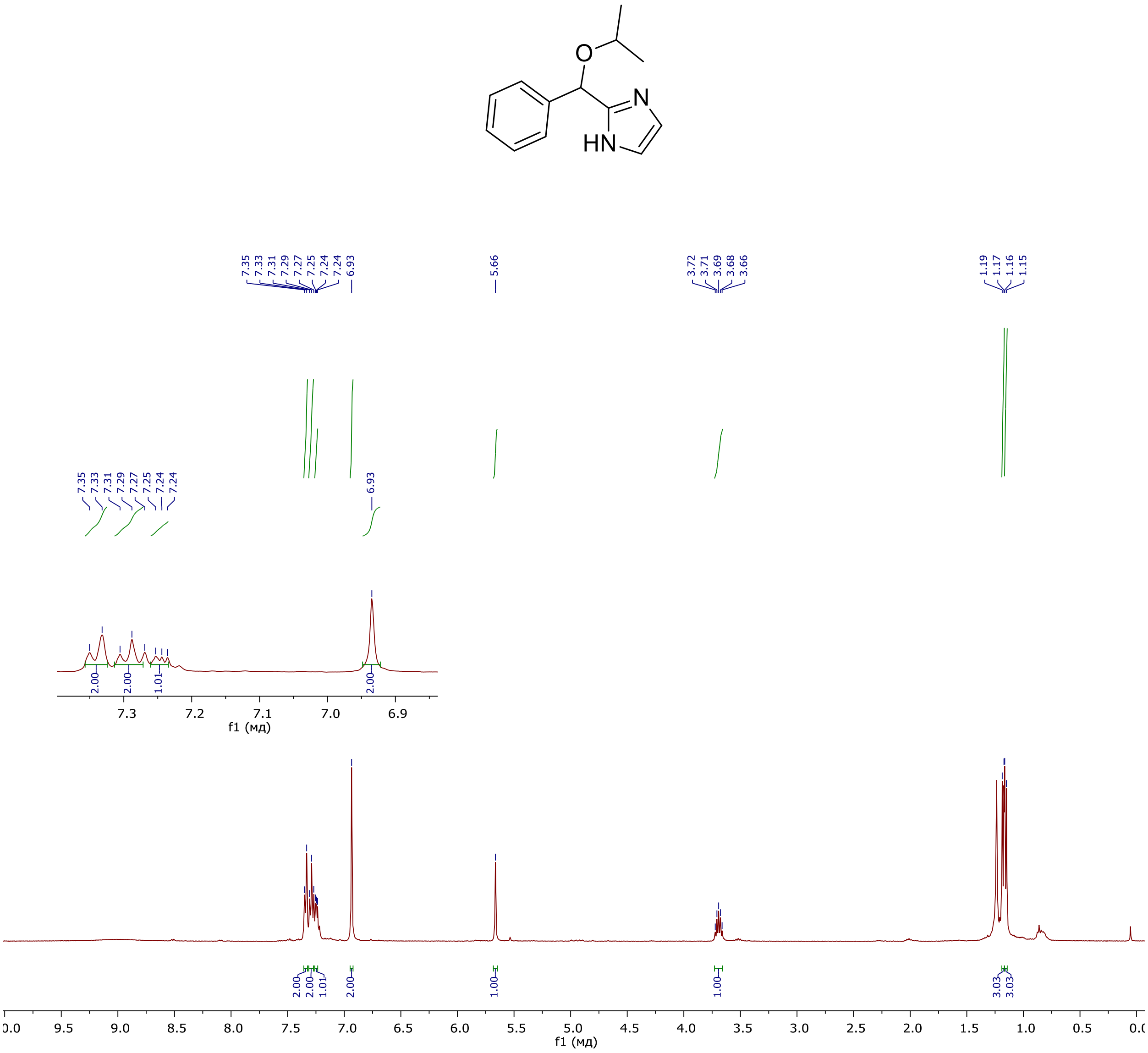

Figure S50. ^{1}H NMR (400 MHz, Chloroform-*d*) spectrum of compound **4d**.

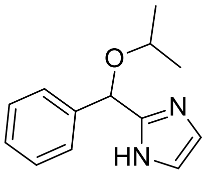

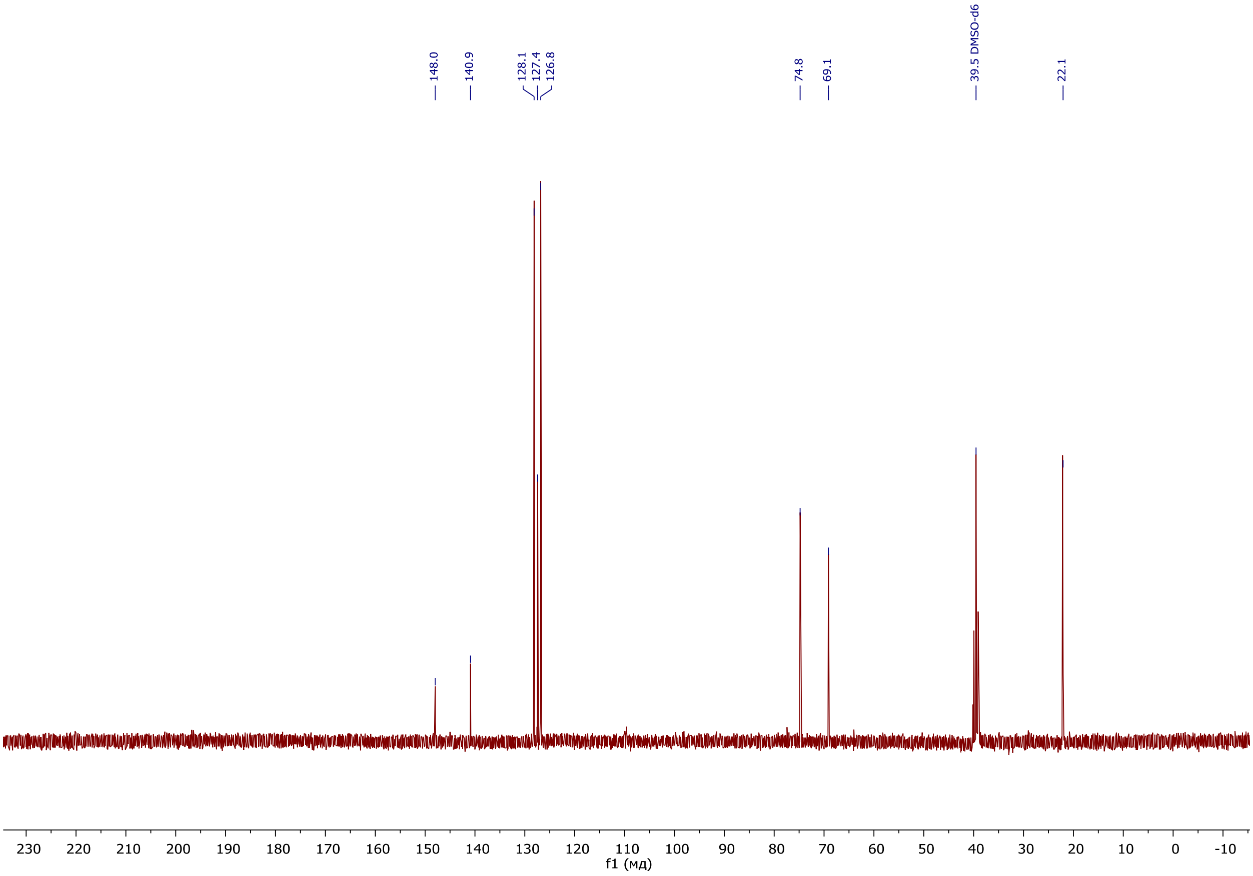

Figure S51. ^{13}C NMR (101 MHz, DMSO-d_6) spectrum of compound **4d**.

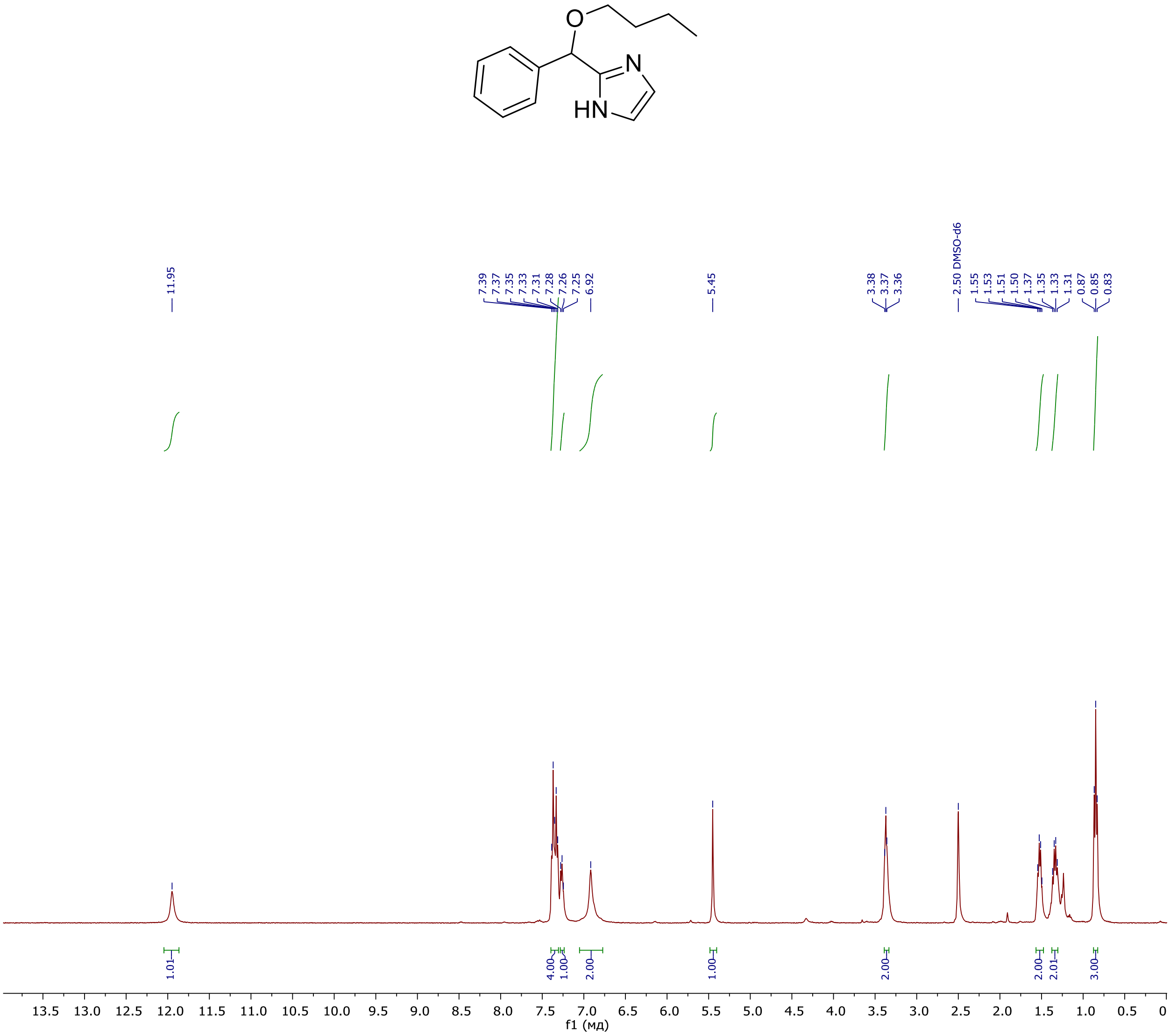

Figure S52. ^{1}H NMR (400 MHz, DMSO-d_6) spectrum of compound **4e**.

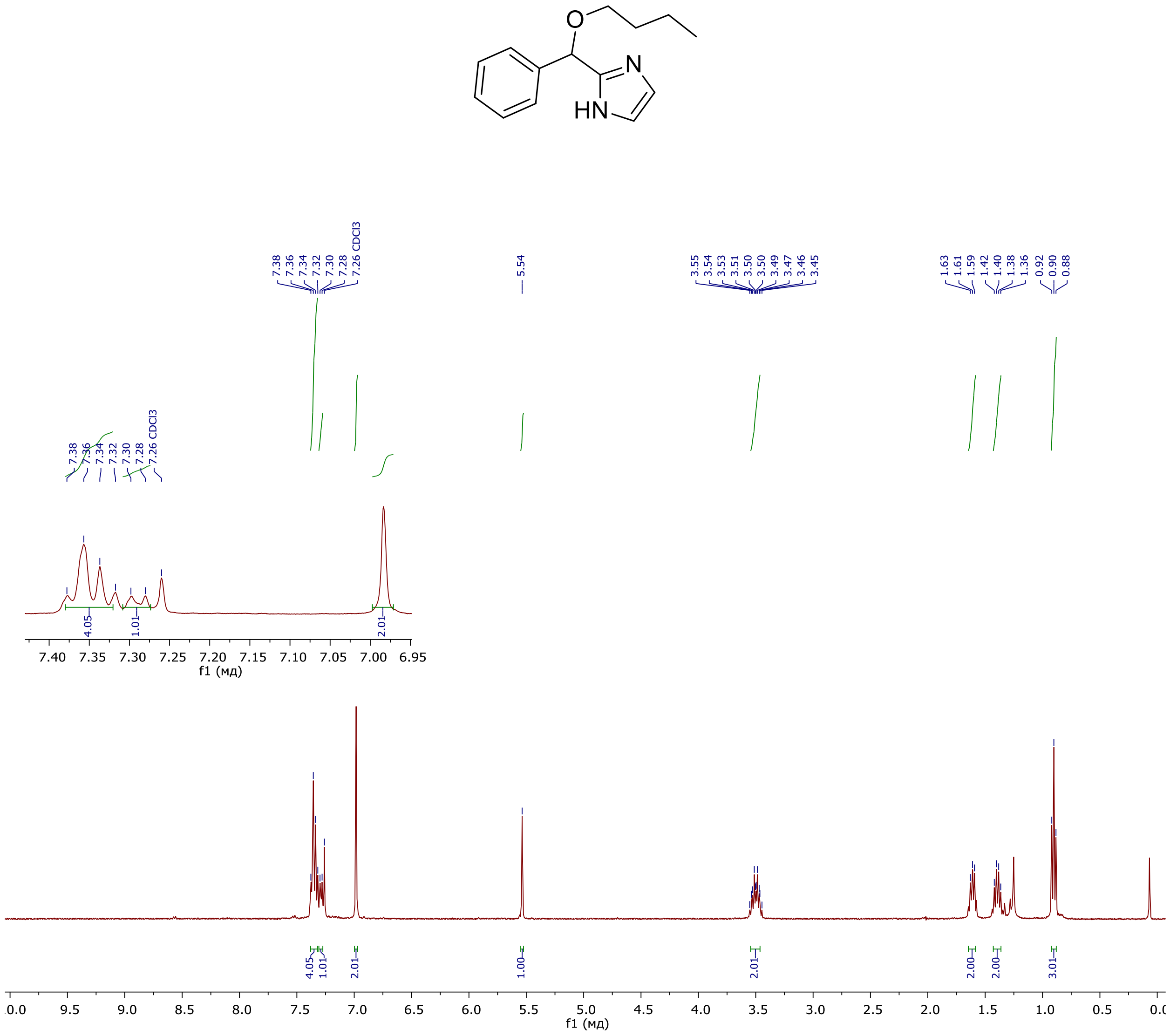

Figure S53. ^{1}H NMR (400 MHz, Chloroform-*d*) spectrum of compound **4e**.

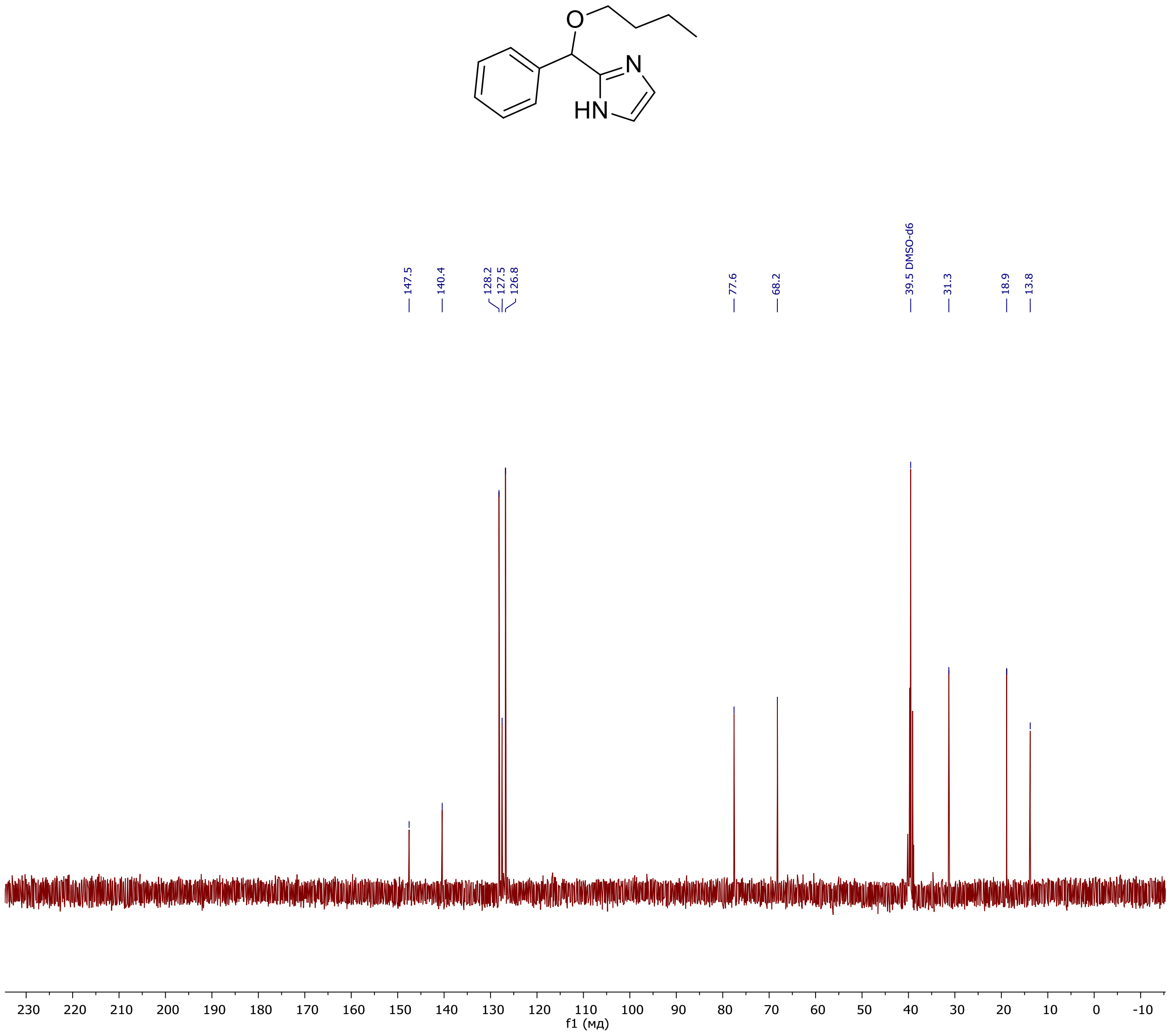

Figure S54. ^{13}C NMR (101 MHz, DMSO-d_6) spectrum of compound **4e**.

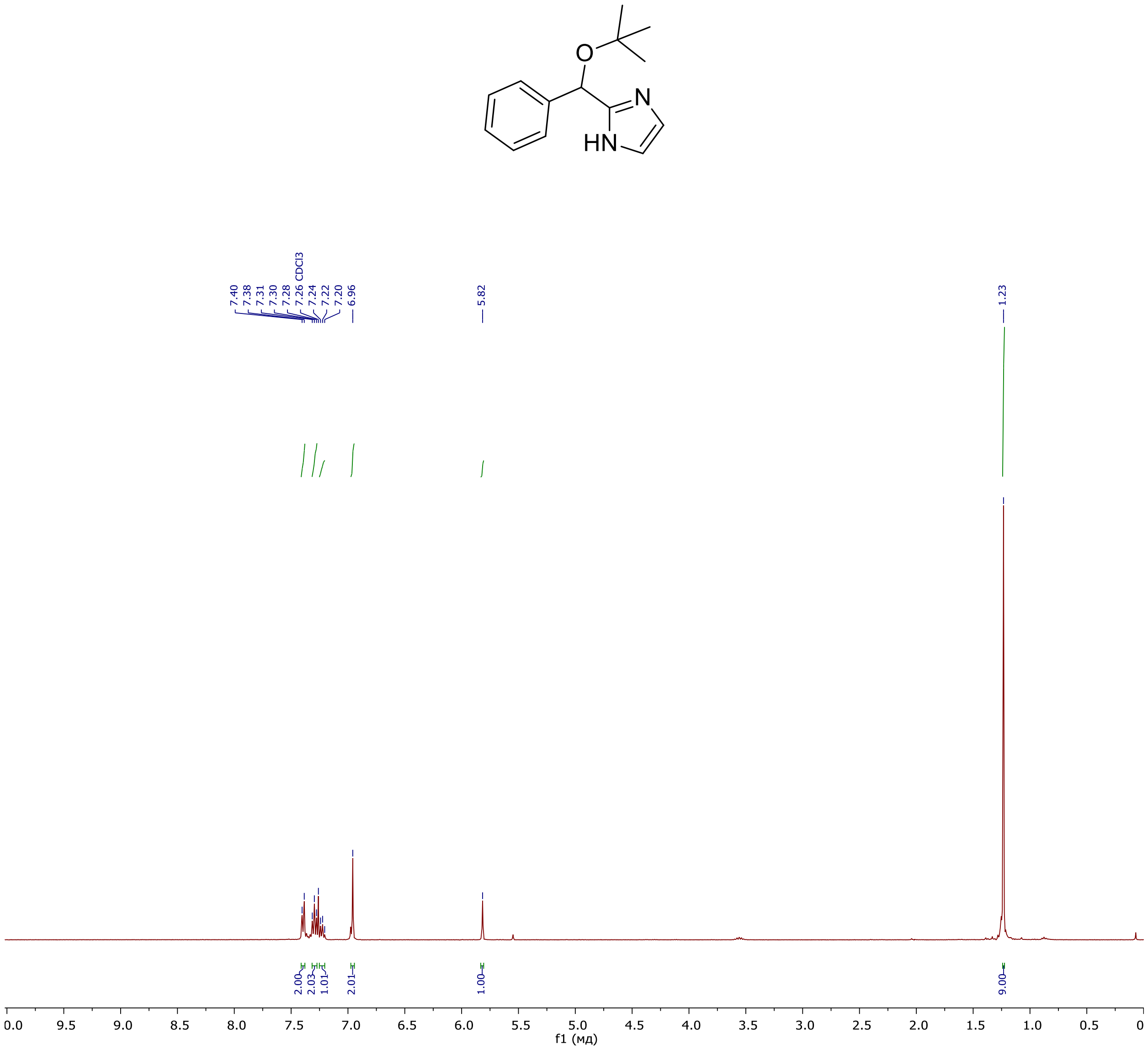

Figure S55. ^{1}H NMR (400 MHz, Chloroform-*d*) spectrum of compound **4f**.

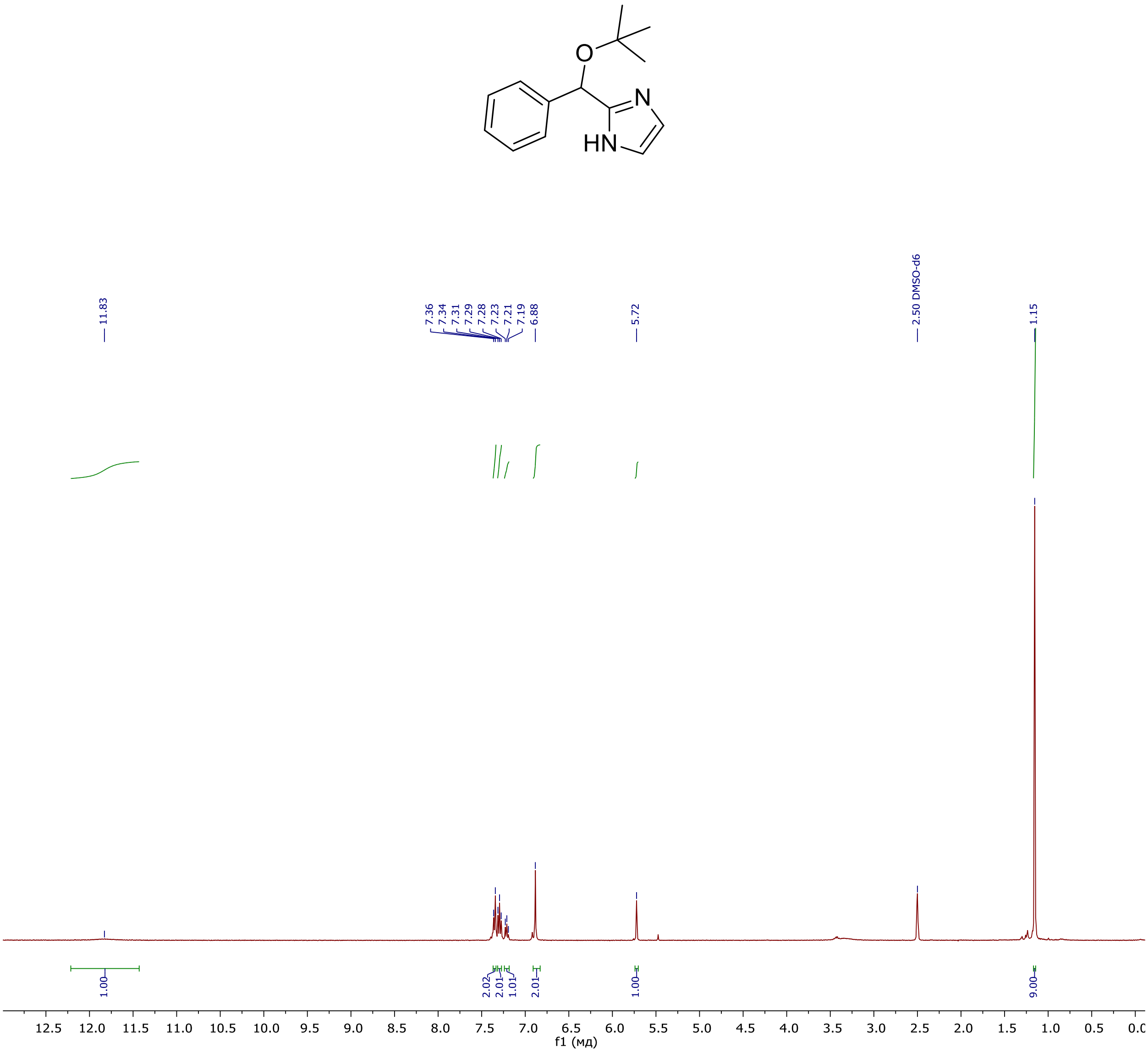

Figure S56. ^{1}H NMR (400 MHz, DMSO-d_6) spectrum of compound **4f**.

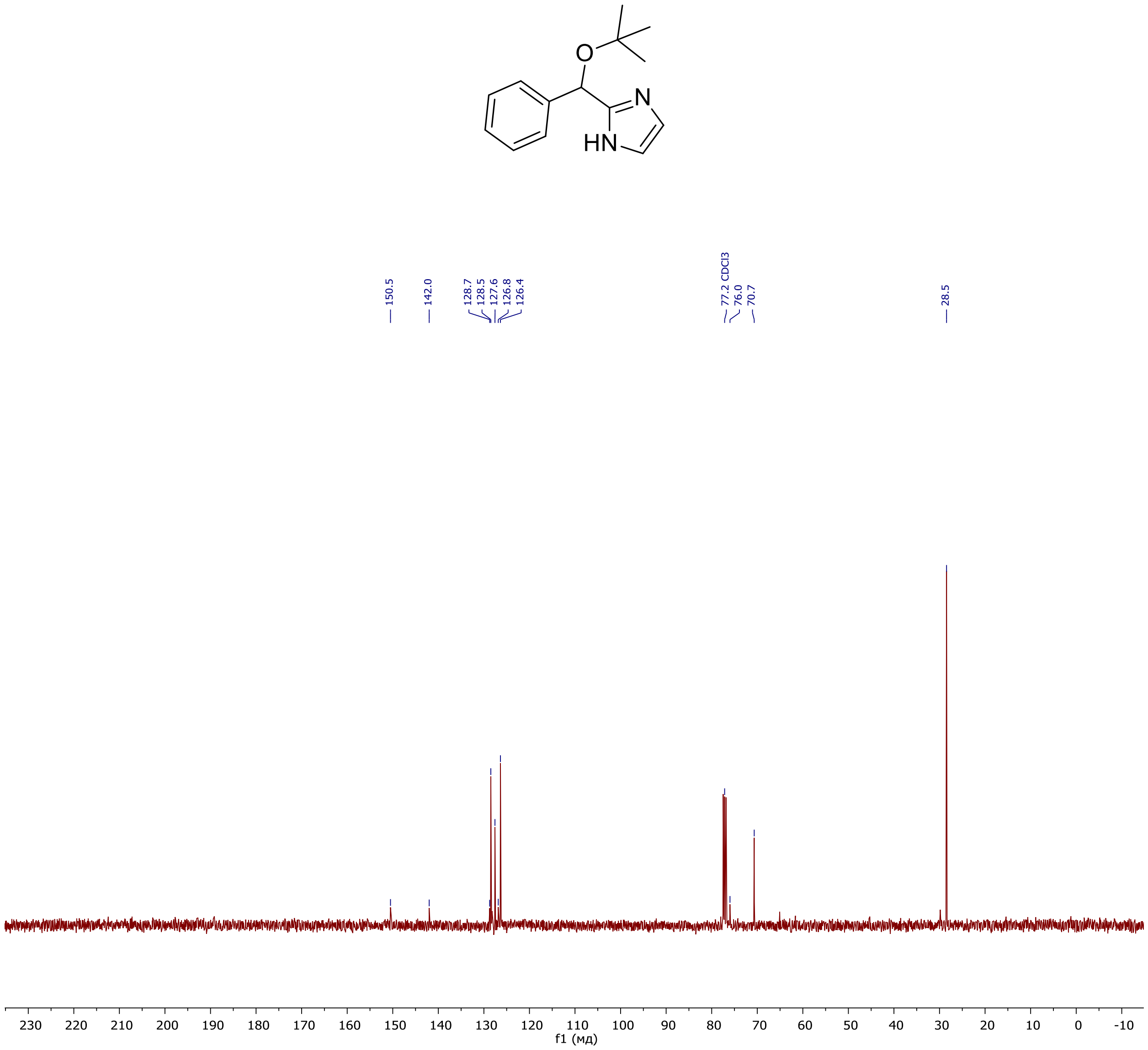

Figure S57. ^{13}C NMR (101 MHz, Chloroform-*d*) spectrum of compound **4f**.

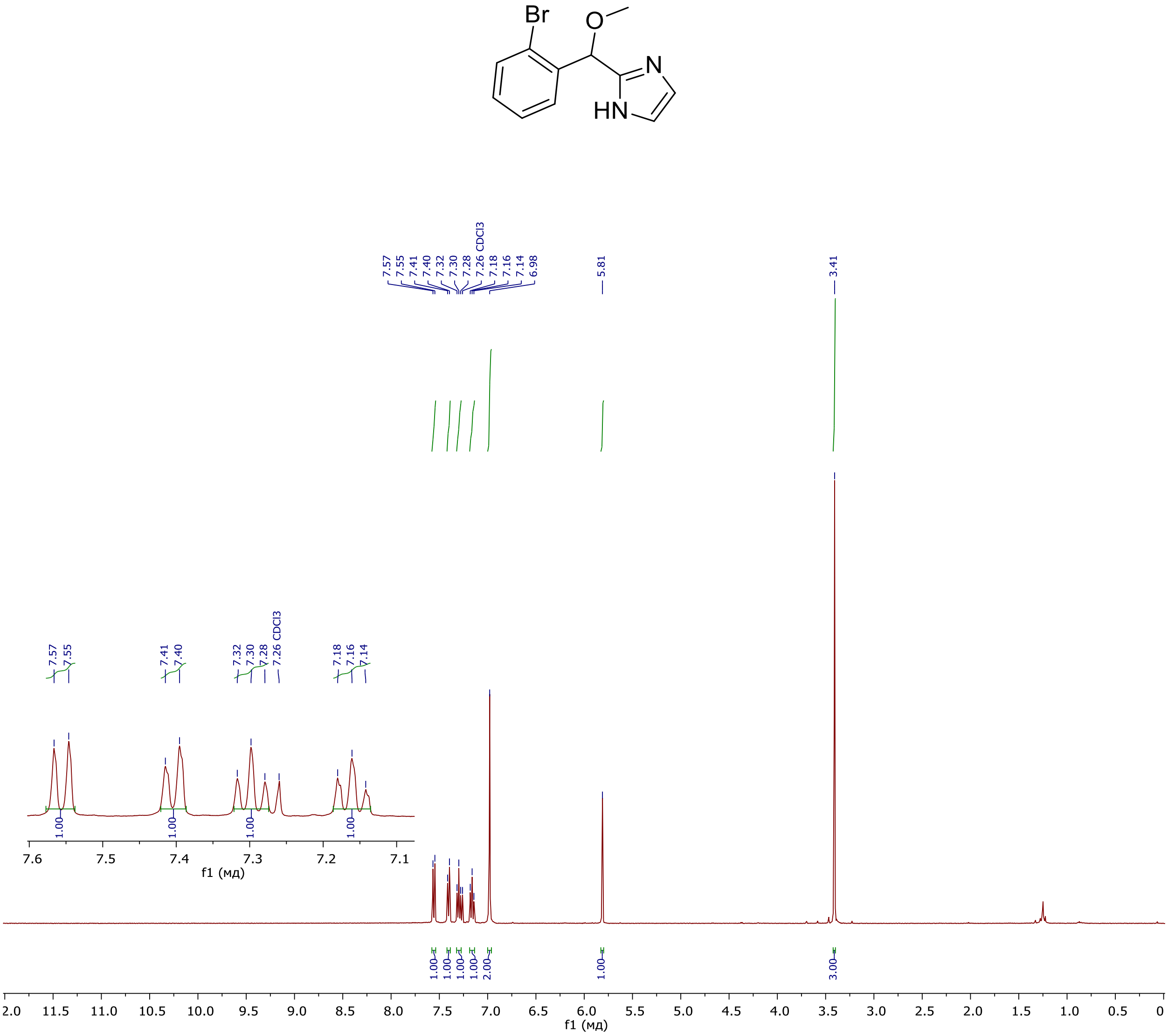

Figure S58. ¹H NMR (400 MHz, Chloroform-*d*) spectrum of compound **4g**.

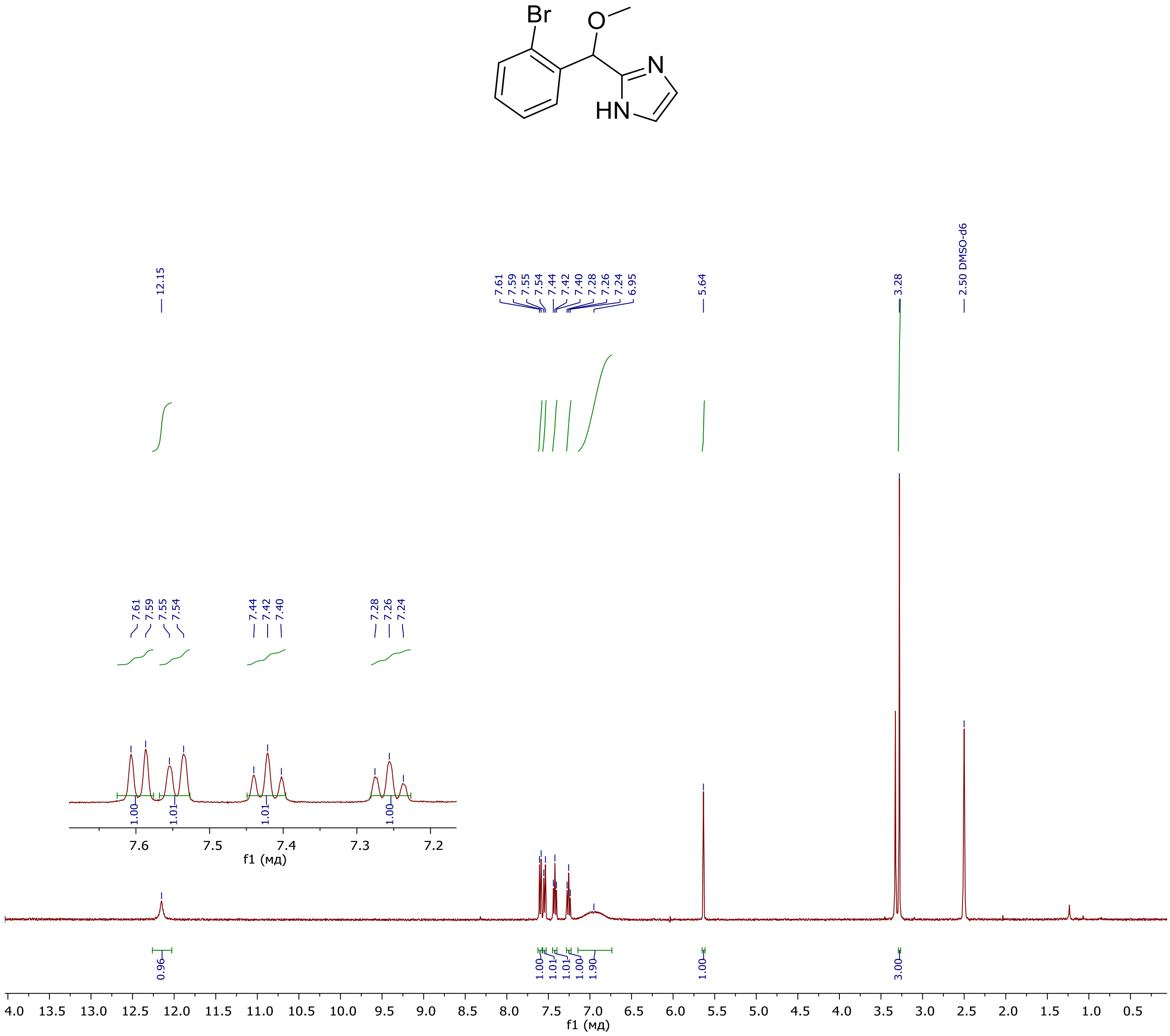

Figure S59. ^{1}H NMR (400 MHz, DMSO-d_6) spectrum of compound **4g**.

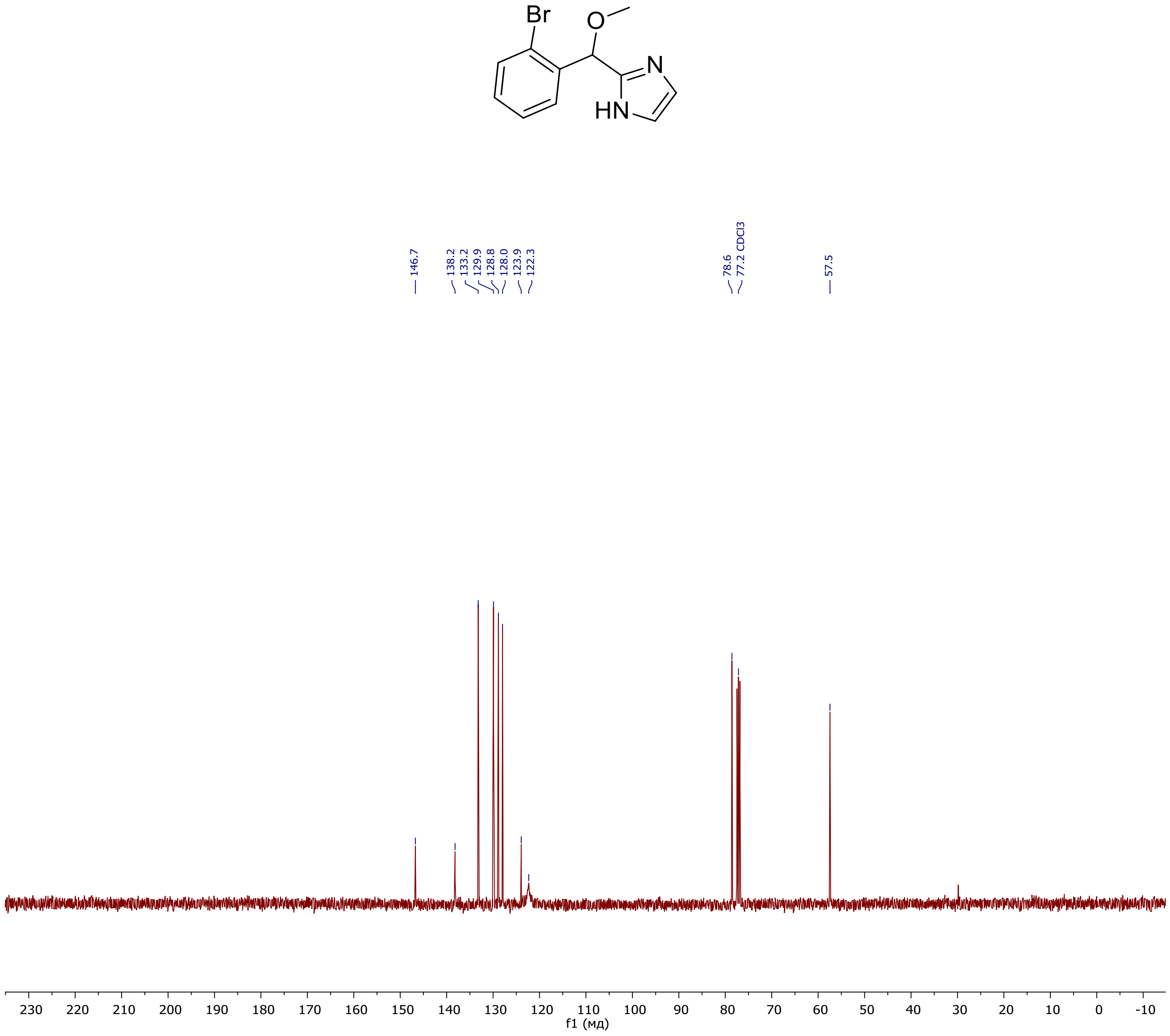

Figure S60. ^{13}C NMR (101 MHz, Chloroform-*d*) spectrum of compound **4g**.

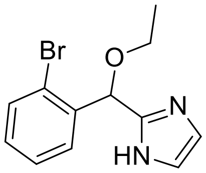

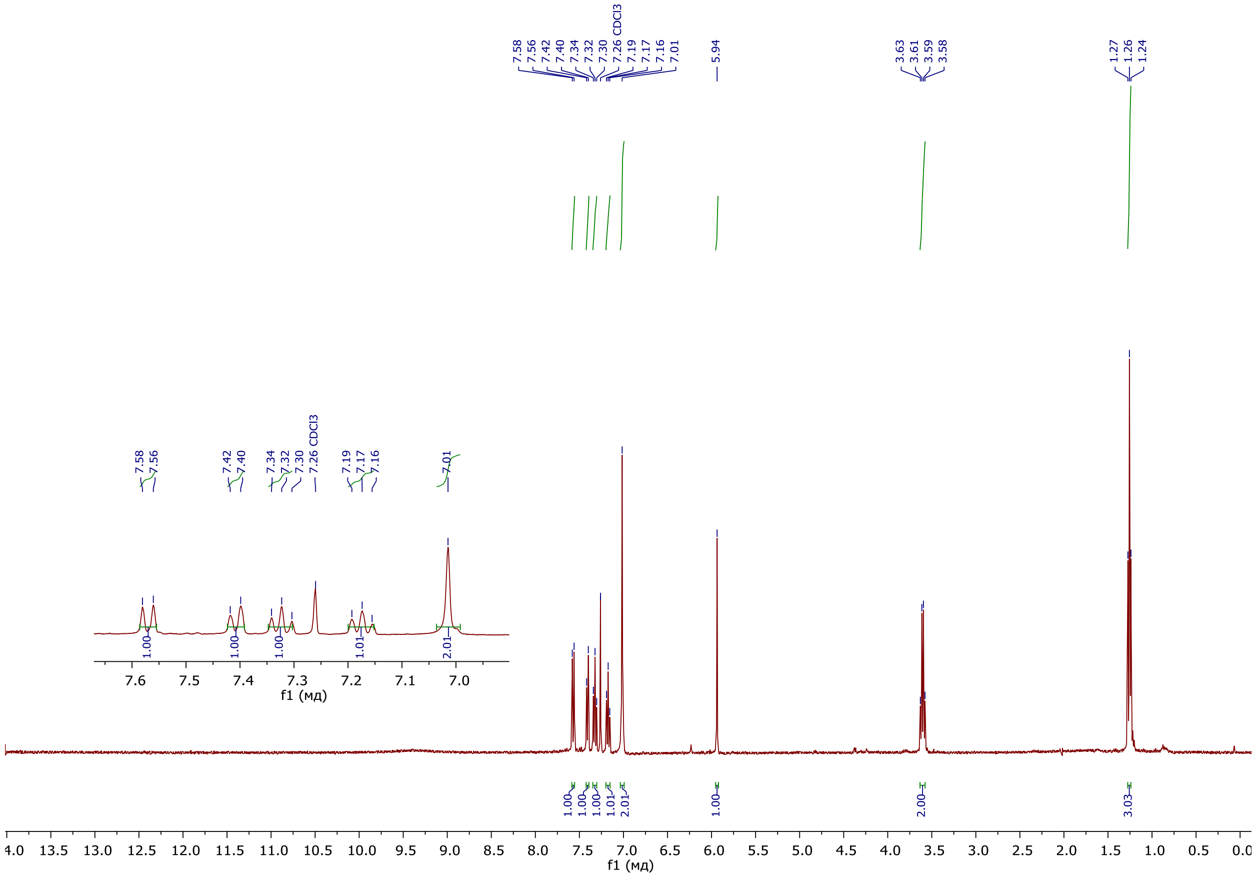

Figure S61. ^{1}H NMR (400 MHz, Chloroform-*d*) spectrum of compound **4h**.

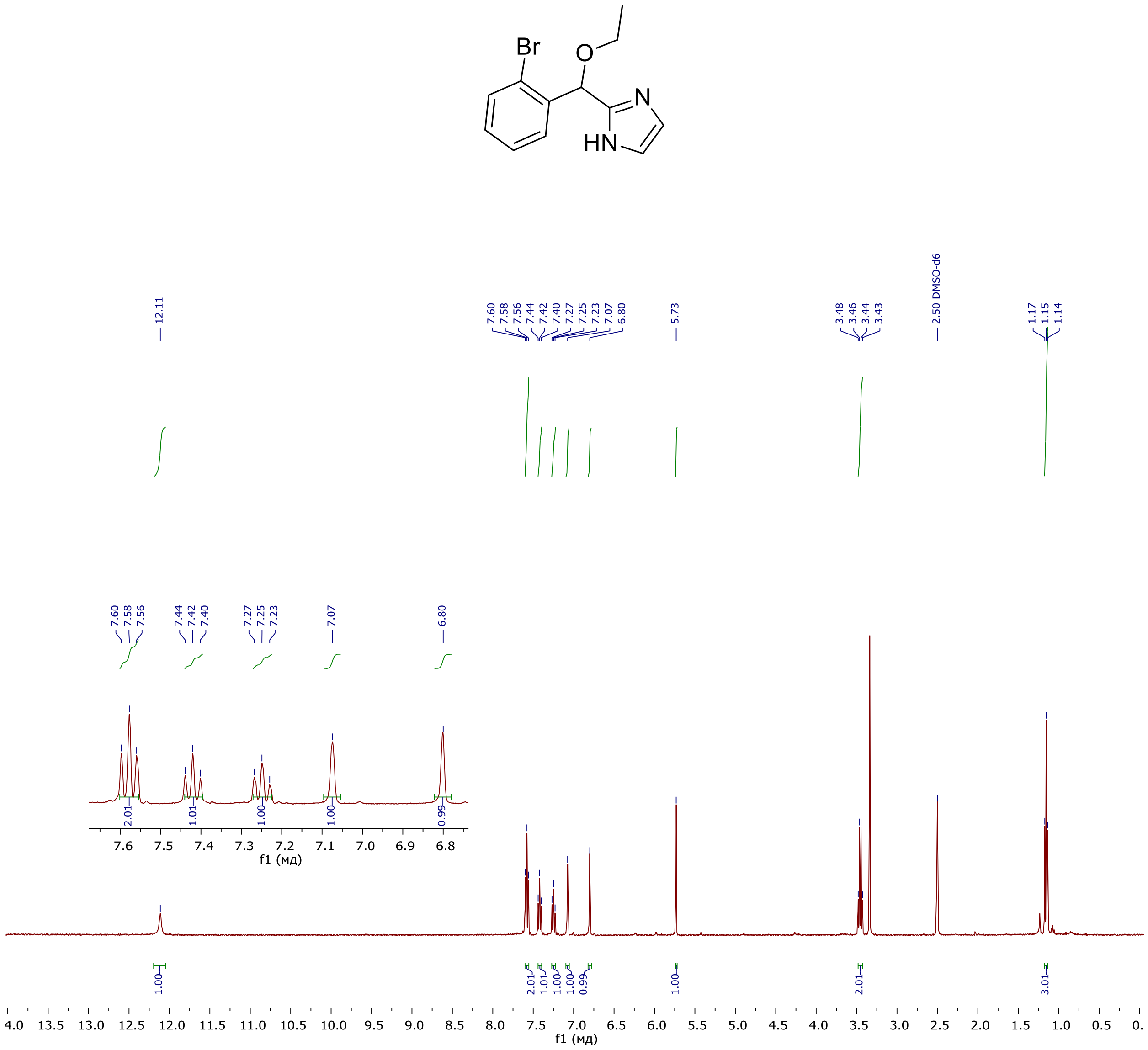

Figure S62. ^{1}H NMR (400 MHz, DMSO-d_6) spectrum of compound **4h**.

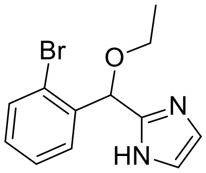

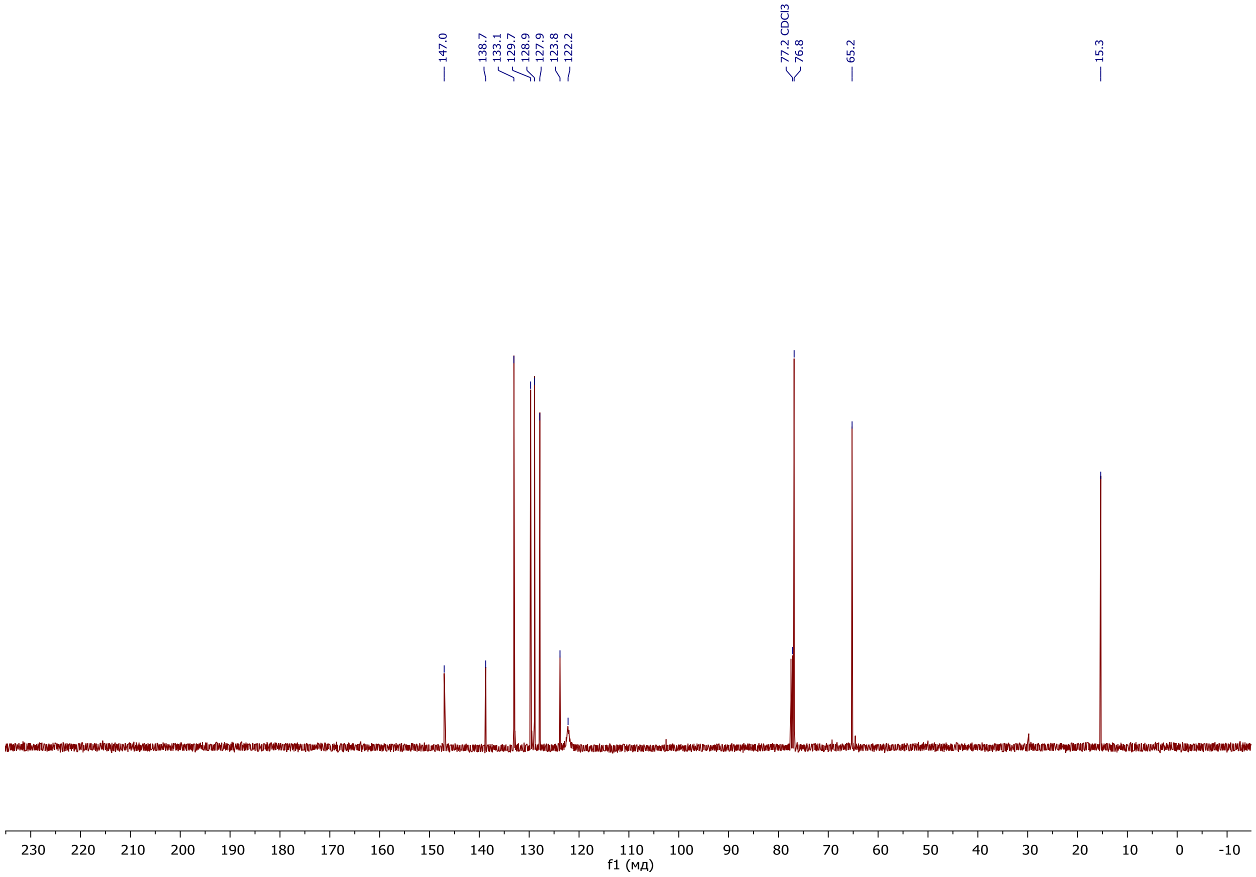

Figure S63. ^{13}C NMR (101 MHz, Chloroform-*d*) spectrum of compound **4h**.

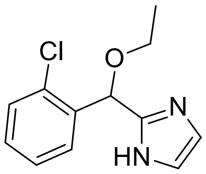

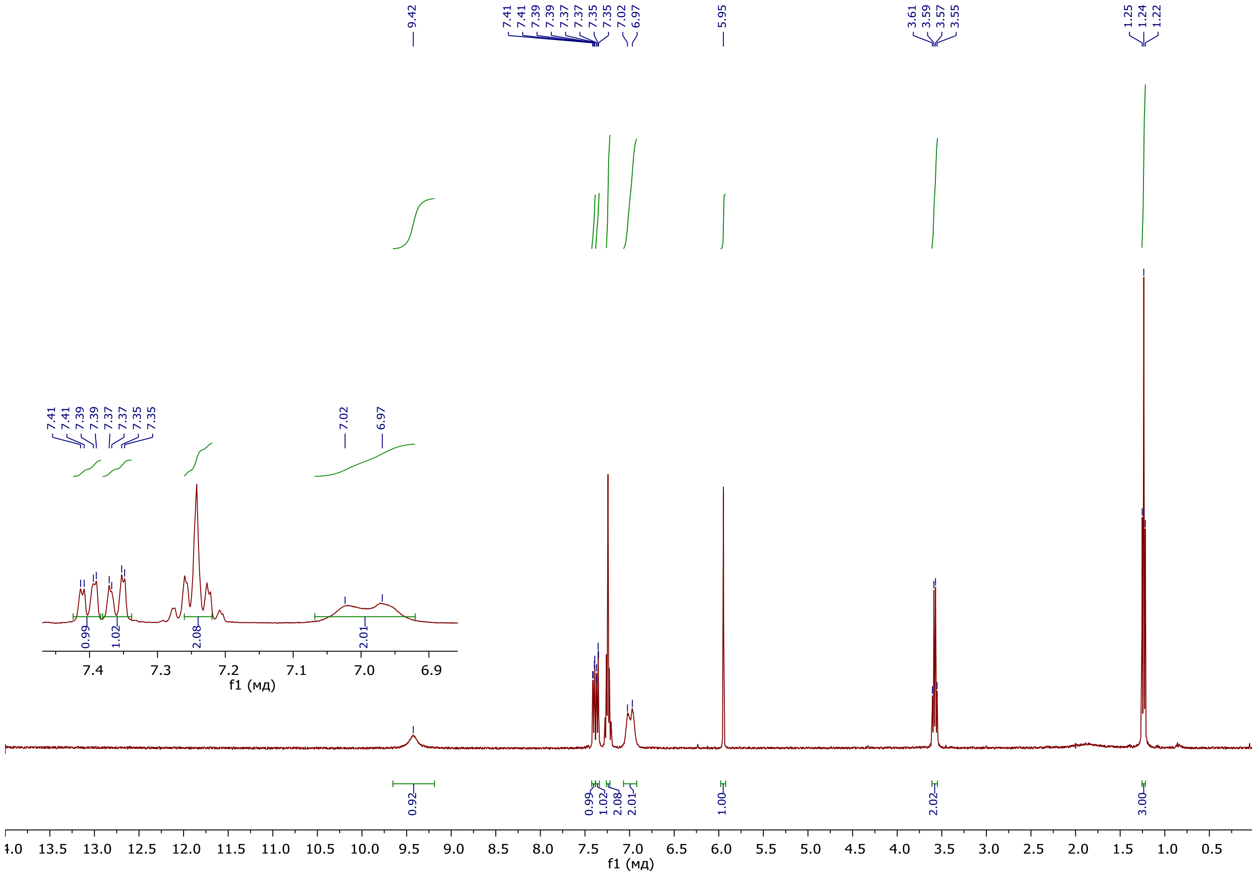

Figure S64. ^{1}H NMR (400 MHz, Chloroform-*d*) spectrum of compound **4i**.

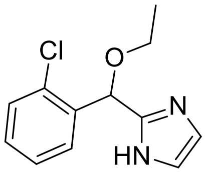

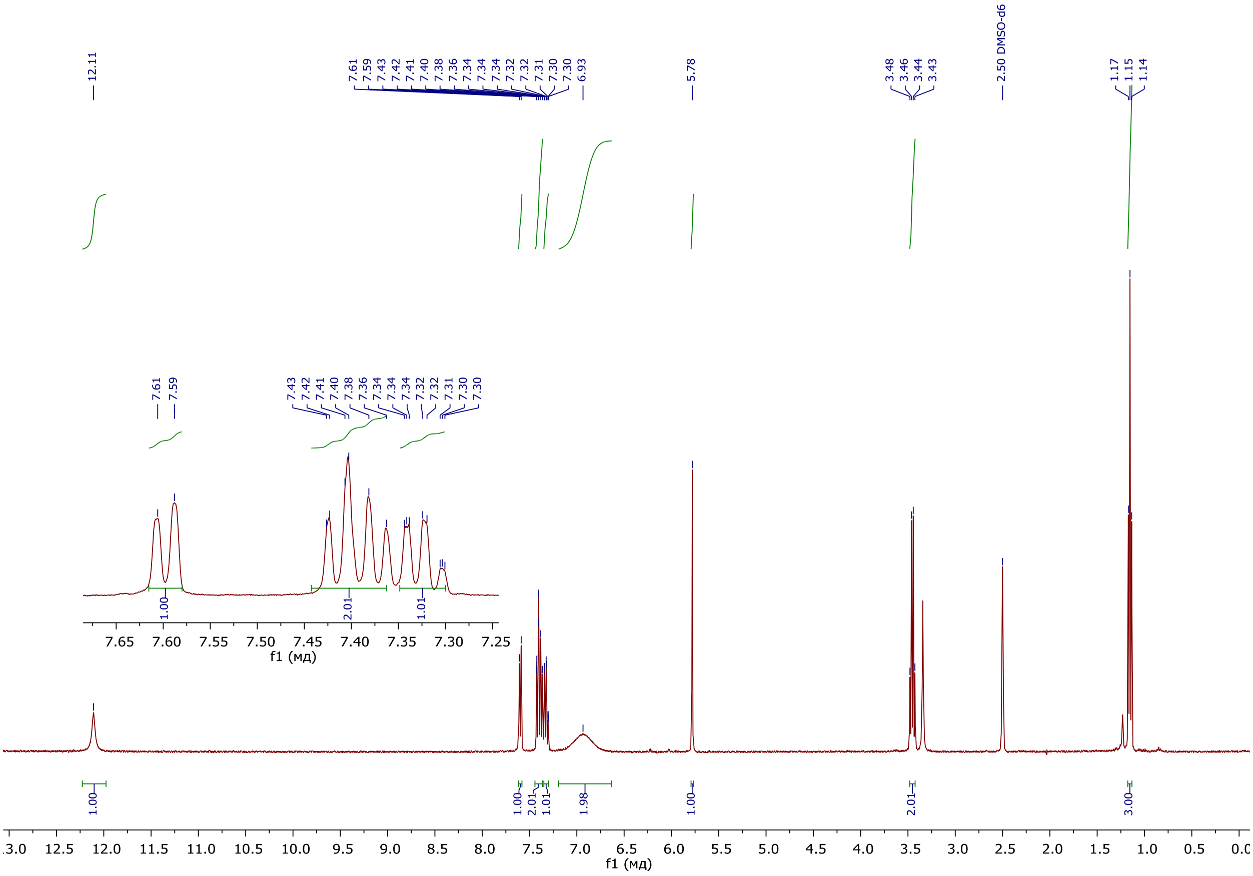

Figure S65. ^{1}H NMR (400 MHz, DMSO-d_6) spectrum of compound **4i**.

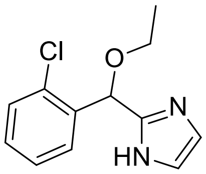

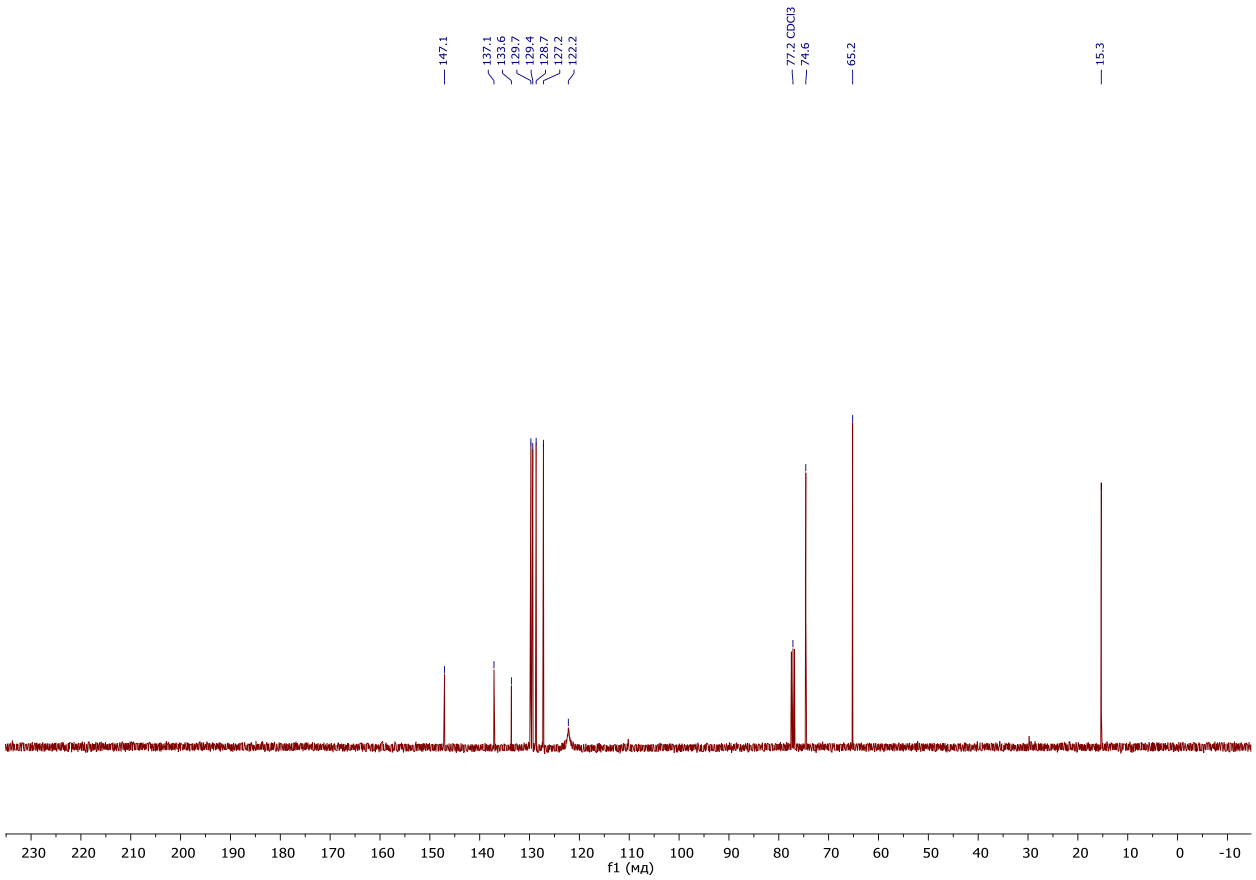

Figure S66. ^{13}C NMR (101 MHz, Chloroform-*d*) spectrum of compound **4i**.

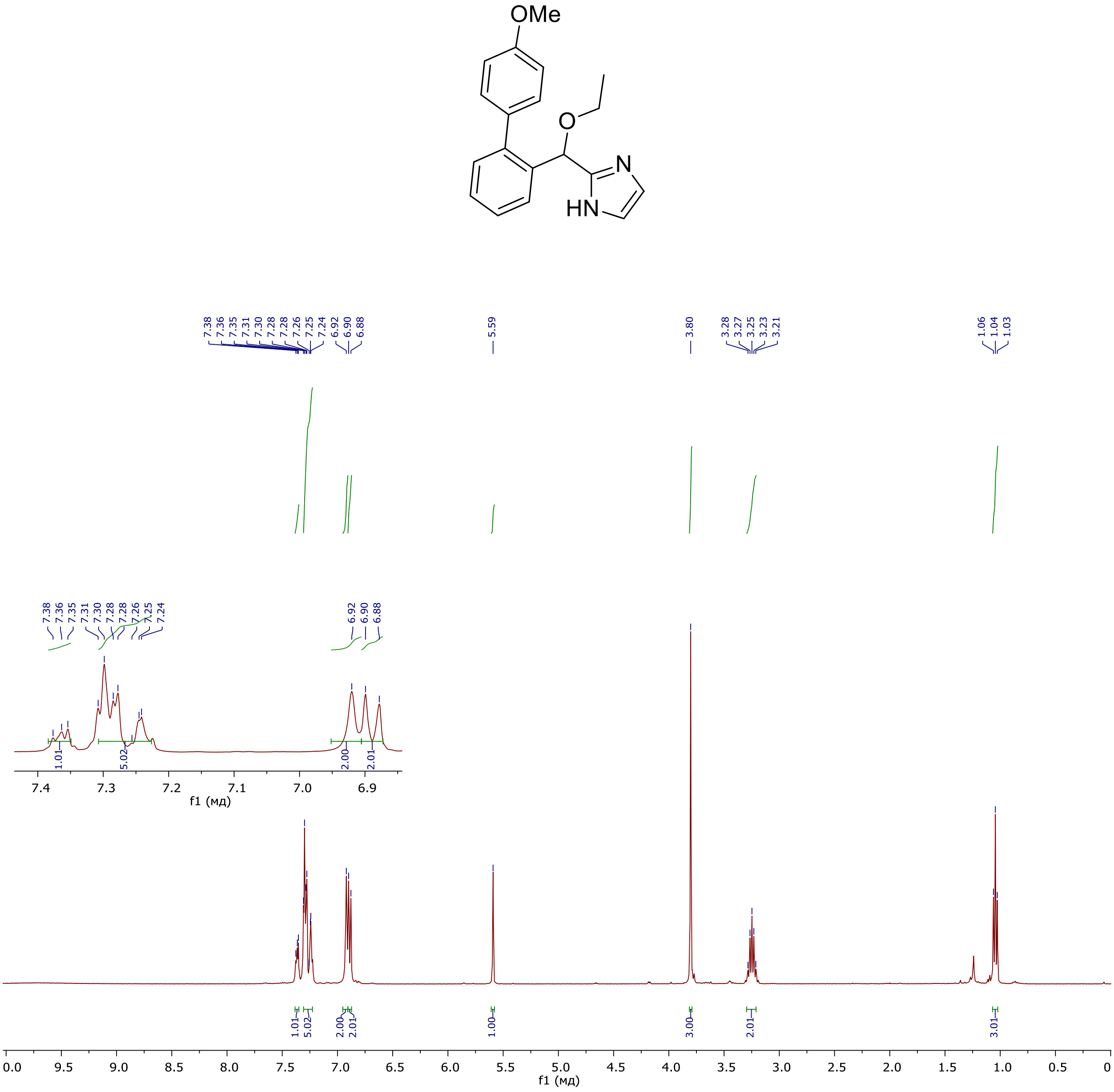

Figure S67. ^{1}H NMR (400 MHz, Chloroform-*d*) spectrum of compound **4j**.

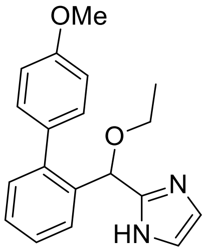

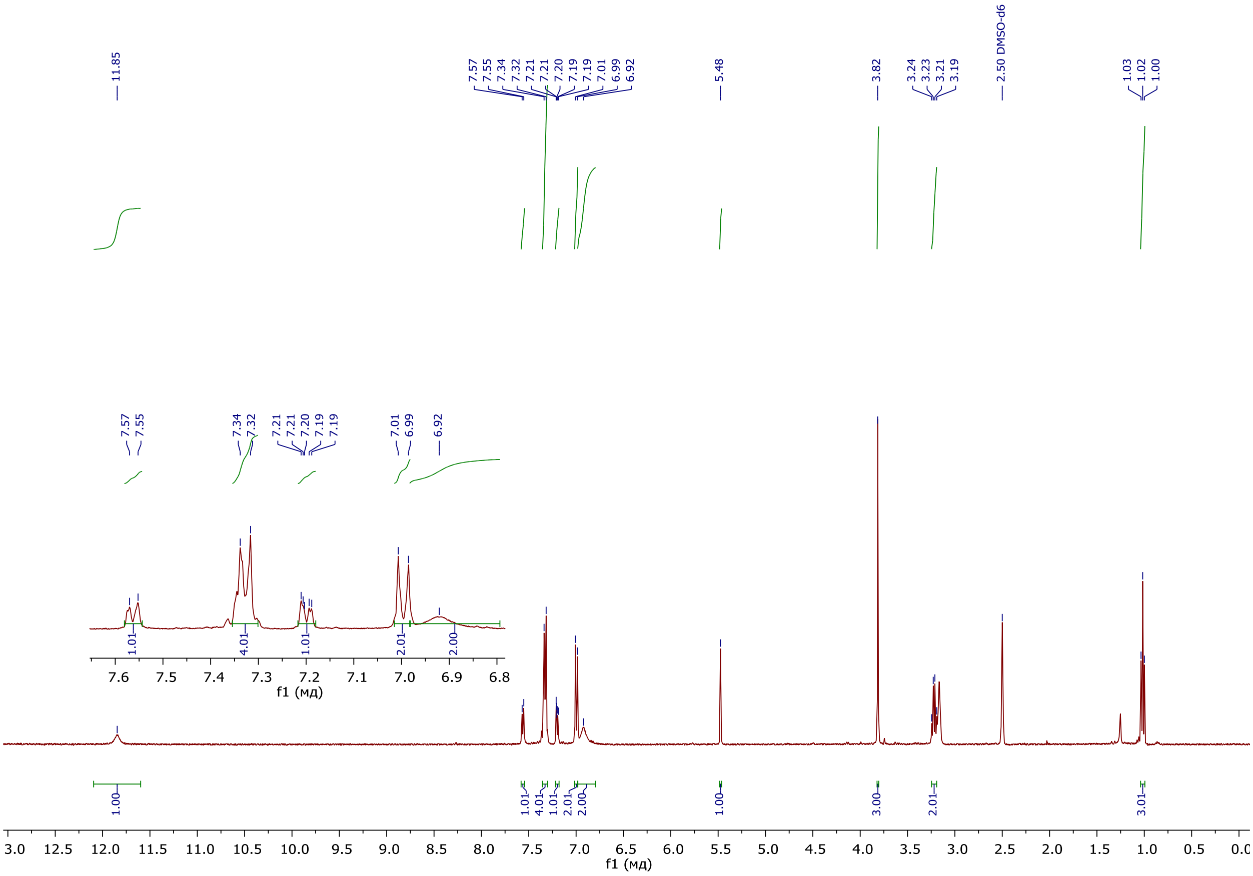

Figure S68. ^{1}H NMR (400 MHz, DMSO-d_6) spectrum of compound **4j**.

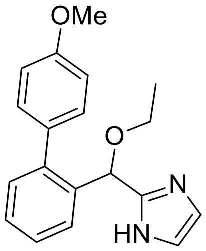

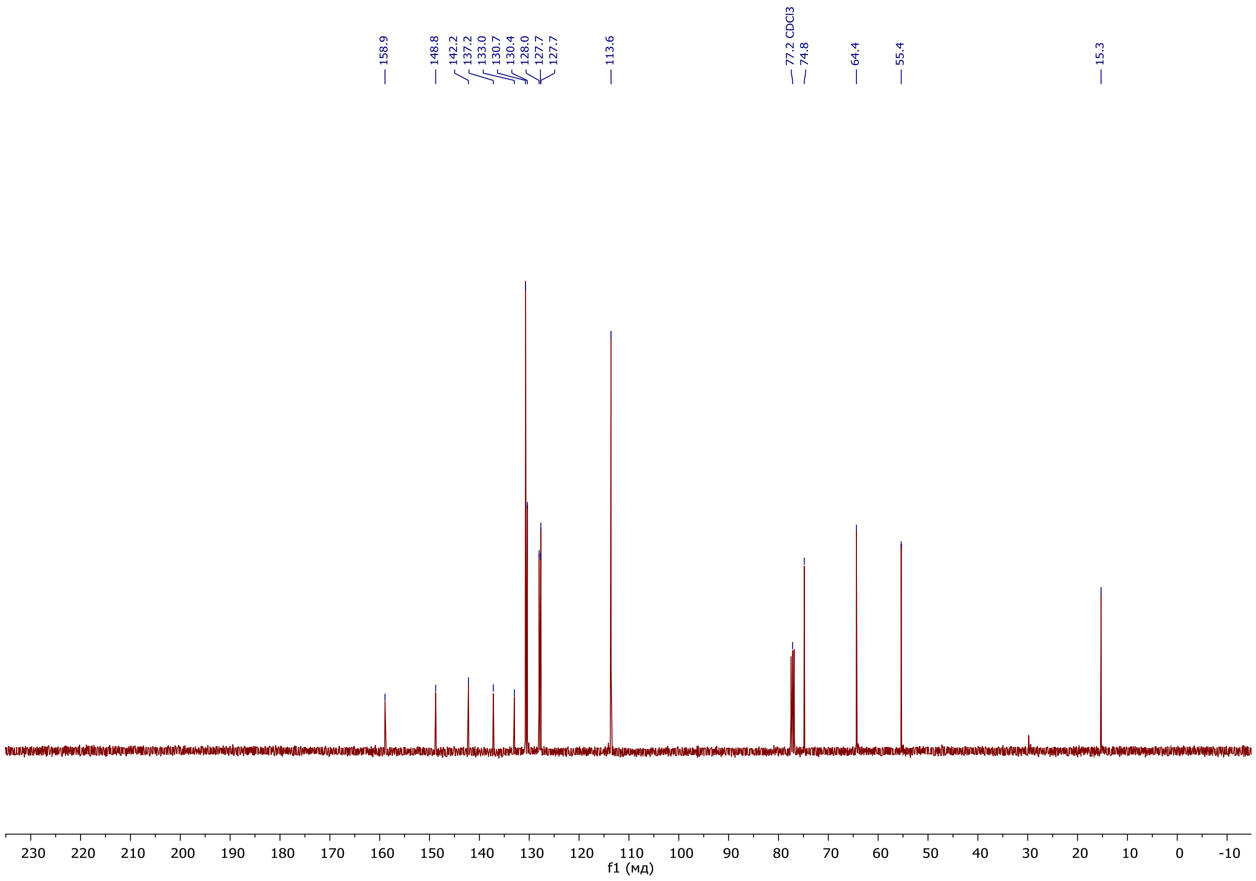

Figure S69. ^{13}C NMR (101 MHz, Chloroform-*d*) spectrum of compound **4j**.

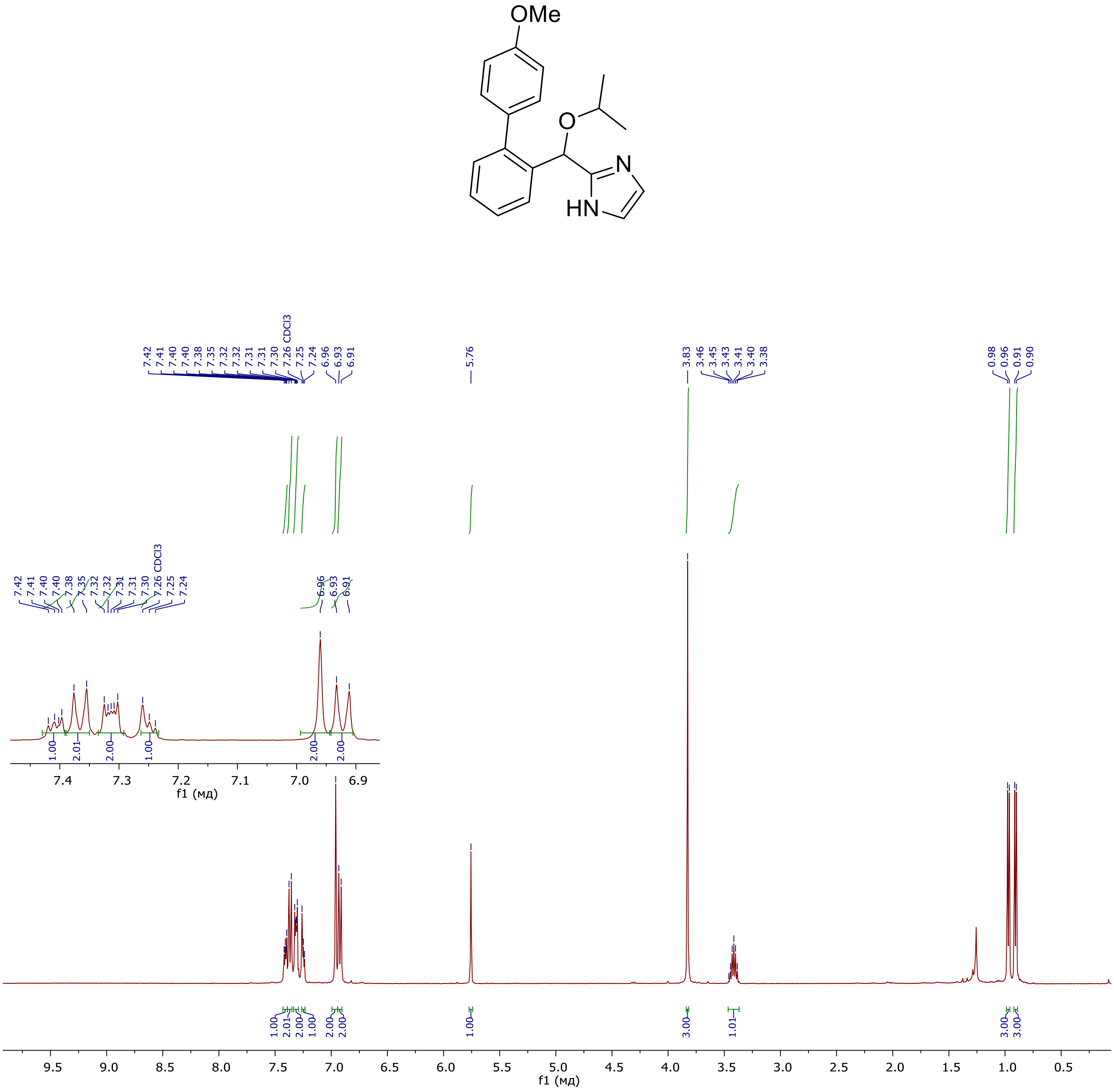

Figure S70. ^{1}H NMR (400 MHz, Chloroform-*d*) spectrum of compound **4k**.

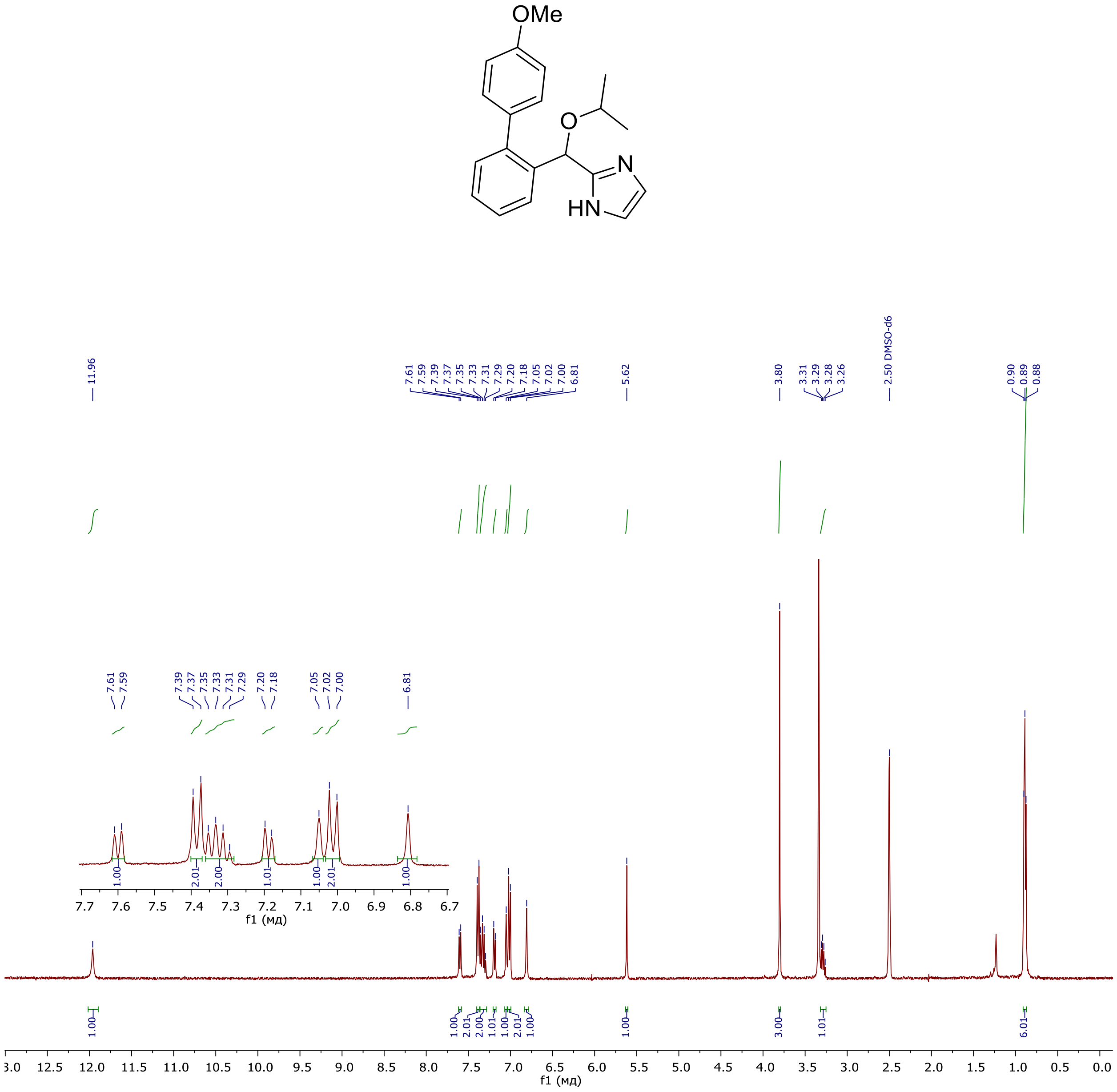

Figure S71. ^{1}H NMR (400 MHz, DMSO-d_6) spectrum of compound **4k**.

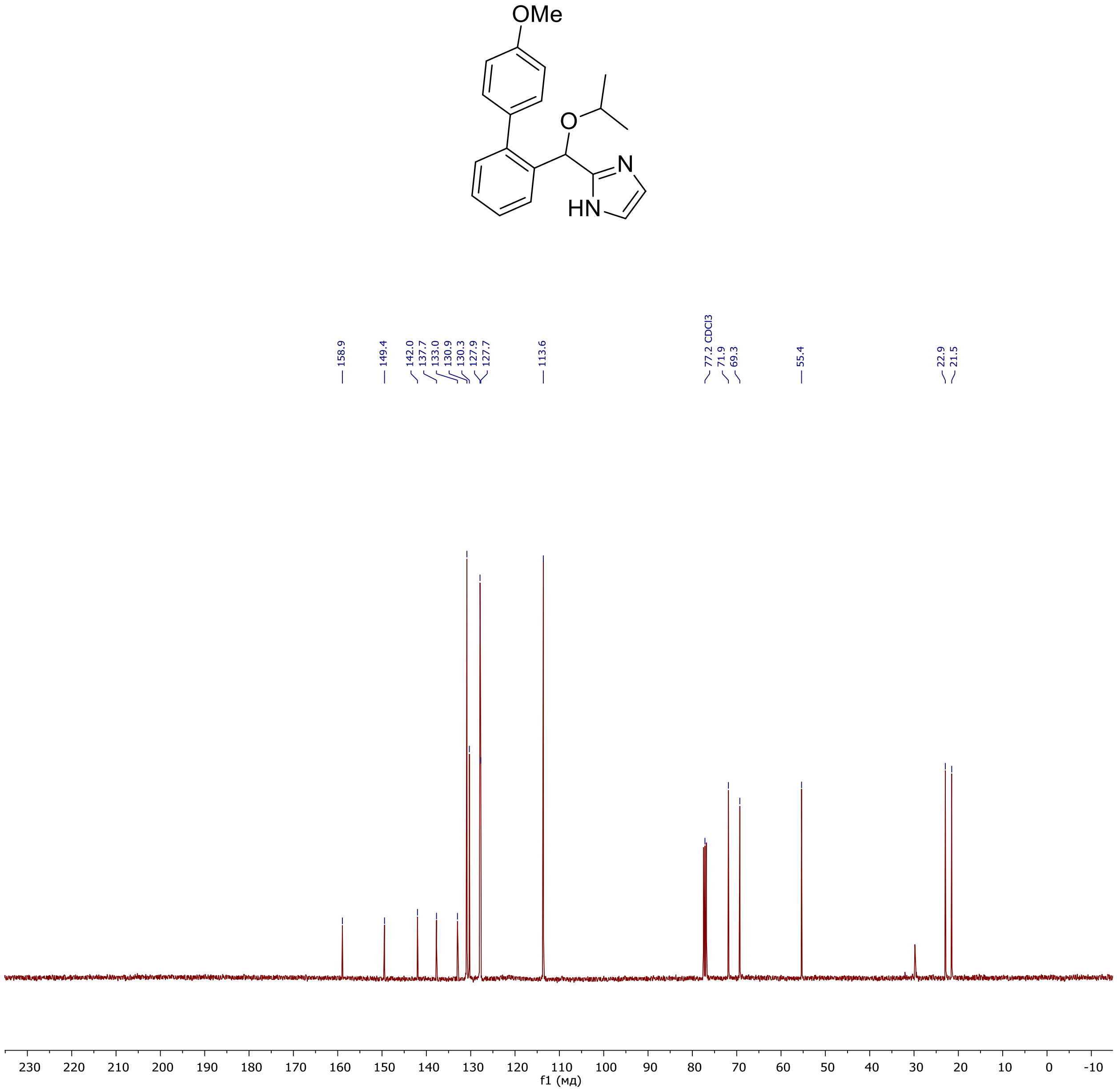

Figure S72. ^{13}C NMR (101 MHz, Chloroform-*d*) spectrum of compound **4k**.

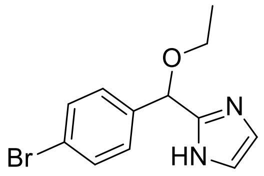

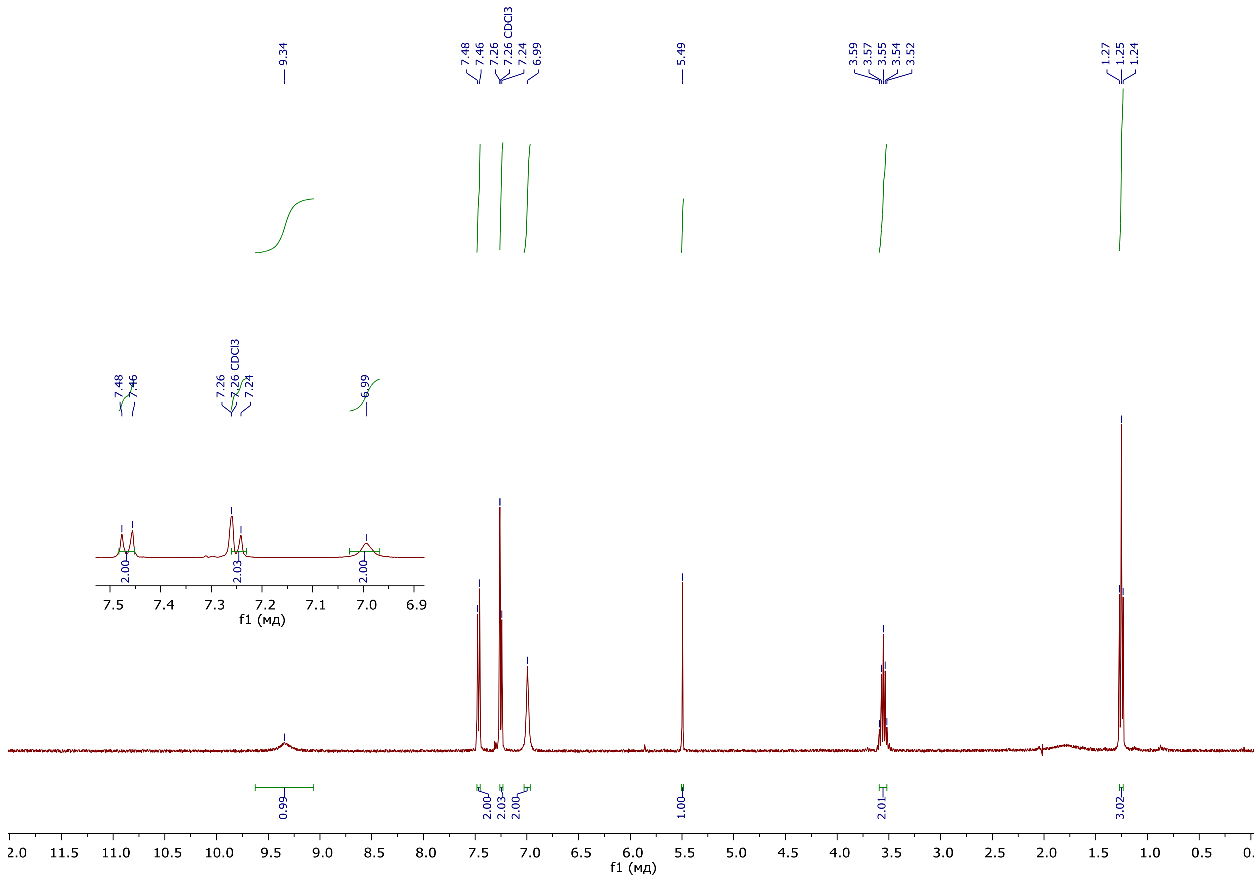

Figure S73. ^{1}H NMR (400 MHz, Chloroform-*d*) spectrum of compound **4l**.

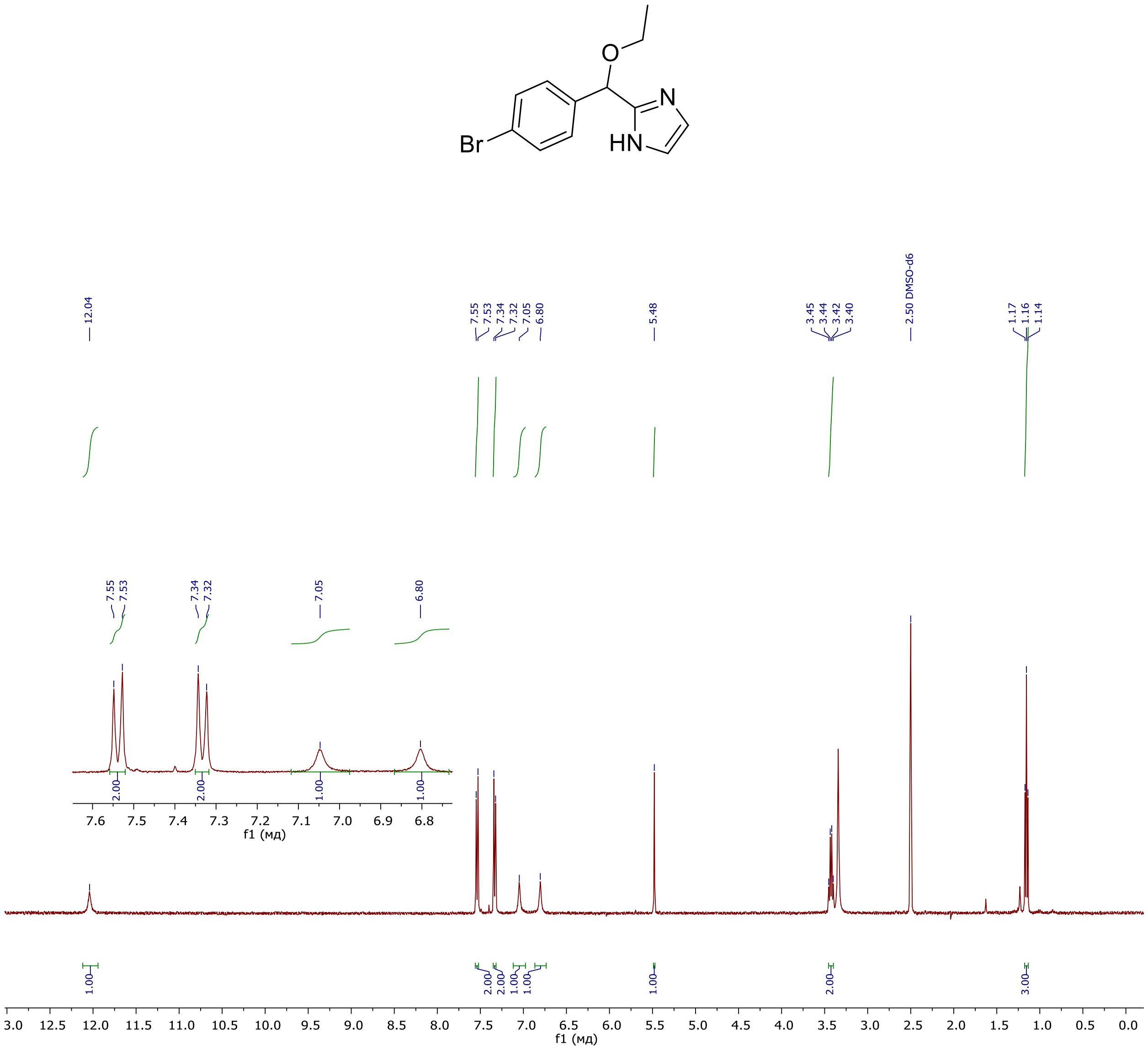

Figure S74. ^{1}H NMR (400 MHz, DMSO-d_6) spectrum of compound **4l**.

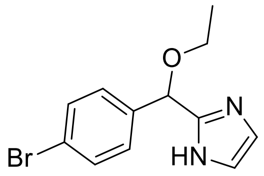

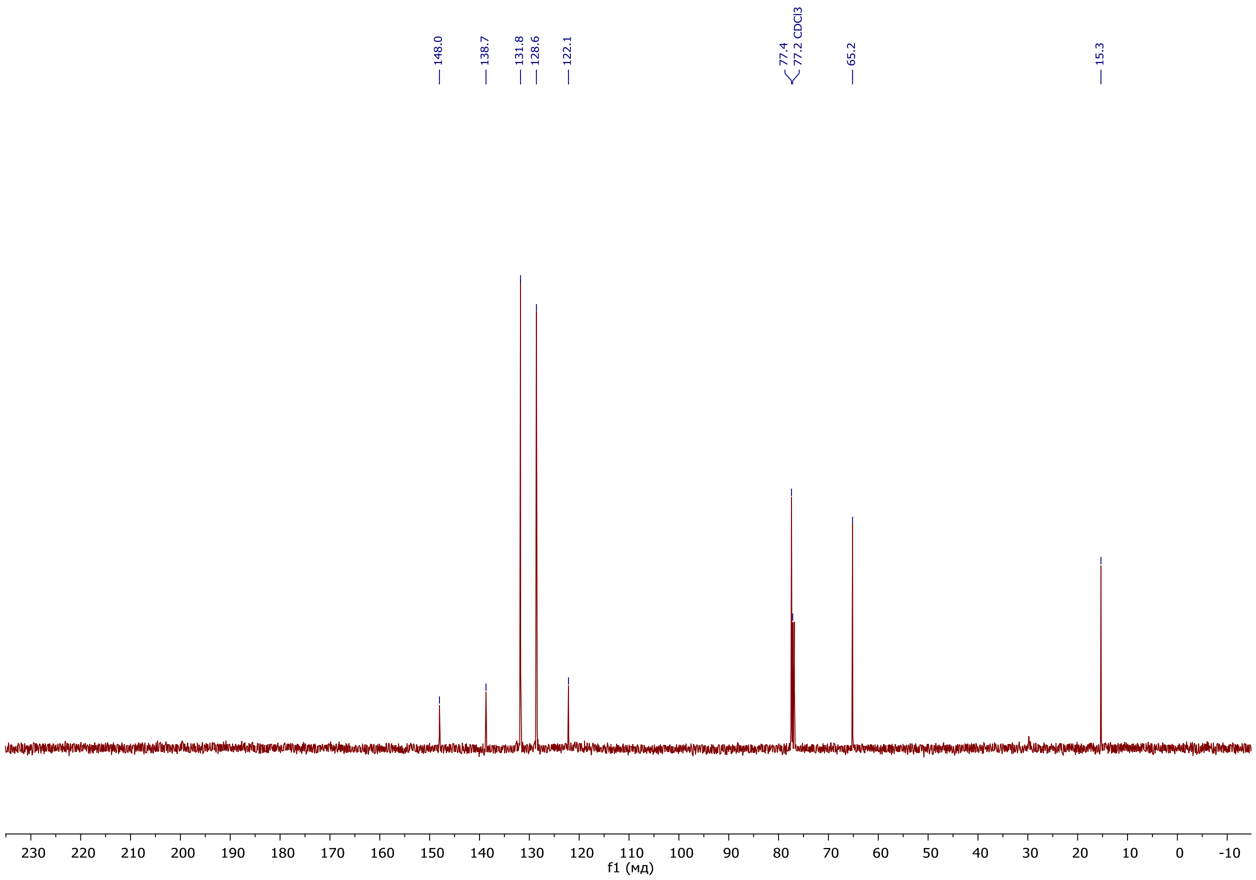

Figure S75. ^{13}C NMR (101 MHz, Chloroform-*d*) spectrum of compound **4l**.

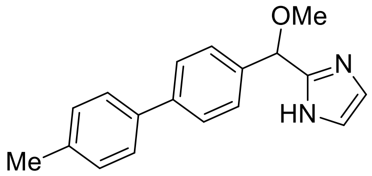

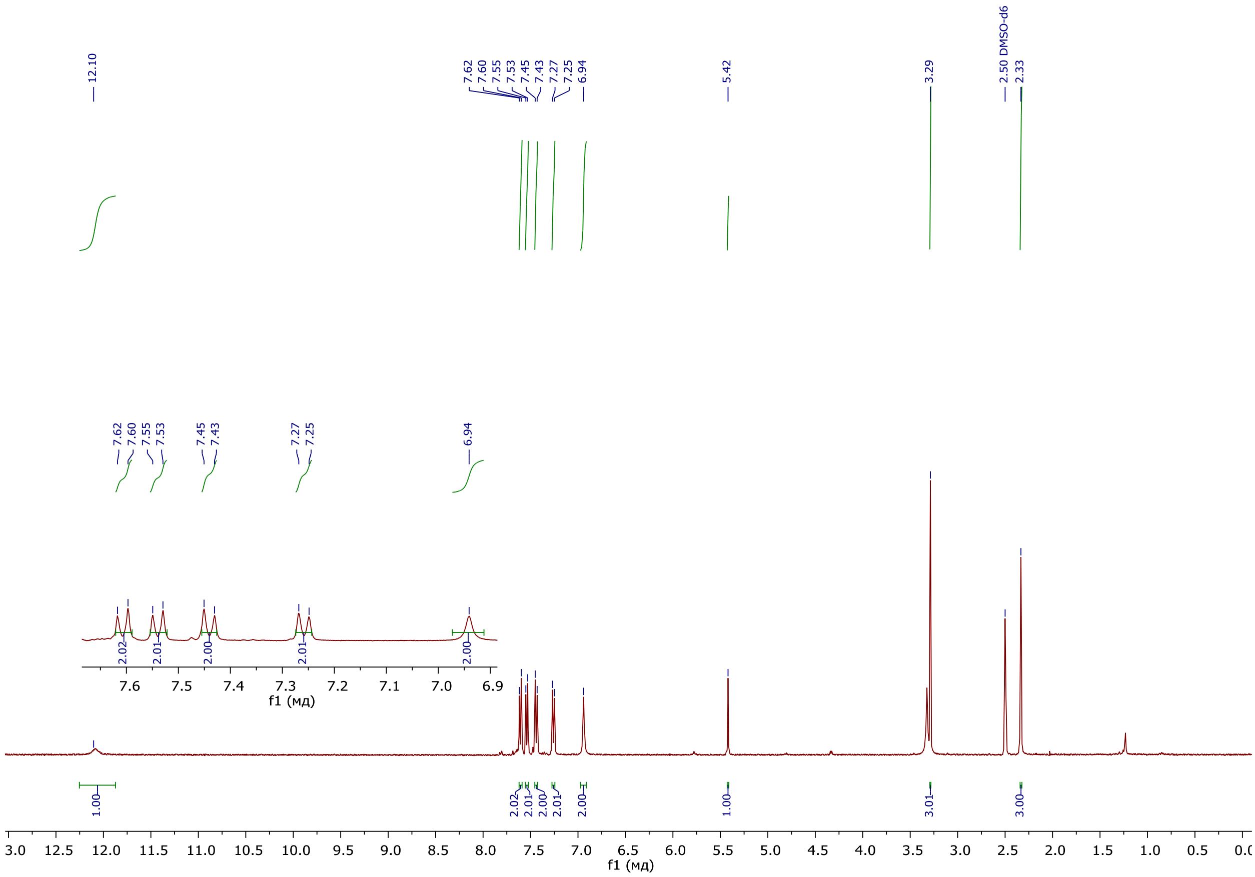

Figure S76. ^{1}H NMR (400 MHz, DMSO-d_6) spectrum of compound **4m**.

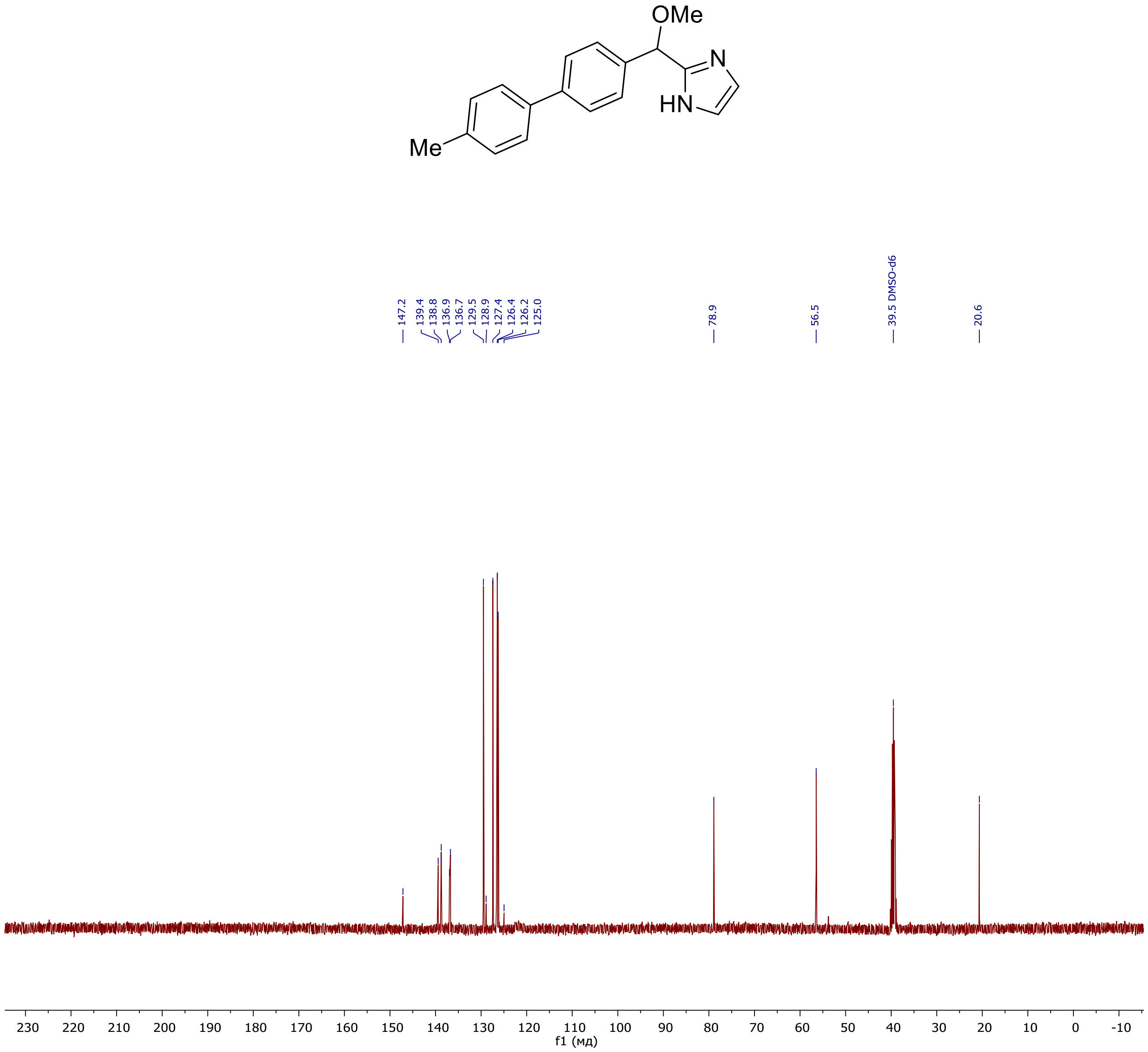

Figure S77. ^{13}C NMR (101 MHz, DMSO-d_6) spectrum of compound **4m**.

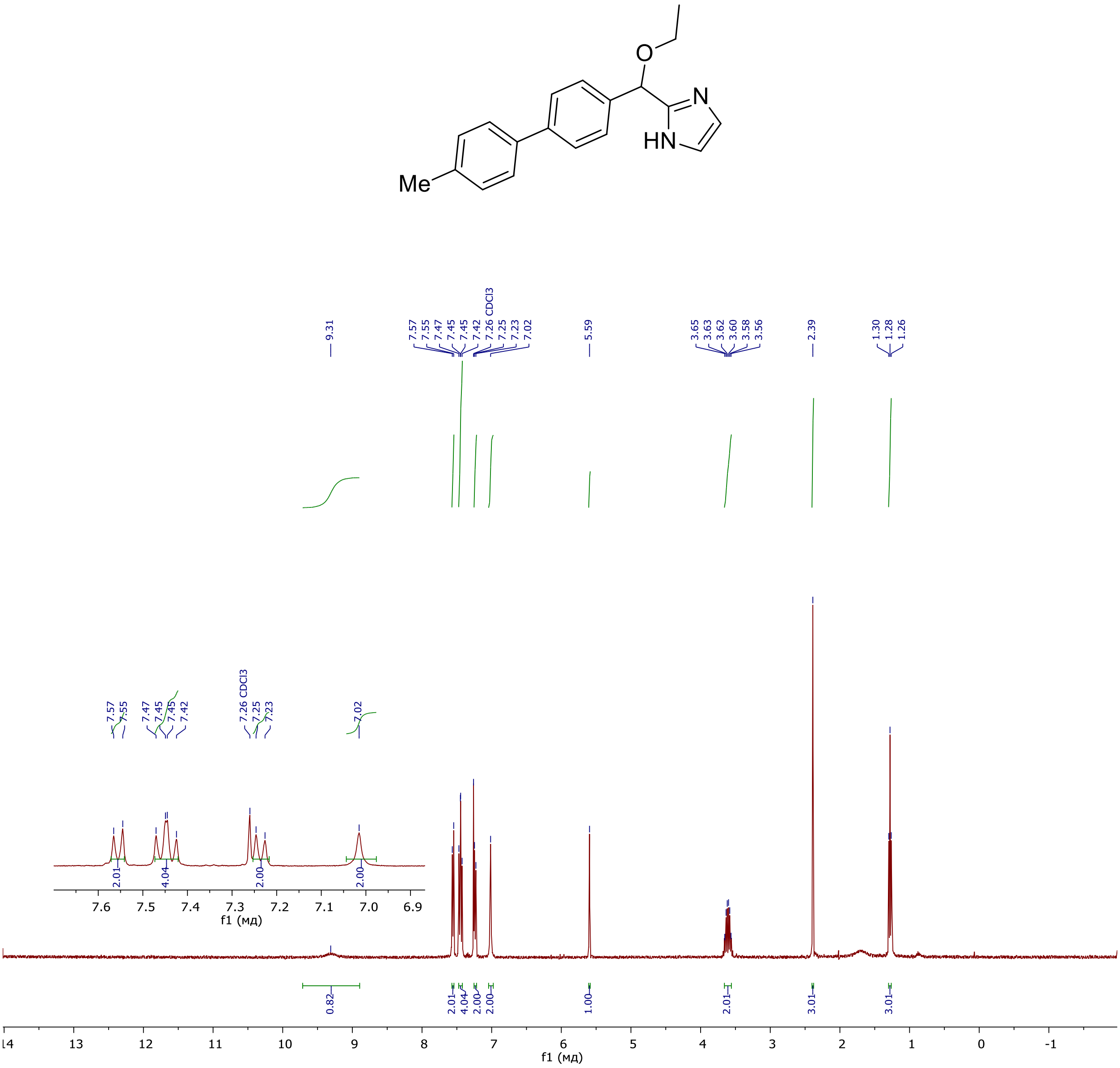

Figure S78. ^{1}H NMR (400 MHz, Chloroform-*d*) spectrum of compound **4n**.

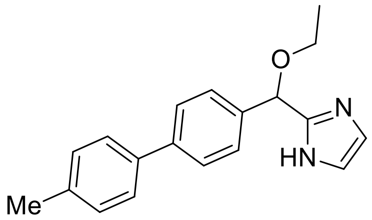

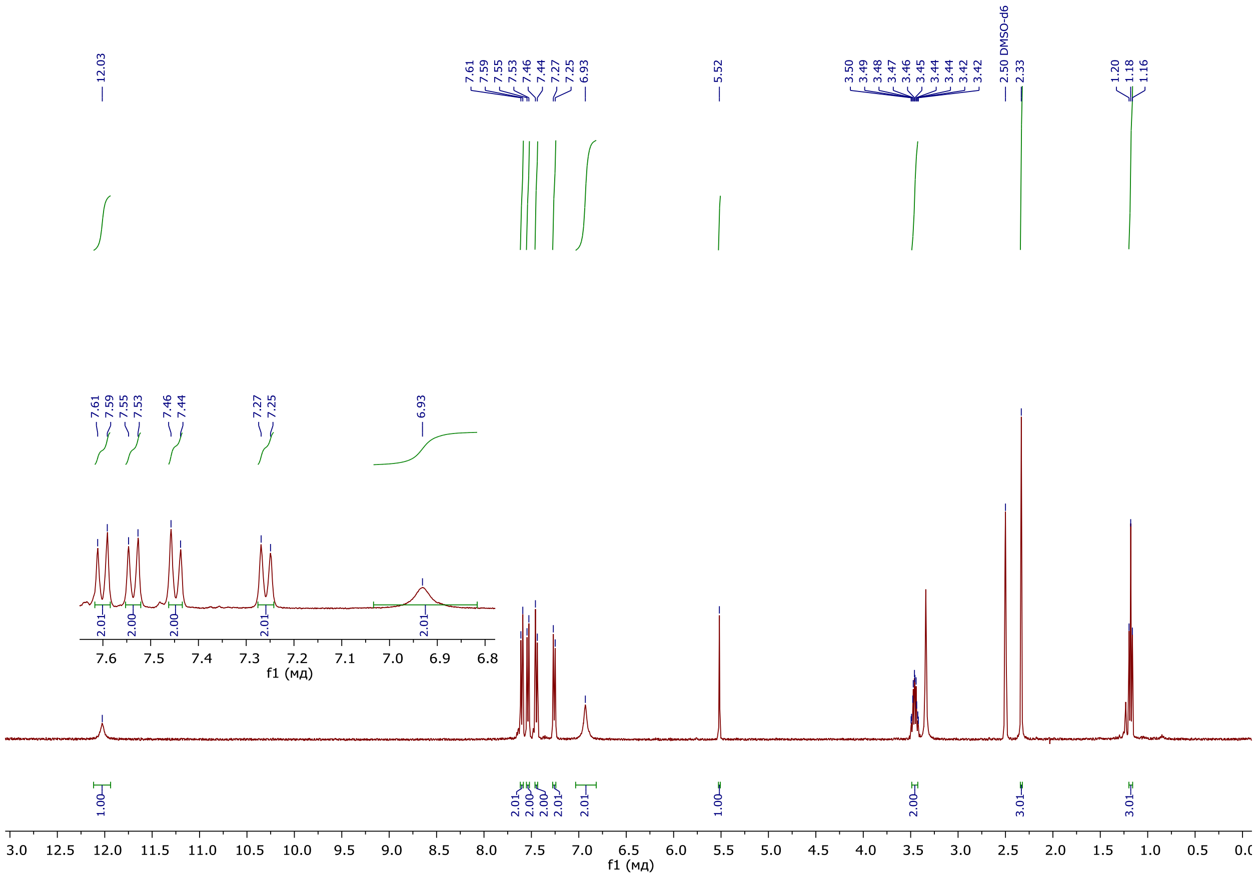

Figure S79. ^{1}H NMR (400 MHz, DMSO-d_6) spectrum of compound **4n**.

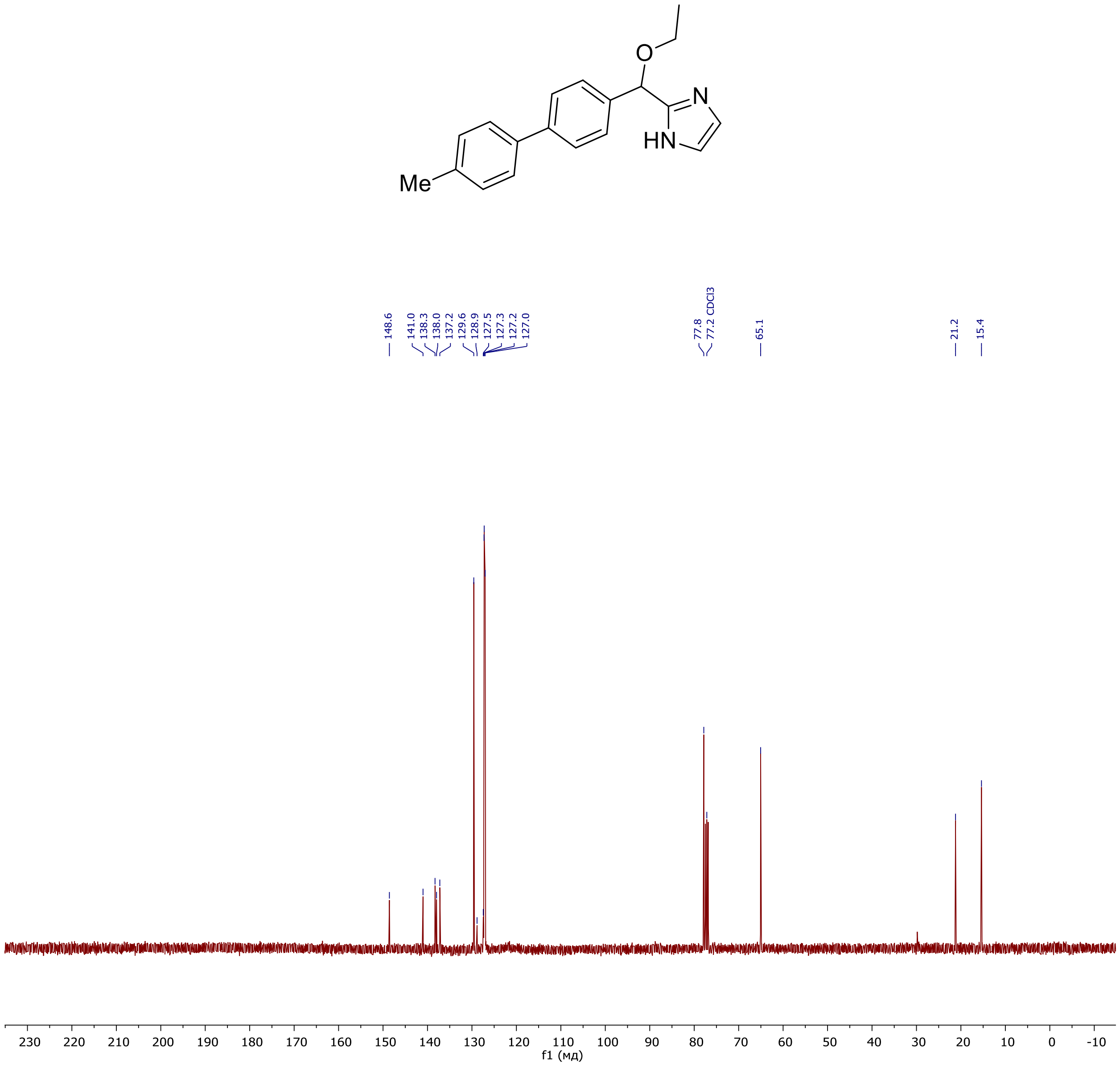

Figure S80. ^{13}C NMR (101 MHz, Chloroform-*d*) spectrum of compound **4n**.

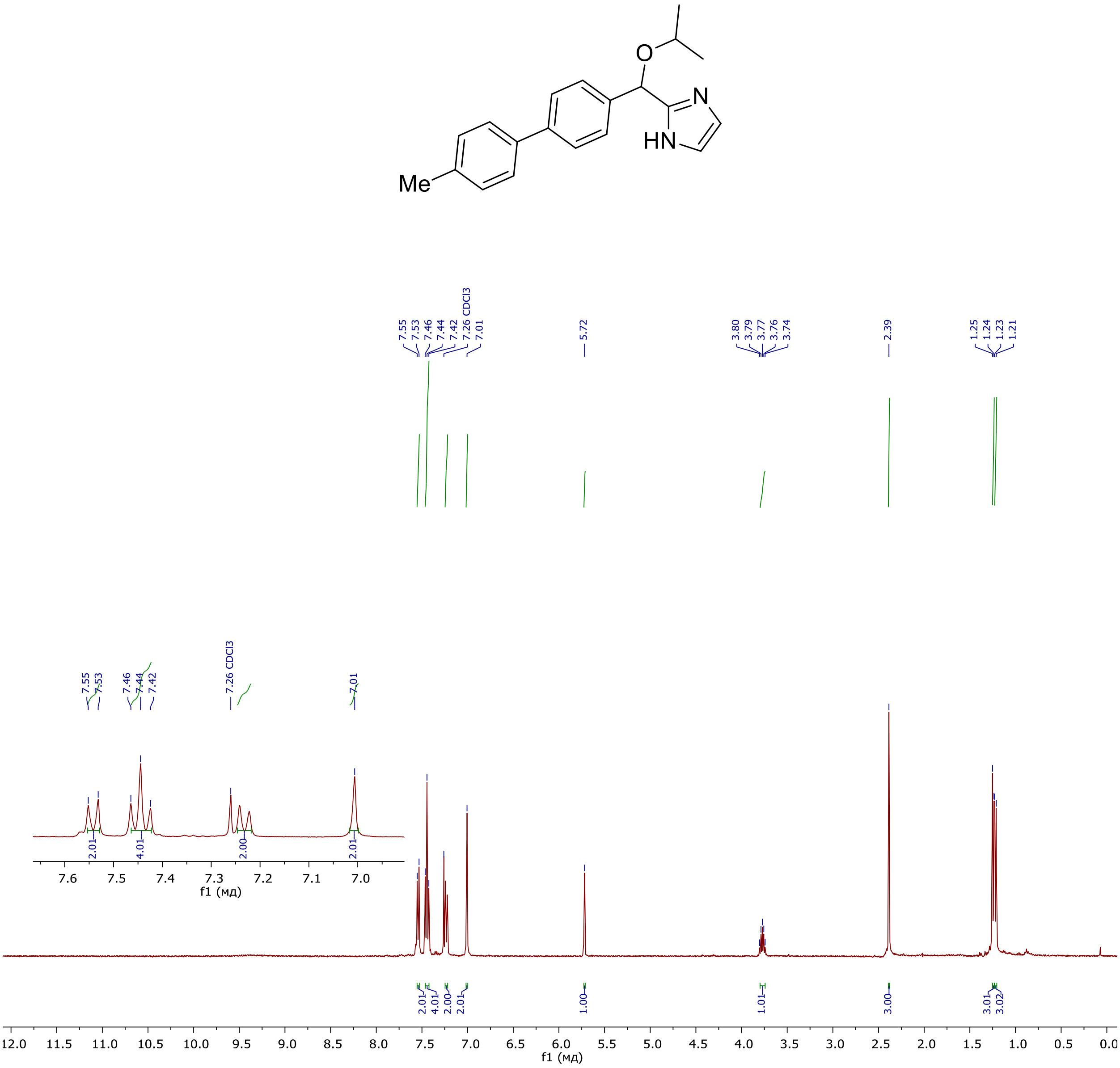

Figure S81. ^{1}H NMR (400 MHz, Chloroform-*d*) spectrum of compound **4o**.

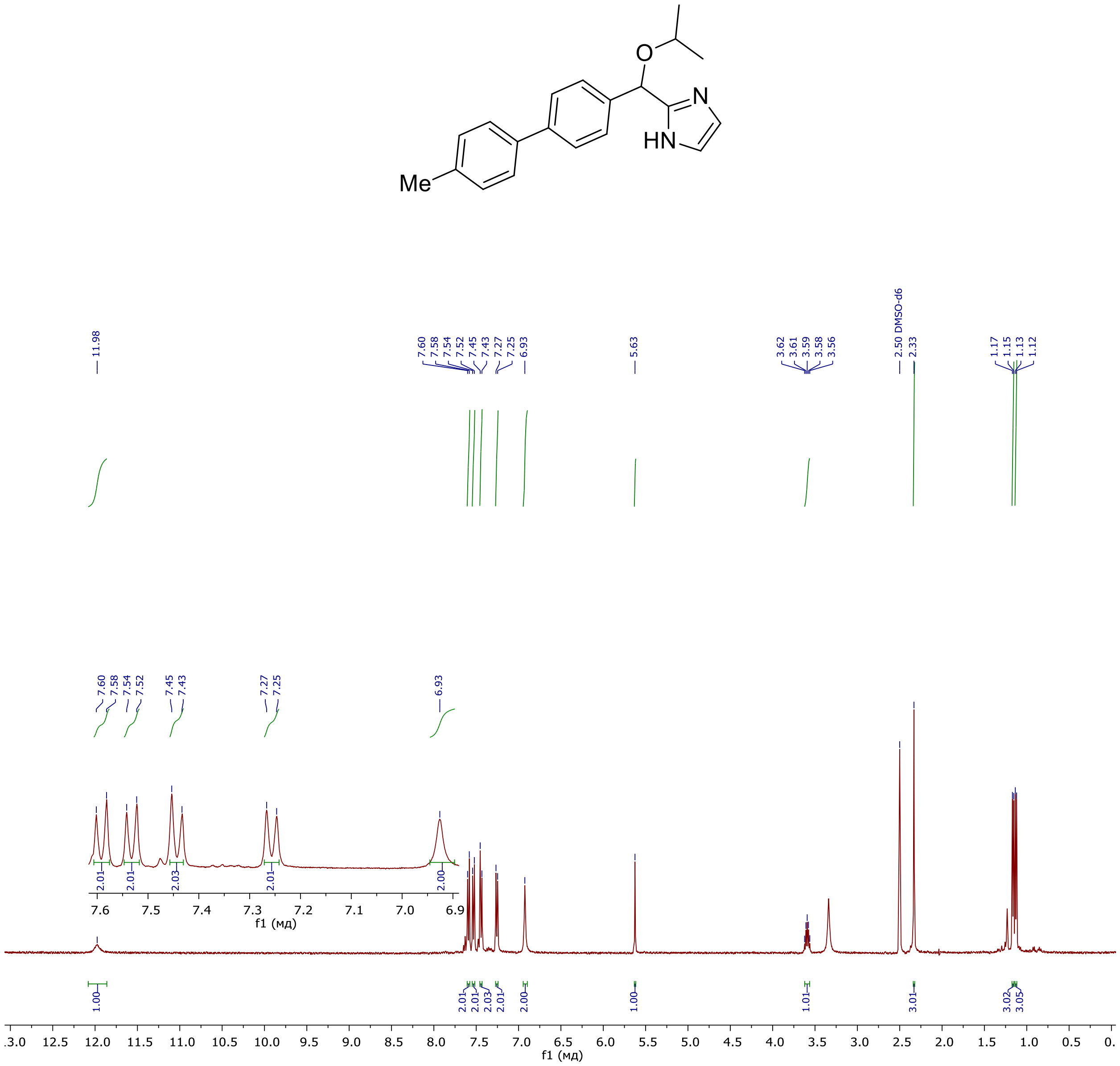

Figure S82. ^{1}H NMR (400 MHz, DMSO-d_6) spectrum of compound **4o**.

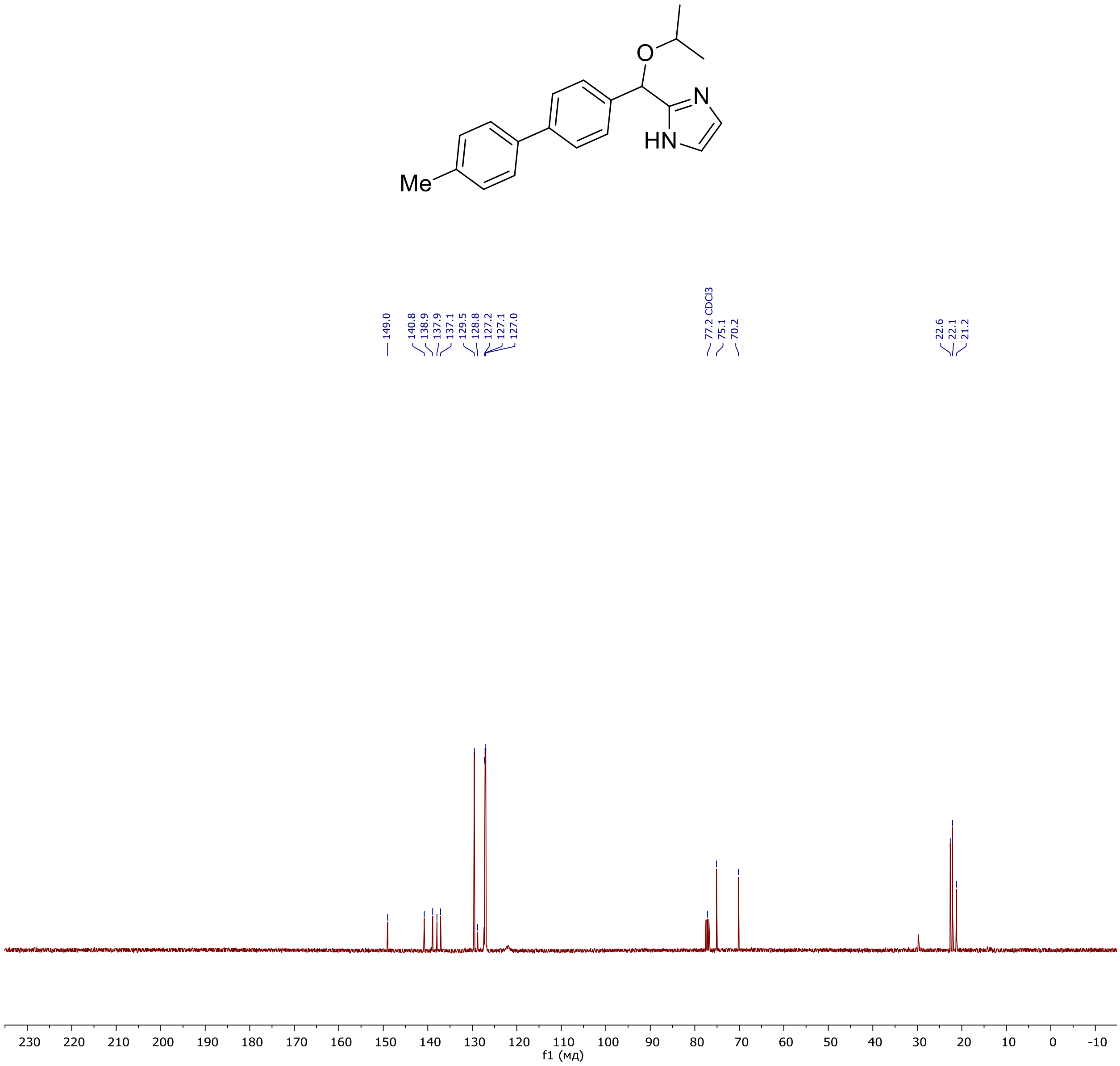

Figure S83. ^{13}C NMR (101 MHz, Chloroform-*d*) spectrum of compound **4o**.

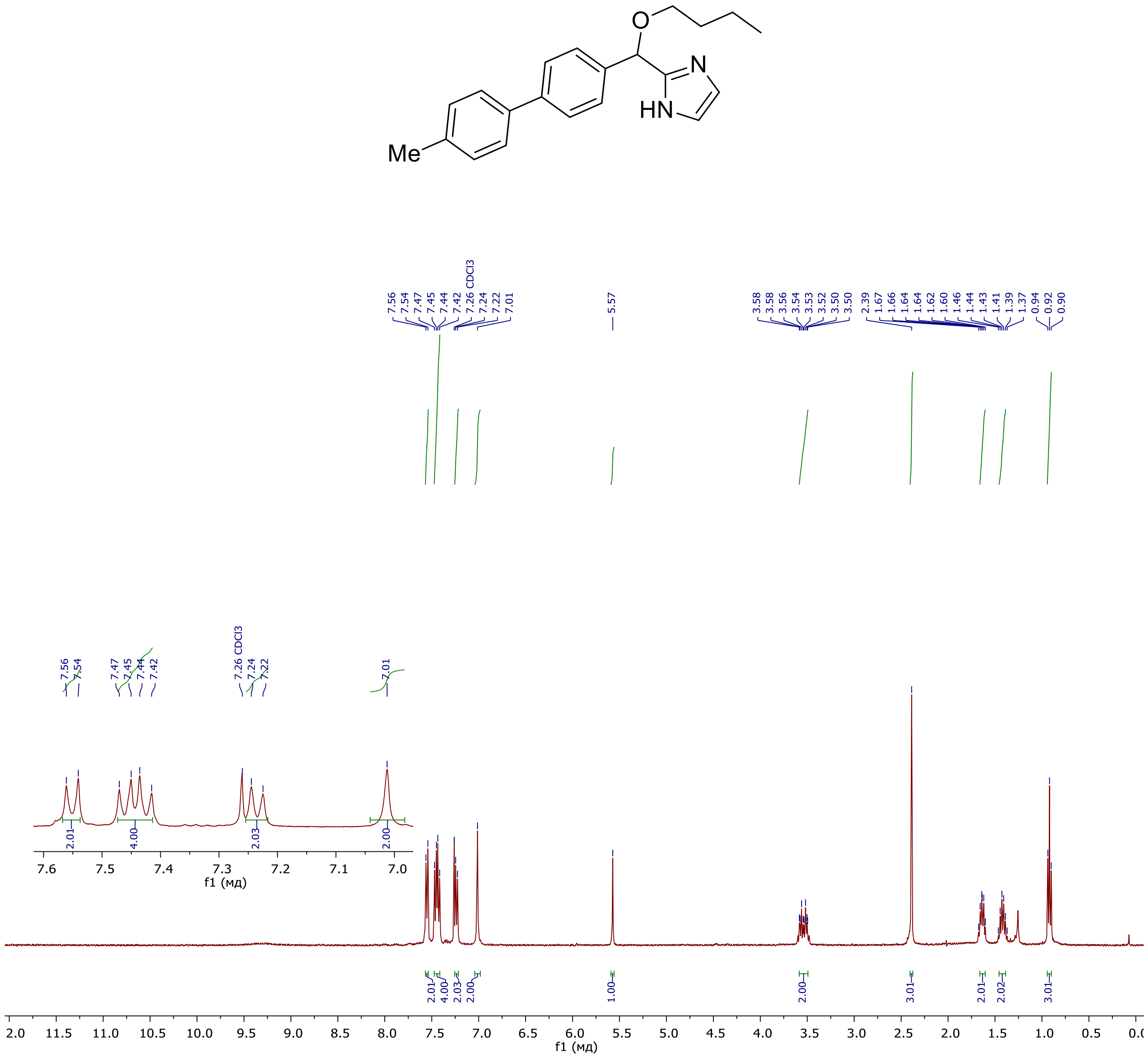

Figure S84. ^{1}H NMR (400 MHz, Chloroform-*d*) spectrum of compound **4p**.

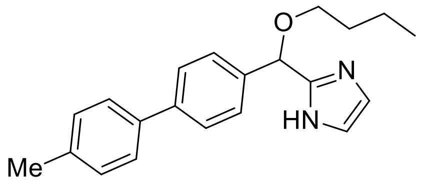

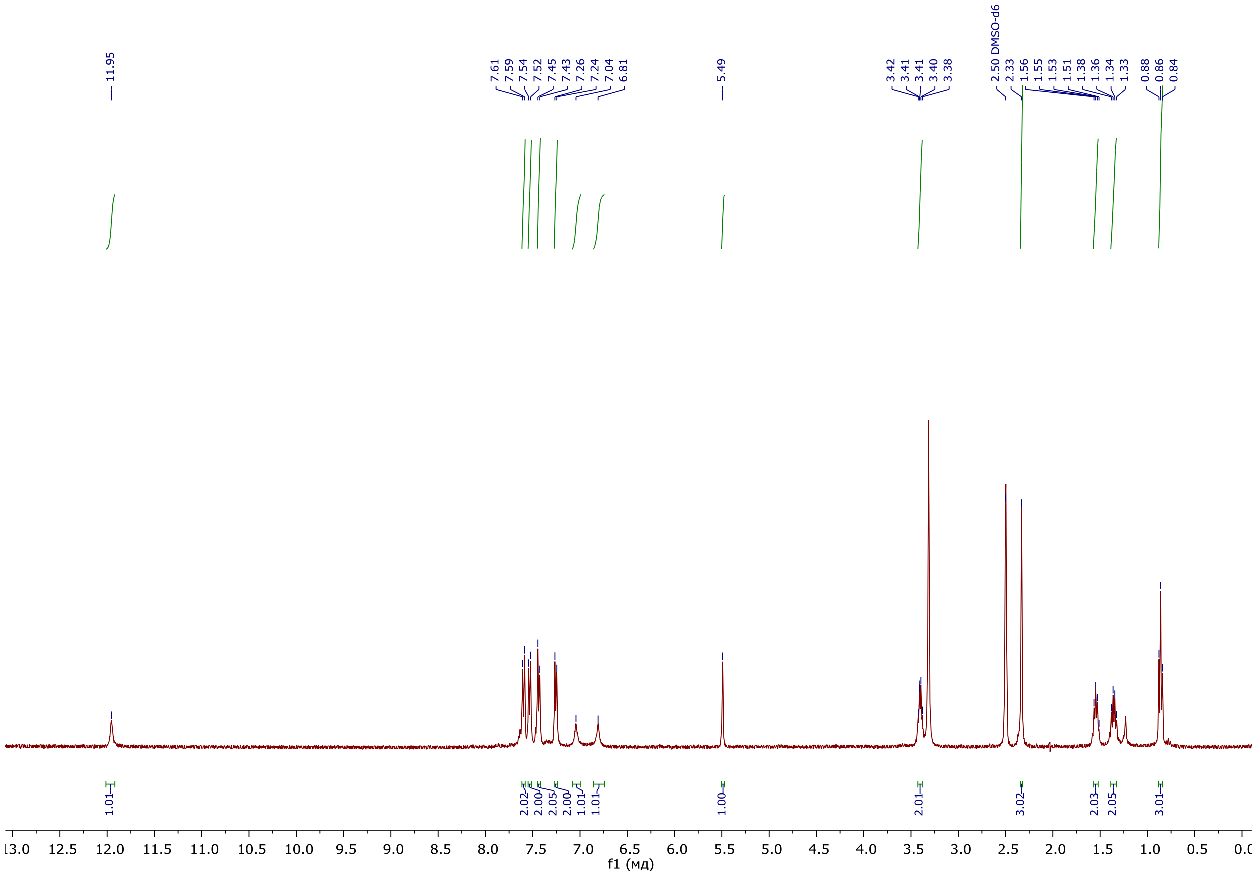

Figure S85. ^{1}H NMR (400 MHz, DMSO-d_6) spectrum of compound **4p.**

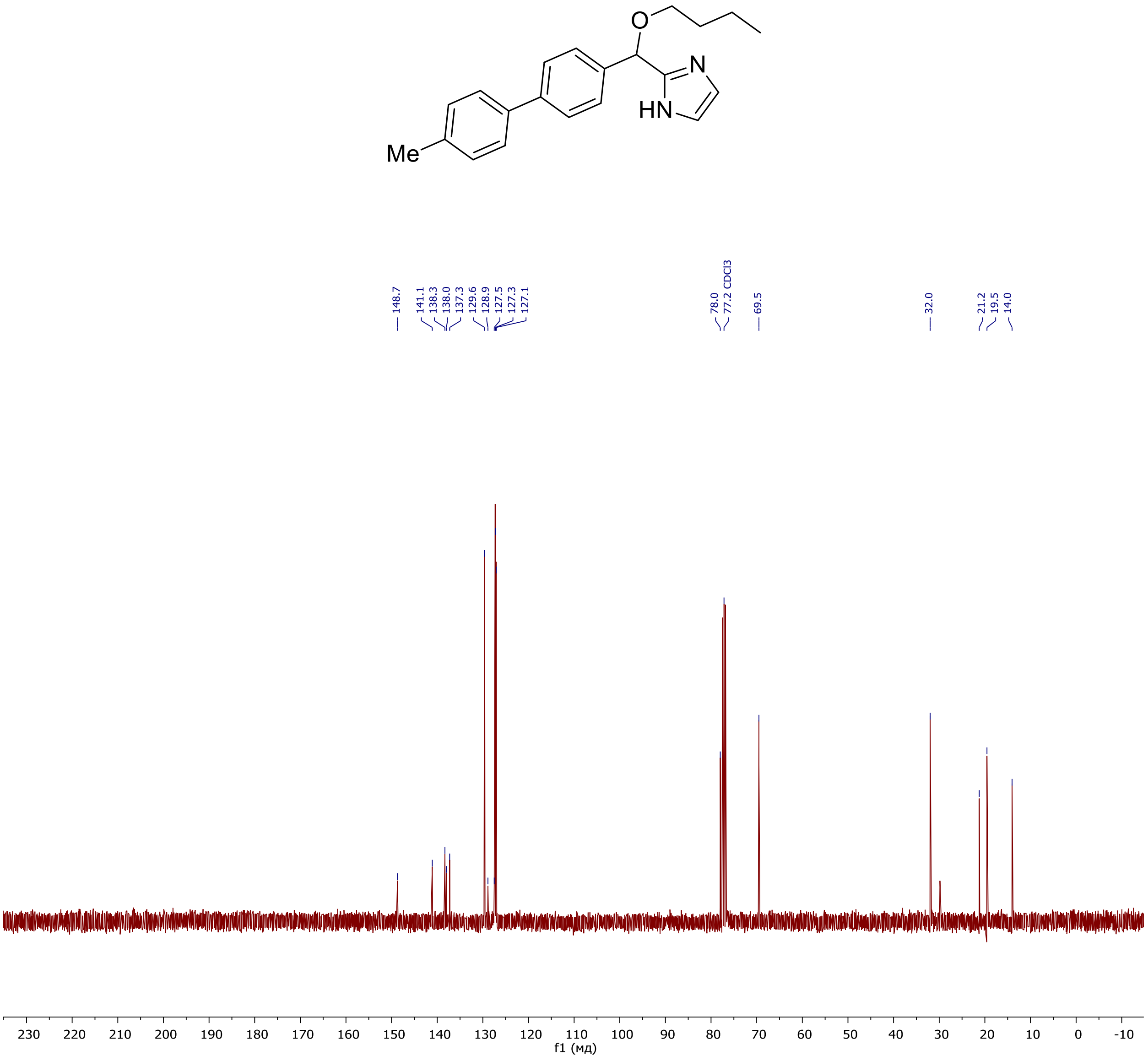

Figure S86. ^{13}C NMR (101 MHz, Chloroform-*d*) spectrum of compound **4p.**

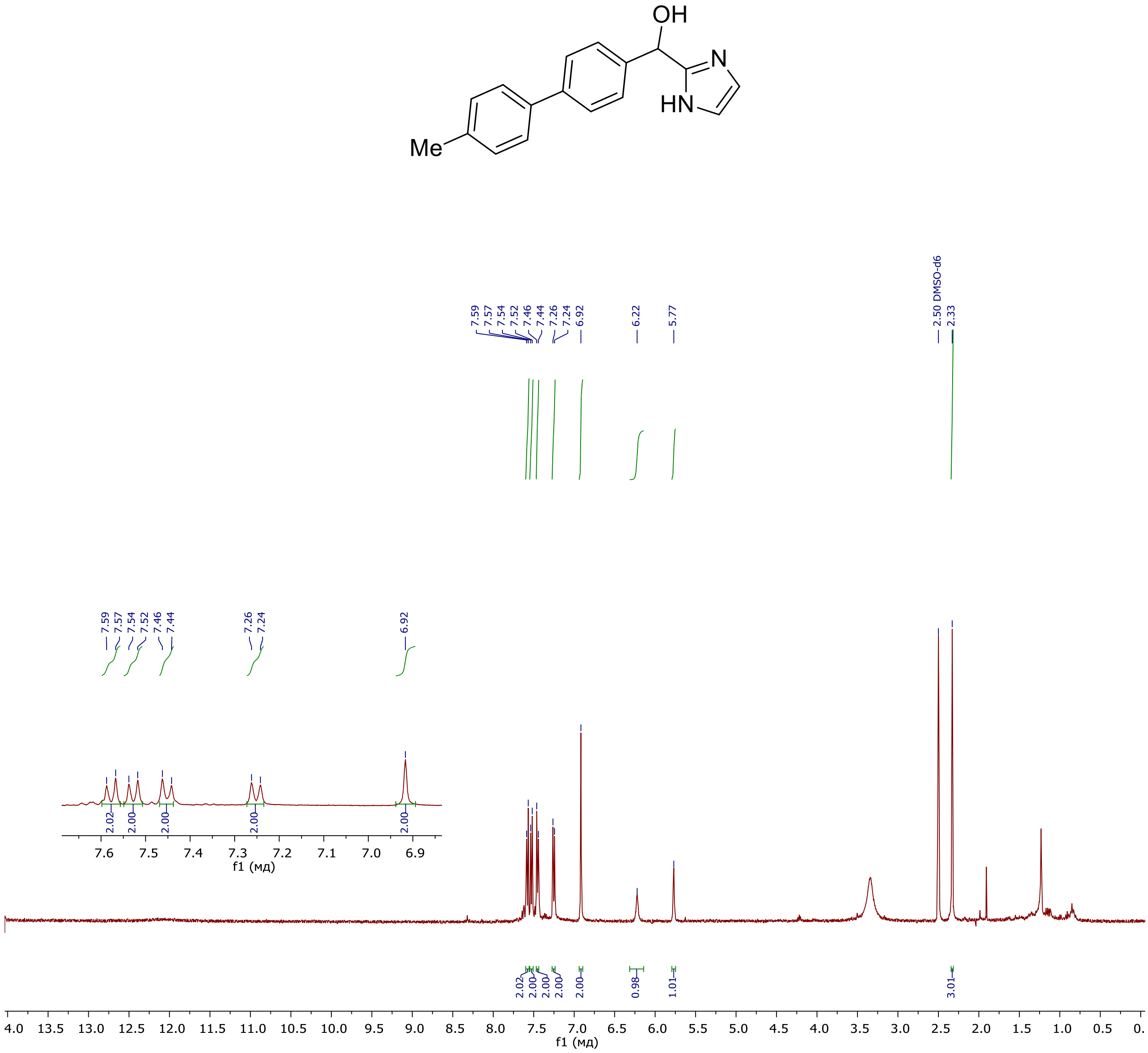

Figure S87. ^{1}H NMR (400 MHz, DMSO-d_6) spectrum of compound **4q.**

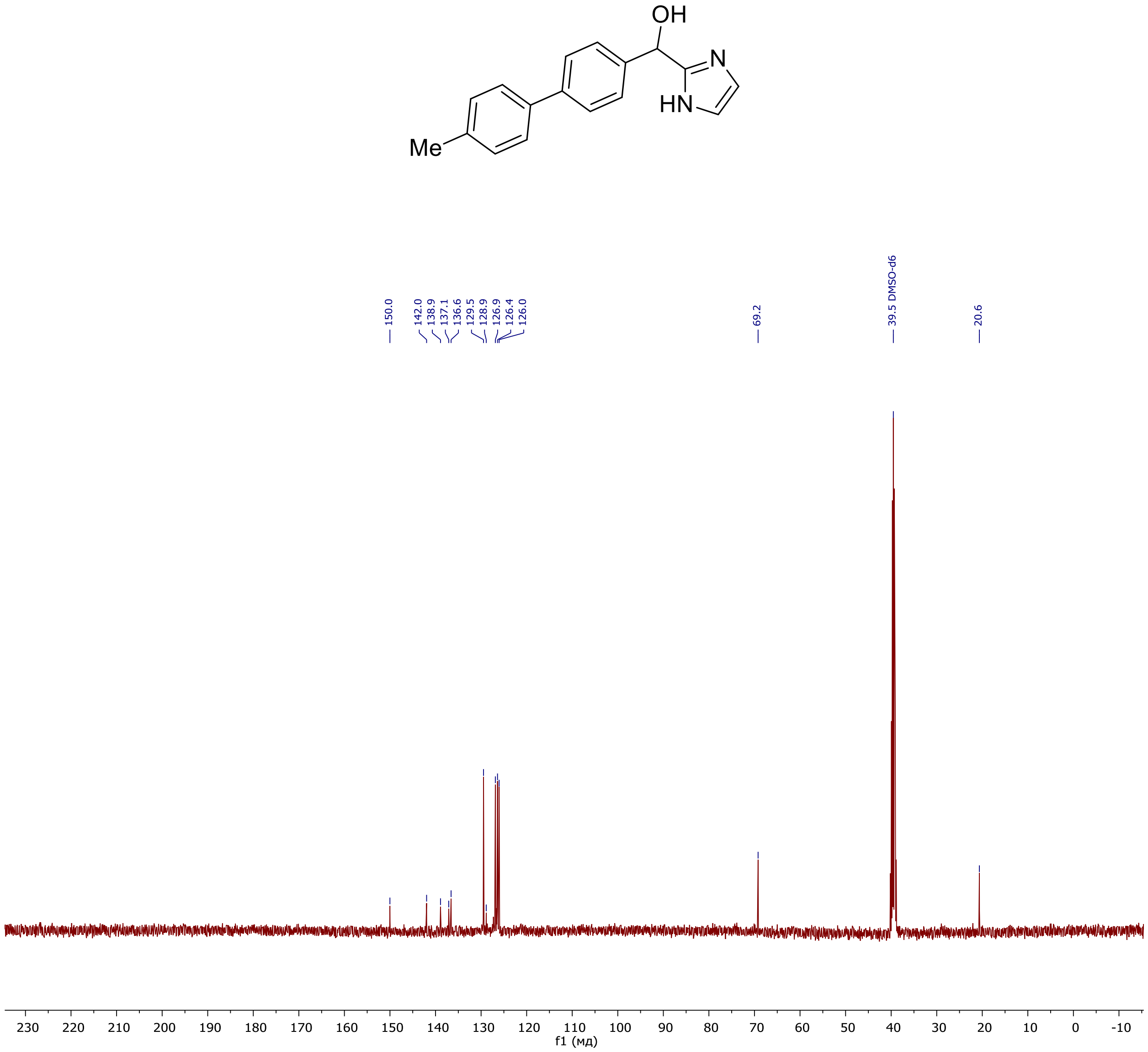

Figure S88. ^{13}C NMR (101 MHz, DMSO-d_6) spectrum of compound **4q.**

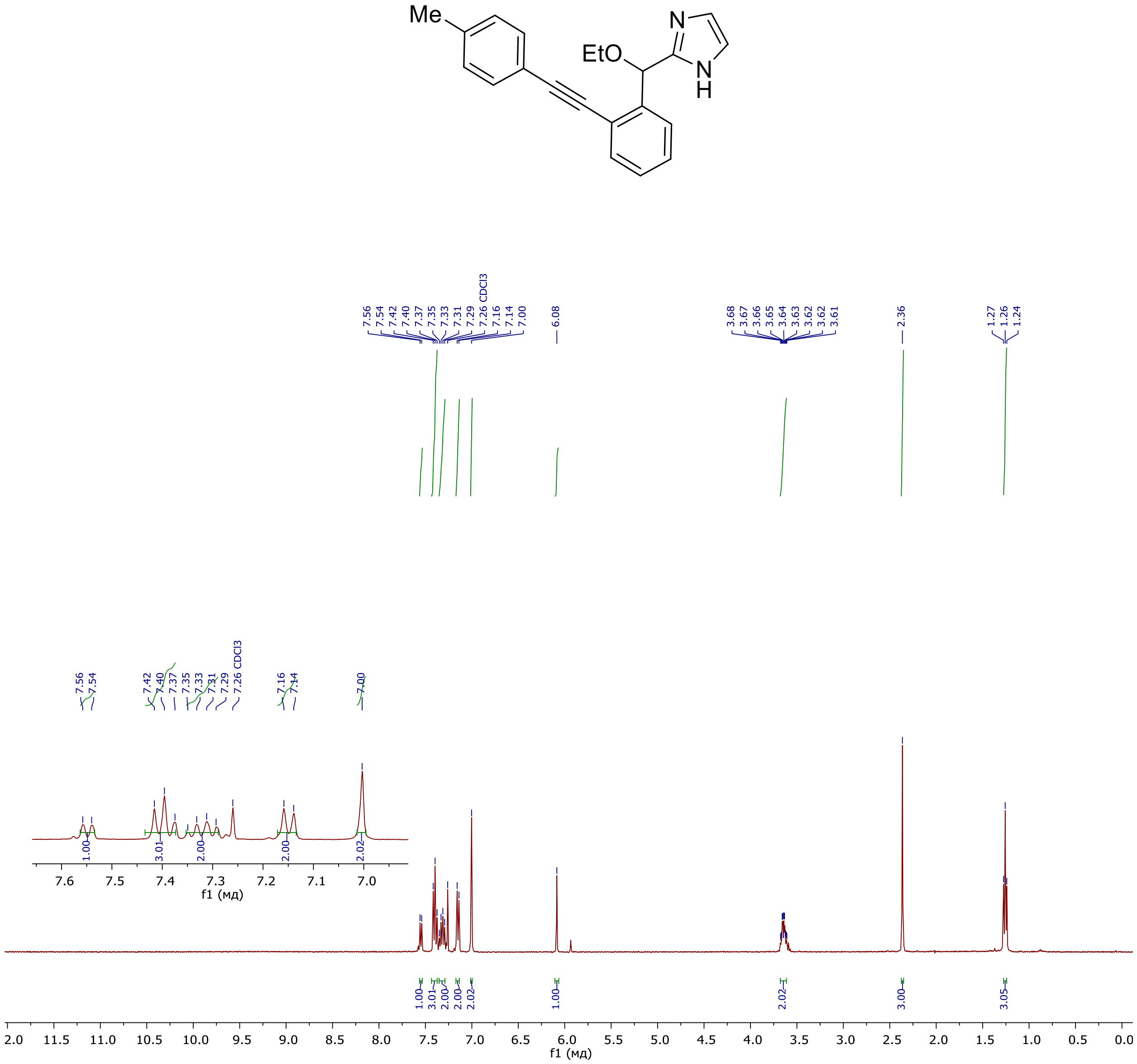

Figure S89. ^{1}H NMR (400 MHz, Chloroform-*d*) spectrum of compound **4r.**

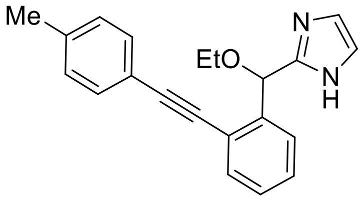

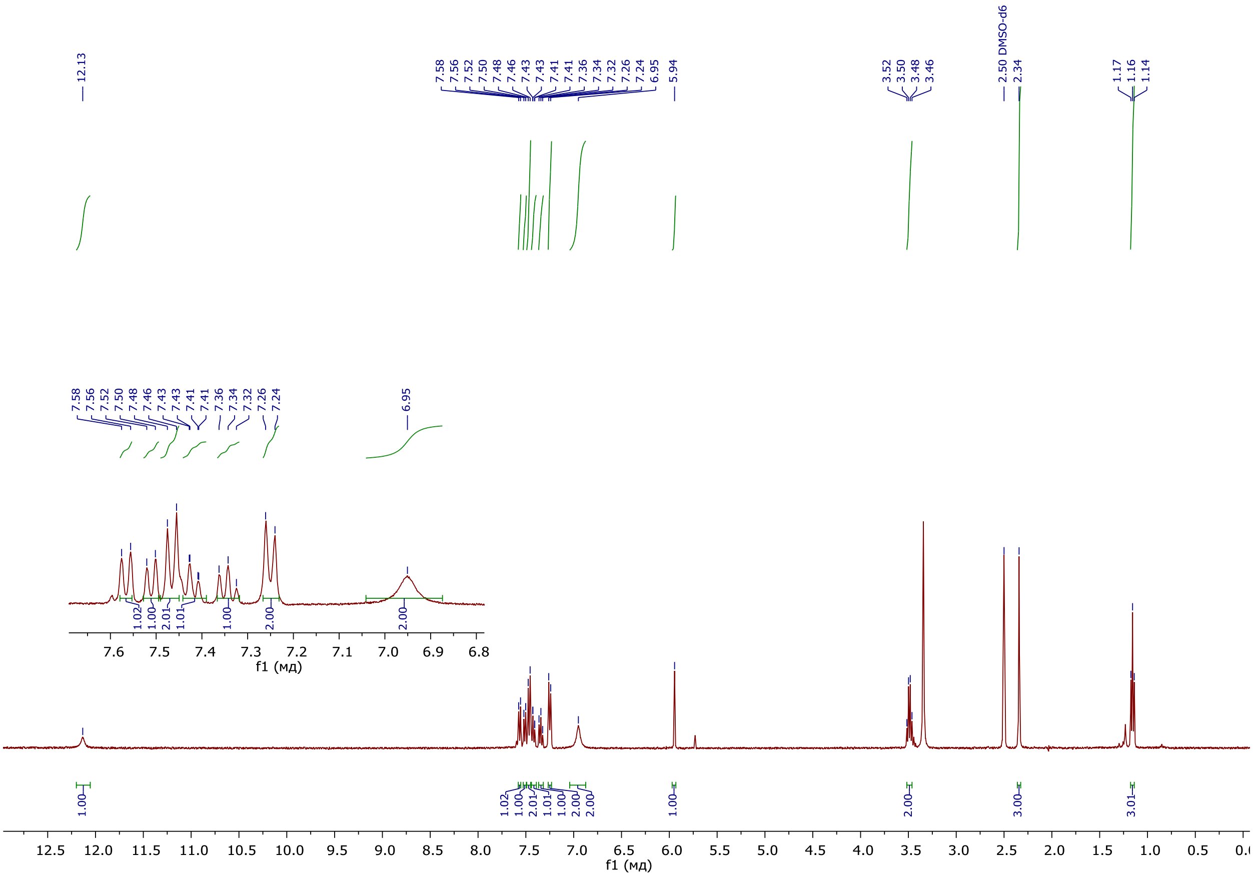

Figure S90 ^{1}H NMR (400 MHz, DMSO-d_6) spectrum of compound **4r.**

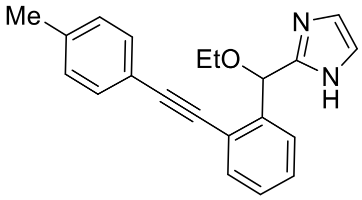

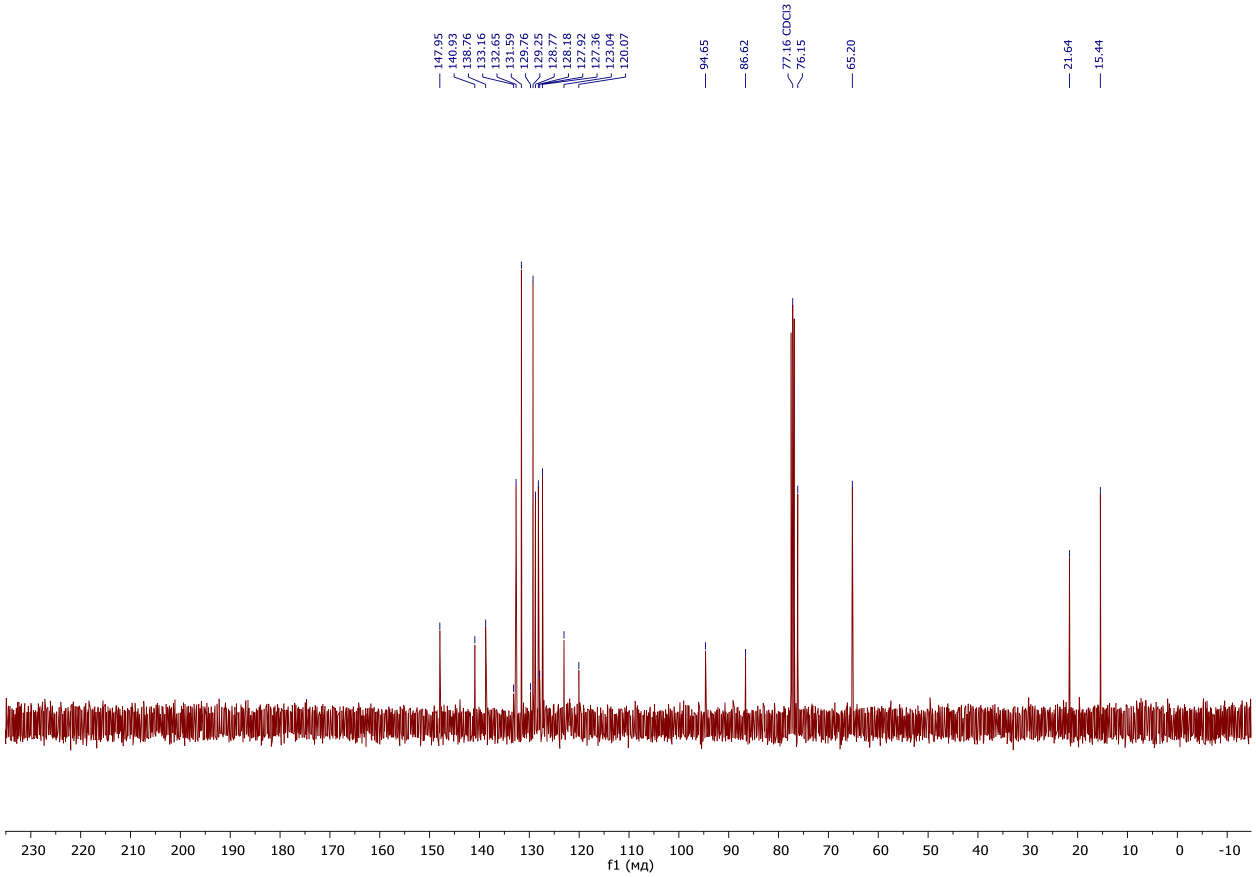

Figure S91. ^{13}C NMR (101 MHz, Chloroform-*d*) spectrum of compound **4r.**

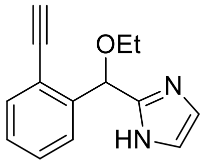

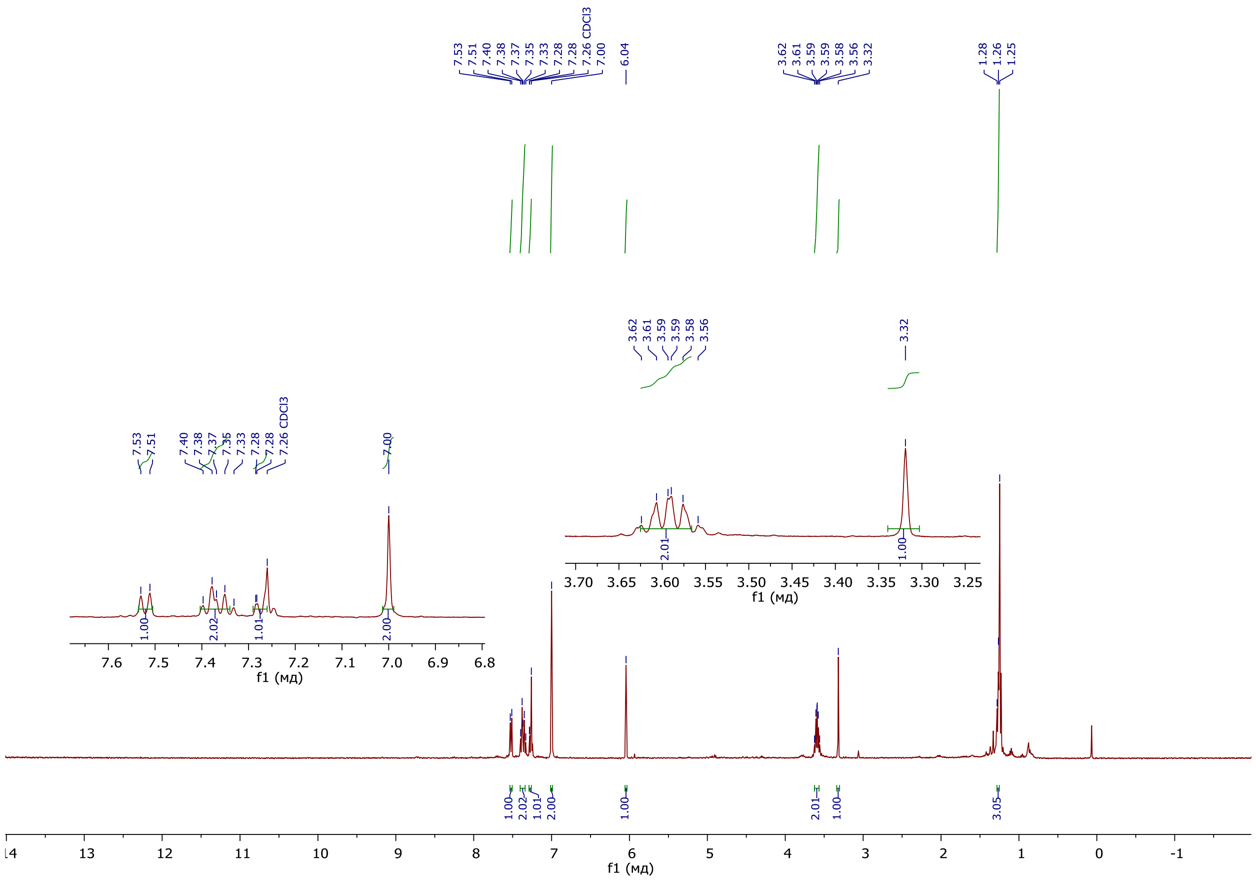

Figure S92. ^{1}H NMR (400 MHz, Chloroform-*d*) spectrum of compound **4s.**

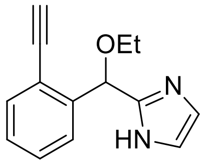

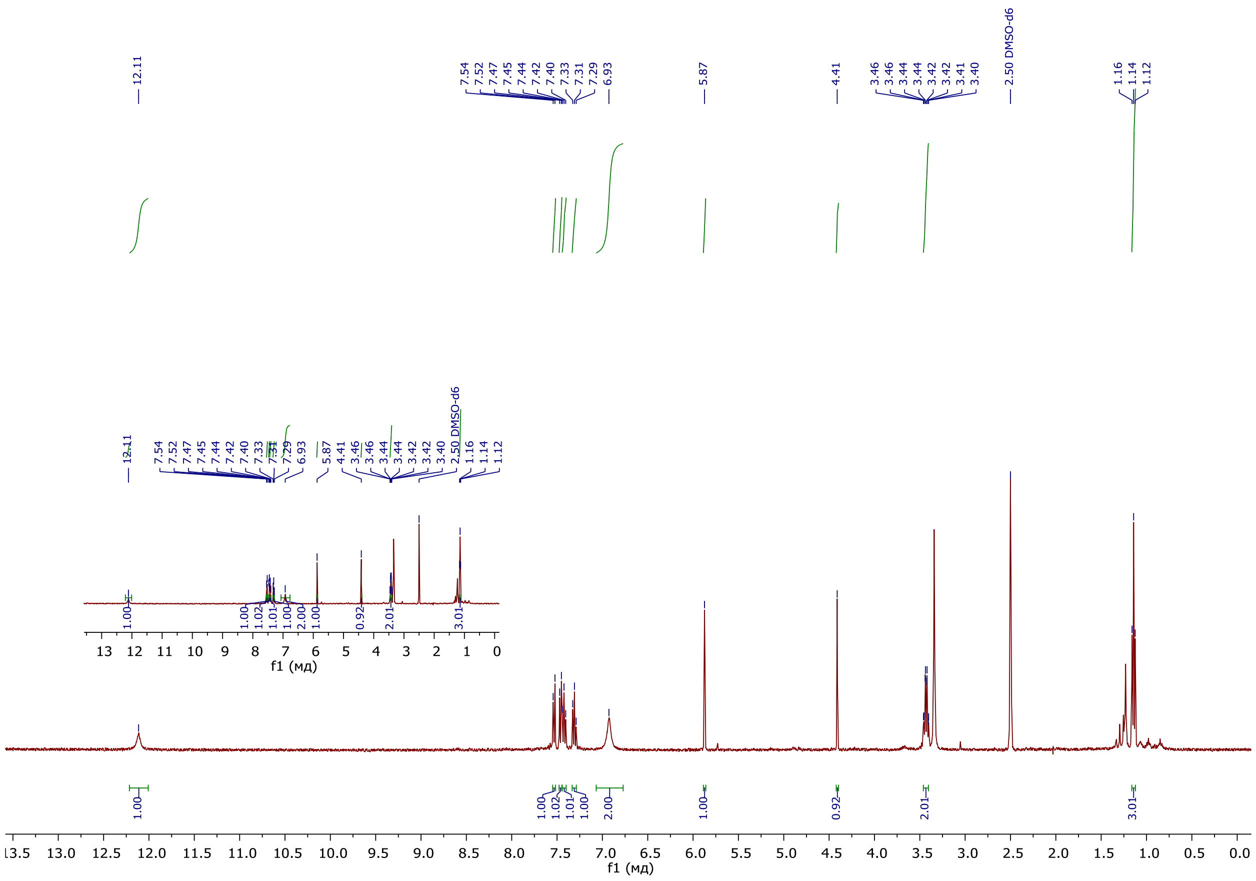

Figure S93. ^{1}H NMR (400 MHz, DMSO-d_6) spectrum of compound **4s.**

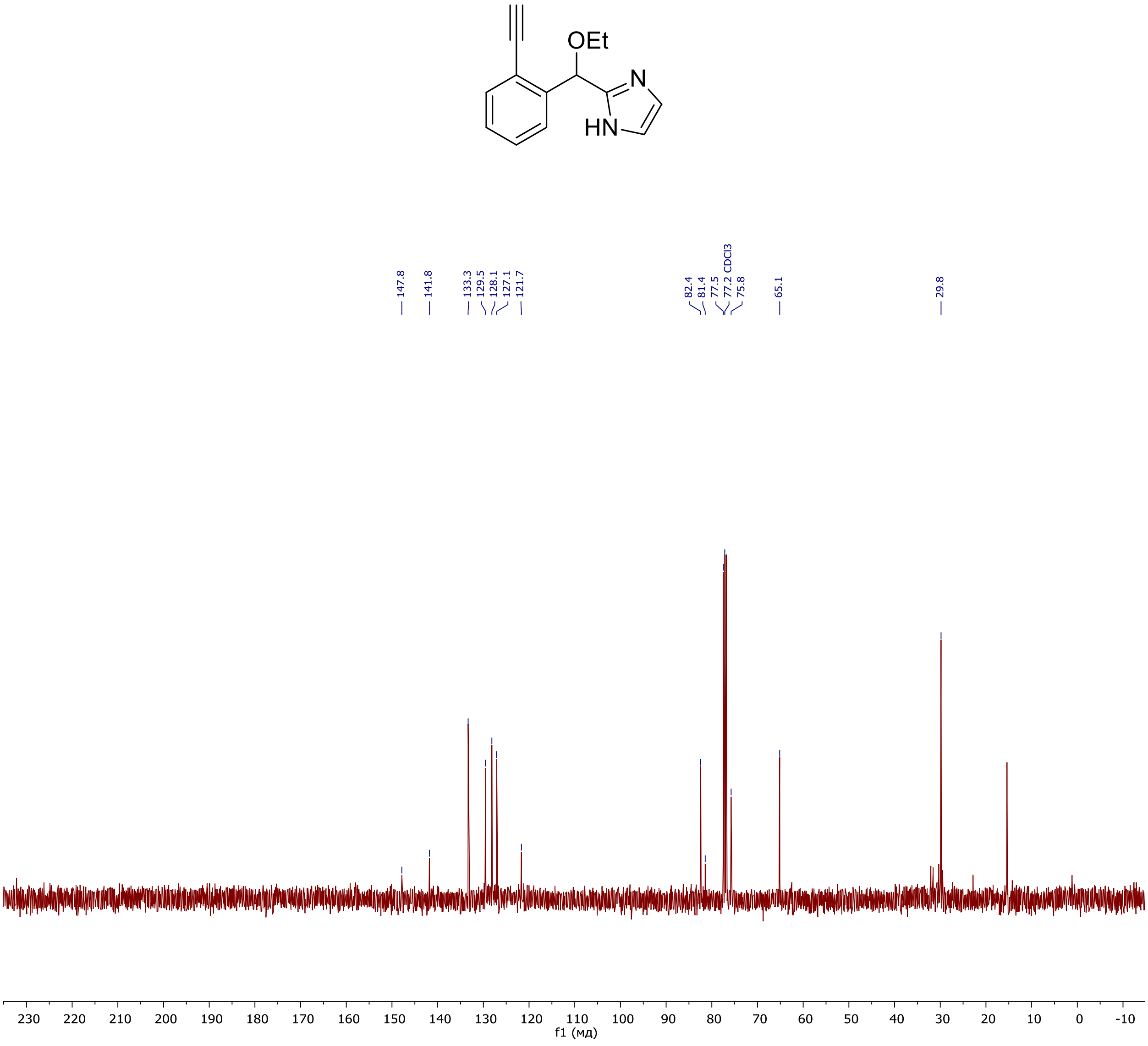

Figure S94. ^{13}C NMR (101 MHz, Chloroform-*d*) spectrum of compound **4s.**

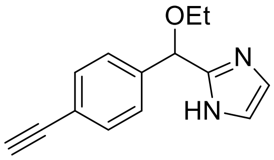

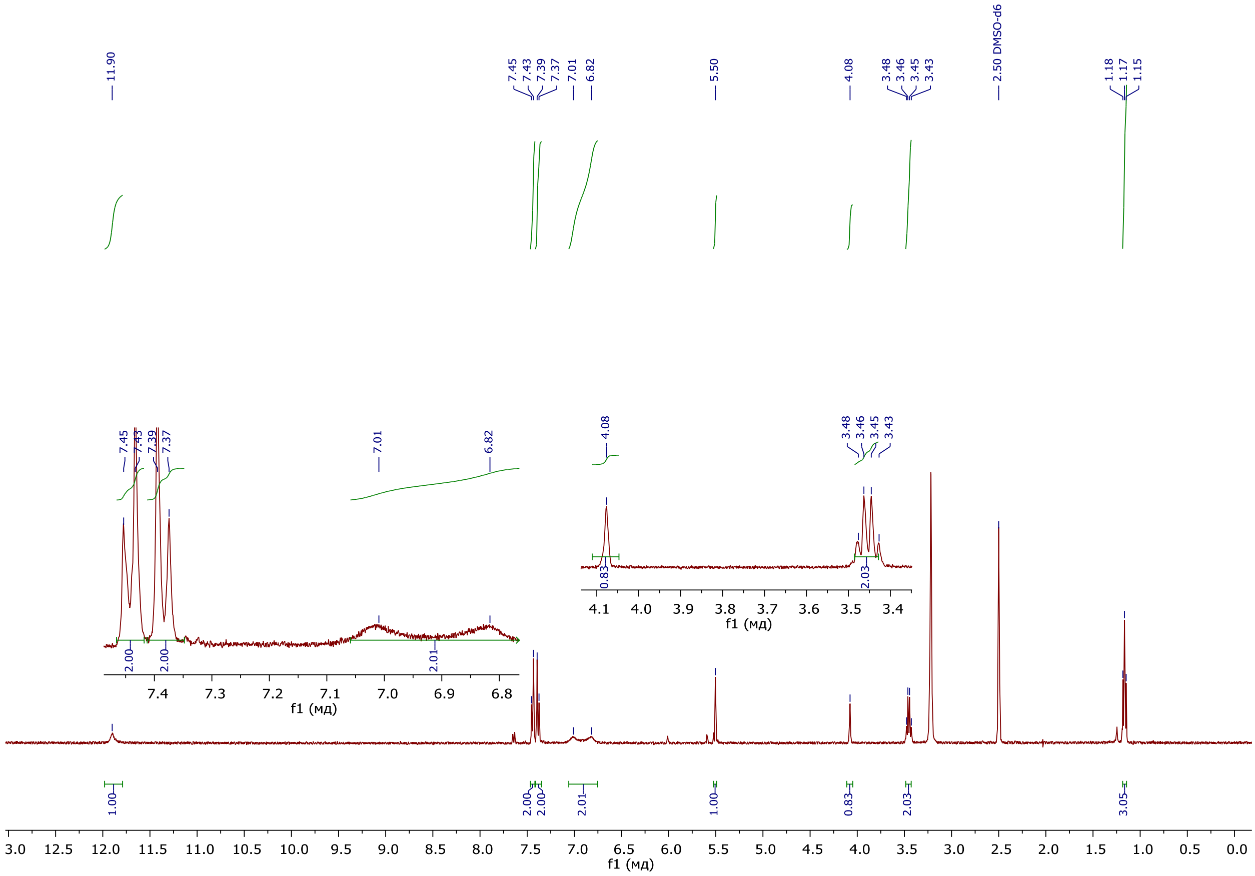

Figure S95. ^{1}H NMR (400 MHz, DMSO-d_6) spectrum of compound **4t.**

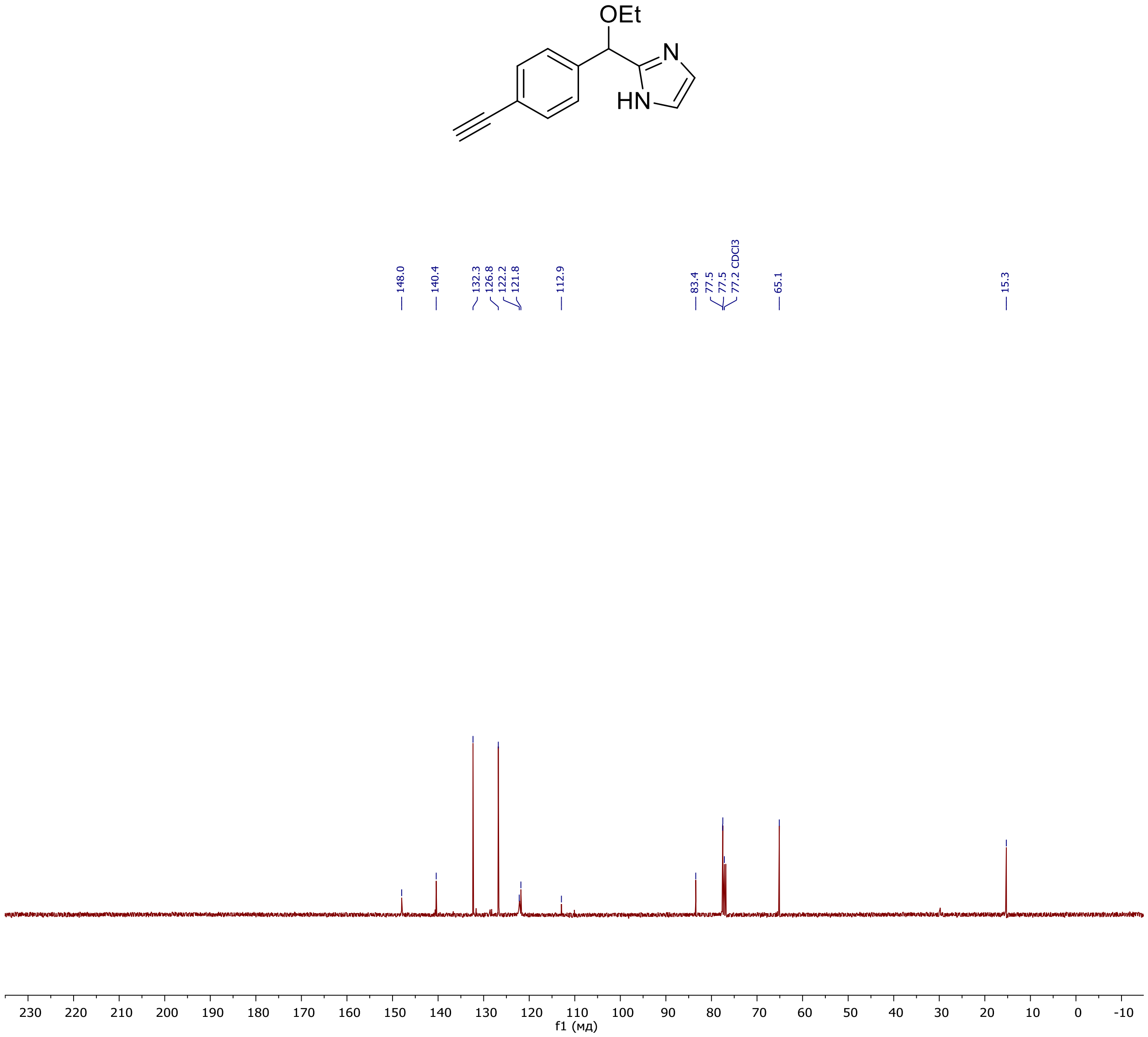

Figure S96. ^{13}C NMR (101 MHz, Chloroform-*d*) spectrum of compound **4t.**

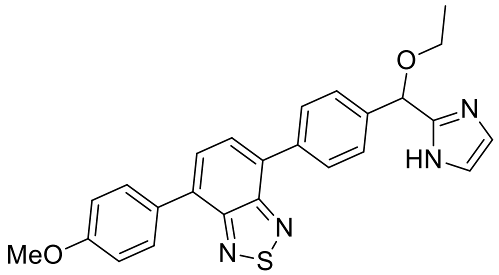

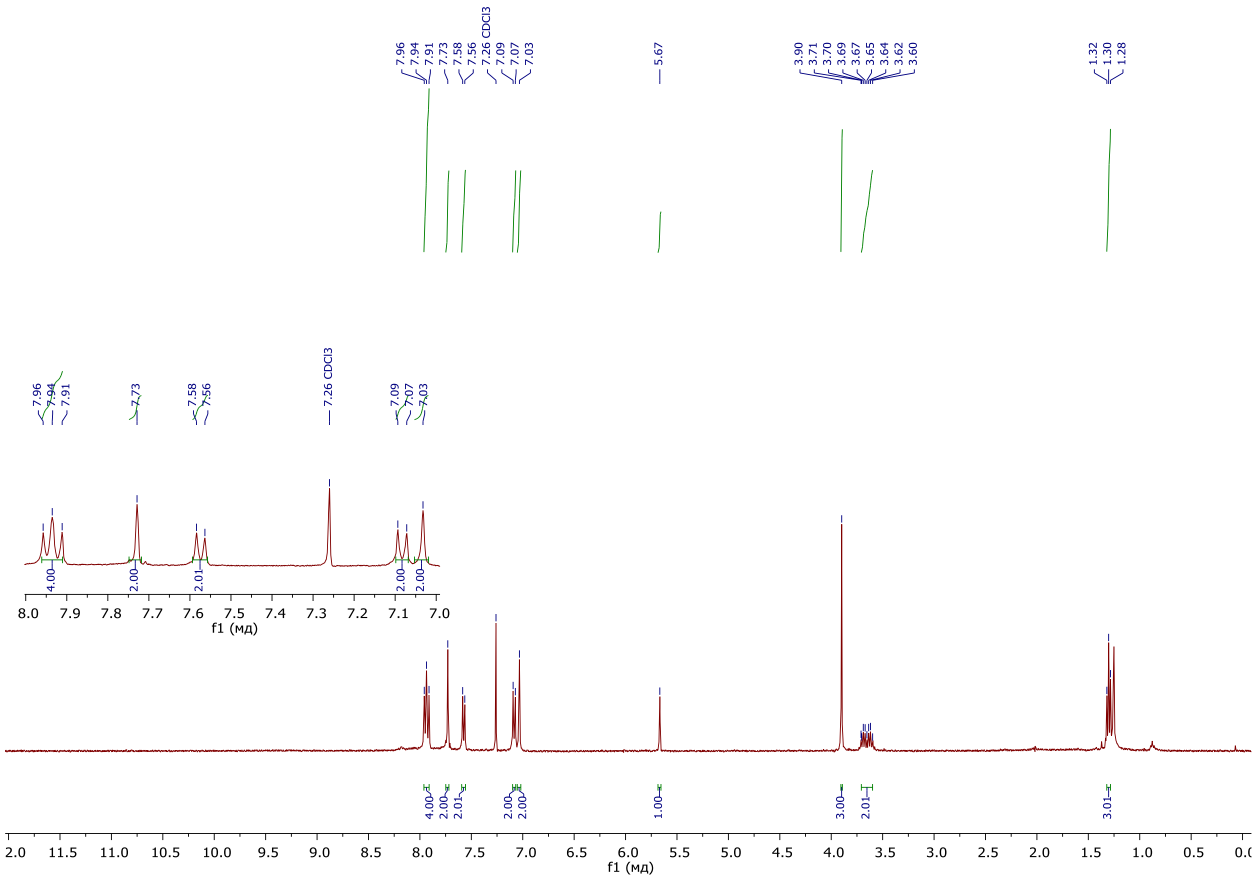

Figure S97. ^{1}H NMR (400 MHz, Chloroform-*d*) spectrum of compound **4u.**

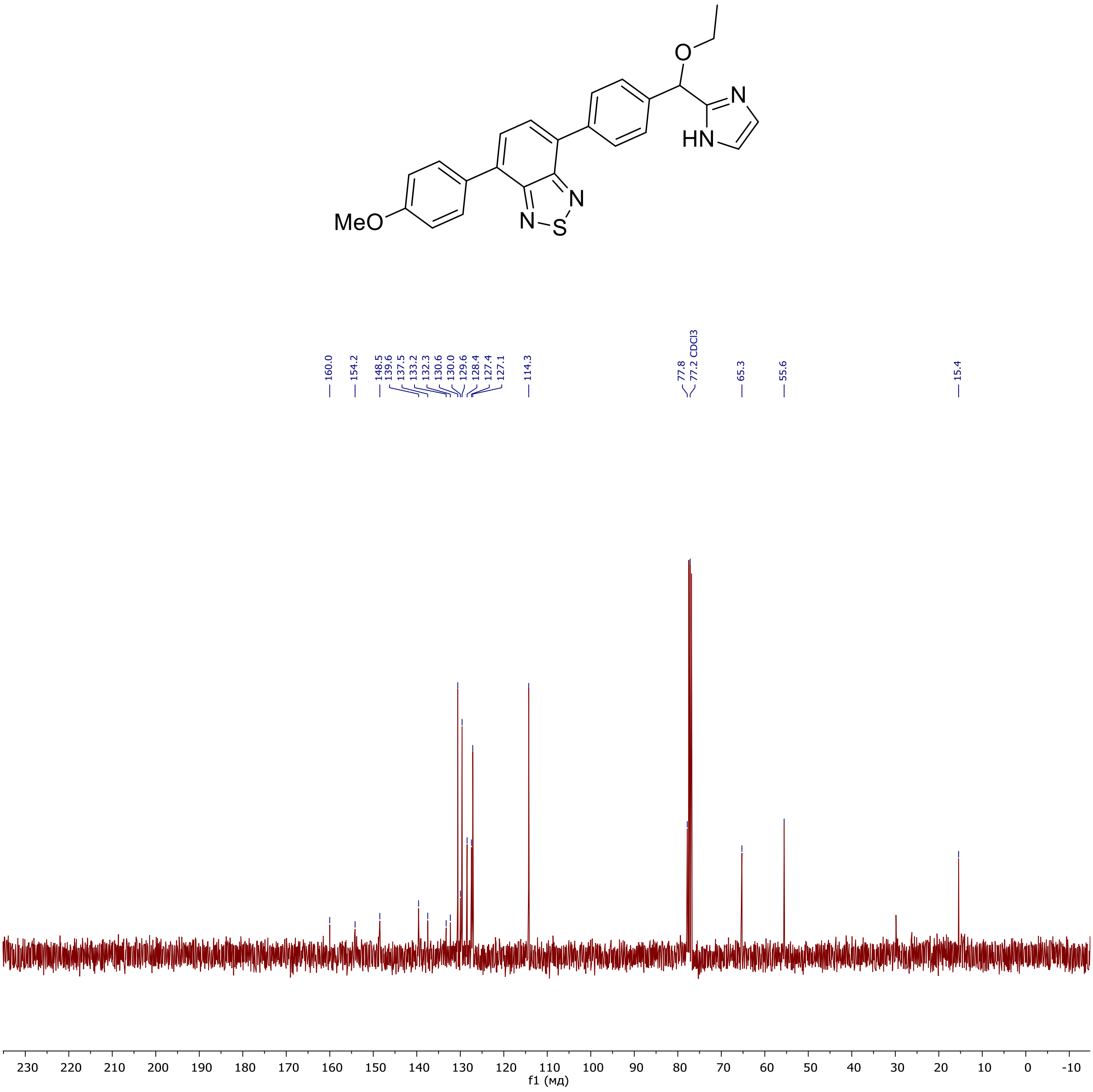

Figure S98. ^{13}C NMR (101 MHz, Chloroform-*d*) spectrum of compound **4u.**

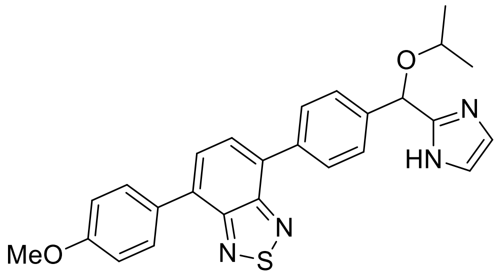

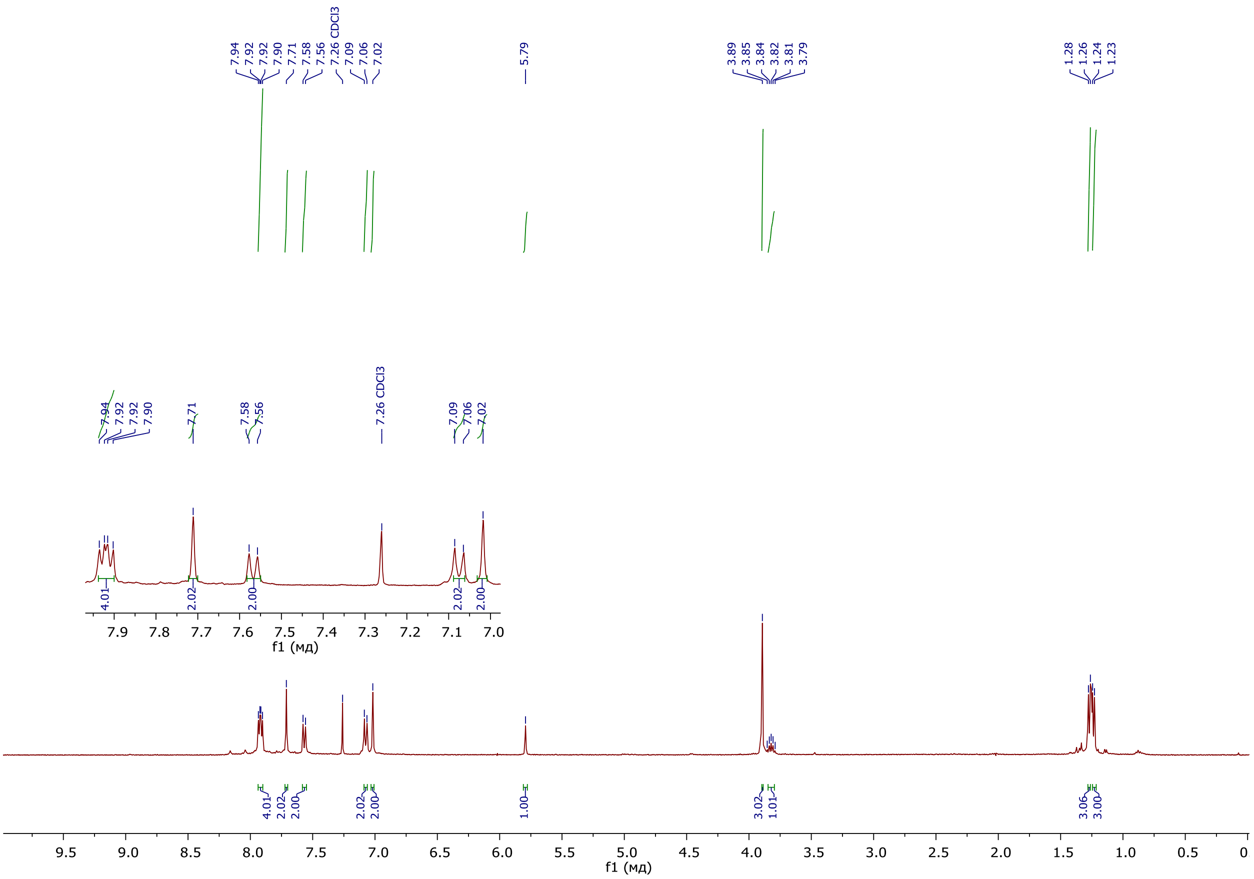

Figure S99 ^{1}H NMR (400 MHz, Chloroform-*d*) spectrum of compound **4v.**

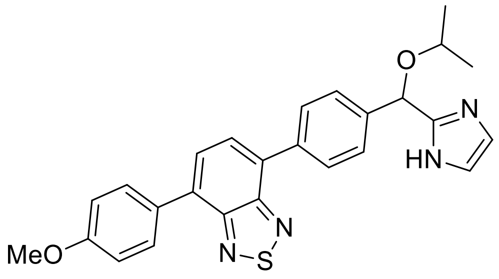

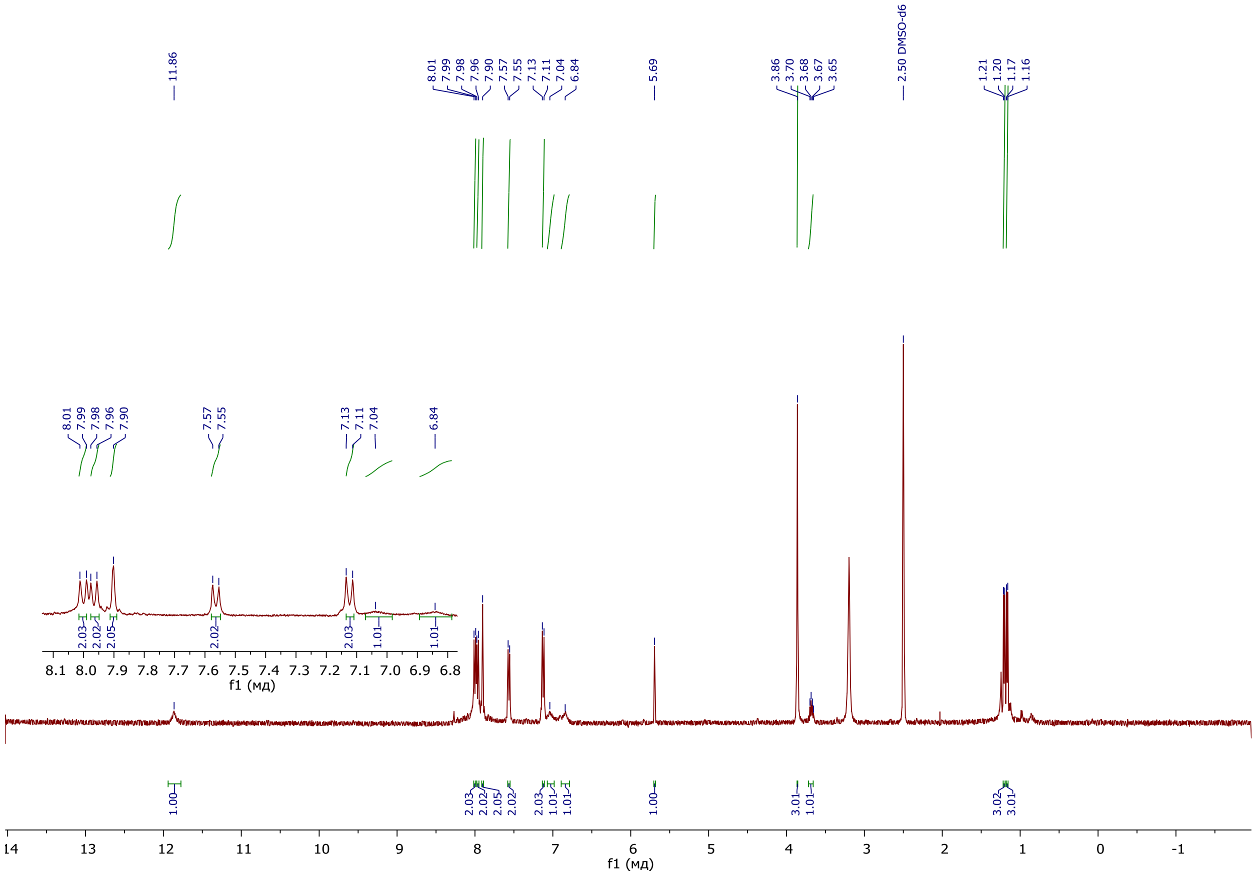

Figure S100. ^{1}H NMR (400 MHz, DMSO-d_6) spectrum of compound **4v.**

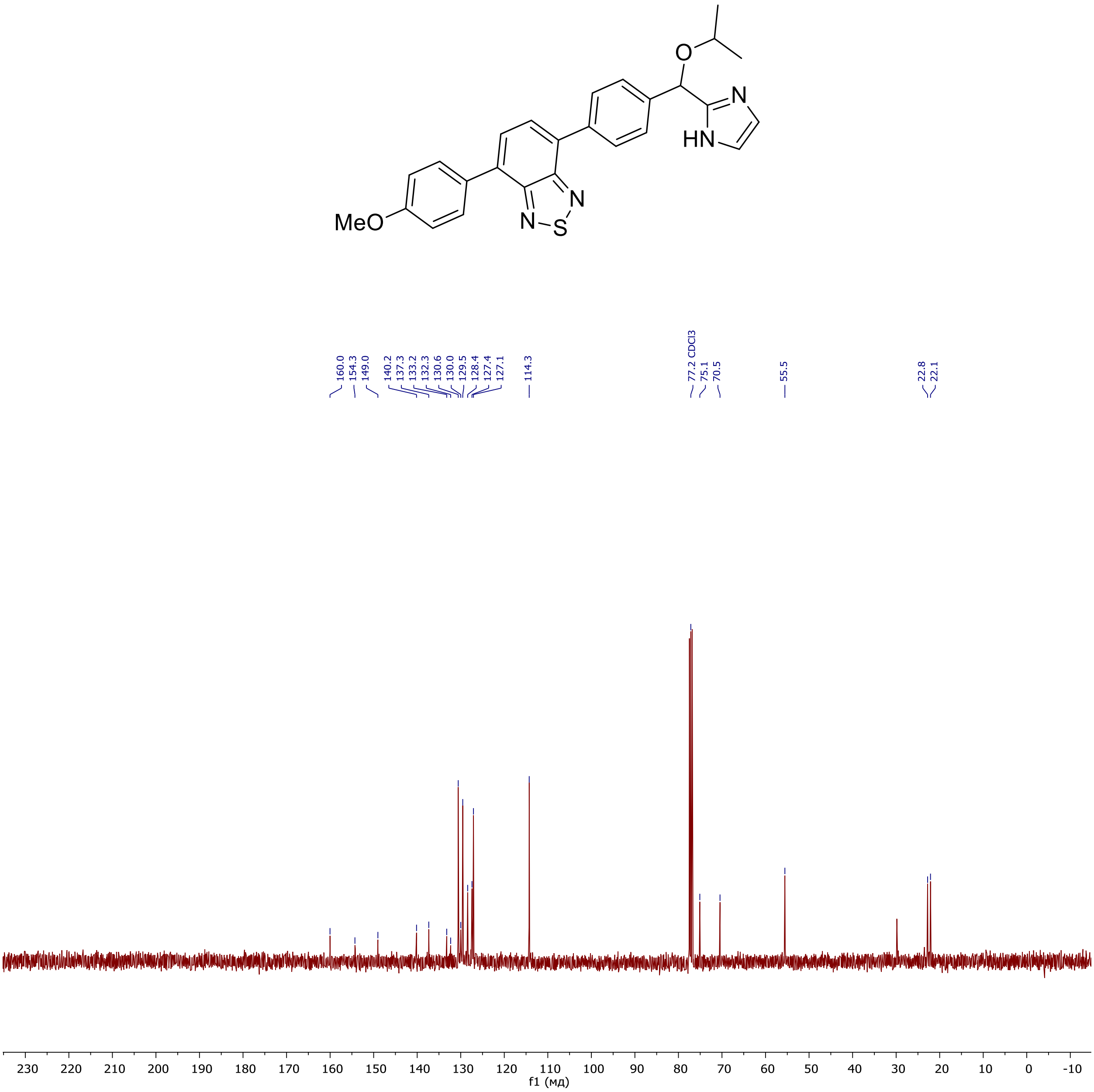

Figure S101. ^{13}C NMR (101 MHz, Chloroform-*d*) spectrum of compound **4v.**

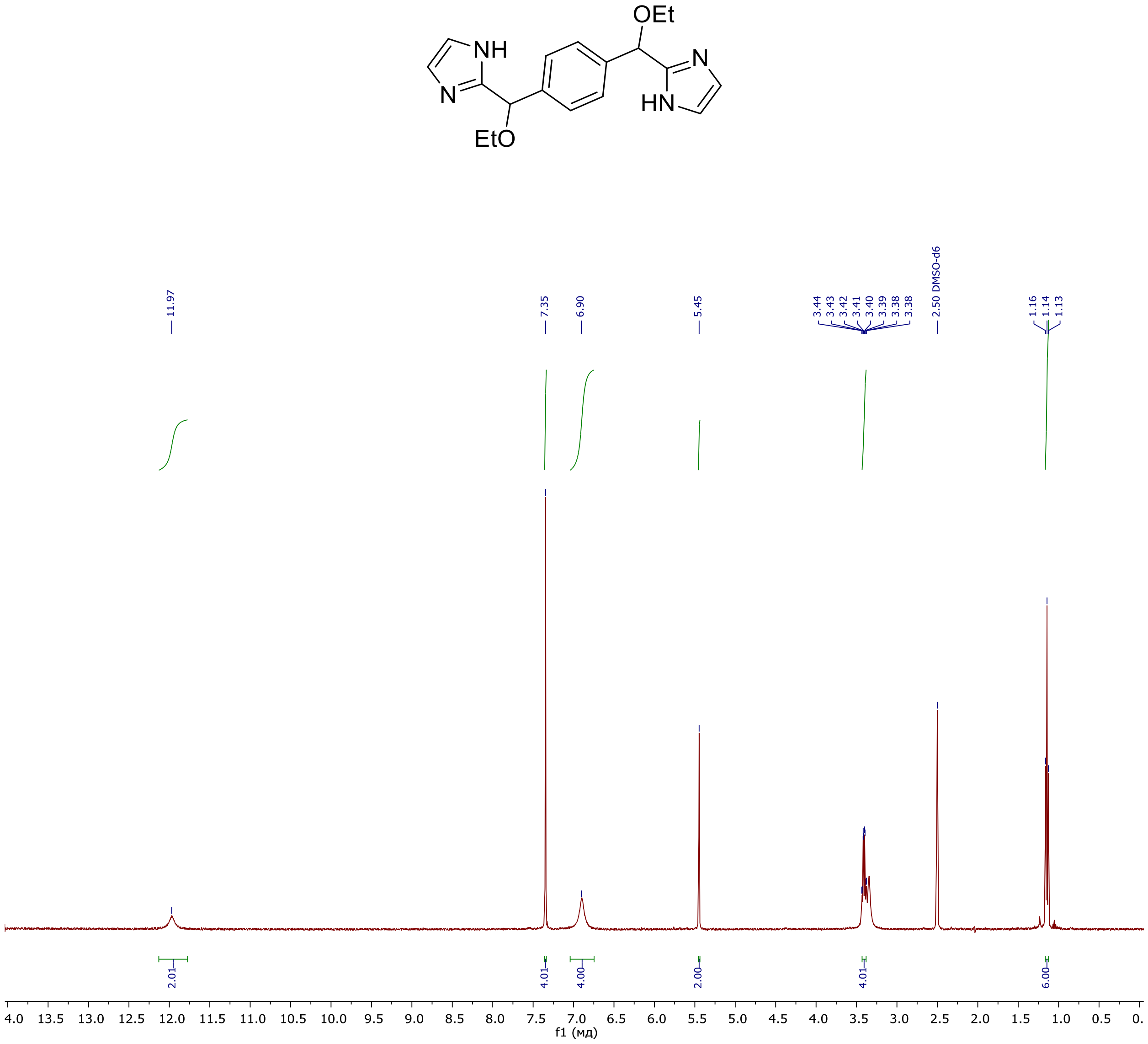

Figure S102. ^{1}H NMR (400 MHz, DMSO-d_6) spectrum of compound **4w.**

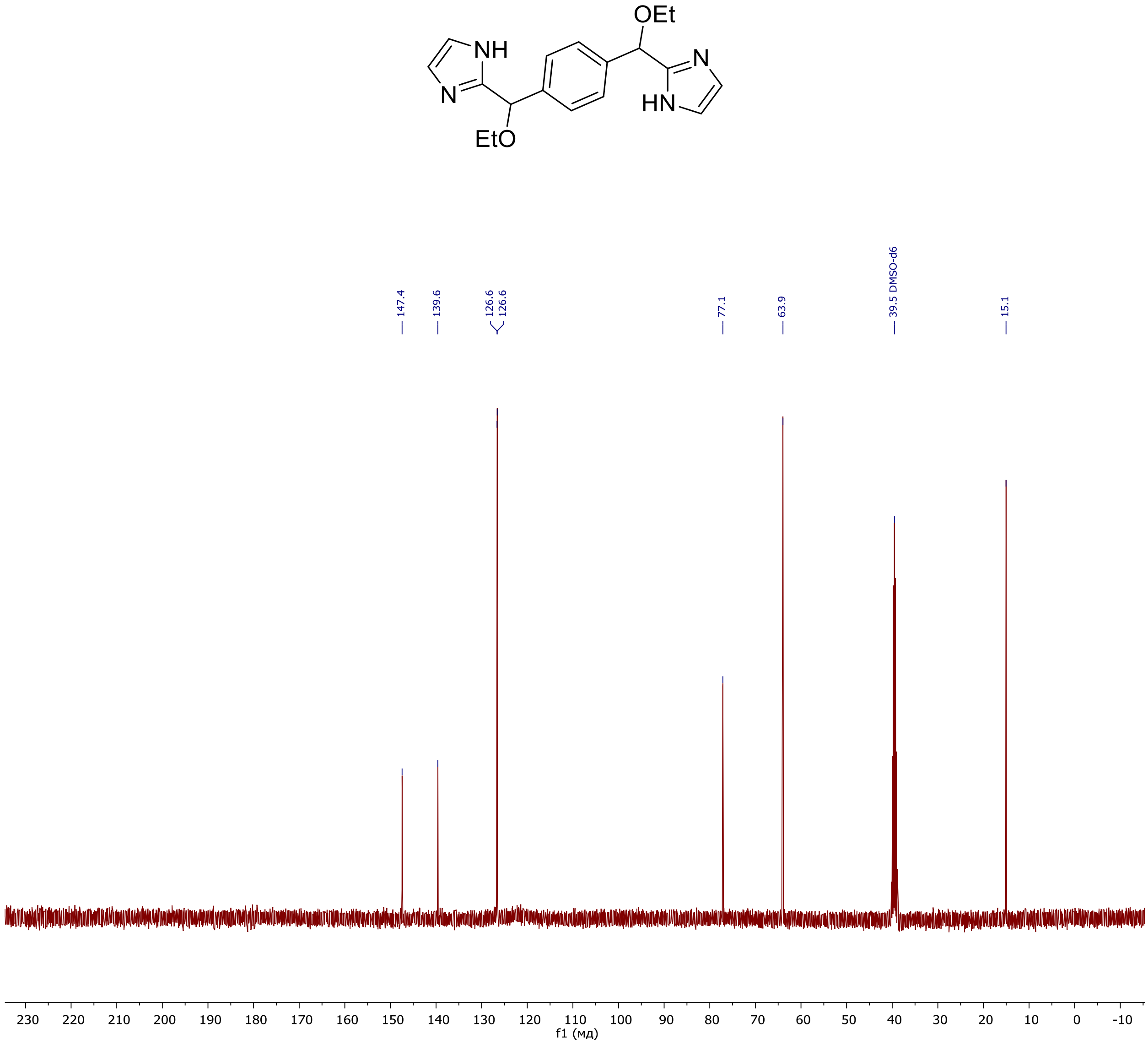

Figure S103. ^{13}C NMR (101 MHz, DMSO-d_6) spectrum of compound **4w.**

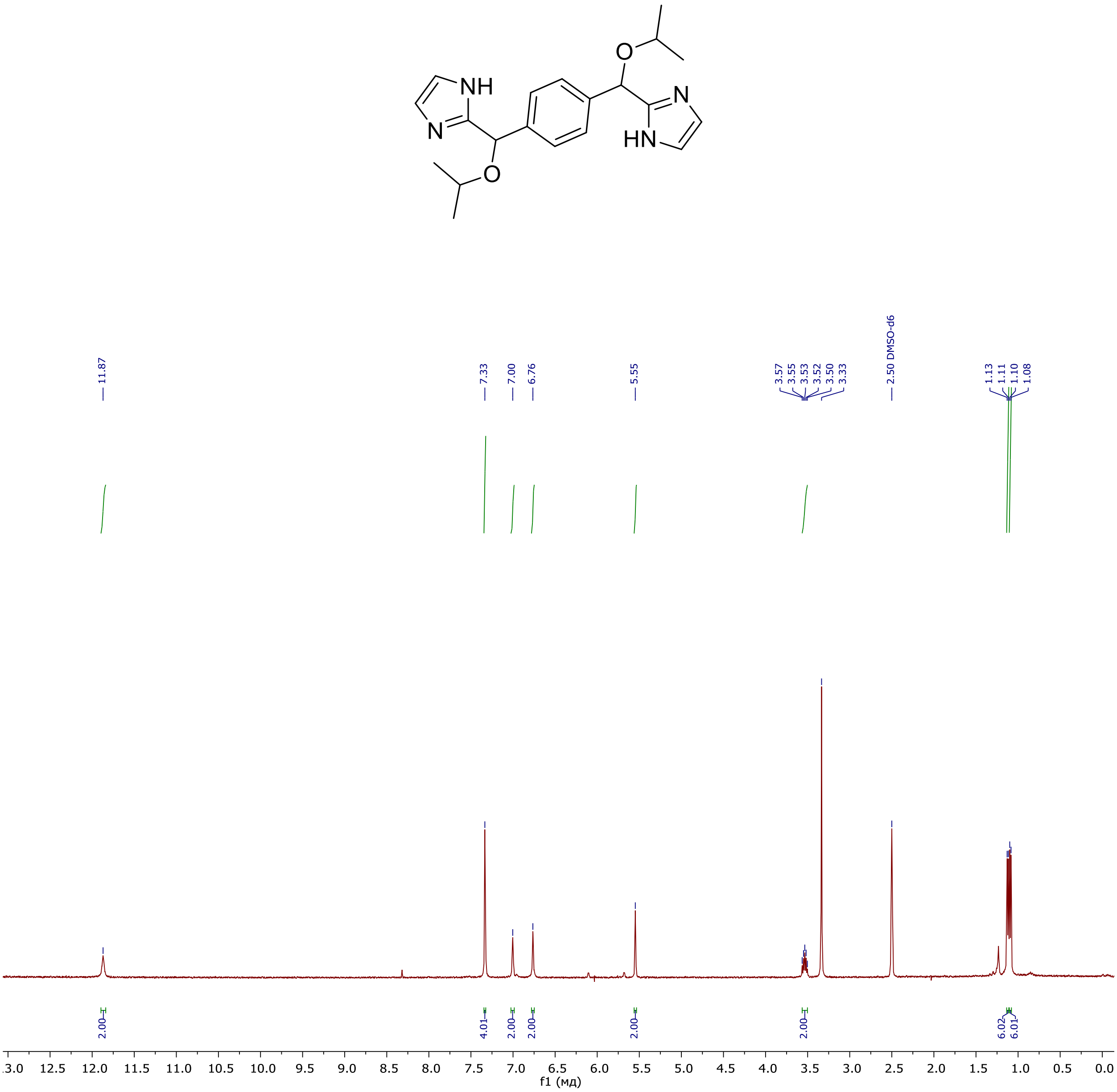

Figure S104. ^{1}H NMR (400 MHz, DMSO-d_6) spectrum of compound **4x.**

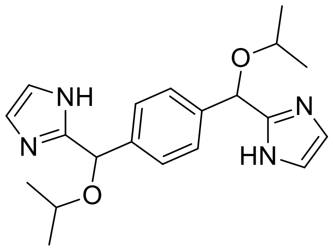

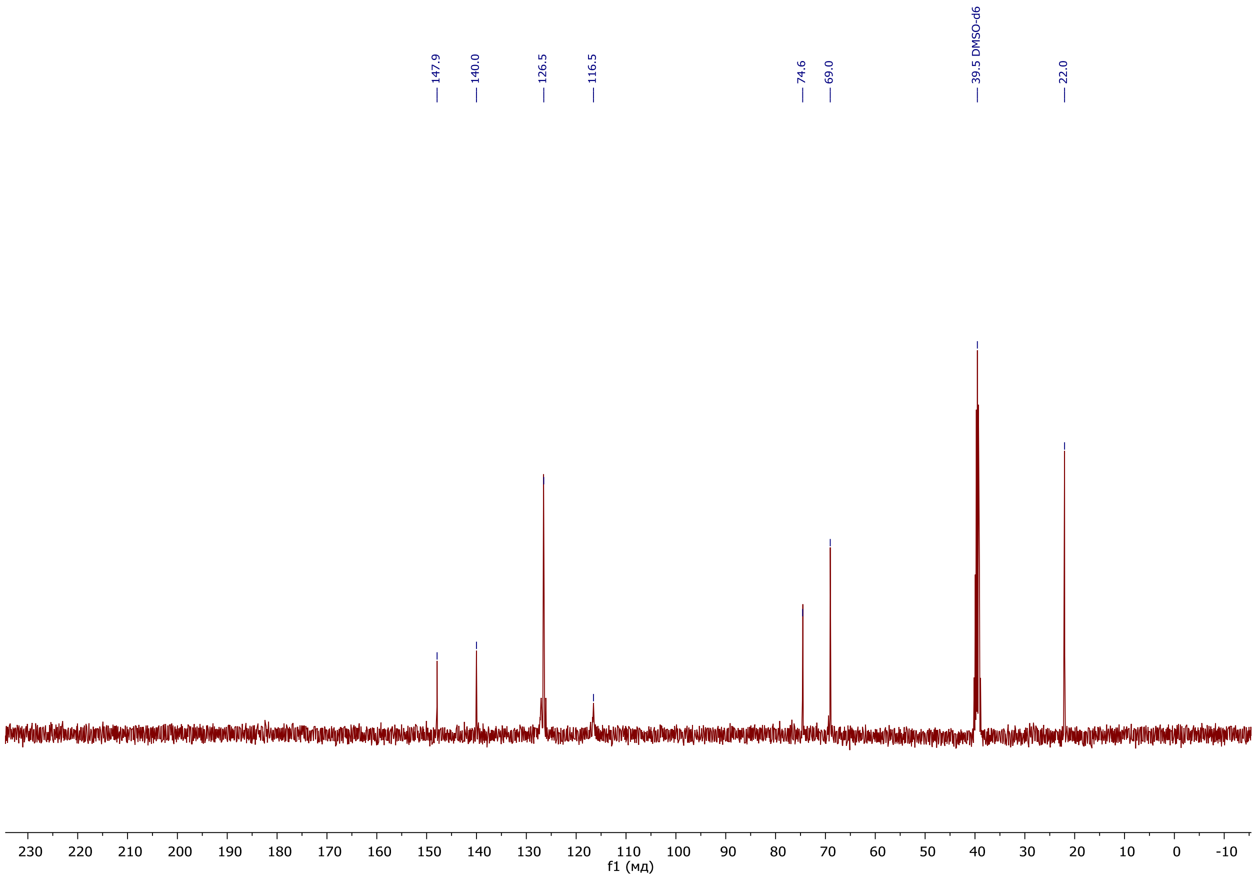

Figure S105. ^{13}C NMR (101 MHz, DMSO-d_6) spectrum of compound **4x.**

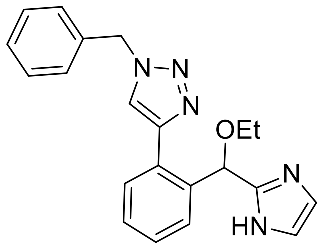

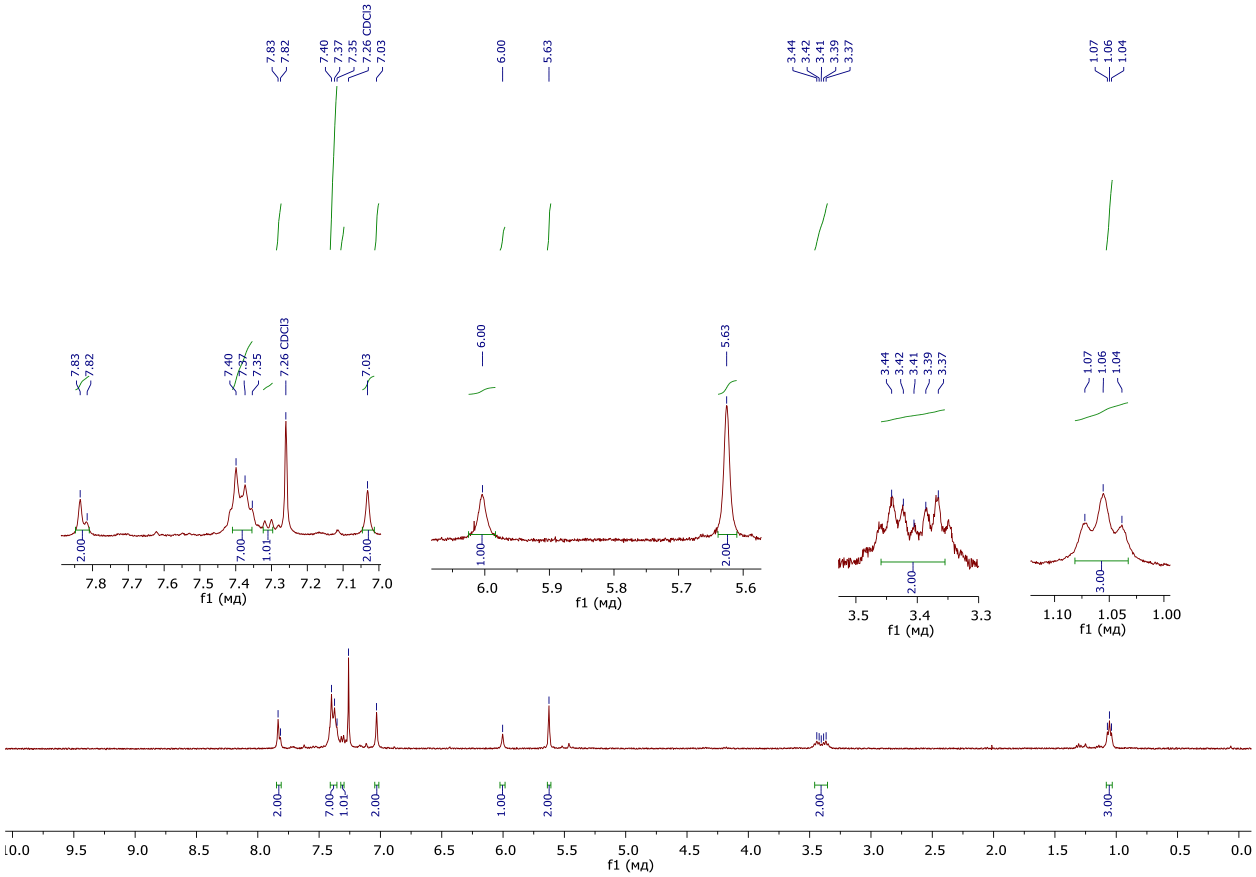

Figure S106. ^{1}H NMR (400 MHz, Chloroform-*d*) spectrum of compound **4y.**

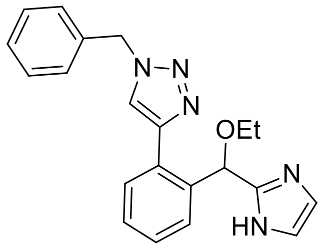

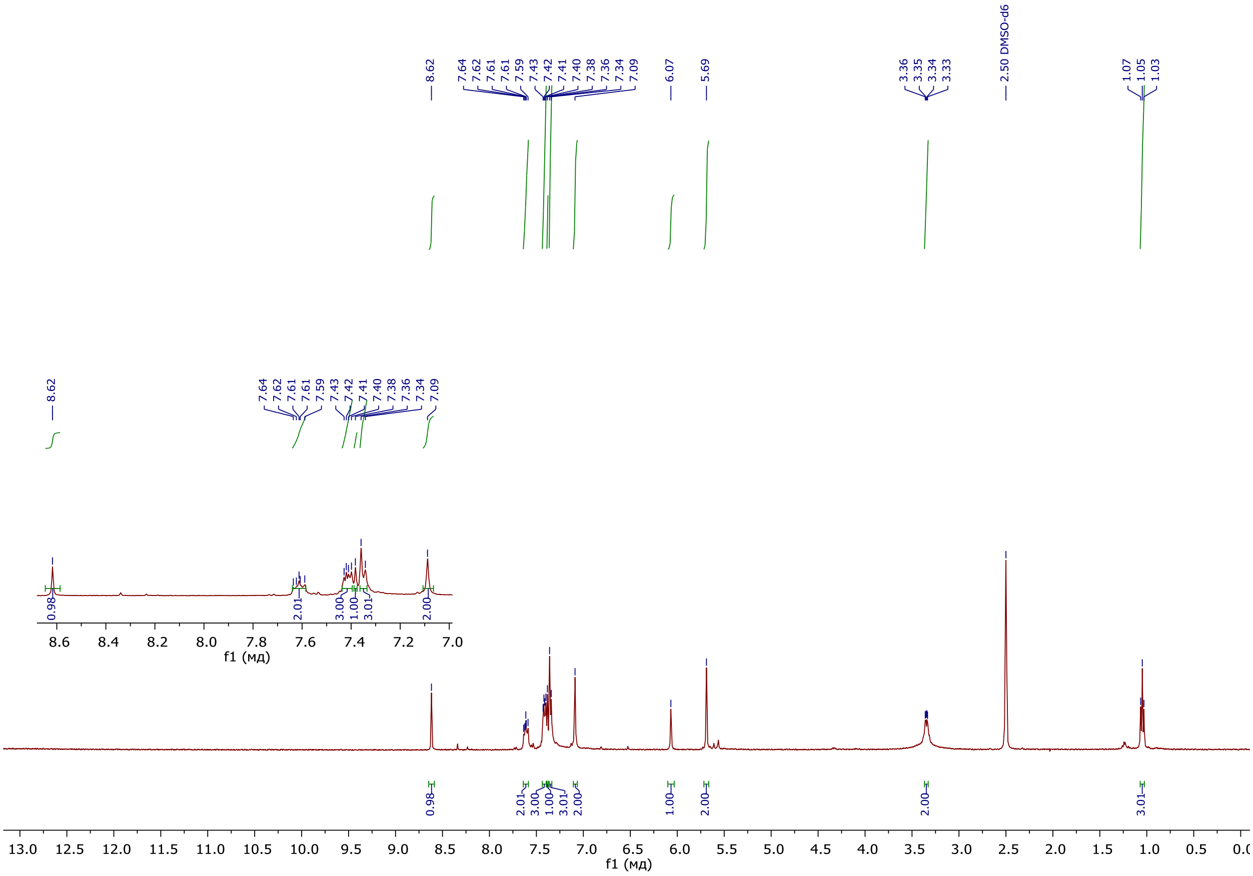

Figure S107. ^{1}H NMR (400 MHz, DMSO-d_6) spectrum of compound **4y.**

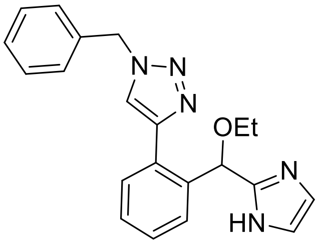

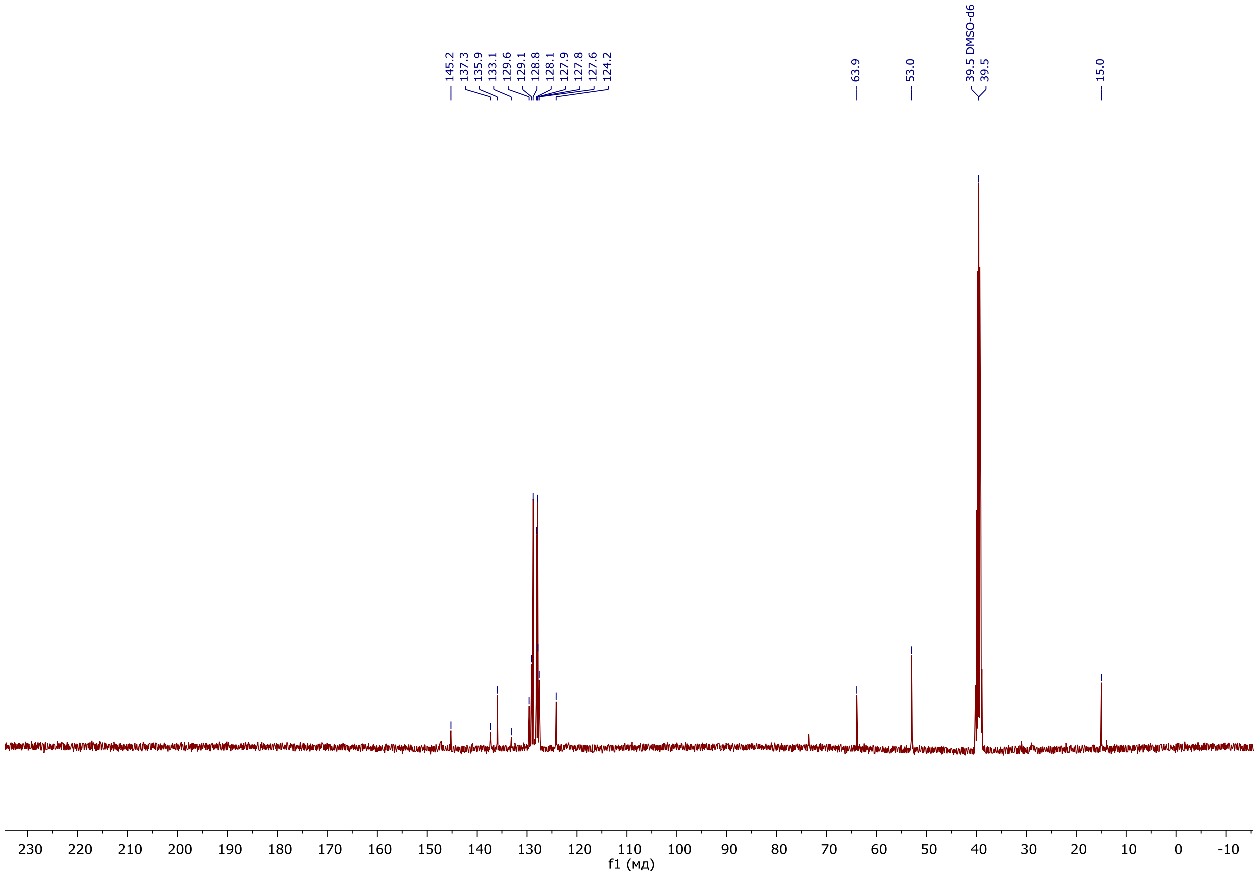

Figure S108. ^{13}C NMR (101 MHz, DMSO-d_6) spectrum of compound **4y.**

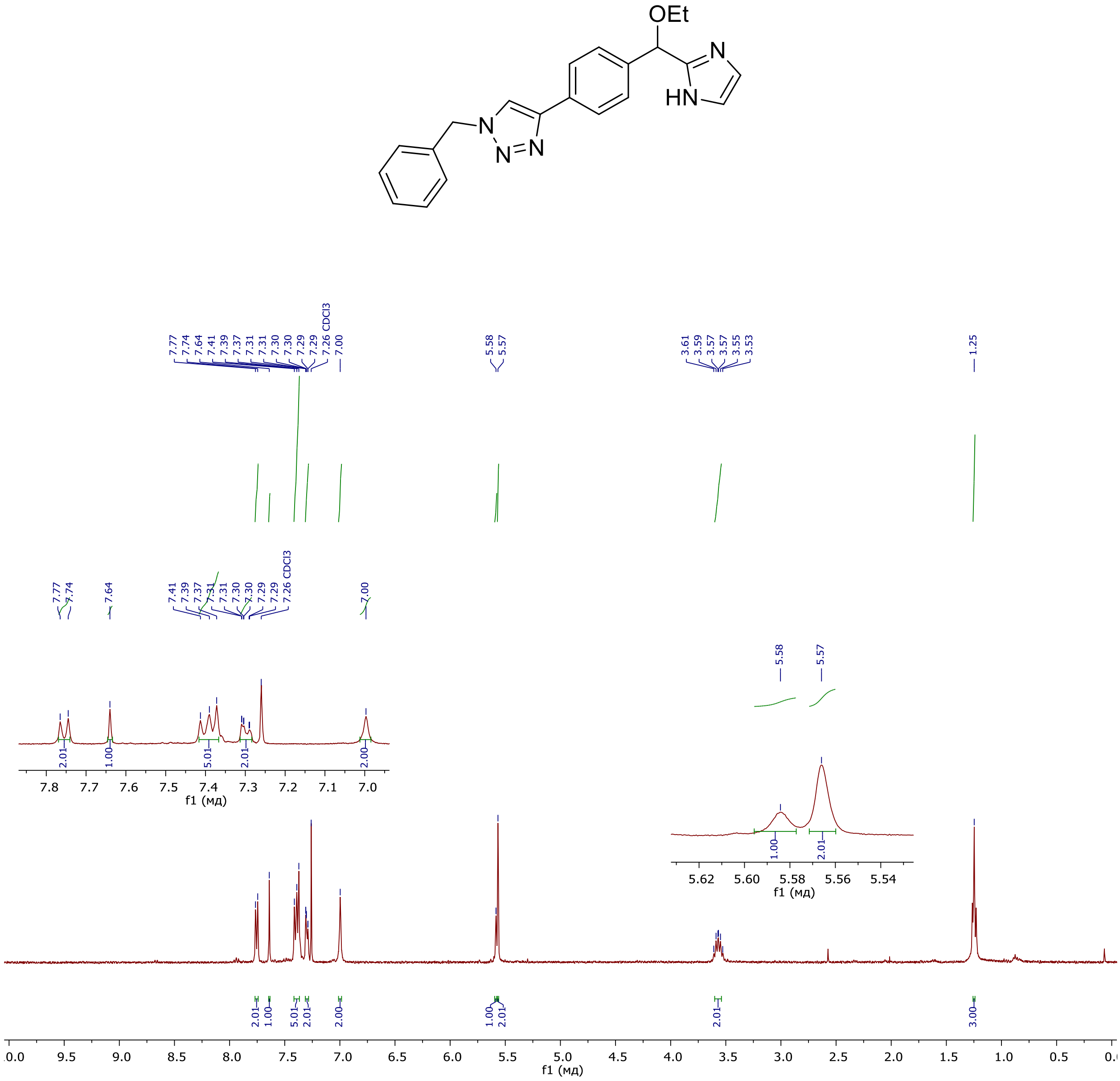

Figure S109. ^{1}H NMR (400 MHz, Chloroform-*d*) spectrum of compound **4z.**

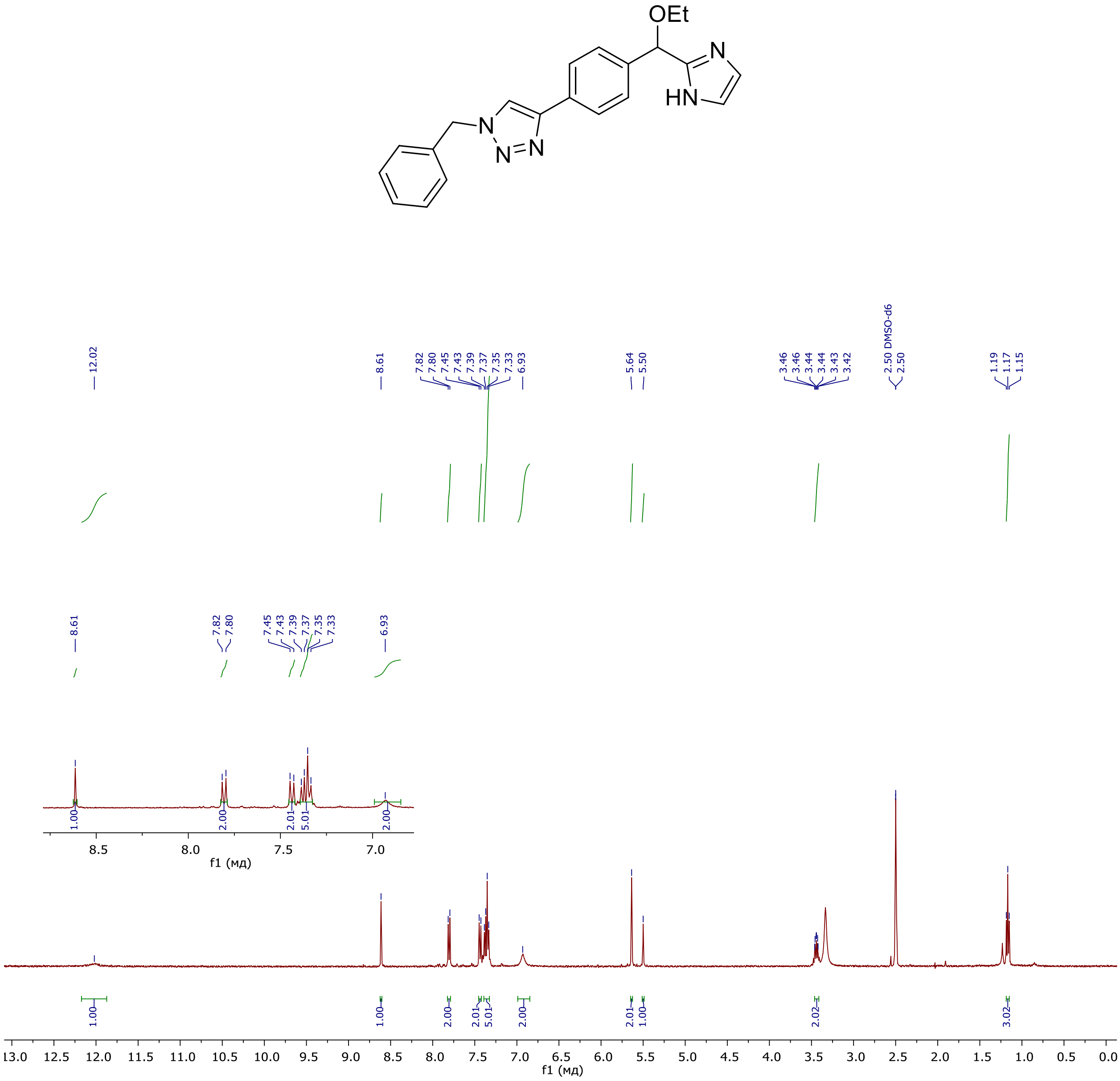

Figure S110. ^{1}H NMR (400 MHz, DMSO-d_6) spectrum of compound **4z.**

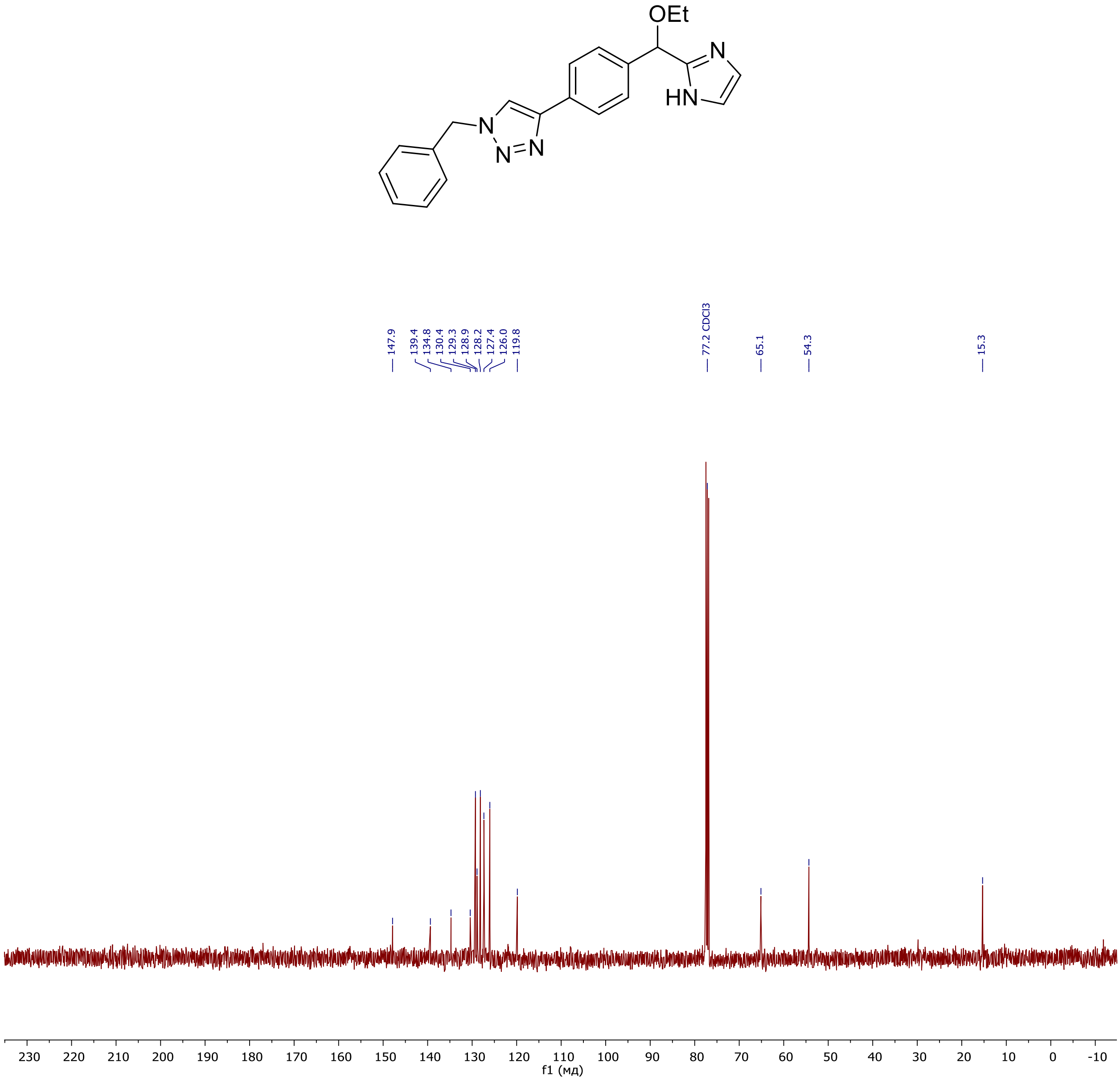

Figure S111. ^{13}C NMR (101 MHz, Chloroform-*d*) spectrum of compound **4z.**

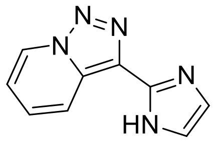

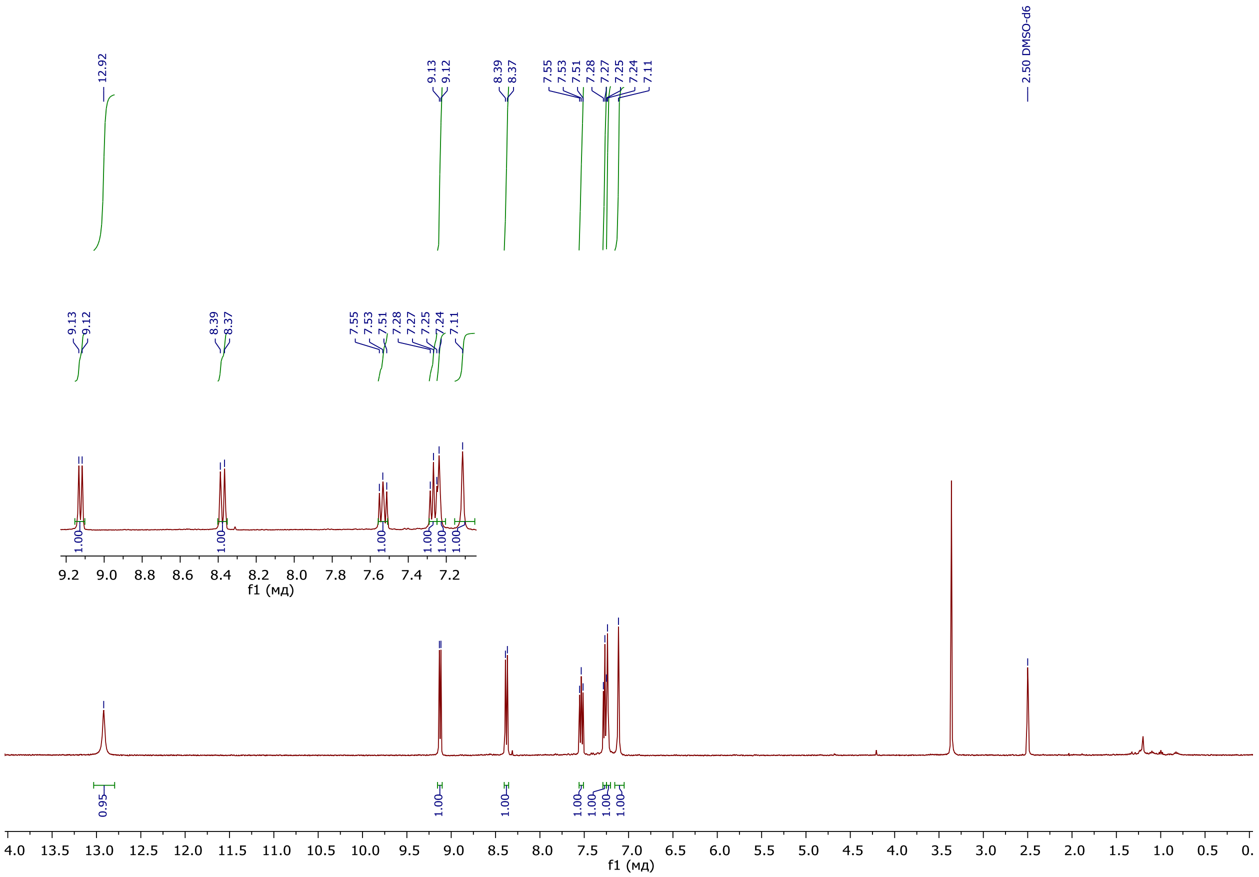

Figure S112. ^{1}H NMR (400 MHz, DMSO-d_6) spectrum of compound **5a.**

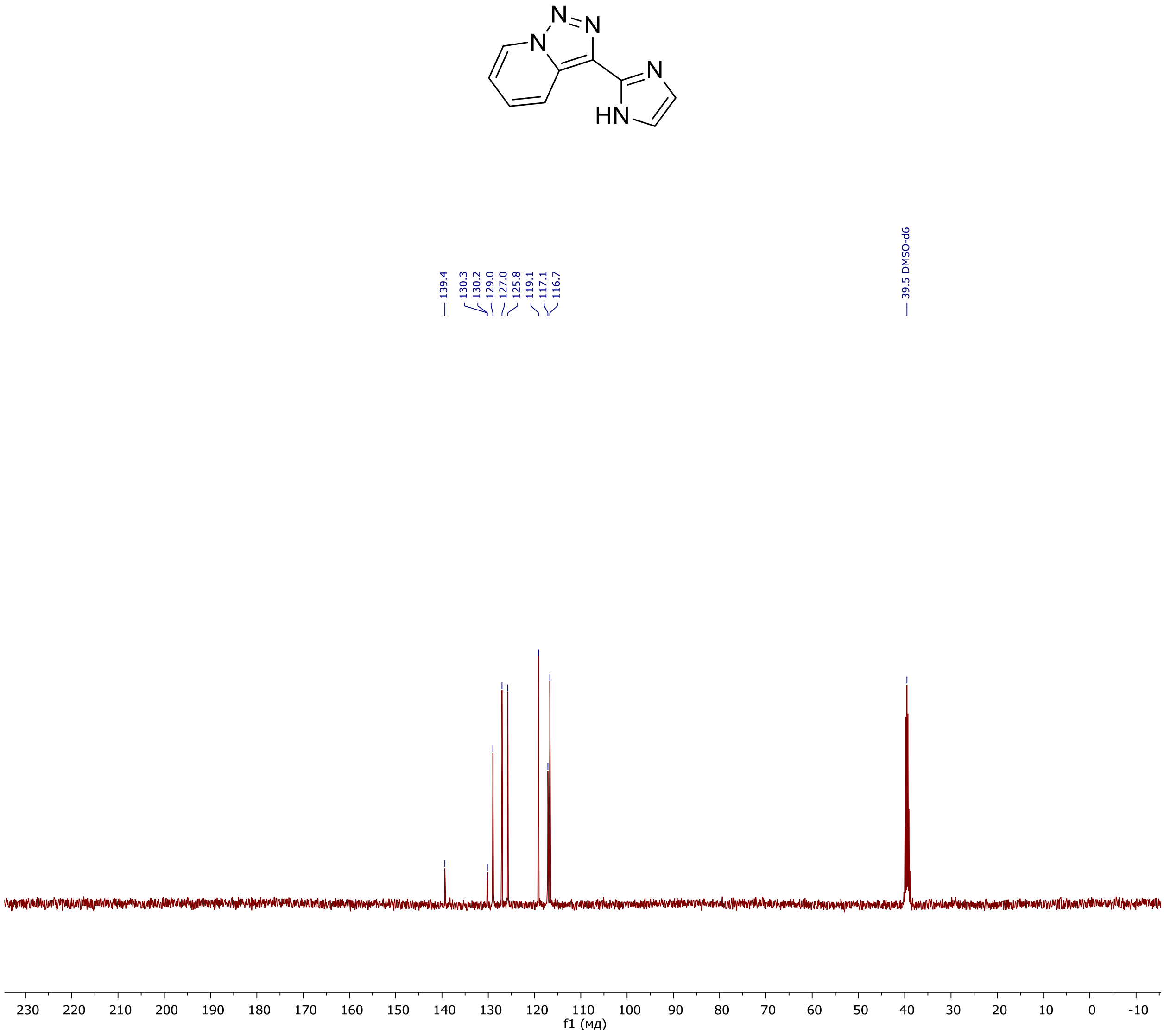

Figure S113. ^{13}C NMR (101 MHz, DMSO-d_6) spectrum of compound **5a.**
